# A cross-dehydrogenative C(*sp*^3^)−H heteroarylation via photo-induced catalytic chlorine radical generation

**DOI:** 10.1038/s41467-021-24280-9

**Published:** 2021-06-29

**Authors:** Chia-Yu Huang, Jianbin Li, Chao-Jun Li

**Affiliations:** Department of Chemistry, FRQNT Center for Green Chemistry and Catalysis, McGill University, Montreal, QC Canada

**Keywords:** Homogeneous catalysis, Synthetic chemistry methodology

## Abstract

Hydrogen atom abstraction (HAT) from C(*sp*^3^)–H bonds of naturally abundant alkanes for alkyl radical generation represents a promising yet underexplored strategy in the alkylation reaction designs since involving stoichiometric oxidants, excessive alkane loading, and limited scope are common drawbacks. Here we report a photo-induced and chemical oxidant-free cross-dehydrogenative coupling (CDC) between alkanes and heteroarenes using catalytic chloride and cobalt catalyst. Couplings of strong C(*sp*^3^)–H bond-containing substrates and complex heteroarenes, have been achieved with satisfactory yields. This dual catalytic platform features the in situ engendered chlorine radical for alkyl radical generation and exploits the cobaloxime catalyst to enable the hydrogen evolution for catalytic turnover. The practical value of this protocol was demonstrated by the gram-scale synthesis of alkylated heteroarene with merely 3 equiv. alkane loading.

## Abstract (web summary)

Hydrogen atom abstraction from C(*sp*^3^)–H bonds of naturally abundant alkanes for alkyl radical generation represents a promising yet underexplored strategy in the alkylation reaction designs. Here the authors show a photo-induced and chemical oxidant-free cross-dehydrogenative coupling between alkanes and heteroarenes using catalytic chloride and cobalt catalyst.

## Introduction

**Fig. 1:**
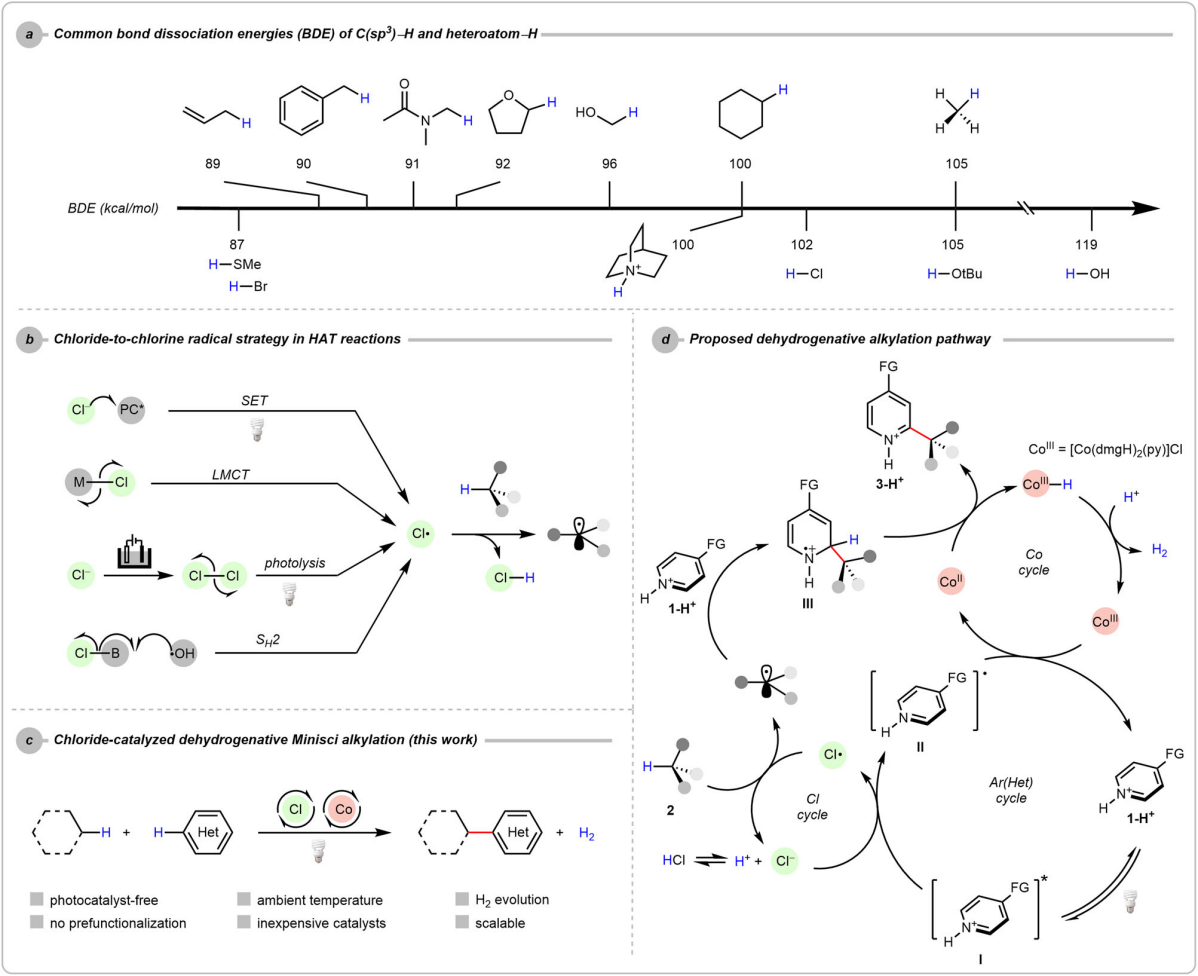
From inspiration to reaction design. **a** Bond dissociation energies (BDEs) of aliphatic C–H bonds and some protonated heteroatom species. **b** The representative chlorine radical generation method in HAT reactions. **c** The designed dehydrogenative Minisci alkylation enabled by catalytic chlorine radical generation. **d** The proposed dehydrogenative Minisci alkylation mechanism; SET single-electron transfer, LMCT ligand-to-metal charge transfer, S_H_2 bimolecular homolytic substitution, FG functional group.

Alkyl radical (R·), one of the most fundamental intermediates in organic synthesis, constitutes important approaches toward rapid molecular construction. Its generation from functionalised precursors such as aliphatic carboxylic acids, boronic reagents, halides, and others have been well-established and broadly applied as efficient paradigms in routine synthesis^1–12^. Of equal importance, direct C(*sp*^3^) radical generation from the readily available non-functionalised alkanes represents a more straightforward and sustainable method^13^. However, due to the high bond dissociation energies (BDEs)^14,15^, current strategies involving the homolysis of strong C(*sp*^3^)–H bonds mostly rely on the hydrogen atom transfer (HAT) with electrophilic heteroatom radicals, for instance, bromo-^16–18^, nitrogen-^15,19^, or oxygen-centred radicals^19–22^ (Fig. 1a). In this context, robust redox catalysts or strongly oxidising reagents are required, whereas precise control over the site selectivity among ubiquitous C(*sp*^3^)–H bonds in a molecule with broad substrate scope imposes grand challenges.

Chlorine radical (Cl·) is an efficient HAT agent that could cleave various C(*sp*^3^)–H bonds. Using chloride (Cl^−^) in Cl· generation brings several benefits in organic syntheses because it is innocuous and abundant in diverse salt forms. Nevertheless, the unfavourable chloride-to-chlorine oxidation (${{{E}}}^{^\circ }$ = 1.36 V vs NHE)^23^ and untamed reactivity of Cl· compared with other halide analogues^17,18,24–26^ make chlorine radical-promoted alkylation rarely explored. In this endeavour, few strategies have been disclosed for the efficient usage of Cl·, including (a) the direct single-electron transfer (SET) from Cl^−^ to photocatalyst under photothermal conditions^27–30^; (b) the ligand-to-metal charge transfer (LMCT), which has been employed for the coupling of alkanes and organohalides by metallophotoredox catalysis^31–36^; (c) the photolysis of in situ generated Cl_2_ via electrooxidation of HCl^37^; (d) the bimolecular homolytic substitution (S_H_2) between chloroborate and an oxy radical for the alkane borylation^38^. These pioneering examples demonstrate the potential of Cl^−^ to realise the HAT process via Cl· intermediate (Fig. 1b).

The Minisci alkylation involves the coupling between heteroarenes and nucleophilic alkyl radicals. In views of the diversity of heteroarenes and their countless applications in material science, agrochemicals, and the pharmaceutical industry, the Minisci alkylation plays a pivotal role in synthetic chemistry. Since the key of the Minisci alkylation is the alkyl radical generation, it prompts us to consider the possibility of merging the chlorine radical-mediated alkyl radical generation manifold with the Minisci alkylation, which could allow the heteroarene diversification simply through the coupling with widely available C(*sp*^3^)-H feedstocks^39^. Furthermore, an H_2_-evolving Minisci alkylation is more desirable for its highest step- and atom-economy^40,41^. Owing to the strong aliphatic C–H bonds and the net oxidative nature, excessive alkane (normally as solvent) and oxidant loadings, high temperature, or precious catalysts are not uncommon. Therefore, a catalytic cross-dehydrogenative Minisci alkylation without stoichiometric chemical oxidants is long-sought-after^39^. Our group has a long-term interest in the arene functionalisation, which was facilitated by the excited aromatics under catalyst-free conditions^42–47^. Recently, we documented a simple and clean Minisci alkylation reaction via formal dehydrative coupling of heteroarenes with alcohols. Taking advantage of the superior redox properties of the excited heteroarenes, metallophotoredox catalysts could be strategically avoided^48^.

Based on these literature precedents^49,50^, we envisioned that heteroarene itself could be an intrinsic photosensitiser, allowing efficient Cl^−^ oxidation in the Minisci reaction. In this work, a dehydrogenative Minisci alkylation using catalytic Cl^−^ under photochemical conditions is presented (Fig. 1c). Owing to the strong hydrogen atom affinity of Cl·, a wide range of inactivated and activated C(*sp*^3^)–H bonds could be arylated with good functional group tolerance and substrate diversity, and notably, with the strategic introduction of the cobaloxime catalyst, we formulate a chemical oxidant-free heteroarene alkylation protocol by releasing H_2_.

## Results

### Reaction design and optimisation

The proposed dehydrogenative alkylation pathway is depicted in Fig. 1d. It was hypothesised that the excited heteroarenes **I** could oxidise the Cl^−^ under irradiation to generate the Cl· for the aliphatic C–H abstraction from alkane **2**. The addition of the so-formed alkyl radical to another equivalent of heteroarene **1-H**^**+**^ followed by hydrogen atom removal would give the desired alkylated heteroarene **3-H**^**+**^. Meanwhile, to efficiently quench the generated radical intermediate **II**, a readily accessible cobaloxime catalyst [Co(dmgH)_2_(py)]Cl was introduced to the system^51–55^ not only to prevent the over-reduction of intermediate **II** but also to serve as a terminal oxidant for the rearomatisation of alkylated intermediate **III** though H_2_ evolution.

In our initial evaluation, 2-phenylquinoline (**1a**) and cyclohexane (**2a**) were chosen as model substrates with trifluoroacetic acid (TFA) and a catalytic amount of Bu_4_NCl and cobaloxime [Co(dmgH)_2_(py)]Cl in CHCl_3_. Delightfully, the desired coupling product **3** could be obtained in good conversion and product yield. After considerable efforts, the optimal reaction conditions yielded 80% of **3** when 20 mol% Bu_4_NCl, 5 mol% [Co(dmgH)_2_(py)]Cl, and 3 equiv. of TFA were used in CHCl_3_ under photo-irradiation for 20 h (Table 1, entry 1). During the optimisations, three key reaction components including Bu_4_NCl, [Co(dmgH)_2_(py)]Cl, and CHCl_3_^48^ were identified, all of which could potentially serve as the Cl· sources. The reaction could proceed smoothly in the presence of any of these chlorides; otherwise, no reaction occurred (entries 2-8). Importantly, the cobaloxime catalyst could improve the reaction productivity by minimising the over-reduction of quinoline to tetrahydroquinoline or other off-target decomposition reactivities. A significant solvent effect was observed in this transformation. In the chlorinated solvent CHCl_3_, heteroarene **1a** was alkylated efficiently without side reaction detected; however, when other solvents were used, poor selectivity and side product formations were often observed (e.g. in entry 4, 10% tetrahydroquinoline was obtained). Additionally, we found that the reaction could be partially suppressed by oxygen (entry 9), and no reaction occurred without light (entry 10).

**Table 1:** Key results in reaction optimisations.


Entry^a^	[Cl^−^]	[Co]	Solvent	Conversion (%)	Yield (%)
1	Bu_4_NCl	[Co(dmgH)_2_(py)]Cl	CHCl_3_	84	80
2	–	[Co(dmgH)_2_(py)]Cl	CHCl_3_	56	56
3	Bu_4_NCl	–	CHCl_3_	83	65
4	Bu_4_NCl	[Co(dmgH)_2_(py)]Cl	MeCN	100	70
5	–	[Co(dmgH)_2_(py)]Cl	MeCN	64	46
6	Bu_4_NCl	Co(dmgBF_2_)_2_	MeCN	78	69
7	–	Co(dmgBF_2_)_2_	CHCl_3_	60	58
8	–	Co(dmgBF_2_)_2_	MeCN	3	0
9^b^	Bu_4_NCl	[Co(dmgH)_2_(py)]Cl	CHCl_3_	48	41
10^c^	Bu_4_NCl	[Co(dmgH)_2_(py)]Cl	CHCl_3_	1	0

*dmg* dimethylglyoxime, *TFA* trifluoroacetic acid.

^a^Reaction conditions: **1a** (0.2 mmol), **2a** (0.6 mL), [Cl^−^] (20 mol%), [Co] (5 mol%), and TFA (0.6 mmol) in solvent (1.5 mL) under light irradiated at 20–25 °C for 20 h under N_2_. Yields were determined by ^1^H NMR using CH_2_Br_2_ as the internal standard.

^b^The reaction was conducted under air.

^c^The reaction was run in dark.

### Substrate scope

**Fig. 2:**
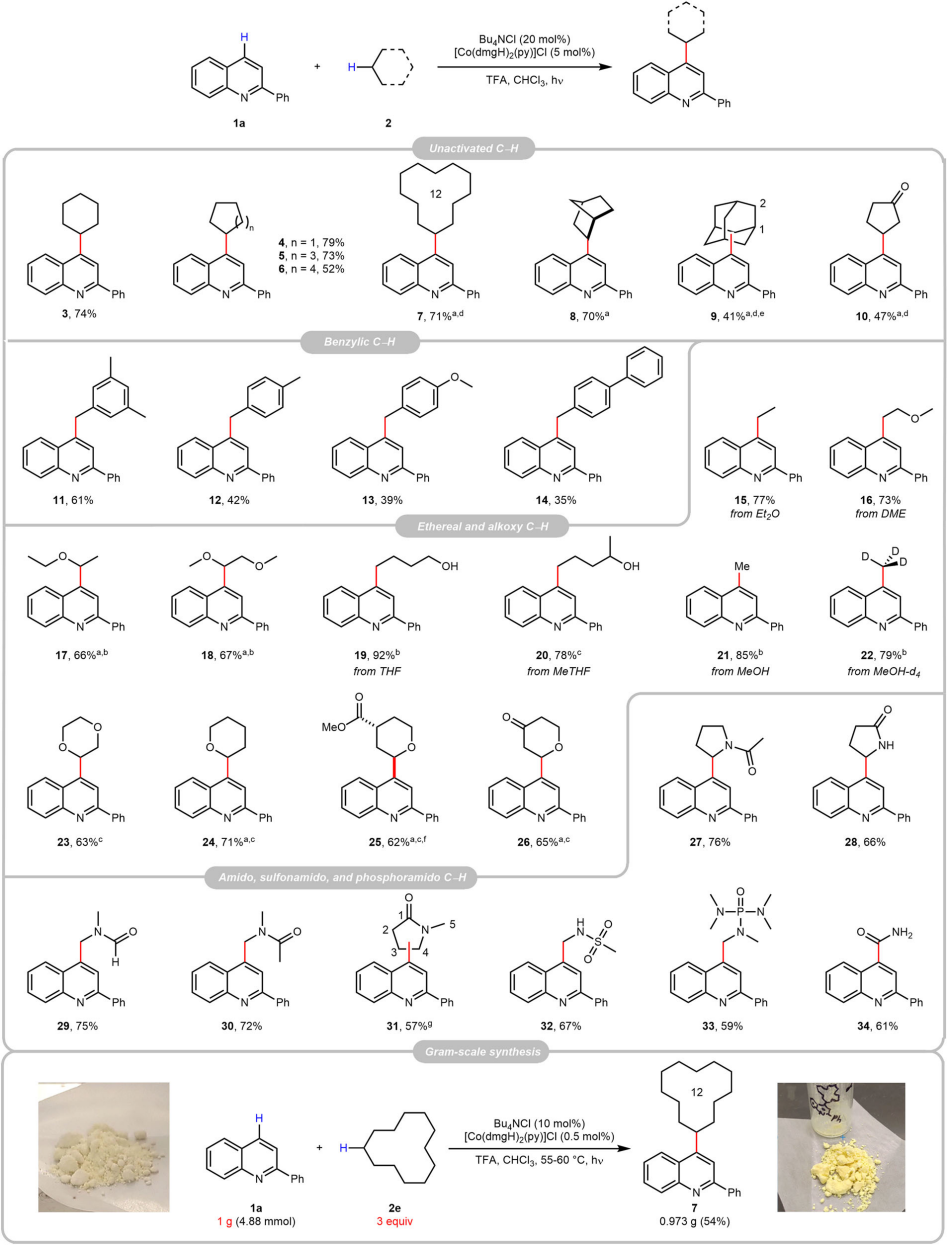
Substrate scope of alkane. Reaction conditions: **1a** (0.2 mmol), **2** (0.6 mL for liquid, or 10 equiv. for solid), Bu_4_NCl (0.04 mmol), [Co(dmgH)_2_(py)]Cl (0.01 mmol), and TFA (0.6 mmol) in CHCl_3_ (1.5 mL) under light irradiated at 20–25 °C for 20 h under N_2_, unless otherwise specified, and the yields were isolated ones. Reaction conditions for the gram-scale synthesis: **1a** (4.88 mmol), **2e** (14.6 mmol), Bu_4_NCl (0.49 mmol), [Co(dmgH)_2_(py)]Cl (0.025 mmol), and TFA (14.6 mmol) in CHCl_3_ (8 mL) under light irradiated at 55–60 °C for 72 h under N_2._^a^The reaction was run for 36 h. ^b^1.2 equiv. of TFA was used. ^c^2 equiv. of TFA was used. ^d^The reaction was heated to 55–60 °C. ^e^A mixture of congeners, C1:C2 = 1:0.9. ^f^A mixture of *trans* isomers. ^g^A mixture of congeners, C4:C5 = 1:0.26; dmg dimethylglyoxime, TFA trifluoroacetic acid, THF tetrahydrofuran, MeTHF 2-methyltetrahydrofuran, DME 1,2-dimethoxyethane.

After obtaining the optimal reaction conditions, we approached the substrate scope to different C(*sp*^3^)–H species using 2-phenylquinoline (**1a**) as the coupling partner (Fig. 2). Simple cyclic alkanes containing five to twelve carbons afforded the corresponding alkylated heteroarenes **3** to **7** in moderate to good yields, and so did bridged alkanes norbornane and adamantane (**8** and **9**). Carbonyl compound cyclopentanone was functionalised at the β-position and afforded the desired product **11**. Benzylic C–H abstractions of methylbenzene derivatives also provided benzylated heteroarenes (**11**–**14)**^56^. It was not surprising that ethylated quinoline **15** was produced as the major product with diethyl ether (Et_2_O) as the alkane source^48^; and the similar C–O cleavage was observed with 1,2-dimethoxyethane (DME) to give the deethoxylated products **16**; gratifyingly, the non-cleaved ethereal compounds **17** and **18** could be obtained by decreasing the acid amount to 1.2 to 2 equiv^37^. However, the C–O cleavage was still inevitable with tetrahydrofuran (THF) and methanol (**19**–**22**), possibly due to the relatively high ring strain and oxidation potential of the alcoholic oxygen atom. Reactions with tetrahydropyran (THP) and its analogues proceeded smoothly and gave good yields of the products **23**–**26**. α-C(*sp*^3^)–H functionalisation of amine derivatives, for example, amides, sulfonamide and phosphoramide, were all successful (**27**–**33**). Interestingly, the HAT of the *N*-methyl group of *N*,*N*-dimethylformamide (DMF) is more favourable than the formyl one and the alkylated heteroarene **29** was obtained as the major product. Few alkane substrates, for instance, cyclododecane, adamantane and cyclopentane were inert at ambient temperature, and a slight temperature increase to 55–60 °C was helpful for their transformations (**7**, **9**, and **10**). The unique regioselectivity of Cl· could be displayed from some substrates. For example, the HAT on adamantane is slightly favourable on the tertiary C–H bond in comparison to the secondary one (**9**), yet 2-methyltetrahydrofuran (MeTHF) only afforded the secondary carbon-functionalised product (**20**); C4 functionalisation of the *N*-methylpyrrolidinone (NMP) primarily occurred at the secondary C4 position rather the primary C5 one (**31**). Other than alkyl substrates, the amidation of heteroarene is also viable with formamide, which furnished the amido product **34**. Noticeably, the potential application of this reaction was briefly demonstrated by a challenging gram-scale reaction of heteroarene **1a** with 3 equiv. of unactivated alkane **3e**, and delightfully the desired product **7** could be obtained in 54% isolated yield.

**Fig. 3:**
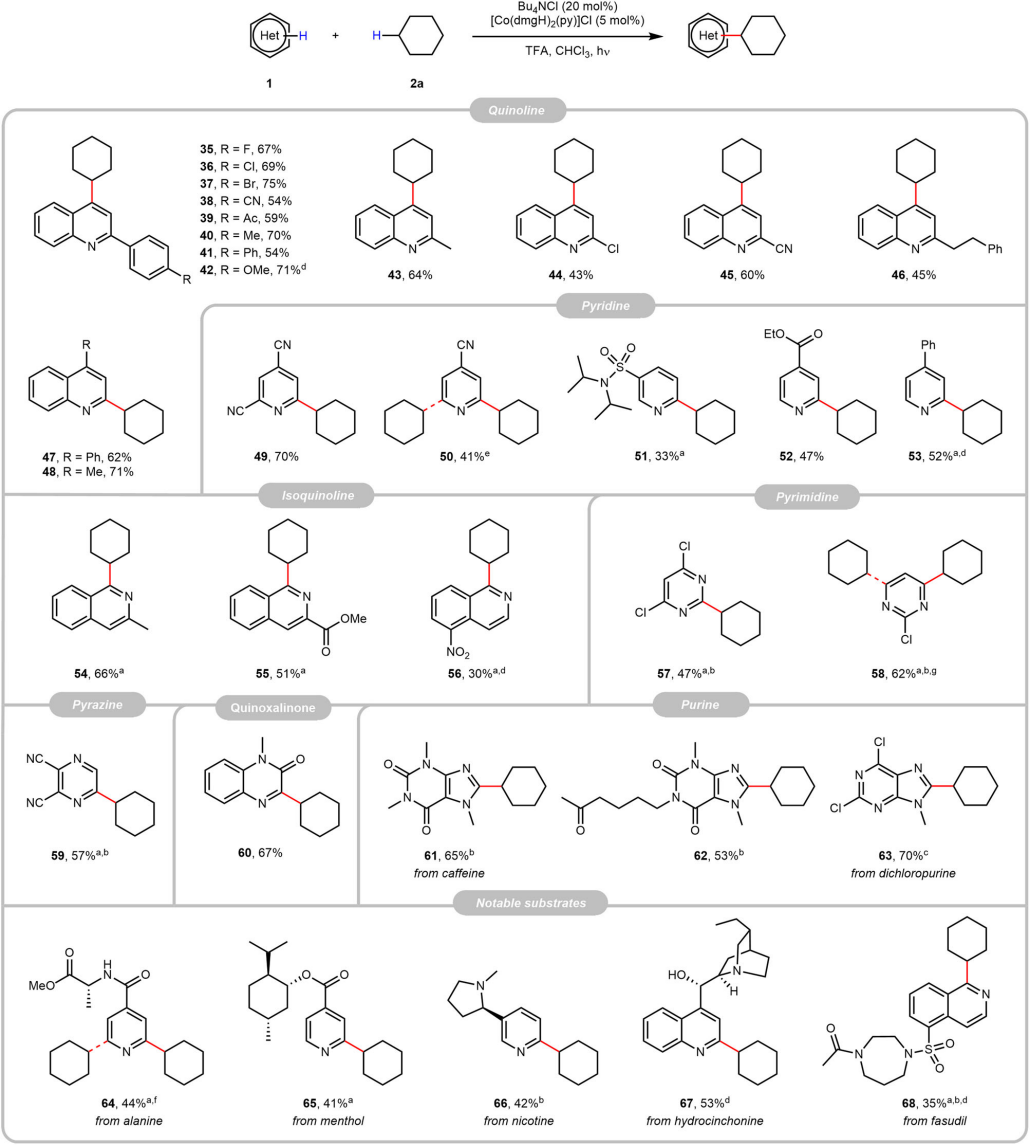
Substrate scope of heteroarenes. Reaction conditions: **1** (0.2 mmol), **2a** (0.6 mL), Bu_4_NCl (0.04 mmol), [Co(dmgH)_2_(py)]Cl (0.01 mmol), and TFA (0.6 mmol) in CHCl_3_ (1.5 mL) under light irradiated at 20–25 °C for 20 h under N_2_, unless otherwise specified, and the yields were isolated ones. ^a^The reaction was run for 36 h. ^b^4 equiv. of TFA was used. ^c^5 equiv. of TFA was used. ^d^The reaction was heated to 55–60 °C. ^e^Separable mixture, mono:di = 1.4:1. ^f^Separable mixture, mono:di = 3.9:1. ^g^Separable mixture, mono:di = 5.7:1; dmg dimethylglyoxime, TFA trifluoroacetic acid.

Further substrate and functional group tolerance were examined by coupling various heterocycles **1** with cyclohexane (**2a**). Satisfyingly, a broad variety of functional groups such as halides, cyano, acetyl, ester, amino, nitro, and sulfonamide are compatible with our method, as shown in Fig. 3. Quinoline, pyridine, and isoquinoline moieties afforded the corresponding alkylated products with up to 75% yield (**35**–**56**); other heterocycles like pyrimidine (**57** and **58**), pyrazine (**59**), quinoxalinone (**60**), and purine (**61**–**63**) were also proven to be feasible (substrates that are incompatible or with low reactivity in the reaction are listed in Supplementary Information Fig. 3). Complex heterocycles bearing different aliphatic C(*sp*^3^)–H bonds were examined, including alanine and menthol-derived pyridines (**64** and **65**), and nicotine (**66**) were successfully turned into the desired products with moderate yields. Alkylation of hydrocinchonine, a common chiral ligand candidate in synthetic chemistry, gave an appreciable yield of the product (**67**) with its hydroxy group preserved. Fasudil, a potent Rho-kinase inhibitor and vasodilator, also provided the desired product (**68**) after protecting its amino group.

### Mechanistic investigations

**Fig. 4:**
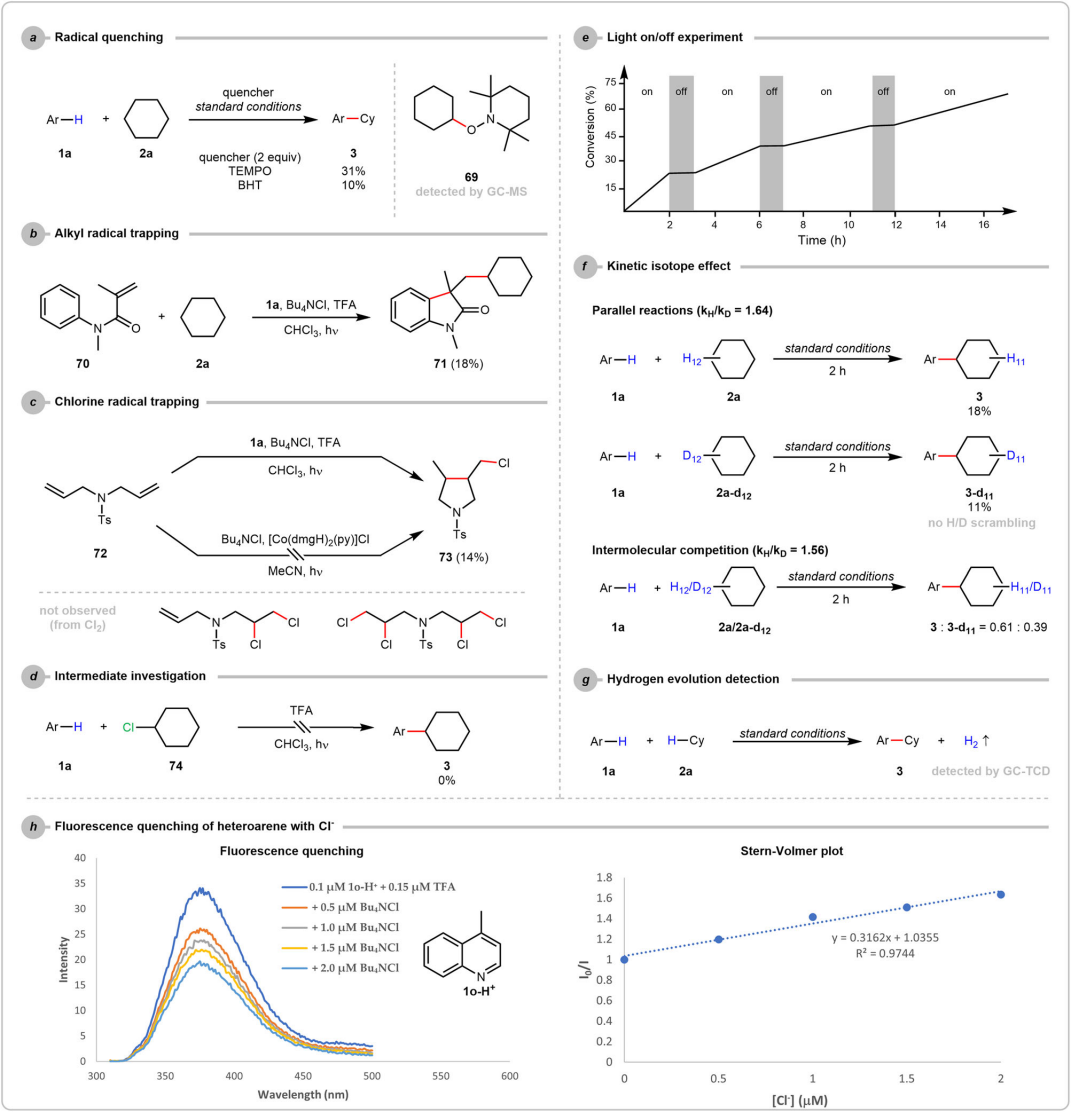
Mechanistic studies. See Supplementary Information for more detailed reaction conditions and descriptions, including **a** radical quenching, **b** R· trapping, **c** Cl· trapping, **d** intermediate investigation, **e** light on/off experiment, **f** kinetic isotope effect, **g** H_2_ detection, and **h** fluorescence quenching. Ar = 2-phenylquin-4-yl, Cy, cyclohexyl. TEMPO 2,2,6,6-tetramethylpiperidine 1-oxyl, BHT 3,5-di-*tert*-4-butylhydroxytoluene; dmg dimethylglyoxime, TFA trifluoroacetic acid.

To gain insight into this transformation, a series of experiments were conducted (See Supplementary Information for details). We found that radical quenchers, 2,2,6,6-tetramethylpiperidine 1-oxyl (TEMPO) and 3,5-di-*tert*-4-butylhydroxytoluene (BHT), significantly suppressed the product formation. Noticeably, a radical adduct **69** was detected by GC-MS in the case of TEMPO, which indicated an alkyl radical-involved mechanistic scenario (Fig. 4a). The involvement of R· was also evidenced by a radical trapping experiment by subjecting the alkene **70** to our alkane arylation reaction with heteroarene **1a** and Bu_4_NCl (Fig. 4b), in which a cycloalkylated product **71** was isolated. All these results supported the presence of R· in the plausible mechanism. To probe more details of the chlorine species, a diallyl sulfonamide **72** was submitted to the alkylation reaction of **1a** with a stoichiometric amount of Bu_4_NCl. As expected, a cyclochlorinated compound **73** was isolated, suggesting the formation of Cl· in our case (Fig. 4c). According to some literature^37,57^, chlorine (Cl_2_) might not be an active intermediate in our system as the characteristic alkenyl dichlorination product**s** were not observed. On the contrary, no cyclochlorination occurred when the same diallyl compound **72** was irradiated with Bu_4_NCl and cobaloxime catalyst in the absence of heteroarene and chlorinated solvent, indicating that the LMCT-induced Cl· generation by the cobaloxime catalyst might not be operative in this reaction. Also, replacing cyclohexane (**2a**) with cyclohexyl chloride (**74**) gave no desired product, indicating that **74** is not an active intermediate in generating the cyclohexyl radical (Fig. 4d). The light on-and-off experiment showed that continuous irradiation was essential for the product formation (Fig. 4e), and the low reaction quantum yield (<1) does not support the radical chain process (see Supplementary Information). Besides, both parallel and competing kinetic isotope effects were examined, giving *k*_H_/*k*_D_ = 1.64 and 1.56, respectively. This suggested that the alkyl C–H cleavage might not be the rate-determining step (Fig. 4f)^8^. Moreover, the H_2_ evolution was confirmed by the GC-TCD analysis (Fig. 4g). Lastly, from the fluorescence quenching experiments, strong interaction between excited heteroarene **1a** and Cl^−^ was observed only in the presence of an acid, emphasising the indispensable role of TFA in protonating the heteroarene for Cl^−^ oxidation (Fig. 4h, see Supplementary Information for details). Taken together, these experiments provide support for the surmised radical generation (R· and Cl·) and H_2_ evolution reaction pathways. More detailed mechanistic studies are still ongoing in our lab. Finally, as a logical extension based on the current mechanistic framework, we believed that this coupling reaction could be realised under visible light irradiation with the catalytic introduction of a more conjugated heteroarene. While this dehydrogenative coupling reaction was proven unsuccessful with most of the commercially available photosensitizers, 43% of the alkylated heteroarene **3** could be furnished with 5 mol% of 2,4-diphenylquinoline as the photocatalyst (See supplementary Information Table 3)^50^. This encouraging result could enlighten more visible light-promoted Cl· generation strategies for coupling reactions.

## Discussion

We have developed an approach toward photo-induced dehydrogenative Minisci alkylation, which couples a wide range of heterocycles with strong aliphatic C–H bonds, while the merger of catalytic Cl· generation and hydrogen evolution grants simple and chemical oxidant-free reaction conditions. Mechanistic studies were revealed to support the direct Cl· generation via the SET between Cl^−^ and excited heteroarene. As a powerful platform, complex molecules bearing diverse C(*sp*^3^)–H patterns and functional groups were feasible for alkylations, and a large-scale synthesis was demonstrated with low alkane loading. We believed that this work would advance the excited arene chemistry and inspire future cross-dehydrogenative coupling designs.

## Methods

### General experimental procedure for the cross-dehydrogenative coupling of alkanes and heteroarenes

To a 10 mL pyrex microwave vial equipped with a Teflon-coated magnetic stirring bar was added heteroarene **1** (0.2 mmol) and [Co(dmgH)_2_(py)]Cl (4 mg, 0.01 mmol). The tube was sealed with a rubber septum, evacuated and backfilled with argon three times before alkane **2** (0.6 mL for liquids. For solid alkanes, 2.0 mmol of alkane was added to the vial before backfilled with argon) was injected into the vial. The mixture was then sequentially added Bu_4_NCl (11.1 mg, 0.04 mmol), CHCl_3_ (1.5 mL), and TFA (46 μL, 0.6 mmol) in the glove box and then sealed with an aluminium cap with a septum. The reaction vial was taken out from the glove box and stirred at 20–25 °C or 55–60 °C under light irradiation of a 300 W Xe lamp with a 280 nm filter for 20–36 h, as the time indicated. After the reaction was completed, the reaction was basified with sat NaHCO_3_ (aq), extracted with EtOAc, and filtered through a short pad of MgSO_4_. The volatiles were removed under reduced pressure to obtain the crude product. The isolated product was obtained by preparative thin-layer chromatography.

## Supplementary information

Supplementary Information

## Data Availability

Most data generated or analysed during this study are included in this published article and its Supplementary Information. All data are available from the author(s) upon reasonable request.
