# Supplementary material for: A cross-dehydrogenative C(sp3)−H heteroarylation via photo-induced catalytic chlorine radical generation
Source: Nat Commun. 2021 Jun 29;12:4010. doi: 10.1038/s41467-021-24280-9 (PMC8241867; doi:10.1038/s41467-021-24280-9)

# Supplementary information for
# A Dehydrogenative C(sp$^3$)–H Heteroarylation via Photo-induced Catalytic Chlorine Radical Generation

Chia-Yu Huang‡, Jianbin Li‡, and Chao-Jun Li*

*Department of Chemistry and FRQNT Centre for Green Chemistry and Catalysis, McGill University, 801 Sherbrooke St. W., Montreal, Quebec H3A 0B8, Canada*

*Email: cj.li@mcgill.ca*

## Table of Contents

**1. Supplementary Notes ........ S2**

**1-1. General Information ........ S2**

**2. Supplementary Methods ........ S5**

**2-1. Coupling of heteroarenes and liquid hydrocarbons ........ S5**

**2-2. Coupling of heteroarenes and solid hydrocarbons ........ S5**

**2-3. Gram-scale synthesis of 7 ........ S6**

**3. Supplementary Discussion ........ S6**

**3-1. Reaction optimizations ........ S6**

**3-1-1. Preliminary investigation on the halide source ........ S6**

**3-1-2. Selected optimization entries ........ S7**

**3-1-3. Unsuccessful substrates (yields <30%) ........ S8**

**3-2. Cross-dehydrogenative coupling under visible light ........ S8**

**3-3. Mechanistic studies ........ S9**

**3-3-1. Radical quenching experiment ........ S9**

**3-3-2. Alkyl radical trapping experiment ........ S10**

**3-3-3. Chlorine radical trapping experiment ........ S10**

**3-3-4. Possible intermediate investigation ........ S12**

**3-3-5. Light on/off experiment ........ S12**

**3-3-6. Quantum yield measurement ........ S13**

**3-3-7. Kinetic isotope effect (KIE) experiment ........ S14**

**3-3-8. Detection of the $H_2$ evolution ........ S15**

**3-3-9. UV-vis and fluorescence quenching ........ S15**

**3-3-10. C(sp$^3$)–H selectivity test ........ S18**

**4. Characterization data for compounds ........................................................................................S18**
**5. Supplementary References.........................................................................................................S43**
**6. NMR spectra.................................................................................................................................S45**

# 1. Supplementary Notes

## 1-1. General Information

Solvents used in this work were dried over 4 Å molecular sieves (beads, 8-12 mesh) and degassed by purging with argon for 30 min. The 4 Å molecular sieves were purchased from Sigma-Aldrich chemical company and were freshly activated in the oven for 12 h at 380 °C before use. Reagents were purchased from Sigma-Aldrich, Combi-Blocks, TCI America, Oakwood, and Fisher scientific chemical companies and were used without further purification unless otherwise specified. For the rest, they were well documented and readily accessed by laboratory synthesis according to the literature reports. Nuclear magnetic resonance (NMR) spectra, including $^1$H NMR, $^{13}$C NMR, $^{19}$F NMR, and $^{31}$P NMR were recorded on Bruker 500 MHz spectrometers, which uses the deuterium lock signal to reference the spectra. The solvent residual peaks, e.g., of chloroform ($CDCl_3$: δ 7.28 ppm and δ 77.02 ppm), were used as references. Data are reported as follows: multiplicity (s = singlet, d = doublet, t = triplet, q = quartet, quint = quintet, m = multiplet, dd = doublet of doublet, etc), coupling constant (J/Hz) and integration. All NMR spectra were recorded at room temperature. Gas chromatography-mass spectroscopy (GC-MS) was obtained from the Agilent gas chromatography-mass spectroscopy system with helium (He) as the carrier gas. High-resolution mass spectrometry (HRMS) was conducted by using electro-spraying ionization (ESI) performed by McGill University on a Thermo-Scientific Exactive Orbitrap. Protonated molecular ions $(M+H)^+$ or sodium adducts $(M+Na)^+$ were used for empirical formula confirmation. Hydrogen gas ($H_2$) was analyzed by gas chromatography-thermal conductivity detector (GC-TCD, Agilent 6890N Network Gas Chromatograph, argon (Ar) as the carrier gas). Ultraviolet-visible (UV-vis) spectrometry was performed by Agilent Cary 5000 series UV-vis-NIR spectrometer. Fluorescence quenching was performed on VARIAN CARY Eclipse fluorescence spectrophotometer. All reactions are stirred magnetically unless otherwise specified. Short packed column chromatography was performed with E. Merck silica gel 60 (230–400 mesh). The experiments were conducted in sealed 10 mL pyrex tubes, or 25 mL Schlenk tube for gram-scale synthesis. The experiments under light irradiation were performed using a low pressure 300 W Xe lamp (from Atlas Specialty Lighting, with PE300BF light bulb from Excelitas. For details see https://www.yumpu.com/en/document/read/19618941/cermaxr-xenon-short-arc-lamps-pe300bf-excelitas-technologies) equipped with a water bath (Chemglass Jacketed Beaker, GC-1107-12) for efficient temperature maintenance, and all the reactions were conducted under an inert atmosphere in sealed tubes unless otherwise noted. The UV box was equipped with 6 x 8 W 254 nm UVC lamps (Luzchem Research Inc, with LZC-UVC lamps).

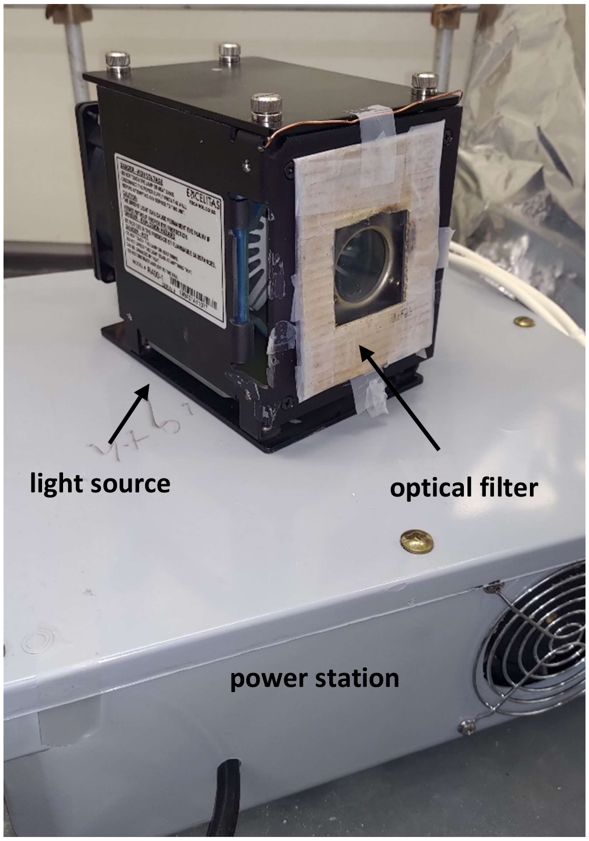


**Supplementary Figure 1a | The Xe lamp used in the general procedures, with a 280, 295, 345, or 395 nm optical filter to block wavelengths below the indicated ones.**

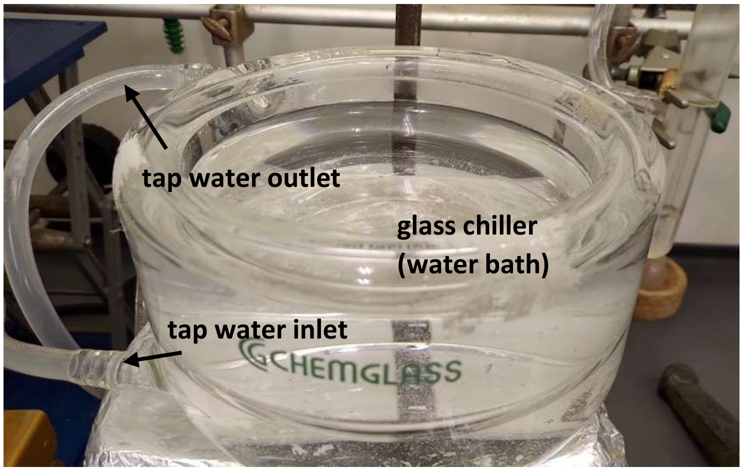


**Supplementary Figure 1b | The water bath used in the general procedures, with cooling water to maintain at 20-25 °C, or heating with a hotplate to reach 55-60 °C.**

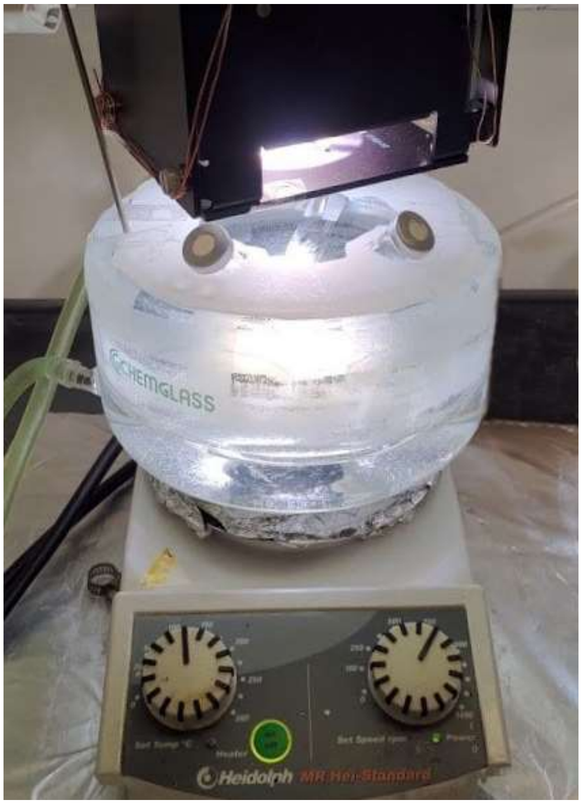


**Supplementary Figure 1c | The reaction setup for the general procedures. The light source is 3 to 5 cm away from the water bath.**

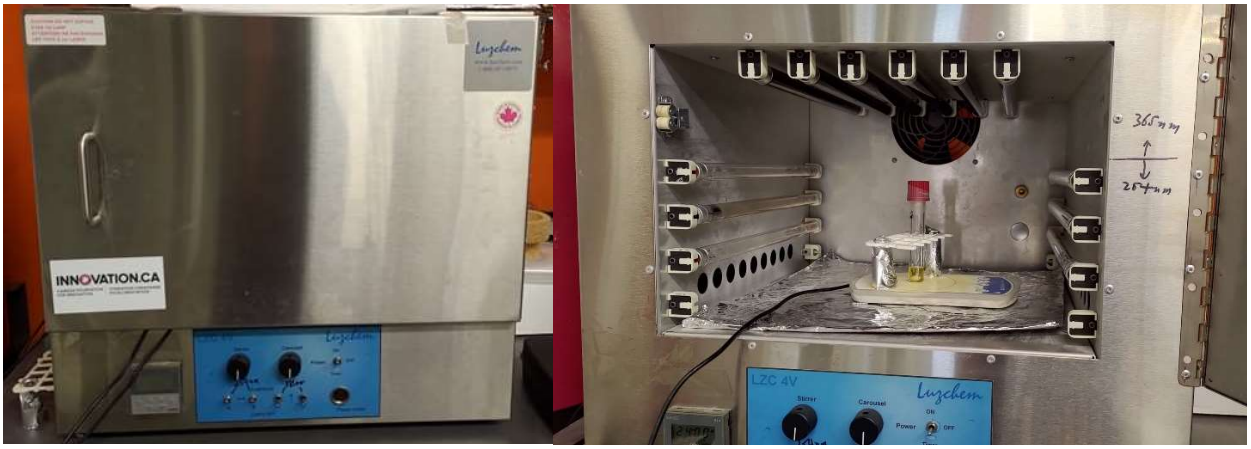


**Supplementary Figure 1d | The UV box used for quantum yield determination. The box is equipped with 6 × 8 W 254 nm lamps, cooling fans, and a stirring plate.**

# 2. Supplementary Methods

## 2-1. Coupling of heteroarenes and liquid hydrocarbons

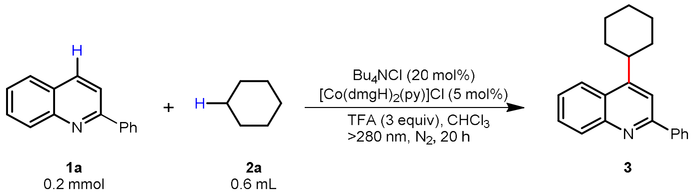


**General procedure A**. The preparation of **3** is representative and applicable to all the coupling of heteroarenes and liquid hydrocarbons unless otherwise noted. To a 10 mL pyrex microwave tube equipped with a Teflon-coated magnetic stirring bar was added heteroarene **1** (0.2 mmol) and $[Co(dmgH)_2(py)]Cl$ (4 mg, 0.01 mmol). The tube was sealed with a rubber septum, evacuated and backfilled with argon three times before alkane **2** (0.6 mL for all liquids) was injected into the vial. The mixture was then sequentially added $Bu_4NCl$ (11.1 mg, 0.04 mmol), $CHCl_3$ (1.5 mL), and TFA (46 µL, 0.6 mmol) in the glovebox ($Bu_4NCl$ can be prepared as a $CHCl_3$ stock solution), and then sealed with an aluminium cap with a septum. The reaction vial was taken out from the glovebox and stirred at 20-25 °C under the irradiation of a 300 W Xe lamp with a 280 nm filter for 20-36 h, as the time indicated. After the reaction was completed, the reaction was basified with sat $NaHCO_3$ (aq), extracted with EtOAc, filtered through a short pad of $MgSO_4$. The volatiles were removed under reduced pressure to obtain the crude product. The product was isolated by preparative thin-layer chromatography.

## 2-2. Coupling of heteroarenes and solid hydrocarbons

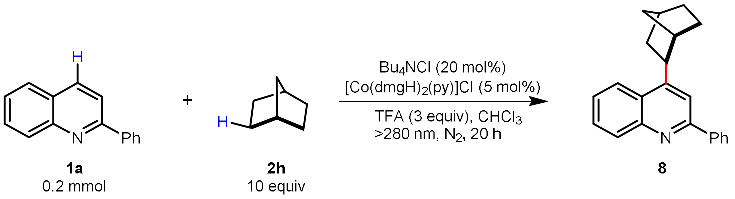


**General procedure B**. The preparation of **8** is representative and applicable to all the coupling of heteroarenes and solid hydrocarbons unless otherwise noted. To a 10 mL pyrex microwave tube equipped with a Teflon-coated magnetic stirring bar was added heteroarene **1** (0.2 mmol), alkane **2** (2 mmol), and $[Co(dmgH)_2(py)]Cl$ (4 mg, 0.01 mmol). The tube was sealed with a rubber septum, evacuated and backfilled with argon three times. The mixture was then sequentially added $Bu_4NCl$ (11.1 mg, 0.04 mmol), $CHCl_3$ (1.5 mL), and TFA (46 µL, 0.6 mmol) in the glovebox ($Bu_4NCl$ can be prepared as a $CHCl_3$ stock solution), and then sealed with an aluminium cap with a septum. The reaction vial was taken out from the glovebox and stirred at 20-25 °C under the irradiation of a 300 W Xe lamp with a 280 nm filter for 20-36 h, as the time indicated. After the reaction was completed, the reaction was basified with sat $NaHCO_3$ (aq), extracted with EtOAc, filtered through a short pad of $MgSO_4$. The volatiles were removed under reduced pressure to obtain the crude product. The product was isolated by preparative thin-layer chromatography.

## 2-3. Gram-scale synthesis of 7

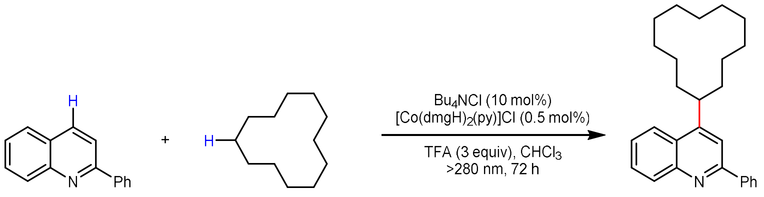


**1a** 1 g | **2e** 3 equiv | **7** 0.975 g (54%)

**Gram scale synthesis of 7**. To a 25 mL Schlenk tube equipped with a Teflon-coated magnetic stirring bar was added heteroarene **1a** (1 g, 4.88 mmol) alkane **2e** (2.46 g, 14.6 mmol), and [Co(dmgH)$_2$(py)]Cl (9.7 mg, 0.024 mmol). The tube was evacuated and backfilled with argon three times. The mixture was then sequentially added Bu$_4$NCl (136 mg, 0.98 mmol), CHCl$_3$ (8 mL), and TFA (1.12 mL, 14.6 mmol) in the glovebox, and then sealed with a screw cap. The reaction vial was taken out from the glovebox and stirred at 55-60 °C under the irradiation of two 300 W Xe lamp with a 280 nm filter for 72 h. After the reaction was completed, the reaction was basified with sat NaHCO$_3$ (aq), extracted with EtOAc, and dried with MgSO$_4$. The volatiles were removed under reduced pressure to obtain the crude product. The product **7** was isolated by column chromatography using EtOAc/Hexane eluent (0-3%) as a pale-yellow solid (0.975 g, 54%).

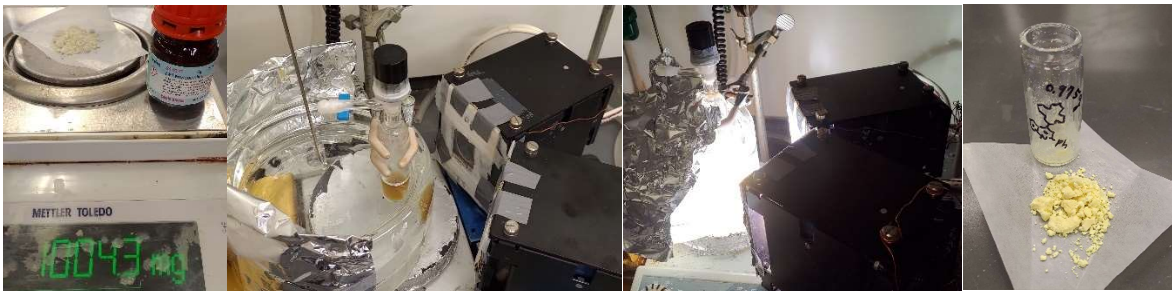


# 3. Supplementary Discussion

## 3-1. Reaction optimizations

### 3-1-1. Preliminary investigation on the halide source

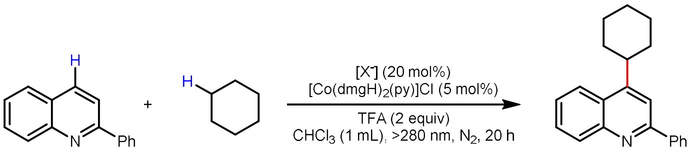


**1a** 0.2 mmol | **2a** 0.6 mL | **3**

| [X⁻] | 3 |
|---|---|
| Bu$_4$NCl | 66% |
| Bu$_4$NBr | 0% |
| Bu$_4$NI | 0% |

**Supplementary Figure 2 | Preliminary investigation of the halide source.** Tetrabutyl ammonium chloride, bromide, and iodide salts were added to the reaction, and only the chloride salt gave the desired product **3**.

## 3-1-2. Selected optimization entries

**Supplementary Table 1 | Key results in reaction optimizations**

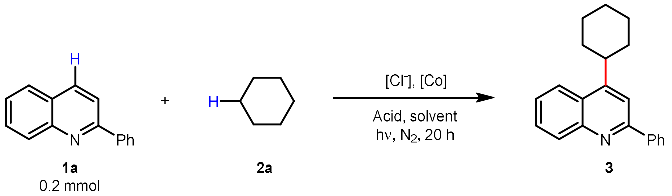


| Entry | 2a (mL) | [Cl⁻] (mol%) | [Co] (mol%) | Acid (equiv) | Solvent (mL) | hν (nm) | 3/1 (%)[a] |
|---|---|---|---|---|---|---|---|
| 1 | 0.2 | $Bu_4NCl$ (10) | 5 | TFA (2) | $CHCl_3$ (1.0) | >280 | 59/40 |
| 2 | 0.2 | $Bu_4NCl$ (10) | 10 | TFA (2) | $CHCl_3$ (1.0) | >280 | 53/47 |
| 3 | 0.2 | $Bu_4NCl$ (10) | 5 | TFA (2) | $CHCl_3$ (1.0) | >280 | 64/33 |
| 4 | 0.2 | $Bu_4NCl$ (10) | 2.5 | TFA (2) | $CHCl_3$ (1.0) | >280 | 61/29 |
| 5 | 0.2 | $Bu_4NCl$ (10) | - | TFA (2) | $CHCl_3$ (1.0) | >280 | 49/18 |
| 6 | 0.4 | $Bu_4NCl$ (10) | 5 | TFA (2) | $CHCl_3$ (1.0) | >280 | 62/34 |
| 7 | 0.6 | $Bu_4NCl$ (10) | 5 | TFA (2) | $CHCl_3$ (1.0) | >280 | 66/33 |
| 8 | 1.0 | $Bu_4NCl$ (10) | 5 | TFA (2) | $CHCl_3$ (1.0) | >280 | 42/55 |
| 9 | 0.6 | $Bu_4NCl$ (10) | 5 | TFA (2) | MeCN (1.0) | >280 | 49/45 |
| 10 | 0.6 | $Bu_4NCl$ (10) | 5 | TFA (2) | $C_6H_6$ (1.0) | >280 | 32/66 |
| 11 | 0.6 | $Bu_4NCl$ (10) | 5 | TFA (2) | $CH_2Cl_2$ (1.0) | >280 | 65/35 |
| 12 | 0.6 | $Bu_4NCl$ (10) | 5 | TFA (2) | $CCl_4$ (1.0) | >280 | 0/99 |
| 13 | 0.6 | $Bu_4NCl$ (10) | 5 | TFA (2) | EtOAc (1.0) | >280 | 42/39 |
| 14 | 0.6 | $Bu_4NCl$ (10) | 5 | TFA (2) | $CHCl_3$ (1.0) | >295 | 53/46 |
| 15 | 0.6 | $Bu_4NCl$ (10) | 5 | TFA (2) | $CHCl_3$ (1.0) | >345 | 41/59 |
| 16 | 0.6 | $Bu_4NCl$ (10) | 5 | TFA (2) | $CHCl_3$ (1.0) | >395 | 1/99 |
| 17 | 0.6 | - | 5 | TFA (2) | $CHCl_3$ (1.0) | >280 | 44/56 |
| 18 | 0.6 | NaCl (20) | 5 | TFA (2) | $CHCl_3$ (1.0) | >280 | 51/47 |
| 19 | 0.6 | $NiCl_2$ (20) | 5 | TFA (2) | $CHCl_3$ (1.0) | >280 | 40/52 |
| 20 | 0.6 | $PdCl_2$ (20) | 5 | TFA (2) | $CHCl_3$ (1.0) | >280 | 24/73 |
| 21 | 0.6 | $FeCl_2$ (20) | 5 | TFA (2) | $CHCl_3$ (1.0) | >280 | 11/60 |
| 22 | 0.6 | $CoCl_2$ (20) | 5 | TFA (2) | $CHCl_3$ (1.0) | >280 | 6/94 |
| 23 | 0.6 | $CoCl_2$ (20) | - | TFA (2) | $CHCl_3$ (1.0) | >280 | 33/61 |
| 24 | 0.6 | CuCl (20) | 5 | TFA (2) | $CHCl_3$ (1.0) | >280 | 29/67 |
| 25 | 0.6 | $Bu_4NCl$ (10) | 5 | TFA (2) | $CHCl_3$ (0.5) | >280 | 65/34 |
| 26 | 0.6 | $Bu_4NCl$ (10) | 5 | TFA (2) | $CHCl_3$ (1.5) | >280 | 77/19 |
| 27 | 0.6 | $Bu_4NCl$ (10) | 5 | TFA (2) | $CHCl_3$ (2.0) | >280 | 74/23 |
| 28 | 0.6 | $Bu_4NCl$ (10) | 5 | - | $CHCl_3$ (1.5) | >280 | 38/60 |
| 29 | 0.6 | $Bu_4NCl$ (10) | 5 | TFA (1.5) | $CHCl_3$ (1.5) | >280 | 56/43 |
| 30 | 0.6 | $Bu_4NCl$ (10) | 5 | TFA (3) | $CHCl_3$ (1.5) | >280 | 80(74)/16 |
| 31 | 0.6 | $Bu_4NCl$ (10) | 5 | TFA (4) | $CHCl_3$ (1.5) | >280 | 83/16 |
| 32 | 0.6 | $Bu_4NCl$ (10) | 5 | TfOH (1.2) | $CHCl_3$ (1.5) | >280 | 15/84 |
| 33 | 0.6 | - | 5 | HCl (2) | $CHCl_3$ (1.5) | >280 | 2/98 |

[a] Yields were determined by $^1$H NMR using $CH_2Br_2$ as the internal standard. [Co], [Co(dmgH)$_2$(py)]Cl; dmg, dimethylglyoxime; TFA, trifluoroacetic acid.

**Supplementary Table 2 | Key results in reaction optimizations (2)**

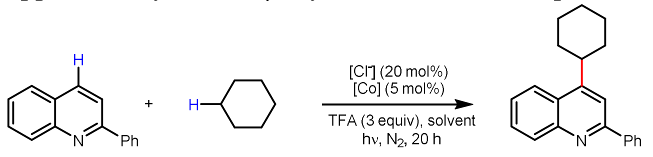


**1a** 0.2 mmol    **2a** 0.6 mL    **3**

| Entry | [Cl⁻] | [Co] | Solvent | hν (nm) | Cl in system? | **3**/**1** (%)[a] |
|---|---|---|---|---|---|---|
| 1 | $Bu_4NCl$ | $[Co(dmgH)_2(py)]Cl$ | $CHCl_3$ | >280 | yes | 80/16[d] |
| 2 | - | $[Co(dmgH)_2(py)]Cl$ | $CHCl_3$ | >280 | yes | 56/44 |
| 3 | $Bu_4NCl$ | - | $CHCl_3$ | >280 | yes | 65/17 |
| 4 | - | $[Co(dmgH)_2(py)]Cl$ | MeCN | >280 | yes | 46/36 |
| 5 | $Bu_4NCl$ | $[Co(dmgH)_2(py)]Cl$ | MeCN | >280 | yes | 70/0 |
| 6 | $Bu_4NCl$ | $Co(dmgBF_2)_2$ | MeCN | >280 | yes | 69/22 |
| 7 | - | $Co(dmgBF_2)_2$ | MeCN | >280 | no | 0/97 |
| 8 | - | $Co(dmgBF_2)_2$ | $CHCl_3$ | >280 | yes | 58/40 |
| 9[b] | $Bu_4NCl$ | $[Co(dmgH)_2(py)]Cl$ | $CHCl_3$ | >280 | yes | 41/52 |
| 10[c] | $Bu_4NCl$ | $[Co(dmgH)_2(py)]Cl$ | $CHCl_3$ | - | yes | 0/99 |
| 11 | $Bu_4NCl$ | $[Co(dmgH)_2(py)]Cl$ | $CHCl_3$ | >295 | yes | 64/33 |
| 12 | $Bu_4NCl$ | $[Co(dmgH)_2(py)]Cl$ | $CHCl_3$ | >345 | yes | 53/46 |
| 13 | $Bu_4NCl$ | $[Co(dmgH)_2(py)]Cl$ | $CHCl_3$ | >395 | yes | 3/95 |

[a] Yields were determined by $^1H$ NMR using $CH_2Br_2$ as the internal standard. [b] Under air. TFA, trifluoroacetic acid. [c] The reaction was run in dark. [d] The isolated **3** was obtained in 74% yield; dmg, dimethylglyoxime; TFA, trifluoroacetic acid.

## 3-1-3. Unsuccessful substrates (yields <30%)

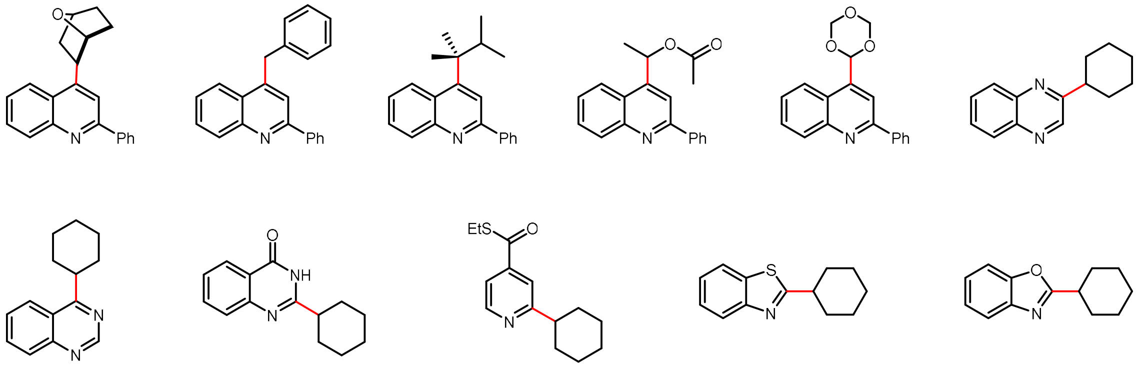


**Supplementary Figure 3 | Unsuccessful substrates.** The listed substrates were suffered from decomposition or low reactivity under photo and photothermal conditions, which did not undergo further optimizations.

## 3-2. Cross-dehydrogenative coupling under visible light

As a logical extension, we believed that this coupling reaction could be realised under visible light irradiation with the catalytic introduction of a more conjugated heteroarene. While this dehydrogenative coupling reaction was proven unsuccessful with most of the commercially available photosensitizers, 43% of the alkylated heteroarene **3** could be furnished with 5 mol% of 2,4-diphenylquinoline as the photocatalyst (Supplementary Table 3, entry 8).[1] This encouraging result could enlighten more visible light-promoted Cl· generation strategies for coupling reactions.

**Supplementary Table 3 | Investigation of photocatalysts under visible light irradiation.**

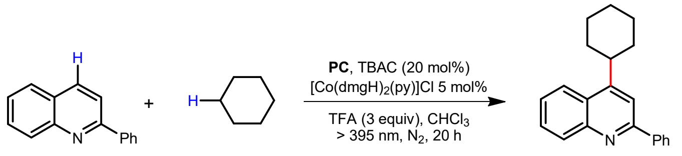


**1a** 0.2 mmol | **2a** 0.6 mL | **3**

| Entry | PC (mol%) | Temp (°C) | Yield (%)[a] | Entry | PC (mol%) | Temp (°C) | Yield (%)[a] |
|---|---|---|---|---|---|---|---|
| 1 | **PC1** (5) | 20-25 | 19 | 7 | **PC3** (5) | 20-25 | 10 |
| 2 | **PC1** (1) | 20-25 | 16 | 8 | **PC3** (5) | 55-60 | 43 |
| 3 | **PC1** (1) | 55-60 | 24 | 9 | **PC4** (5) | 20-25 | 0 |
| 4 | **PC2** (5) | 20-25 | 17 | 10 | **PC5** (5) | 20-25 | 0 |
| 5 | **PC2** (1) | 20-25 | 25 | 11 | **PC6** (5) | 20-25 | 0 |
| 6 | **PC2** (1) | 55-60 | 31 | | | | |

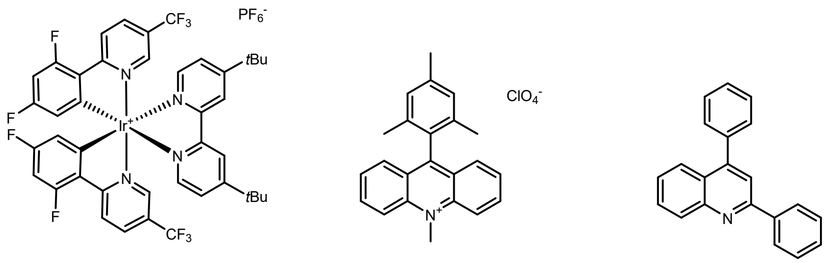

**PC1** *(Ir[dF(CF3)ppy]2(dtbpy))PF6* | **PC2** *Mes-Acr-Me+ ClO4-* | **PC3** *2,4-diphenylquinoline*

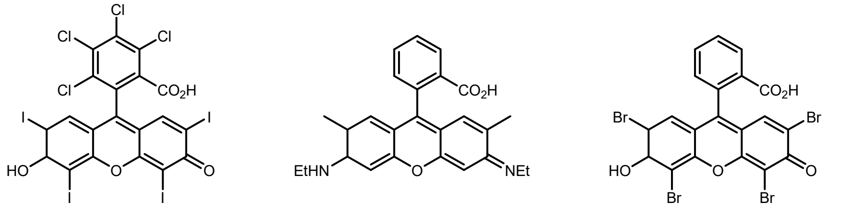

**PC4** *rose bengal* | **PC5** *Rhodamine 6G* | **PC6** *eosin Y*

[a] Yields were determined by $^{1}$H NMR using $CH_2Br_2$ as the internal standard; dmg, dimethylglyoxime; TFA, trifluoroacetic acid.

## 3-3. Mechanistic studies

## 3-3-1. Radical quenching experiment

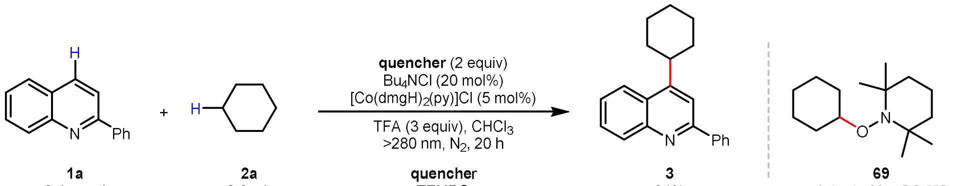


| **1a** | **2a** | **quencher** | **3** | **69** |
|---|---|---|---|---|
| 0.1 mmol | 0.3 mL | TEMPO | 31% | detected by GC-MS |
| | | BHT | 10% | |

quenchers:

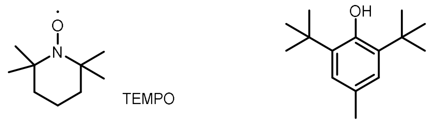

To a 10 mL pyrex microwave tube equipped with a Teflon-coated magnetic stirring bar was added heteroarene **1** (0.1 mmol), [Co(dmgH)$_2$(py)]Cl (2 mg, 0.005 mmol), and a radical quencher (TEMPO or BHT, 0.2 mmol). The tube was sealed with a rubber septum, evacuated and backfilled with argon three times before alkane **2a** (0.3 mL) was injected into the vial. The mixture was then sequentially added $Bu_4NCl$ (5.6 mg, 0.02 mmol), $CHCl_3$ (0.75 mL), and TFA (23 μL, 0.3 mmol) in the glovebox, and then sealed with an aluminium cap with a septum. The reaction vial was taken out from the glovebox and stirred at 20-25 °C under the irradiation of a 300 W Xe lamp with a 280 nm filter for 20 h. After the reaction was completed, the reaction was basified with sat $NaHCO_3$ (aq), extracted with EtOAc, filtered through a short pad of $MgSO_4$. The volatiles were removed under reduced pressure to obtain the crude product. The yield of **3** was determined by $^1$H NMR using $CH_2Br_2$ as the internal standard.

The formation of **3** was significantly suppressed by radical quenchers (31% with TEMPO, 10% with BHT), which suggested the reaction proceeds through a radical-involved pathway. When TEMPO was used as the radical quencher, the side product **69** was detected by GC-MS, which could be evidence for the cyclohexyl radical formation.

### 3-3-2. Alkyl radical trapping experiment

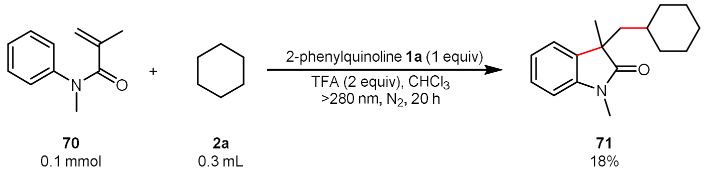


To a 10 mL pyrex microwave tube equipped with a Teflon-coated magnetic stirring bar was added alkyl radical acceptor **70** (17.5 mg, 0.1 mmol) and heteroarene **1a** (20.5 mg, 0.1 mmol). The tube was sealed with a rubber septum, evacuated and backfilled with argon three times before alkane **2a** (0.3 mL) was injected into the vial. The mixture was then sequentially added $Bu_4NCl$ (5.6 mg, 0.02 mmol), $CHCl_3$ (0.75 mL), and TFA (15.5 μL, 0.2 mmol) in the glovebox, and then sealed with an aluminium cap with a septum. The reaction vial was taken out from the glovebox and stirred at 20-25 °C under the irradiation of a 300 W Xe lamp with a 280 nm filter for 20 h. After the reaction was completed, the reaction was added $Et_2O$ and stirred for a minute, and then filtered through a short pad of silica gel with $Et_2O$. The volatiles were removed under reduced pressure to obtain the crude product. The isolated **71** was obtained in 18% yield by preparative thin-layer chromatography using Hex/EtOAc (5:1) as the eluent.

It is notable that Co(dmgH)$_2$(py)]Cl was not added in this experiment to eliminate the potential metal-catalyzed cycloalkylation pathway, although alkylated compound **71** was also observed in the presence of cobaloxime catalyst. The formation of **71** in this reaction suggested the reaction might proceed with the formation of alkyl radical.

### 3-3-3. Chlorine radical trapping experiment

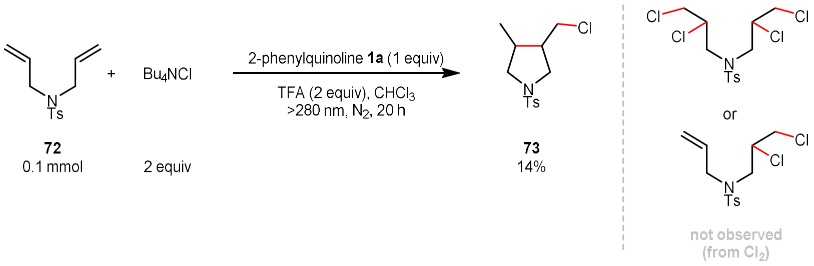

To a 10 mL pyrex microwave tube equipped with a Teflon-coated magnetic stirring bar was added chlorine radical acceptor **72** (25.1 mg, 0.1 mmol) and heteroarene **1a** (20.5 mg, 0.1 mmol). The tube was sealed with a rubber septum, evacuated and backfilled with argon three times. The mixture was then sequentially added $Bu_4NCl$ (55.6 mg, 0.2 mmol), $CHCl_3$ (0.75 mL), and TFA (15.5 μL, 0.2 mmol) in the glovebox, and then sealed with an aluminium cap with a septum. The reaction vial was taken out from the glovebox and stirred at 20-25 °C under the irradiation of a 300 W Xe lamp with a 280 nm filter for 20 h. After the reaction was completed, the reaction was added $Et_2O$ and stirred for a minute, and then filtered through a short pad of silica gel with $Et_2O$.The volatiles were removed under reduced pressure to obtain the crude product. The isolated **73** was obtained in 14% yield by preparative thin-layer chromatography using Hex/EtOAc (5:1) as the eluent.

It is notable that Co(dmgH)$_2$(py)]Cl was not added in this experiment to eliminate the potential metal-catalyzed chlorination pathway, although chlorinated compound **73** was also observed in the presence of cobaloxime catalyst. According to the radical trapping results reported by the groups of Lin and Xu,[2-3] the formation of **73** in this reaction suggested the reaction might proceed with the formation of chlorine radical without the formation of chlorine, as no dichlorination of alkene was detected by GC-MS and $^1$H NMR.

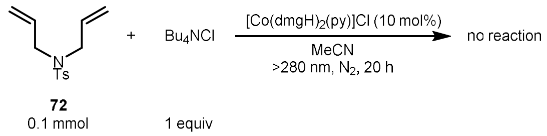


To a 10 mL pyrex microwave tube equipped with a Teflon-coated magnetic stirring bar was added chlorine radical acceptor **72** (25.1 mg, 0.1 mmol) and Co(dmgH)$_2$(py)]Cl (4 mg, 0.01 mmol). The tube was sealed with a rubber septum, evacuated and backfilled with argon three times. The mixture was then sequentially added $Bu_4NCl$ (27.8 mg, 0.1 mmol) and MeCN (0.75 mL) in the glovebox, and then sealed with an aluminium cap with a septum. The reaction vial was taken out from the glovebox and stirred at 20-25 °C under the irradiation of a 300 W Xe lamp with a 280 nm filter for 20 h. After the reaction was completed, the reaction was filtered through a short pad of silica gel. The volatiles were removed under reduced pressure to obtain the crude sample. No reaction was observed from GC-MS and $^1$H NMR, indicating the ligand-to-metal charge transfer (LMCT)-induced chlorine radical generation on the cobalt catalyst might not be involved in this reaction.

To further identify the chlorine radical source in this reaction, some more control experiments were conducted. As shown in Table 4, both **1a**/$Bu_4NCl$ (entry 3) and $CHCl_3$ (entry 6) are evidenced to be effective chlorine radical sources for the generation of **73**. We could not detect **73** when **1a**/[Co(dmgH)$_2$(py)]Cl was used as the chlorine radical source (entry 7), which could be rationalized by reasons such as the low chloride loading (20-50 mol% of the cobalxime) or the absorption interference from the cobaloxime species.[4]

**Supplementary Table 4 | Investigation of the chlorine radical sources**

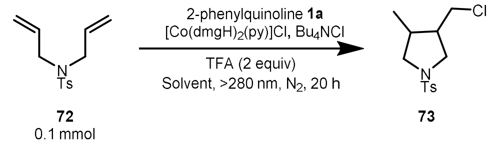


| Entry | 1 equiv **1a** | 2 equiv $Bu_4NCl$ | $[Co(dmgH)_2(py)]Cl$ | Solvent | **73** (%)[a] |
|---|---|---|---|---|---|
| 1 | yes | yes | yes (5 mol%) | $CHCl_3$ | 8 |
| 2 | yes | yes | no | $CHCl_3$ | 14 |
| 3 | yes | yes | no | MeCN | 6 |
| 4 | no | yes | no | MeCN | n.d. |
| 5 | yes | no | no | $CHCl_3$ | <5 |
| 6 | no | no | no | $CHCl_3$ | <5 |
| 7 | yes | no | yes (20, 50, or 100 mol%) | MeCN | n.d. |
| 8 | no | no | yes (20, 50, or 100 mol%) | MeCN | n.d. |

[a] Yields were determined by $^1H$ NMR using $CH_2Br_2$ as the internal standard. [Co], $[Co(dmgH)_2(py)]Cl$; TFA, trifluoroacetic acid; n.d., not detected.

## 3-3-4. Possible intermediate investigation

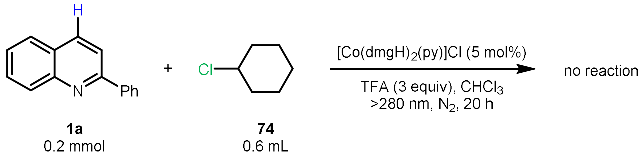


To a 10 mL pyrex microwave tube equipped with a Teflon-coated magnetic stirring bar was added heteroarene **1a** (20.5 mg, 0.1 mmol) and $[Co(dmgH)_2(py)]Cl$ (2 mg, 0.005 mmol). The tube was sealed with a rubber septum, evacuated and backfilled with argon three times before chlorinated alkane **74** (0.3 mL) was injected into the vial. The mixture was then sequentially added $CHCl_3$ (0.75 mL) and TFA (15.5 μL, 0.2 mmol) in the glovebox, and then sealed with an aluminium cap with a septum. The reaction vial was taken out from the glovebox and stirred at 20-25 °C under the irradiation of a 300 W Xe lamp with a 280 nm filter for 20 h. After the reaction was completed, the reaction was basified with sat $NaHCO_3$ (aq), extracted with EtOAc, filtered through a short pad of $MgSO_4$. The volatiles were removed under reduced pressure to obtain the crude product. No reaction was observed from GC-MS and $^1H$ NMR, which suggested the reaction should not go through the formation of chlorinated alkane followed by homolysis of the C($sp^3$)–Cl bond to generate the alkyl radical for the heteroarene addition.

## 3-3-5. Light on/off experiment

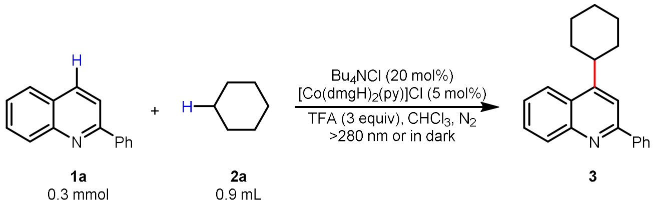


To a 10 mL pyrex microwave tube equipped with a Teflon-coated magnetic stirring bar was added heteroarene **1a** (61.5 mg, 0.3 mmol) and $[Co(dmgH)_2(py)]Cl$ (6 mg, 0.015 mmol). The tube was sealed with a rubber septum, evacuated and backfilled with argon three times before alkane **2a** (0.9 mL) was injected into the vial. The mixture was then sequentially added $Bu_4NCl$ (16.7 mg, 0.03 mmol), $CHCl_3$ (2.3 mL), and

TFA (69 μL, 0.9 mmol) in the glovebox, and then sealed with an aluminium cap with a septum. The reaction vial was taken out from the glovebox and stirred at 20-25 °C, with or without the irradiation of a 300 W Xe lamp with a 280 nm filter, as the time period indicated in Figure 4. At the end of each period, a small portion (100 to 150 μL) of the reacting solution was taken by a syringe, basified with sat $NaHCO_3$ (aq), extracted with EtOAc, and the volatiles were removed under reduced pressure to obtain the crude sample, which was taken for $^1$H NMR analysis.

| Time (h) | Light | Conevrsion of **1a** to **3** (%) |
| --- | --- | --- |
| 2 | on | 23 |
| 3 | off | 23 |
| 6 | on | 37 |
| 7 | off | 37 |
| 11 | on | 51 |
| 12 | off | 51 |
| 17 | on | 70 |

⟹

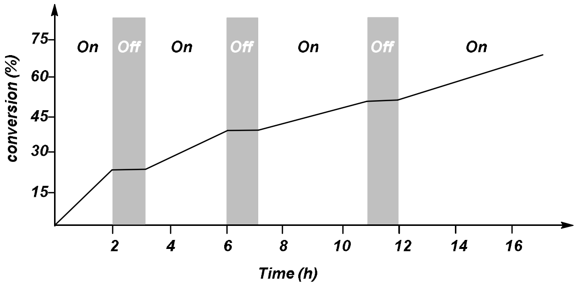


**Supplementary Figure 4 | Time frame for the conversion of 1a to 3**

Because no side reaction or loss of the starting **1a** was observed for this model reaction in our previous investigations, the conversion yield from **1a** to **3** was considered as the NMR yield of **3** in this experiment. Although it could not exclude the potential radical chain pathway because this process could be on the second or sub-second timescale,[5-6] the results clearly show that continuous light irradiation is essential for the product formation.

## 3-3-6. Quantum yield measurement

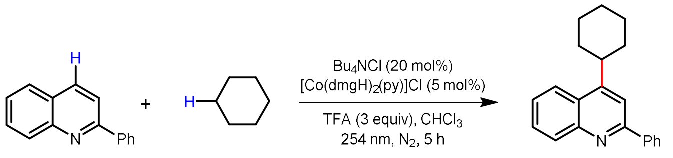


**1a** 0.1 mmol | **2a** 0.3 mL | **3** 6 % 7% conversion

Since the Xe lamp used in the standard procedure has a wide wavelength spectrum, here we used single wavelength 254 nm lamps to conduct the experiment. To a 10 mL quartz tube equipped with a Teflon-coated magnetic stirring bar was added heteroarene **1a** (20.5 mg, 0.1 mmol) and $[Co(dmgH)_2(py)]Cl$ (2 mg, 0.005 mmol). The tube was sealed with a rubber septum, evacuated and backfilled with argon three times before alkane **2a** (0.3 mL) was injected into the vial. The mixture was then sequentially added $Bu_4NCl$ (5.6 mg, 0.01 mmol), $CHCl_3$ (0.75 mL), and TFA (23 μL, 0.3 mmol) in the glovebox, and then sealed with a screw cap. The reaction tube was taken out from the glovebox and stirred in the UV box at room temperature under irradiation of 6×8 W 254 nm UVC lamps. After 5 h, the reaction was basified with sat $NaHCO_3$ (aq), extracted with EtOAc. The volatiles were removed under reduced pressure to obtain the crude sample, which was taken for $^1$H NMR analysis. 6% of product **3** was formed without side reaction.

The light power of the 6×8 W 254 nm UVC lamps was determined by an optical power meter to be 7.6 mW.

$$\text{photon flux} = \frac{P}{N_A \cdot hc/\lambda} = \frac{7.6 \cdot 10^{-3}}{6.02 \cdot 10^{23} \cdot 6.63 \cdot 10^{-3} \ \cdot 3 \cdot 10^{8}/(254 \cdot 10^{-9})} = 1.6 \cdot 10^{-8} \text{ einsteins} \cdot s^{-1}$$

Assuming all the incident light was absorbed by **1a** under this reaction conditions (f > 0.999), the production of $6.4 \cdot 10^{-6}$ mol **3a** (6.4%) in 5 h ($1.8 \cdot 10^{4}$ s) led to the quantum yield (Φ) of 2.1%.

$$\Phi = \frac{\text{mol product}}{\text{photon flux} \cdot t \cdot f} = \frac{6 \cdot 10^{-6}}{1.6 \cdot 10^{-8} \cdot 1.8 \cdot 10^{4} \cdot 1} = 2.1\ \%$$

Since the Φ is far smaller than 1, although we still could not exclude the possibility of the radical chain process of this reaction, the quantum yield calculation does not support the radical chain process.

### 3-3-7. Kinetic isotope effect (KIE) experiment

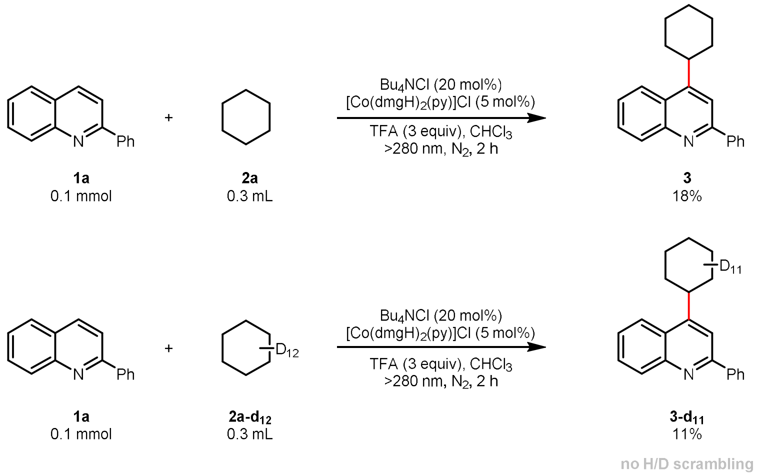


**Parallel reactions**. Two 10 mL pyrex microwave tubes equipped with Teflon-coated magnetic stirring bars were added heteroarene **1a** (20.5 mg, 0.1 mmol) and $[Co(dmgH)_2(py)]Cl$ (2 mg, 0.005 mmol). The tubes were sealed with rubber septa, evacuated and backfilled with argon three times before alkane **2a** (0.3 mL) and deuterated alkane **2a-d$_{12}$** (0.3 mL) were injected into vials, separately. The mixtures were then sequentially added $Bu_4NCl$ (5.6 mg, 0.02 mmol), $CHCl_3$ (0.75 mL), and TFA (23 μL, 03 mmol) in the glovebox, and sealed with aluminium caps with septa. The reaction vials were taken out from the glovebox and stirred at 20-25 °C under the irradiation of a 300 W Xe lamp with a 280 nm filter. After 2 h, they were was basified with sat $NaHCO_3$ (aq), extracted with EtOAc, filtered through a short pad of $MgSO_4$. The volatiles were removed under reduced pressure to obtain the crude product. The NMR yields were determined to be 18% for **3** and 11% for **3-d$_{11}$** using $CH_2Br_2$ as the internal standard. The isolated product **3-d$_{11}$** was obtained by preparative thin-layer chromatography using Hex/EtOAc (10:1) as the eluent. No H/D scrambling was observed on **3-d$_{11}$**. $k_H/k_D = 1.64$.

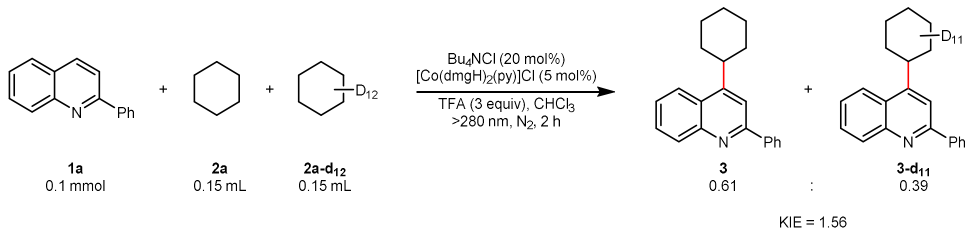


**Intermolecular competition**. To a 10 mL pyrex microwave tube equipped with a Teflon-coated magnetic stirring bar was added heteroarene **1a** (20.5 mg, 0.1 mmol) and [Co(dmgH)$_2$(py)]Cl (2 mg, 0.005 mmol). The tube was sealed with a rubber septum, evacuated and backfilled with argon three times before alkane **2a** (0.15 mL) and alkane **2a-d$_{12}$** (0.15 mL) were injected into the vial. The mixture was then sequentially added $Bu_4NCl$ (5.6 mg, 0.02 mmol), $CHCl_3$ (0.75 mL), and TFA (23 μL, 0.3 mmol) in the glovebox, and sealed with an aluminium cap with a septum. The reaction vial was taken out from the glovebox and stirred at 20-25 °C under the irradiation of a 300 W Xe lamp with a 280 nm filter. After 2 h, the reaction was basified with sat $NaHCO_3$ (aq), extracted with EtOAc, filtered through a short pad of $MgSO_4$. The volatiles were removed under reduced pressure to obtain the crude product. The NMR yield of the mixture was determined to be 16% using $CH_2Br_2$ as the internal standard, with 0.61:0.39 ratio of **3**:**3-d$_{11}$**. $k_H/k_D$ = 1.56.

Neither of these experiments showed a prominent isotope effect, suggesting that the alkyl C–H cleavage might not be the rate-determine step.[7]

### 3-3-8. Detection of the $H_2$ evolution

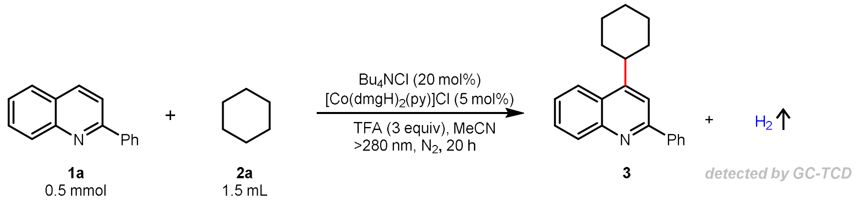


To a 10 mL pyrex microwave tube equipped with a Teflon-coated magnetic stirring bar was added heteroarene **1a** (102.5 mg, 0.5 mmol) and [Co(dmgH)$_2$(py)]Cl (10 mg, 0.025 mmol). The tube was sealed with a rubber septum, evacuated and backfilled with argon three times before alkane **2a** (1.5 mL) was injected into the vial. The mixture was then sequentially added $Bu_4NCl$ (27.8 mg, 0.1 mmol), MeCN (3.75 mL), and TFA (115 μL, 1.5 mmol) in the glovebox, and then sealed with an aluminium cap with a septum. The reaction vial was taken out from the glovebox and stirred at 20-25 °C under the irradiation of a 300 W Xe lamp with a 280 nm filter for 20 h. After the reaction was completed, the upper 1 mL gas content of the tube was taken by a gas-tight syringe and analyzed by GC-TCD. The detection of the $H_2$ signal indicated the formation of hydrogen during the reaction.

### 3-3-9. UV-vis and fluorescence quenching

**Preparation.** Three formulated solutions were prepared with degassed $CHCl_3$ in 10 mL volumetric flasks. For flask A, 4-methylquinoline (**1o**, 4-MeQN, 0.5 mmol, 66 μL) was added; for flask B, 4-methylquinoline (**1o**, 4-MeQN, 0.5 mmol, 66 μL) and TFA (57 μL, 0.75 mmol) were added; for the flask C, $Bu_4NCl$ (0.5 mmol, 139 mg) was added. All these flasks were diluted to 10 mL to set the concentration to be 0.05 M.

**UV-vis spectrometric experiments.** A quartz cuvette (1 cm ×1 cm ×3.5 cm) was filled with 2 mL of the abovementioned 0.05 mM solutions from flasks A and B to perform the UV-vis experiment (800 nm to 200 nm). The resulting spectra are shown in Figure 5.

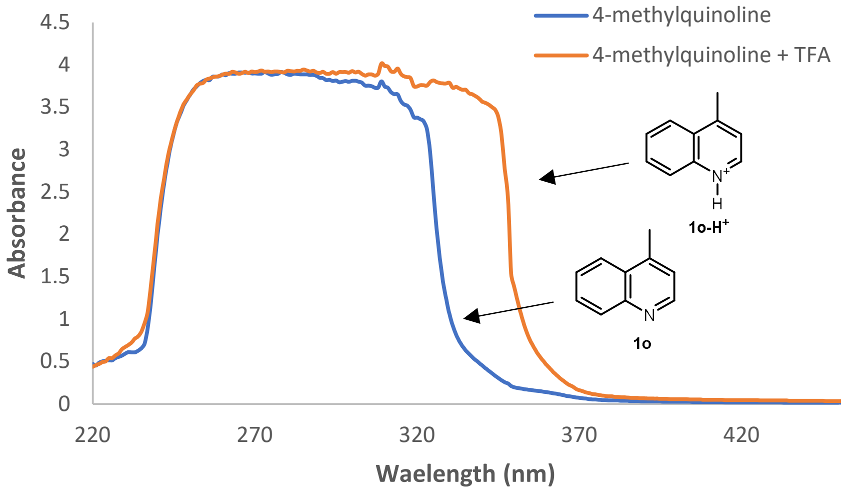


**Supplementary Figure 5 | UV-vis spectra of 0.05 M heteroarene 1o in $CHCl_3$, with or without TFA.**

**Fluorescence quenching experiments of 1o.** A quartz cuvette (1 cm ×1 cm ×3.5 cm) was added 18 μL of the formulated solution from flask A and was diluted to 3 mL as a 0.3 μM **1o** solution, which was then irradiated at 300 nm. Duplicate experiments were performed with the addition of 0, 60, 120, 180, 240, 300 μL 0.05 mM $Bu_4NCl$ solution from flask C before diluted to 3 mL. The resulting fluorescence emission spectra are shown in Figure 6. No significant fluorescence quenching between excited **1o** and $Bu_4NCl$ was observed.

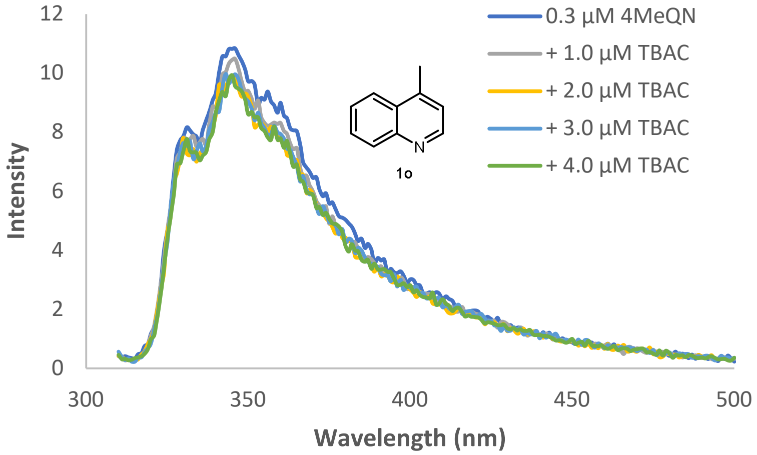


**Supplementary Figure 6 | Emission intensity of 0.3 μM heteroarene 1o in $CHCl_3$, with varied amount of $Bu_4NCl$.**

**Fluorescence quenching experiments of 1o-H⁺.** A quartz cuvette (1 cm ×1 cm ×3.5 cm) was added 6 µL of the formulated solution from flask A and was diluted to 3 mL as a 0.1 µM **1o** solution, which was then irradiated at 300 nm. Duplicate experiments were performed with the addition of 0, 30, 60, 90, 120, 240 µL 0.05 mM $Bu_4NCl$ solution from flask C before diluted to 3 mL. The resulting fluorescence emission spectra are shown in Figure 7.

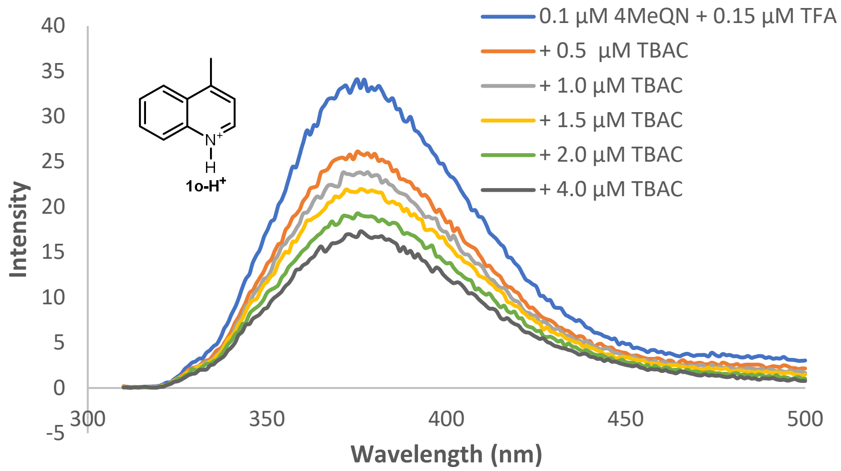


**Supplementary Figure 7 | Emission intensity of 0.1 µM heteroarene 1o-H⁺ in $CHCl_3$, with varied amount of $Bu_4NCl$.**

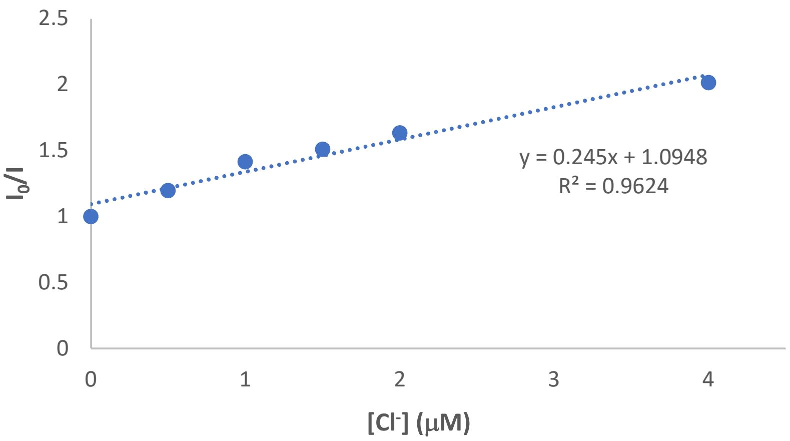


**Supplementary Figure 8| Stern-Volmer plot of heteroarene 1o-H⁺ and $Bu_4NCl$.**

While no significant fluorescence quenching of heteroarene **1o** was observed in Figure 6, prominent fluorescence quenching between excited **1o-H⁺** and $Bu_4NCl$ was observed (Figures 7 and 8), indicating that the protonated heteroarene **1o-H⁺** should be the active species for $Cl^-$ oxidation.

### 3-3-10. C(sp$^3$)–H selectivity test

To showcase the importance of controlling Cl· concentration, C(sp$^3$)–H selectivity experiments were conducted with 2-phenylquinoline (**1a**) and N-methyl-2-pyrrolidone (**2aa**) to observe the ratio between 2° and 3° C(sp$^3$)–H adducts (**31a** and **31b**), by changing the amount of $Cl^-$ and solvent (Table 5).

**Supplementary Table 5 | C(sp$^3$)–H selectivity test of the generated chlorine radical.**

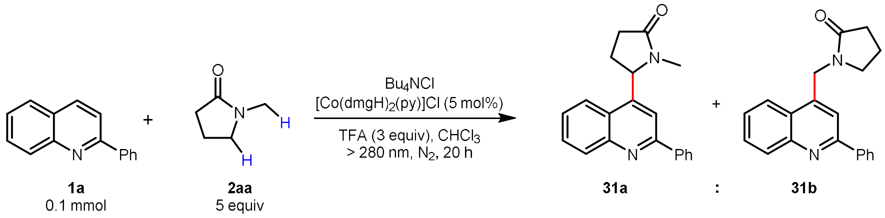


| Entry | $Bu_4NCl$ (mol%) | [M] of **1a** (M) | **31a** : **31b** |
|---|---|---|---|
| 1 | 20 | 0.2 | 1 : 0.26 |
| 2 | 20 | 0.1 | 1 : 0.12 |
| 3 | 20 | 0.05 | 1 : 0.13 |
| 4 | 200 | 0.2 | 1 : 0.31 |
| 5 | 200 | 0.1 | 1 : 0.26 |
| 6 | 200 | 0.05 | 1 : 0.23 |

[a] Yields were determined by $^1$H NMR using $CH_2Br_2$ as the internal standard. TFA, trifluoroacetic acid; n.d.

As shown in Table 5, the 2° C(sp$^3$)–H adduct **31a** became more major under lower Cl· concentration, showing the Cl· concentration could be critical in effecting the regioselectivity outcome.

## 4. Characterization data for compounds

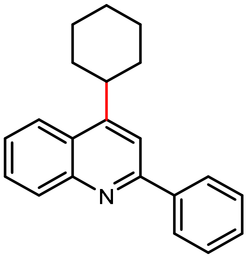


**4-cyclohexyl-2-phenylquinoline (3)**. Following the general procedure A, the product was isolated by preparative TLC with Hex/EtOAc (10:1) as the eluent to give a white solid (42.5 mg, 74%). **$^1$H NMR** (500 MHz, $CDCl_3$) δ 8.21 (d, $J$ = 8.4 Hz, 1H), 8.18 – 8. 15 (m, 2H), 8.12 (d, $J$ = 8.4 Hz, 1H), 7.78 (s, 1H), 7.75 – 7.70 (m, 1H), 7.58 – 7.52 (m, 3H), 7.50 – 7.45 (m, 1H), 3.46 – 3.35 (m, 1H), 2.15 – 2.07 (m, 2H), 2.02 – 1.95 (m, 2H), 1.94 – 1.87 (m, 1H), 1.73 – 1.55 (m, 4H), 1.47 – 1.35 (m, 1H). **$^{13}$C NMR** (125 MHz, $CDCl_3$) δ 157.4, 153.9, 148.6, 140.3, 130.7, 129.1, 129.0, 128.8, 127.6, 125.9, 122.9, 115.5, 39.1, 33.7, 27.0, 26.4. **GC-MS** (EI, m/z) for $C_{21}H_{21}N$ Calcd: 287.2, found: 287.1. Spectra data are consistent with the reported literature.[3]

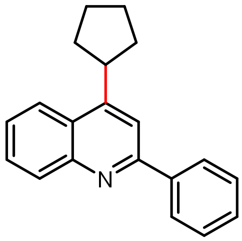

**4-cyclopentyl-2-phenylquinoline (4)**. Following the general procedure A, the product was isolated by preparative TLC with Hex/EtOAc (10:1) as the eluent to give a colorless oil (43.2 mg, 79%). **$^1$H NMR** (500 MHz, $CDCl_3$) δ 8.22 (d, *J* = 7.1 Hz, 1H), 8.19 – 8.13 (m, 3H), 7.81 (s, 1H), 7.76 – 7.70 (m, 1H), 7.58 – 7.52 (m, 3H), 7.51 – 7.46 (m, 1H), 3.90 – 3.80 (m, 1H), 2.35 – 2.23 (m, 2H), 2.01 – 1.80 (m, 6H). **$^{13}$C NMR** (125 MHz, $CDCl_3$) δ 157.3, 152.8, 148.6, 140.3, 130.6, 129.1, 129.0, 128.8, 127.6, 126.8, 125.8, 123.6, 115.2, 40.8, 33.4, 25.5. **GC-MS** (EI, m/z) for $C_{20}H_{19}N$ Calcd: 273.2, found: 273.1. Spectra data are consistent with the reported literature.[3]

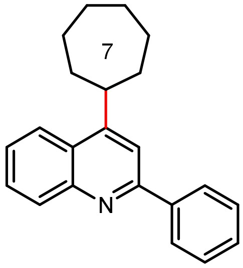

**4-cycloheptyl-2-phenylquinoline (5)**. Following the general procedure A, the product was isolated by preparative TLC with Hex/EtOAc (10:1) as the eluent to give a colorless oil (44.0 mg, 73%). **$^1$H NMR** (500 MHz, $CDCl_3$) δ 8.22 (d, *J* = 8.4 Hz, 1H), 8.19 – 8.15 (m, 2H), 8.11 (dd, *J* = 8.6, 1.4 Hz, 1H), 7.79 (s, 1H), 7.75 – 7.70 (m, 1H), 7.59 – 7.52 (m, 3H), 7.51 – 7.46 (m, 1H), 3.61 – 3.52 (m, 1H), 2.19 – 2.10 (m, 2H), 2.00 – 1.79 (m, 6H), 1.79 – 1.67 (m, 4H). **$^{13}$C NMR** (125 MHz, $CDCl_3$) δ 157.3, 155.9, 148.6, 140.3, 130.7, 129.1, 129.0, 128.8, 127.6, 125.9, 125.6, 123.0, 115.8, 40.8, 35.9, 27.9, 27.6. **GC-MS** (EI, m/z) for $C_{22}H_{23}N$ Calcd: 301.2, found: 301.1. Spectra data are consistent with the reported literature.[3]

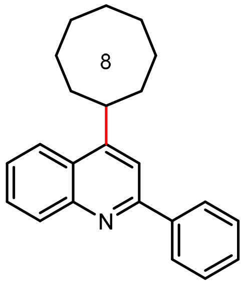

**4-cyclooctyl-2-phenylquinoline (6)**. Following the general procedure A, the product was isolated by preparative TLC with Hex/EtOAc (10:1) as the eluent to give a colorless oil (32.8 mg, 52%). **$^1$H NMR** (500 MHz, $CDCl_3$) δ 8.22 (d, *J* = 8.8 Hz, 1H), 8.20 – 8.14 (m, 2H), 8.12 (d, *J* = 8.7 Hz, 1H), 7.78 (s, 1H), 7.75

– 7.68 (m, 1H), 7.60 – 7.51 (m, 3H), 7.51 – 7.45 (m, 1H), 3.74 – 3.61 (m, 1H), 2.10 – 1.96 (m, 4H), 1.95 – 1.85 (m, 2H), 1.85 – 1.73 (m, 8H). **$^{13}$C NMR** (125 MHz, $CDCl_3$) δ 157.3, 156.4, 148.7, 140.3, 130.8, 129.1, 129.0, 128.8, 127.6, 125.9, 125.6, 123.1, 116.3, 33.8, 26.9, 26.6, 26.2. **GC-MS** (EI, m/z) for $C_{23}H_{25}N$ Calcd: 315.2, found: 315.2. Spectra data are consistent with the reported literature.[3]

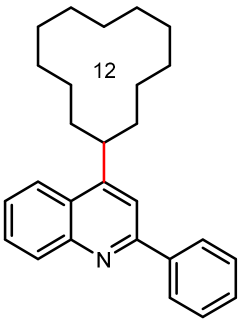


**4-cyclododecyl-2-phenylquinoline (7)**. Following the general procedure B, the reaction was run at 55-60 °C for 36 h,the product was isolated by preparative TLC with Hex/EtOAc (10:1) as the eluent to give a white solid (52.8 mg, 71%). **$^1$H NMR** (500 MHz, $CDCl_3$) δ 8.24 (d, $J$ = 8.4 Hz, 1H), 8.22 – 8.12 (m, 3H), 7.79 (s, 1H), 7.77 – 7.70 (m, 1H), 7.61 – 7.54 (m, 3H), 7.53 – 7.47 (m, 1H), 3.80 – 3.66 (m, 1H), 2.06 – 1.95 (m, 2H), 1.83 – 1.73 (m, 2H), 1.65 – 1.38 (m, 16H), 1.38 – 1.26 (m, 2H). **$^{13}$C NMR** (125 MHz, $CDCl_3$) δ 157.1, 153.9, 148.7, 140.3, 130.8, 129.1, 129.0, 128.8, 127.7, 126.6, 126.0, 122.9, 116.7, 34.6, 30.2, 24.1, 23.6, 23.5, 22.6. **GC-MS** (EI, m/z) for $C_{27}H_{33}N$ Calcd: 371.3.2, found: 371.3. Spectra data are consistent with the reported literature.[3]

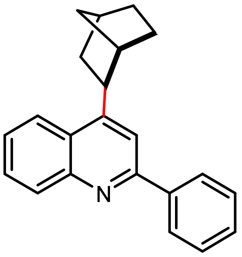


**4-bicyclo[2.2.1]heptan-2-yl)-2-phenylquinoline (8)**. Following the general procedure B, the reaction was run for 36 h, and the product was isolated by preparative TLC with Hex/EtOAc (10:1) as the eluent to give a colorless oil (41.9 mg, 70%). **$^1$H NMR** (500 MHz, $CDCl_3$) δ 8.21 (d, $J$ = 8.5 Hz, 1H), 8.18 – 8.14 (m, 2H), 8.09 (d, $J$ = 8.4 Hz, 1H), 7.77 (s, 1H), 7.75 – 7.69 (m, 1H), 7.59 – 7.52 (m, 3H), 7.52 – 7.46 (m, 1H), 3.48 – 3.41 (m, 1H), 2.70 (d, $J$ = 4.1 Hz, 1H), 2.52 – 2.43 (m, 1H), 2.12 – 2.03 (m, 1H), 1.85 – 1.66 (m, 4H), 1.63 – 1.54 (m, 1H), 1.51 – 1.44 (m, 1H), 1.43 – 1.37 (m, 1H). **$^{13}$C NMR** (125 MHz, $CDCl_3$) δ 157.2, 153.3, 148.7, 140.4, 130.5, 129.1, 129.0, 128.8, 127.7, 126.5, 125.8, 123.9, 114.9, 43.0, 41.3, 39.3, 37.0, 36.7, 30.3, 29.1. **GC-MS** (EI, m/z) for $C_{22}H_{21}N$ Calcd: 299.2, found: 299.1. Spectra data are consistent with the reported literature.[3]

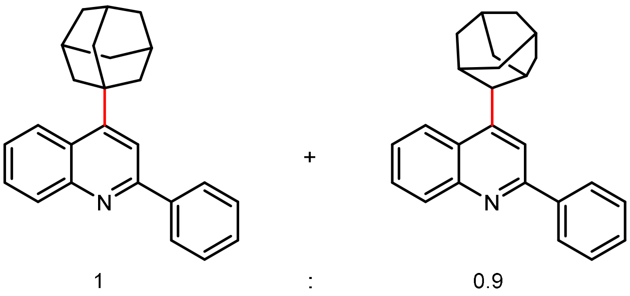

**(4-adamantan-1-yl)-2-phenylquinoline (9a) and 4-adamantan-2-yl)-2-phenylquinoline (9b), 1:0.9 congeners**. Following the general procedure B, the reaction was run at 55-60 °C for 36 h, and the product was isolated by preparative TLC with Hex/EtOAc (10:1) as the eluent to give a colorless oil (27.8 mg, 41%). **$^1$H NMR** (500 MHz, $CDCl_3$) δ 8.62 (d, *J* = 8.7 Hz, 1H), 8.26 – 8.12 (m, 3H+2.7H), 8.03 (s, 0.9H), 7.94 (d, *J* = 8.4 Hz, 0.9H), 7.80 (s, 1H), 7.71 – 7.63 (m, 1H+0.9H), 7.58 – 7.43 (m, 3H+2.7H), 3.79 (s, 0.9H), 2.50 (s, 1.8H), 2.40 – 2.34 (m, 6H), 2.28 – 1.69 (m, 9H+10.8H). **$^{13}$C NMR** (125 MHz, $CDCl_3$) δ $^{13}$C NMR (125 MHz, $CDCl_3$) δ 157.2, 156.9, 156.0, 151.8, 149.8, 148.8, 140.4, 131.7, 130.9, 129.1, 129.1, 128.8, 128.8, 128.8, 128.2, 127.6, 127.6, 126.5, 126.1, 125.8, 125.7, 124.6, 123.8, 117.4, 116.4, 45.5, 42.3, 40.1, 38.9, 37.8, 37.0, 33.0, 32.7, 29.1, 28.3, 27.7. **HRMS** (M+H$^+$) for $C_{25}H_{26}N$ Calcd: 340.2060, found: 340.2054. The compound was not reported.

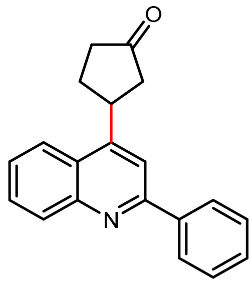

**3-(2-phenylquinolin-4-yl)cyclopentan-1-one (10)**. Following the general procedure A, the reaction was run at 55-60 °C for 36 h, and the product was isolated by preparative TLC with Hex/EtOAc (5:2) as the eluent to give a colorless oil (27.0 mg, 47%). **$^1$H NMR** (500 MHz, $CDCl_3$) δ 8.25 (d, *J* = 8.7 Hz, 1H), 8.18 – 8.13 (m, 2H), 8.10 (d, *J* = 7.8 Hz, 1H), 7.81 – 7.74 (m, 2H), 7.64 – 7.59 (m, 1H), 7.59 – 7.53 (m, 2H), 7.53 – 7.47 (m, 1H), 4.33 – 4.23 (m, 1H), 2.94 – 2.85 (m, 1H), 2.70 – 2.53 (m, 3H), 2.53 – 2.43 (m, 1H), 2.34 – 2.23 (m, 1H). **$^{13}$C NMR** (125 MHz, $CDCl_3$) δ 217.1, 157.3, 149.1, 148.7, 139.7, 130.9, 129.5, 129.5, 128.9, 127.6, 126.5, 126.0, 122.8, 114.9, 45.0, 38.1, 37.4, 29.8. **GC-MS** (EI, m/z) for $C_{20}H_{17}NO$ Calcd: 287.1, found: 287.0. Spectra data are consistent with the reported literature.[8]

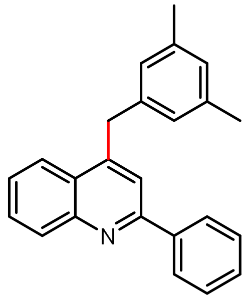

**4-(3,5-dimethylbenzyl)-2-phenylquinoline (11)**. Following the general procedure A, the product was isolated by preparative TLC with Hex/EtOAc (10:1) as the eluent to give a colorless oil (39.5 mg, 61%). **$^1$H NMR** (500 MHz, $CDCl_3$) δ 8.23 (d, *J* = 7.2 Hz, 1H), 8.19 – 8.14 (m, 2H), 8.06 (d, *J* = 7.8 Hz, 1H), 7.76 – 7.69 (m, 2H), 7.57 – 7.46 (m, 4H), 6.91 (s, 1H), 6.88 (s, 2H), 4.45 (s, 2H), 2.30 (s, 6H). **$^{13}$C NMR** (125 MHz, $CDCl_3$) δ 157.2, 148.6, 147.3, 139.8, 138.7, 138.3, 130.4, 129.3, 129.3, 128.8, 128.3, 127.6, 126.7, 126.7, 126.3, 123.9, 120.0, 38.5, 21.3. **GC-MS** (EI, m/z) for $C_{24}H_{21}N$ Calcd: 323.2, found: 323.1. Spectra data are consistent with the reported literature.[3]

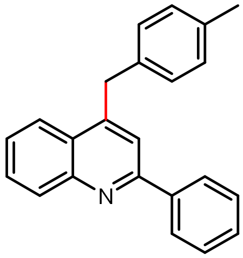


**4-(4-methylbenzyl)-2-phenylquinoline (12)**. Following the general procedure A, the product was isolated by preparative TLC with Hex/EtOAc (10:1) as the eluent to give a colorless oil (26.0 mg, 42%). **$^1$H NMR** (500 MHz, $CDCl_3$) δ 8.21 (d, *J* = 8.2 Hz, 1H), 8.15 – 8.11 (m, 2H), 8.05 (d, *J* = 9.0 Hz, 1H), 7.75 – 7.70 (m, 1H), 7.68 (s, 1H), 7.56 – 7.50 (m, 3H), 7.49 – 7.44 (m, 1H), 7.28 (s, 1H), 7.19 – 7.11 (m, 4H), 4.49 (s, 2H), 2.35 (s, 3H). **$^{13}$C NMR** (125 MHz, $CDCl_3$) δ 157.2, 148.6, 147.3, 139.8, 136.2, 135.7, 130.5, 129.4, 129.3, 129.2, 128.8, 128.7, 127.6, 126.6, 126.3, 123.8, 119.9, 38.2, 21.1. **GC-MS** (EI, m/z) for $C_{23}H_{19}N$ Calcd: 309.2, found: 309.1. Spectra data are consistent with the reported literature.[3]

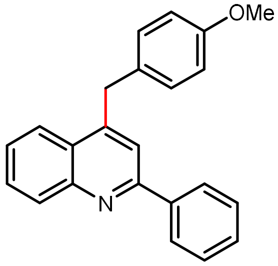


**4-(4-methoxybenzyl)-2-phenylquinoline (13).** Following the general procedure A, the product was isolated by preparative TLC with Hex/EtOAc (10:1) as the eluent to give a yellow solid (25.4 mg, 39%). **$^1$H NMR** (500 MHz, $CDCl_3$) δ 8.22 (d, *J* = 7.9 Hz, 1H), 8.16 – 8.10 (m, 2H), 8.06 (dd, *J* = 8.4, 1.4 Hz, 1H), 7.77 – 7.70 (m, 1H), 7.65 (s, 1H), 7.56 – 7.50 (m, 3H), 7.50 – 7.44 (m, 1H), 7.19 (d, *J* = 8.6 Hz, 2H), 6.88 (d, *J* = 8.7 Hz, 2H), 4.47 (s, 2H), 3.82 (s, 3H). **$^{13}$C NMR** (125 MHz, $CDCl_3$) δ 158.3, 157.2, 148.6, 147.5, 139.8, 130.7, 130.5, 129.9, 129.3, 129.3, 128.8, 127.6, 126.6, 126.3, 123.7, 119.7, 114.2, 55.3, 37.7. **HRMS** ($M+H^+$) for $C_{23}H_{20}NO$ Calcd: 326.1539, found: 326.1543. The compound was not reported.

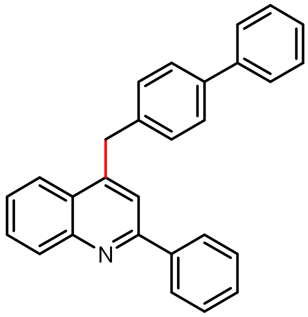


**4-([1,1'-biphenyl]-4-ylmethyl)-2-phenylquinoline (14).** Following the general procedure B, the product was isolated by preparative TLC with Hex/EtOAc (10:1) as the eluent to give a yellow solid (26.0 mg, 35%). **$^1$H NMR** (500 MHz, $CDCl_3$) δ 8.25 (d, *J* = 8.0 Hz, 1H), 8.18 – 8.14 (m, 2H), 8.09 (d, *J* = 7.7, 1H), 7.78 – 7.72 (m, 2H), 7.64 – 7.51 (m, 7H), 7.51 – 7.42 (m, 3H), 7.40 – 7.31 (m, 3H), 4.57 (s, 2H). **$^{13}$C NMR** (125 MHz, $CDCl_3$) δ 157.2, 148.6, 146.9, 140.7, 139.8, 139.6, 137.9, 130.5, 129.4, 129.3, 129.3, 128.8, 128.8, 127.6, 127.5, 127.3, 127.0,

126.6, 126.4, 123.8, 120.0, 38.2. **HRMS** (M+H$^+$) for $C_{28}H_{22}N$ Calcd: 372.1747, found: 372.1745. The compound was not reported.

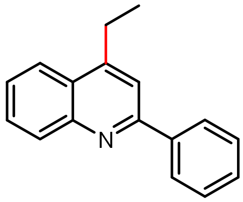

**4-ethyl-2-phenylquinoline (15)**. Following the general procedure A, the product was isolated by preparative TLC with Hex/EtOAc (10:1) as the eluent to give a colorless oil (35.9 mg, 77%). **$^1$H NMR** (500 MHz, $CDCl_3$) δ 8.24 – 8.16 (m, 3H), 8.07 (dd, *J* = 8.3, 1.3 Hz, 1H), 7.78 – 7.70 (m, 2H), 7.59 – 7.52 (m, 3H), 7.52 – 7.46 (m, 1H), 3.21 (q, *J* = 0.8 Hz, 2H), 1.48 (t, *J* = 7.6 Hz, 3H). **$^{13}$C NMR** (125 MHz, $CDCl_3$) δ 157.3, 150.5, 148.4, 140.1, 130.5, 129.2, 129.2, 128.8, 127.6, 126.4, 126.0, 123.3, 117.8, 25.4, 14.3. **GC-MS** (EI, m/z) for $C_{17}H_{15}N$ Calcd: 233.1, found: 233.1. Spectra data are consistent with the reported literature.[9]

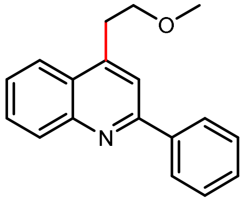

**4-(2-methoxyethyl)-2-phenylquinoline (16)**. Following the general procedure A, the product was isolated by preparative TLC with Hex/EtOAc (10:1) as the eluent to give a colorless oil (38.4 mg, 73%). **$^1$H NMR** (500 MHz, $CDCl_3$) δ 8.23 – 8.16 (m, 3H), 8.08 (d, *J* = 8.4 Hz, 1H), 7.80 (s, 1H), 7.77 – 7.71 (m, 1H), 7.60 – 7.53 (m, 3H), 7.51 – 7.46 (m, 1H), 3.84 (t, *J* = 7.0 Hz, 2H), 3.45 (t, *J* = 7.0 Hz, 2H), 3.43 (s, 3H). **$^{13}$C NMR** (125 MHz, $CDCl_3$) δ 157.1, 148.5, 145.4, 139.8, 130.6, 129.3, 129.2, 128.8, 127.6, 126.7, 126.2, 123.3, 119.5, 72.1, 58.9, 32.8. **HRMS** (M+H$^+$) for $C_{18}H_{18}NO$ Calcd: 264.1383, found: 264.1371. The compound was not reported.

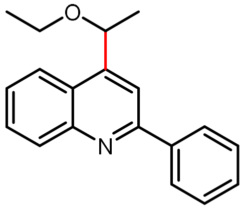

**4-(1-ethoxyethyl)-2-phenylquinoline (17)**. Following the general procedure A, the reaction was run with 1.2 equiv of TFA for 36 h, and the product was isolated by preparative TLC with Hex/EtOAc (10:1) as the eluent to give a colorless oil (33.6 mg, 66%). **$^1$H NMR** (500 MHz, $CDCl_3$) δ 8.27 – 8.19 (m, 3H), 8.13 (d, *J* = 8.4 Hz, 1H), 8.03 (s, 1H), 7.77 – 7.72 (m, 1H), 7.59 – 7.54 (m, 3H), 7.52 – 7.47 (m, 1H), 5.22 (q, *J* = 6.6 Hz, 1H), 3.58 – 3.49 (m, 2H), 1.67 (d, *J* = 6.6 Hz, 3H), 1.31 (t, *J* = 7.0 Hz, 3H). **$^{13}$C NMR** (125 MHz, $CDCl_3$) δ 157.4, 150.3, 148.7, 139.8, 130.7, 129.3, 129.2, 128.8, 127.6, 126.1, 125.1, 122.9, 115.4, 74.6, 64.8, 23.5, 15.6. **GC-MS** (EI, m/z) for $C_{19}H_{19}NO$ Calcd: 277.1, found: 277.1. Spectra data are consistent with the reported literature.[3]

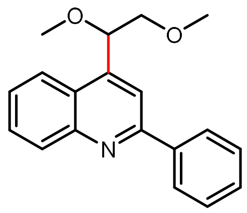

**4-(1,2-dimethoxyethyl)-2-phenylquinoline (18)**. Following the general procedure A, the reaction was run with 1.2 equiv of TFA for 36 h, and the product was isolated by preparative TLC with Hex/EtOAc (10:1) as the eluent to give a colorless oil (39.3 mg, 67%). **$^1$H NMR** (500 MHz, $CDCl_3$) δ 8.28 – 8.21 (m, 3H), 8.13 (d, *J* = 6.7 Hz, 1H), 8.04 (s, 1H), 7.79 – 7.74 (m, 1H), 7.62 – 7.54 (m, 3H), 7.53 – 7.47 (m, 1H), 5.25 (dd, *J* = 7.7, 3.0 Hz, 1H), 3.79 – 3.67 (m, 2H), 3.47 (s, 6H). **$^{13}$C NMR** (125 MHz, $CDCl_3$) δ 157.2, 148.7, 144.8, 139.6, 130.8, 129.5, 129.4, 128.9, 127.6, 126.5, 125.4, 122.6, 116.7, 80.1, 76.5, 59.4, 57.8. **GC-MS** (EI, m/z) for $C_{19}H_{19}NO_2$ Calcd: 293.1, found: 293.1. Spectra data are consistent with the reported literature.[3]

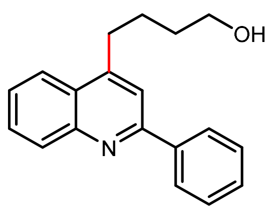

**4-(2-phenylquinolin-4-yl)butan-1-ol (19)**. Following the general procedure A, the reaction was run with 1.2 equiv of TFA, and the product was isolated by preparative TLC with Hex/EtOAc (5:3) as the eluent to give a colorless oil (51.0 mg, 92%). **$^1$H NMR** (500 MHz, $CDCl_3$) δ 8.22 (d, *J* = 8.4 Hz, 1H), 8.16 – 8.12 (m, 2H), 8.06 – 8.00 (m, 1H), 7.75 – 7.67 (m, 2H), 7.57 – 7.51 (m, 3H), 7.50 – 7.44 (m, 1H), 3.75 – 3.62 (m, 2H), 3.20 – 3.08 (m, 2H), 2.04 – 1.84 (m, 3H), 1.77 – 1.67 (m, 2H). **$^{13}$C NMR** (125 MHz, $CDCl_3$) δ 157.2, 148.9, 148.5, 139.9, 130.4, 129.3, 129.2, 128.8, 127.6, 126.5, 126.1, 123.4, 118.8, 62.4, 32.6, 32.2, 26.3. **GC-MS** (EI, m/z) for $C_{19}H_{19}N$ Calcd: 277.1, found: 277.1. Spectra data are consistent with the reported literature.[3]

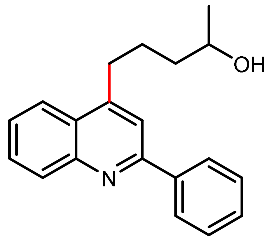

**5-(2-phenylquinolin-4-yl)pentan-2-ol (20)**. Following the general procedure A, the reaction was run with 2 equiv of TFA, and the product was isolated by preparative TLC with Hex/EtOAc (5:2) as the eluent to give a colorless oil (45.5 mg, 78%). **$^1$H NMR** (500 MHz, $CDCl_3$) δ 8.22 (d, *J* = 8.4 Hz, 1H), 8.18 – 8.13 (m, 2H), 8.06 – 8.02 (m, 1H), 7.75 – 7.70 (m, 2H), 7.57 – 7.51 (m, 3H), 7.50 – 7.45 (m, 1H), 3.92 – 3.81 (m, 1H), 3.21 – 3.07 (m, 2H), 2.00 – 1.70 (m, 3H), 1.68 – 1.54 (m, 2H), 1.22 (d, *J* = 6.1 Hz, 3H). **$^{13}$C NMR** (125 MHz, $CDCl_3$) δ 157.1, 148.9, 148.5, 139.9, 130.5, 129.3, 129.2, 128.8, 127.6, 126.5, 126.1, 123.4, 118.8, 67.8, 39.1, 32.4, 26.3, 23.7. **HRMS** ($M+H^+$) for $C_{20}H_{22}NO$ Calcd: 292.1696, found: 292.1682. The compound was not reported.

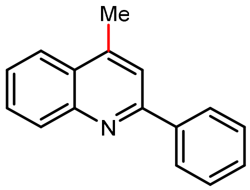

**4-methyl-2-phenylquinoline (21)**. Following the general procedure A, the reaction was run with 1.2 equiv of TFA, and the product was isolated by preparative TLC with Hex/EtOAc (10:1) as the eluent to give a colorless oil (37.3 mg, 85%). **$^{1}$H NMR** (500 MHz, $CDCl_3$) δ 8.23 – 8.16 (m, 3H), 8.03 (d, *J* = 7.1 Hz, 1H), 7.77 – 7.72 (m, 2H), 7.60 – 7.53 (m, 3H), 7.51 – 7.46 (m, 1H), 2.80 (s, 3H). **$^{13}$C NMR** (125 MHz, $CDCl_3$) δ 157.1, 148.2, 144.8, 139.9, 130.3, 129.3, 129.2, 128.8, 127.6, 127.3, 126.0, 123.6, 119.8, 19.0. **GC-MS** (EI, m/z) for $C_{16}H_{13}N$ Calcd: 219.1, found: 219.1. Spectra data are consistent with the reported literature.[1]

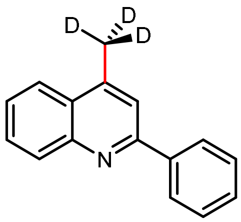

**4-(methyl-*d*$_3$)-2-phenylquinoline (22)**. Following the general procedure A, the product was isolated by preparative TLC with Hex/EtOAc (10:1) as the eluent to give a colorless oil (35.1 mg, 79%). **$^{1}$H NMR** (500 MHz, $CDCl_3$) δ 8.23 – 8.15 (m, 3H), 8.02 (dd, *J* = 8.4, 1.4 Hz, 1H), 7.78 – 7.71 (m, 2H), 7.60 – 7.51 (m, 3H), 7.51 – 7.45, (m, 1H). **$^{13}$C NMR** (125 MHz, $CDCl_3$) δ 157.1, 148.2, 144.7, 139.9, 130.3, 129.3, 129.2, 128.8, 127.6, 127.3, 126.0, 123.6, 119.8. HRMS ($M+Na^+$) for $C_{16}H_{10}D_3NNa$ Calcd: 245.1129, found: 245.1125. The compound was not reported.

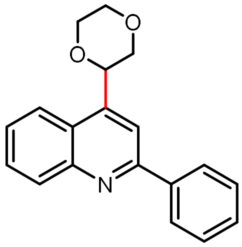

**4-(1,4-dioxan-2-yl)-2-phenylquinoline (23)**. Following the general procedure A, the reaction was run with 2 equiv of TFA, and the product was isolated by preparative TLC with Hex/EtOAc (5:1) as the eluent to give a white solid (36.7 mg, 63%). **$^{1}$H NMR** (500 MHz, $CDCl_3$) δ 8.26 – 8.21 (m, 3H), 8.13 (s, 1H), 8.03 (d, *J* = 8.4 Hz, 1H), 7.78 – 7.73 (m, 1H), 7.60 – 7.53 (m, 3H), 7.52 – 7.47 (m, 1H), 5.45 (dd, *J* = 9.9, 2.0 Hz, 1H), 4.21 (dd, *J* = 11.9, 2.7 Hz, 1H), 4.17 – 4.08 (m, 2H), 3.96 – 3.91 (m, 1H), 3.90 – 3.83 (m, 1H), 3.54 (dd, *J* = 11.9, 9.9 Hz, 1H). **$^{13}$C NMR** (125 MHz, $CDCl_3$) δ 157.4, 148.3, 144.1, 139.6, 130.8, 129.4, 129.4, 128.8, 127.6, 126.5, 124.2, 122.3, 116.2, 74.4, 72.1, 67.4, 66.7. **GC-MS** (EI, m/z) for $C_{19}H_{17}NO_2$ Calcd: 291.1, found: 291.1. Spectra data are consistent with the reported literature.[3]

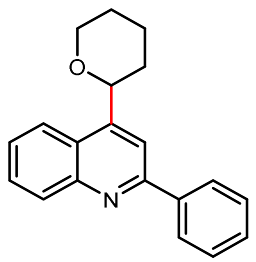

**2-phenyl-4-(tetrahydro-2*H*-pyran-2-yl)quinoline (24)**. Following the general procedure A, the reaction was run with 2 equiv of TFA for 36 h, and the product was isolated by preparative TLC with Hex/EtOAc (5:1) as the eluent to give a colorless oil (41.1 mg, 71%). **$^1$H NMR** (500 MHz, $CDCl_3$) δ 8.26 – 8.19 (m, 3H), 8.09 (s, 1H), 8.01 (d, *J* = 10.1 Hz, 1H), 7.76 – 7.70 (m, 1H), 7.58 – 7.51 (m, 3H), 7.51 – 7.45 (m, 1H), 5.12 (dd, *J* = 11.2, 2.1 Hz, 1H), 4.35 – 4.27 (m, 1H), 3.85 – 3.76 (m, 1H), 2.18 – 2.11 (m, 1H), 2.09 – 2.01 (m, 1H), 1.92 – 1.79 (m, 2H), 1.76 – 1.64 (m, 2H). **$^{13}$C NMR** (125 MHz, $CDCl_3$) δ 157.5, 149.2, 148.5, 139.9, 130.7, 129.2, 129.1, 128.7, 127.7, 126.0, 124.3, 122.9, 115.4, 76.4, 69.3, 33.8, 26.0, 24.1. **GC-MS** (EI, m/z) for $C_{20}H_{19}NO$ Calcd: 289.1, found: 289.1. Spectra data are consistent with the reported literature.[3]

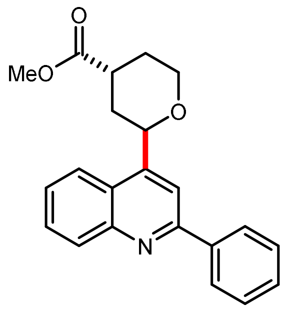

**methyl 2-(*trans*-2-phenylquinolin-4-yl)tetrahydro-2*H*-pyran-4-carboxylate (25)**. Following the general procedure A, the reaction was run with 2 equiv of TFA for 36 h, and the product was isolated by preparative TLC with Hex/EtOAc (5:1) as the eluent to give a white solid (43.1 mg, 62%). **$^1$H NMR** (500 MHz, $CDCl_3$) δ 8.25 – 8.20 (m, 3H), 8.17 (d, *J* = 10.1 Hz, 1H), 8.10 (s, 1H), 7.76 – 7.71 (m, 1H), 7.62 – 7.57 (m, 1H), 7.57 – 7.52 (m, 2H), 7.50 – 7.45 (m, 1H), 5.41 (d, *J* = 13.7 Hz, 1H), 4.22 (dd, *J* = 12.1, 4.7 Hz, 1H), 3.93 – 3.81 (m, 4H), 3.08 – 2.99 (m, 1H), 2.67 – 2.58 (m, 1H), 2.25 – 2.07 (m, 2H), 1.83 – 1.73 (m, 1H). **$^{13}$C NMR** (125 MHz, $CDCl_3$) δ 175.0, 157.4, 149.0, 148.4, 139.9, 130.6, 129.2, 129.2, 128.7, 127.7, 126.3, 124.2, 122.9, 114.9, 72.9, 65.9, 52.1, 37.5, 34.6, 27.4. **GC-MS** (EI, m/z) for $C_{22}H_{21}NO_3$ Calcd: 347.2, found: 347.1. Spectra data are consistent with the reported literature.[3]

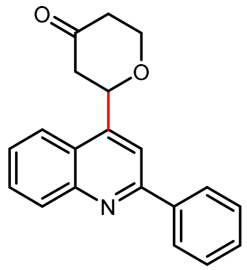

**2-(2-phenylquinolin-4-yl)tetrahydro-4*H*-pyran-4-one (26)**. Following the general procedure A, the reaction was run with 2 equiv of TFA for 36 h, and the product was isolated by preparative TLC with Hex/EtOAc (5:2) as the eluent to give a white solid (39.4 mg, 65%). **$^1$H NMR** (500 MHz, $CDCl_3$) δ 8.27 –

8.21 (m, 3H), 8.15 (s, 1H), 7.93 (d, $J$ = 8.4 Hz, 1H), 7.78 – 7.73 (m, 1H), 7.60 – 7.54 (m, 3H), 7.52 – 7.47 (m, 1H), 5.44 (dd, $J$ = 11.4, 2.7 Hz, 1H), 4.60 (ddd, $J$ = 11.7, 7.5, 1.5 Hz, 1H), 4.04 (td, $J$ = 12.0, 2.9 Hz, 1H), 2.96 (dt, $J$ = 14.7, 2.4 Hz, 1H), 2.91 – 2.82 (m, 1H), 2.76 – 2.68 (m, 1H), 2.61 – 2.55 (m, 1H). **$^{13}$C NMR** (125 MHz, $CDCl_3$) δ 205.4, 157.4, 148.6, 146.3, 139.6, 130.9, 129.5, 129.5, 128.9, 127.6, 126.6, 123.6, 122.3, 115.2, 76.0, 67.0, 49.3, 42.2. **HRMS** (M+H$^+$) for $C_{20}H_{18}NO_2$ Calcd: 304.1332, found: 304.1331. The compound was not reported.

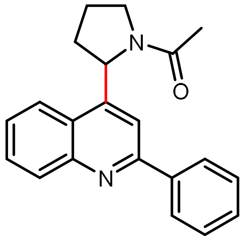


**1-(2-(2-phenylquinolin-4-yl)pyrrolidin-1-yl)ethan-1-one, 1:0.65 rotamers (27)**. Following the general procedure A, the product was isolated by preparative TLC with Hex/EtOAc (1:3) as the eluent to give a yellow oil (48.1 mg, 76%). **$^1$H NMR** (500 MHz, $CDCl_3$) δ 8.27 (d, $J$ = 8.4 Hz, 1H), 8.22 (d, $J$ = 7.9 Hz, 0.65H), 8.17 – 8.10 (m, 2H), 8.12 – 8.05 (m, 1.3H), 8.03 (d, $J$ = 9.7 Hz, 0.65H), 8.00 (d, $J$ = 8.3 Hz, 1H), 7.83 – 7.77 (m, 1H), 7.75 – 7.70 (m, 0.65H), 7.65 – 7.60 (m, 2H), 7.57 – 7.45 (m, 3H+3.25H), 6.00 (d, $J$ = 9.4 Hz, 0.65H), 5.72 (d, $J$ = 7.4 Hz, 1H), 4.01 – 3.90 (m, 1H+0.65H), 3.84 – 3.70 (m, 1H+0.65H), 2.72 – 2.61 (m, 1H), 2.55 – 2.47 (m, 0.65H), 2.28 (s, 1.95H), 2.19 – 1.97 (m, 3H+1.95H), 1.92 (s, 3H). **$^{13}$C NMR** (125 MHz, $CDCl_3$) δ 170.2, 169.5, 157.3, 157.2, 148.8, 148.8, 148.4, 148.3, 140.2, 139.3, 131.0, 130.6, 129.7, 129.7, 129.3, 129.2, 129.0, 128.8, 127.7, 127.7, 126.7, 126.2, 124.7, 124.2, 123.2, 122.3, 114.8, 114.4, 58.9, 57.3, 48.6, 47.0, 34.5, 32.7, 24.0, 22.8, 22.5, 22.2. **GC-MS** (EI, m/z) for $C_{21}H_{20}N_2O$ Calcd: 316.2, found: 316.1. Spectra data are consistent with the reported literature.[3]

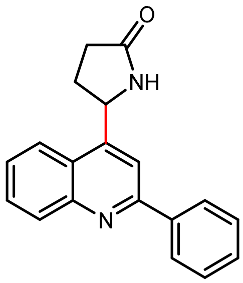


**5-(2-phenylquinolin-4-yl)pyrrolidin-2-one (28)**. Following the general procedure A, the product was isolated by preparative TLC with Hex/EtOAc/MeOH (2:1:0.5) as the eluent to give a white solid (38.1 mg, 66%). **$^1$H NMR** (500 MHz, $CDCl_3$) δ 8.27 (d, $J$ = 6.9 Hz, 1H), 8.14 – 8.08 (m, 2H), 7.88 (d, $J$ = 8.4 Hz, 1H), 7.84 (s, 1H), 7.81 – 7.75 (m, 1H), 7.62 – 7.56 (m, 1H), 7.52 – 7.43 (m, 3H), 6.85 (s, 1H), 5.50 (dd, $J$ = 8.6, 5.3 Hz, 1H), 2.94 – 2.83 (m, 1H), 2.49 (t, $J$ = 8.1 Hz, 2H), 2.11 – 2.01 (m, 1H). **$^{13}$C NMR** (125 MHz, $CDCl_3$) δ 178.8, 157.3, 148.6, 148.5, 139.3, 130.9, 129.7, 129.6, 128.9, 127.6, 126.7, 124.2, 122.1, 113.6, 53.9, 29.7, 29.6. **HRMS** (M+Na$^+$) for $C_{19}H_{16}N_2NaO$ Calcd: 311.1155, found: 311.1158. The compound was not reported.

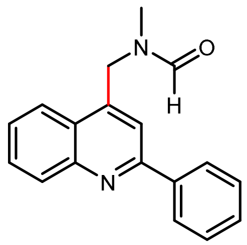

***N*-methyl-*N*-((2-phenylquinolin-4-yl)methyl)formamide, 1:0.5 rotamers (29).** Following the general procedure A, the product was isolated by preparative TLC with Hex/EtOAc/MeOH (1:1:0.2) as the eluent to give a yellow oil (41.5 mg, 75%). **$^1$H NMR** (500 MHz, $CDCl_3$) δ 8.43 (s, 0.5H), 8.30 – 8.19 (m, 2H+0.5H), 8.21 – 8.14 (m, 2H+1H), 8.09 (d, *J* = 8.7 Hz, 1H), 7.92 (d, *J* = 8.4 Hz, 0.5H), 7.83 – 7.72 (m, 2H+0.5H), 7.66 (s, 0.5H), 7.63 – 7.53 (m, 3H+1.5H), 7.52 – 7.48 (m, 1H+0.5H), 5.07 (s, 2H), 4.98 (s, 1H), 2.99 (s, 1.5H), 2.91 (s, 3H). **$^{13}$C NMR** (125 MHz, $CDCl_3$) δ 163.4, 162.6, 157.3, 157.0, 148.6, 148.5, 141.9, 141.7, 139.4, 139.1, 130.9, 130.5, 130.0, 129.9, 129.7, 129.5, 129.0, 128.9, 127.6, 127.6, 126.9, 126.9, 125.6, 125.1, 123.2, 121.8, 118.9, 116.7, 50.1, 45.2, 34.4, 30.5. **HRMS** (M+H$^+$) for $C_{18}H_{17}N_2O$ Calcd: 277.1335, found: 277.1330. The compound was not reported.

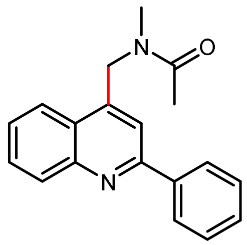

***N*-methyl-*N*-((2-phenylquinolin-4-yl)methyl)acetamide, 1:0.5 rotamers (30).** Following the general procedure A, the product was isolated by preparative TLC with Hex/EtOAc (1:1) as the eluent to give a yellow oil (41.8 mg, 72%). **$^1$H NMR** (500 MHz, $CDCl_3$) δ 8.28 (d, *J* = 10.2 Hz, 0.5H), 8.23 (d, *J* = 10.4 Hz, 1H), 8.19 – 8.12 (m, 2H+1H), 8.09 (d, *J* = 6.4 Hz, 1H), 7.90 (d, *J* = 8.2 Hz, 0.5H), 7.84 – 7.79 (m, 0.5H), 7.79 – 7.74 (m, 1H), 7.70 (s, 1H), 7.65 – 7.54 (m, 3H+2H), 7.53 – 7.49 (m, 1H+0.5H), 5.17 (s, 2H), 5.10 (s, 1H), 3.17 (s, 1.5H), 3.00 (s, 3H), 2.27 (s, 3H), 2.18 (s, 1.5H). **$^{13}$C NMR** (125 MHz, $CDCl_3$) δ 171.5, 170.8, 157.6, 157.1, 148.6, 148.4, 143.1, 142.4, 139.6, 139.2, 130.9, 130.5, 129.9, 129.7, 129.7, 129.4, 129.0, 128.9, 127.6, 127.6, 126.8, 126.8, 125.8, 124.9, 123.3, 121.6, 118.5, 114.7, 51.5, 47.9, 35.7, 34.7, 21.9, 21.3. **HRMS** (M+Na$^+$) for $C_{19}H_{18}N_2NaO$ Calcd: 313.1311, found: 313.1299. The compound was not reported.

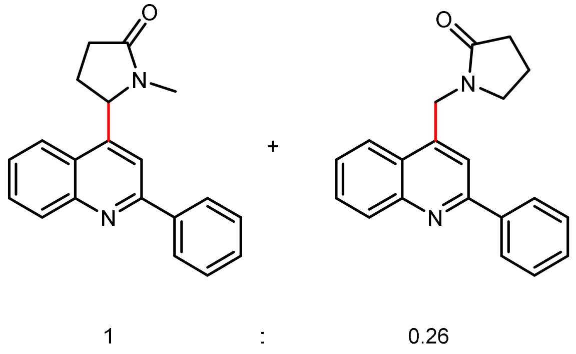

**1-methyl-5-(2-phenylquinolin-4-yl)pyrrolidin-2-one (31a) and 1-((2-phenylquinolin-4-yl)methyl)pyrrolidin-2-one (31b), 1:0.26 congeners**. Following the general procedureA, the product was isolated by preparative TLC with Hex/EtOAc/MeOH (2:1:0.4) as the eluent to give a yellow oil (34.5 mg, 57%). $^1$H NMR (500 MHz, $CDCl_3$) δ 8.28 (d, *J* = 8.4 Hz, 1H), 8.23 (d, J = 8.4 Hz, 0.26H), 8.19 – 8.13 (m, 2H+0.52H), 7.96 (d, *J* = 8.5 Hz, 1H), 7.82 – 7.74 (m, 1H+0.52H), 7.65 – 7.53 (m, 4H+0.78H), 7.53 – 7.46 (m, 1H+0.52H), 5.41 (s, 1H), 4.99 (s, 0.52H), 3.28 (t, *J* = 7.0 Hz, 0.52H), 2.95 (s, 3H), 2.82 – 2.71 (m, 1H), 2.65 – 2.48 (m, 2H+0.52H), 2.09 – 1.94 (m, 1H+0.52H). $^{13}$C NMR (125 MHz, $CDCl_3$) δ 176.0, 174.9, 157.4, 157.0, 149.0, 148.6, 146.7, 142.4, 139.4, 139.2, 131.0, 130.5, 129.9, 129.8, 129.7, 129.5, 129.0, 128.9, 128.9, 127.6, 127.5, 127.0, 126.8, 125.7, 124.7, 123.4, 122.0, 119.1, 113.9, 60.0, 46.8, 44.2, 30.8, 29.5, 29.1, 27.2, 17.8. HRMS ($M+H^+$) for $C_{20}H_{18}N_2NaO$ Calcd: 325.1311, found: 325.1309. The compound was not reported.

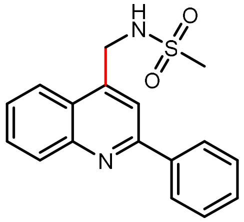

***N*-((2-phenylquinolin-4-yl)methyl)methanesulfonamide (32)**. Following the general procedure A, the product was isolated by preparative TLC with Hex/EtOAc/MeOH (2:1:0.1) as the eluent to give a yellow solid (41.9 mg, 67%). **$^1$H NMR** (500 MHz, $CDCl_3$) δ 8.23 (d, *J* = 9.2 Hz, 1H), 8.16 – 8.12 (m, 2H), 7.97 (d, *J* = 7.7 Hz, 1H), 7.88 (s, 1H), 7.79 – 7.74 (m, 1H), 7.62 – 7.57 (m, 1H), 7.56 – 7.47 (m, 3H), 5.09 (t, *J* = 6.2 Hz, 1H), 4.82 (d, *J* = 0.9 Hz, 2H), 2.99 (s, 3H). **$^{13}$C NMR** (125 MHz, $CDCl_3$) δ 157.1, 148.4, 142.3, 139.1, 130.7, 129.9, 129.7, 128.9, 127.5, 127.0, 124.9, 122.4, 117.8, 44.1, 41.2. **GC-MS** (EI, m/z) for $C_{17}H_{16}N_2O_2S$ Calcd: 312.1, found: 312.0. Spectra data are consistent with the reported literature.[3]

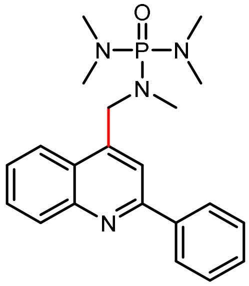

***N*,*N'*,*N'*,*N''*,*N''*-pentamethyl-*N*-((2-phenylquinolin-4-yl)methyl)phosphoramide (33)**. Following the general procedure A, the product was isolated by preparative TLC with Hex/EtOAc/MeOH (1:1:0.5) as the eluent to give a yellow oil (45.1 mg, 59%). **$^1$H NMR** (500 MHz, $CDCl_3$) δ 8.28 (d, *J* = 8.3 Hz, 1H), 8.23 – 8.19 (m, 3H), 7.99 (s, 1H), 7.75 – 7.70 (m, 1H), 7.58 – 7.52 (m, 3H), 7.50 – 7.45 (m, 1H), 4.74 (d, *J* = 8.0 Hz, 2H), 2.70 (d, *J* = 9.5 Hz, 12H), 2.67 (d, *J* = 9.0 Hz, 3H). **$^{13}$C NMR** (125 MHz, $CDCl_3$) δ 157.0, 148.6, 144.5, 139.7, 130.4, 129.5, 129.4, 128.9, 127.5, 126.3, 126.0, 123.3, 117.9, 50.4, 50.4, 36.9, 36.9, 34.9, 34.8. **$^{31}$P NMR** (203 MHz, $CDCl_3$) δ 25.2 (s, 1P). **GC-MS** (EI, m/z) for $C_{21}H_{27}N_4OP$ Calcd: 382.2, found: 382.2. Spectra data are consistent with the reported literature.[3]

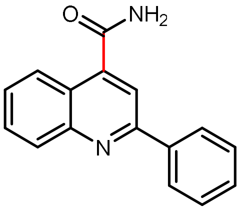


**2-phenylquinoline-4-carboxamide (34)**. Following the general procedure A, the product was isolated by preparative TLC with Hex/EtOAc/MeOH (1:1:0.5) as the eluent to give a white solid (30.3 mg, 61%). **$^1$H NMR** (500 MHz, $CDCl_3$) δ 8.28 (d, *J* = 10.1 Hz, 1H), 8.22 (d, *J* = 8.4 Hz, 1H), 8.18 – 8.15 (m, 2H), 7.98 (s, 1H), 7.81 – 7.76 (m, 1H), 7.63 – 7.58 (m, 1H), 7.58 – 7.48 (m, 3H), 6.22 (s, 2H). **$^{13}$C NMR** (125 MHz, $CDCl_3$) δ 169.4, 156.8, 148.8, 141.7, 138.7, 130.3, 130.2, 129.8, 129.0, 127.5, 127.5, 124.9, 123.1, 116.6. **GC-MS** (EI, m/z) for $C_{16}H_{12}N_2O$ Calcd: 248.1, found: 248.1. Spectra data are consistent with the reported literature.[10]

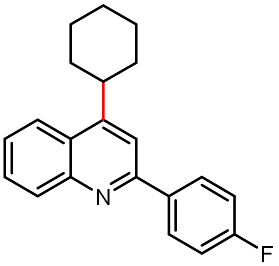


**4-cyclohexyl-2-(4-fluorophenyl)quinoline (35)**. Following the general procedure A, the product was isolated by preparative TLC with Hex/EtOAc (10:1) as the eluent to give a white solid (40.9 mg, 67%). **$^1$H NMR** (500 MHz, $CDCl_3$) δ 8.21 – 8.15 (m, 3H), 8.11 (d, *J* = 8.5 Hz, 1H), 7.75 – 7.70 (m, 2H), 7.58 – 7.53 (m, 1H), 7.26 – 7.21 (m, 2H), 3.45 – 3.35 (m, 1H), 2.14 – 2.08 (m, 2H), 2.02 – 1.96 (m, 2H), 1.94 – 1.87 (m, 1H), 1.70 – 1.55 (m, 4H), 1.46 – 1.34 (m, 1H). **$^{13}$C NMR** (125 MHz, $CDCl_3$) δ 163.7 (d, *J* = 249 Hz), 156.2, 154.1, 148.6, 136.4 (d, *J* = 3 Hz), 130.6, 129.3 (d, *J* = 33 Hz), 129.1, 126.0, 125.8, 122.9, 115.7 (d, *J* = 22 Hz), 115.1, 39.1, 33.7, 27.0, 26.3. **$^{19}$F NMR** (470 MHz, $CDCl_3$) δ -112.86 – -112.94 (m, 1F). **HRMS** ($M+H^+$) for $C_{21}H_{21}FN$ Calcd: 306.1653, found: 306.1644. The compound was not reported.

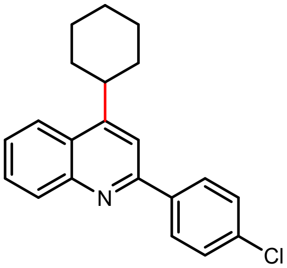


**2-(4-chlorophenyl)-4-cyclohexylquinoline (36)**. Following the general procedure A, the product was isolated by preparative TLC with Hex/EtOAc (10:1) as the eluent to give a white solid (44.4 mg, 69%). **$^1$H NMR** (500 MHz, $CDCl_3$) δ 8.19 (dd, *J* = 8.4, 1.3 Hz, 1H), 8.16 – 8.09 (m, 3H), 7.76 – 7.70 (m, 2H), 7.60 – 7.55 (m, 1H), 7.55 – 7.48 (m, 2H), 3.44 – 3.35 (m, 1H), 2.16 – 2.07 (m, 2H), 2.03 – 1.96 (m, 2H), 1.95 – 1.87 (m, 1H), 1.70 – 1.54 (m, 4H), 1.46 – 1.35 (m, 1H). **$^{13}$C NMR** (125 MHz, $CDCl_3$) δ 156.0, 154.2, 148.6, 138.7, 135.3, 130.7, 129.2, 128.9, 128.9, 126.1, 125.9, 122.9, 115.0, 39.2, 33.7, 27.0, 26.3. **HRMS** ($M+H^+$) for $C_{21}H_{21}ClN$ Calcd: 322.1357, found: 322.1351. The compound was not reported.

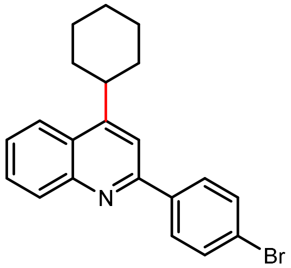


**2-(4-bromophenyl)-4-cyclohexylquinoline (37)**. Following the general procedure A, the product was isolated by preparative TLC with Hex/EtOAc (10:1) as the eluent to give a white solid (54.9 mg, 75%). **$^{1}$H NMR** (500 MHz, $CDCl_3$) δ 8.19 (d, *J* = 8.4 Hz, 1H), 8.11 (d, *J* = 6.9 Hz, 1H), 8.09 – 8.04 (m, 2H), 7.76 – 7.70 (m, 2H), 7.70 – 7.64 (m, 2H), 7.59 – 7.54 (m, 1H), 3.44 – 3.36 (m, 1H), 2.15 – 2.06 (m, 2H), 2.02 – 1.95 (m, 2H), 1.95 – 1.87 (m, 1H), 1.71 – 1.55 (m, 4H), 1.46 – 1.36 (m, 1H). **$^{13}$C NMR** (125 MHz, $CDCl_3$) δ 156.0, 154.3, 148.6, 139.1, 131.9, 130.7, 129.2, 129.2, 126.1, 126.0, 123.7, 122.9, 115.0, 39.2, 33.7, 27.0, 26.3. **HRMS** ($M+H^+$) for $C_{21}H_{21}BrN$ Calcd: 366.0852, found: 366.0836. The compound was not reported.

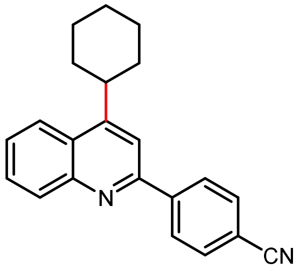


**4-(4-cyclohexylquinolin-2-yl)benzonitrile (38)**. Following the general procedure A, the product was isolated by preparative TLC with Hex/EtOAc (5:1) as the eluent to give a white solid (33.7 mg, 54%). **$^{1}$H NMR** (500 MHz, $CDCl_3$) δ 8.31 (d, *J* = 8.7 Hz, 2H), 8.21 (d, *J* = 8.4 Hz, 1H), 8.14 (d, *J* = 8.5 Hz, 1H), 7.83 (d, *J* = 8.0 Hz, 2H), 7.79 – 7.73 (m, 2H), 7.64 – 7.58 (m, 1H), 3.47 – 3.37 (m, 1H), 2.15 – 2.07 (m, 2H), 2.04 – 1.96 (m, 2H), 1.96 – 1.87 (m, 1H), 1.71 – 1.54 (m, 4H), 1.47 – 1.38 (m, 1H). **$^{13}$C NMR** (125 MHz, $CDCl_3$) δ 154.9, 154.8, 148.6, 144.3, 132.6, 130.9, 129.5, 128.1, 126.7, 126.2, 123.0, 118.9, 115.1, 112.5, 39.2, 33.7, 26.9, 26.3. **HRMS** ($M+H^+$) for $C_{22}H_{21}N_2$ Calcd: 313.1699, found: 313.1689. The compound was not reported.

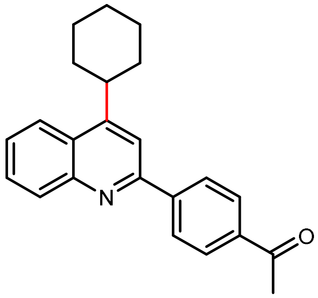


**1-(4-(4-cyclohexylquinolin-2-yl)phenyl)ethan-1-one (39)**. Following the general procedure A, the product was isolated by preparative TLC with Hex/EtOAc (5:2) as the eluent to give a white solid (38.9

mg, 59%). **$^1$H NMR** (500 MHz, $CDCl_3$) δ 8.27 (d, *J* = 8.4 Hz, 2H), 8.21 (d, *J* = 6.6 Hz, 1H), 8.15 – 8.10 (m, 3H), 7.80 (s, 1H), 7.76 – 7.71 (m, 1H), 7.61 – 7.56 (m, 1H), 3.46 – 3.37 (m, 1H), 2.69 (s, 3H), 2.14 – 2.06 (m, 2H), 2.02 – 1.94 (m, 2H), 1.94 – 1.86 (m, 1H), 1.71 – 1.55 (m, 4H), 1.46 – 1.36 (m, 1H). **$^{13}$C NMR** (125 MHz, $CDCl_3$) δ 198.0, 155.9, 154.4, 148.6, 144.5, 137.2, 130.8, 129.3, 128.8, 127.8, 126.4, 126.2, 122.9, 115.4, 39.2, 33.7, 27.0, 26.8, 26.3. **HRMS** ($M+H^+$) for $C_{23}H_{24}NO$ Calcd: 330.1852, found: 330.1841. The compound was not reported.

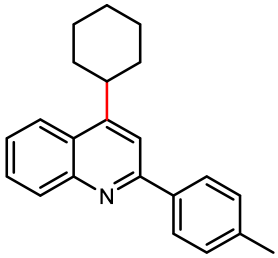


**4-cyclohexyl-2-(*p*-tolyl)quinoline (40)**. Following the general procedure A, the product was isolated by preparative TLC with Hex/EtOAc (10:1) as the eluent to give a colorless oil (42.2 mg, 70%). **$^1$H NMR** (500 MHz, $CDCl_3$) δ 8.21 (d, *J* = 8.4 Hz, 1H), 8.13 – 8.06 (m, 3H), 7.77 (s, 1H), 7.74 – 7.68 (m, 1H), 7.57 – 7.52 (m, 1H), 7.36 (d, *J* = 8.0 Hz, 2H), 3.45 – 3.34 (m, 1H), 2.47 (s, 3H), 2.15 – 2.07 (m, 2H), 2.03 – 1.95 (m, 2H), 1.94 – 1.86 (m, 1H), 1.70 – 1.55 (m, 4H), 1.45 – 1.35 (m, 1H). **$^{13}$C NMR** (125 MHz, $CDCl_3$) δ 157.3, 153.8, 148.6, 139.2, 137.5, 130.6, 129.5, 128.9, 127.5, 125.8, 125.7, 122.8, 115.3, 39.1, 33.7, 27.0, 26.4, 21.4. **HRMS** ($M+H^+$) for $C_{22}H_{24}N$ Calcd: 302.1903, found: 302.1892. The compound was not reported.

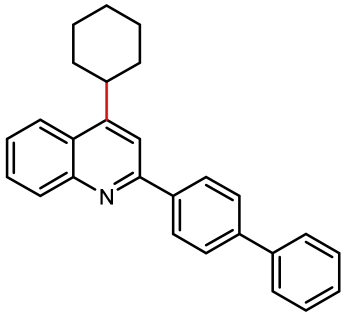


**2-([1,1'-biphenyl]-4-yl)-4-cyclohexylquinoline (41)**. Following the general procedure A, the product was isolated by preparative TLC with Hex/EtOAc (10:1) as the eluent to give a white solid (39.3 mg, 54%). **$^1$H NMR** (500 MHz, $CDCl_3$) δ 8.28 (d, *J* = 6.7 Hz, 2H), 8.25 (d, *J* = 10.1 Hz, 1H), 8.13 (d, *J* = 8.5 Hz, 1H), 7.84 (s, 1H), 7.80 (d, *J* = 8.5 Hz, 2H), 7.77 – 7.69 (m, 3H), 7.60 – 7.55 (m, 1H), 7.54 – 7.49 (m, 2H), 7.44 – 7.39 (m, 1H), 3.47 – 3.37 (m, 1H), 2.17 – 2.10 (m, 2H), 2.03 – 1.97 (m, 2H), 1.97 – 1.88 (m, 1H), 1.75 – 1.57 (m, 4H), 1.48 – 1.37 (m, 1H). **$^{13}$C NMR** (125 MHz, $CDCl_3$) δ 156.9, 154.0, 148.7, 141.9, 140.7, 139.2, 130.7, 129.1, 128.9, 128.0, 127.6, 127.5, 127.2, 126.0, 125.9, 122.9, 115.4, 39.2, 33.7, 27.0, 26.4. **HRMS** ($M+H^+$) for $C_{27}H_{26}N$ Calcd: 364.2060, found: 364.2049. The compound was not reported.

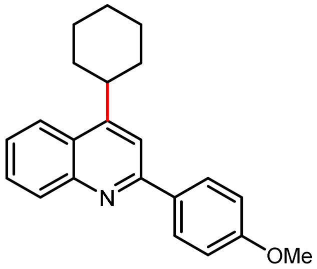


**4-cyclohexyl-2-(4-methoxyphenyl)quinoline (42)**. Following the general procedure A, the reaction was run at 55-60 °C, and the product was isolated by preparative TLC with Hex/EtOAc (10:1) as the eluent to give a white solid (45.1 mg, 71%). **$^1$H NMR** (500 MHz, $CDCl_3$) δ 8.20 – 8.12 (m, 3H), 8.09 (d, $J$ = 8.4 Hz, 1H), 7.74 (s, 1H), 7.72 – 7.68 (m, 1H), 7.56 – 7.50 (m, 1H), 7.07 (d, $J$ = 8.8 Hz, 2H), 3.92 (s, 3H), 3.42 – 3.34 (m, 1H), 2.14 – 2.07 (m, 2H), 2.02 – 1.95 (m, 2H), 1.94 – 1.86 (m, 1H), 1.71 – 1.54 (m, 4H), 1.47 – 1.35 (m, 1H). **$^{13}$C NMR** (125 MHz, $CDCl_3$) δ 160.7, 156.9, 153.7, 148.6, 132.9, 130.5, 128.9, 128.9, 125.6, 125.5, 122.8, 115.0, 114.2, 55.4, 39.1, 33.7, 27.0, 26.4. **HRMS** (M+H$^+$) for $C_{22}H_{24}NO$ Calcd: 318.1852, found: 318.1850. The compound was not reported.

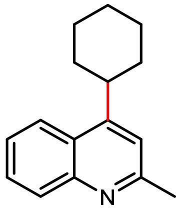


**4-cyclohexyl-2-methylquinoline (43)**. Following the general procedure A, the product was isolated by preparative TLC with Hex/EtOAc (10:1) as the eluent to give a colorless oil (28.8 mg, 64%). **$^1$H NMR** (500 MHz, $CDCl_3$) δ 8.06 (d, $J$ = 8.5 Hz, 2H), 7.70 – 7.65 (m, 1H), 7.54 – 7.48 (m, 1H), 7.19 (s, 1H), 3.37 – 3.27 (m, 1H), 2.74 (s, 3H), 2.07 – 1.99 (m, 2H), 1.99 – 1.91 (m, 2H), 1.91 – 1.85 (m, 1H), 1.62 – 1.50 (m, 4H), 1.42 – 1.32 (m, 1H). **$^{13}$C NMR** (125 MHz, $CDCl_3$) δ 158.8, 153.4, 148.1, 129.5, 128.8, 125.3, 125.2, 122.8, 118.3, 38.8, 33.6, 27.0, 26.3, 25.5. **GC-MS** (EI, m/z) for $C_{16}H_{19}N$ Calcd: 225.2, found: 225.1. Spectra data are consistent with the reported literature.[11]

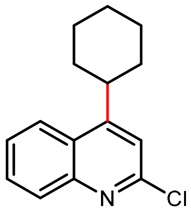


**2-chloro-4-cyclohexylquinoline (44)**. Following the general procedure A, the product was isolated by preparative TLC with Hex/EtOAc (10:1) as the eluent to give a white solid (21.1 mg, 43%). **$^1$H NMR** (500 MHz, $CDCl_3$) δ 8.08 – 8.02 (m, 2H), 7.75 – 7.68 (m, 1H), 7.61 – 7.56 (m, 1H), 7.28 (s, 1H), 3.36 – 3.27 (m, 1H), 2.08 – 1.92 (m, 4H), 1.92 – 1.83 (m, 1H), 1.61 – 1.49 (m 4H), 1.42 – 1.31 (m, 1H). **$^{13}$C NMR** (125 MHz, $CDCl_3$) δ 156.9, 151.1, 148.2, 130.0, 129.5, 126.5, 125.6, 123.2, 118.7, 39.1, 33.4, 26.8, 26.2. **GC-MS** (EI, m/z) for $C_{15}H_{16}ClN$ Calcd: 245.1, 247.1, found: 245.1, 247.0. Spectra data are consistent with the reported literature.[12]

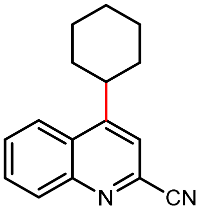

**4-cyclohexylquinoline-2-carbonitrile (45)**. Following the general procedure A, the product was isolated by preparative TLC with Hex/EtOAc (10:1) as the eluent to give a yellow solid (28.4 mg, 60%). **$^{1}$H NMR** (500 MHz, $CDCl_3$) δ 8.19 (dd, *J* = 8.6, 1.3 Hz, 1H), 8.16 (dd, *J* = 8.6, 1.3 Hz, 1H), 7.85 – 7.80 (m, 1H), 7.75 – 7.70 (m, 1H), 7.60 (s, 1H), 3.44 – 3.32 (m, 1H), 2.08 – 1.93 (m, 4H), 1.93 – 1.84 (m, 1H), 1.65 – 1.52 (m, 4H), 1.44 – 1.33 (m, 1H). **$^{13}$C NMR** (125 MHz, $CDCl_3$) δ 155.7, 148.4, 133.8, 131.0, 130.5, 129.0, 127.5, 123.1, 120.0, 118.0, 39.1, 33.5, 26.7, 26.1. **GC-MS** (EI, m/z) for $C_{16}H_{15}N_2$ Calcd: 236.1, found: 236.1. Spectra data are consistent with the reported literature.[12]

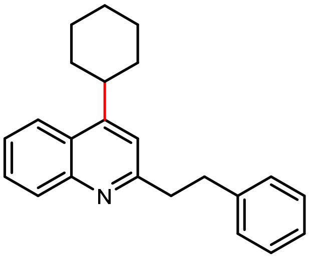

**4-cyclohexyl-2-phenethylquinoline (46)**. Following the general procedure A, the product was isolated by preparative TLC with Hex/EtOAc (7:1) as the eluent to give a yellow oil (28.4 mg, 45%). **$^{1}$H NMR** (500 MHz, $CDCl_3$) δ 8.11 (d, *J* = 8.5 Hz, 1H), 8.07 (d, *J* = 6.9 Hz, 1H), 7.72 – 7.66 (m, 1H), 7.56 – 7.49 (m, 1H), 7.33 – 7.20 (m, 6H), 3.34 – 3.24 (m, 3H), 3.21 – 3.12 (m, 2H), 2.01 – 1.90 (m, 4H), 1.90 – 1.83 (m, 1H), 1.63 – 1.42 (m, 4H), 1.41 – 1.30 (m, 1H). **$^{13}$C NMR** (125 MHz, $CDCl_3$) δ 161.6, 153.3, 148.3, 141.7, 129.8, 128.7, 128.6, 128.4, 126.0, 125.5, 125.4, 122.9, 118.0, 41.3, 38.8, 36.1, 33.5, 26.9, 26.3. **HRMS** ($M+H^+$) for $C_{23}H_{26}N$ Calcd: 316.2060, found: 316.2057. The compound was not reported.

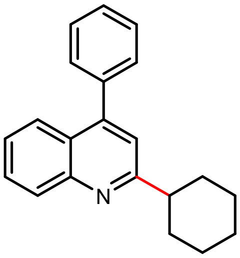

**2-cyclohexyl-4-phenylquinoline (47)**. Following the general procedure A, the product was isolated by preparative TLC with Hex/EtOAc (5:1) as the eluent to give a colorless oil (35.6 mg, 62%). **$^{1}$H NMR** (500 MHz, $CDCl_3$) δ 8.14 (d, *J* = 7.0 Hz, 1H), 7.89 (dd, *J* = 8.4, 1.4 Hz, 1H), 7.73 – 7.68 (m, 1H), 7.57 – 7.49 (m, 5H), 7.47 – 7.43 (m, 1H), 7.29 (s, 1H), 2.99 (tt, *J* = 12.0, 3.4 Hz, 1H), 2.14 – 2.06 (m, 2H), 1.96 – 1.89 (m, 2H), 1.86 – 1.78 (m, 1H), 1.75 – 1.63 (m, 2H), 1.56 – 1.45 (m, 2H), 1.41 – 1.30 (m, 1H). **$^{13}$C NMR** (125 MHz, $CDCl_3$) δ 166.4, 148.6, 148.3, 138.6, 129.6, 129.4, 129.1, 128.5, 128.3, 125.7, 125.6, 125.5, 119.9, 47.7, 32.9, 26.6, 26.1. **GC-MS** (EI, m/z) for $C_{21}H_{21}N$ Calcd: 287.2, found: 287.1. Spectra data are consistent with the reported literature.[13]

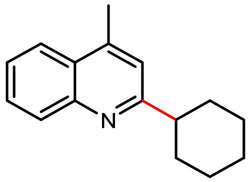

**2-cyclohexyl-4-methylquinoline (48)**. Following the general procedure A, the product was isolated by preparative TLC with Hex/EtOAc (10:1) as the eluent to give a colorless oil (32.0 mg, 71%). **$^{1}$H NMR** (500 MHz, $CDCl_3$) δ 8.07 (dd, *J* = 8.4, 1.3 Hz, 1H), 7.96 (dd, *J* = 8.3, 1.4 Hz, 1H), 7.71 – 7.65 (m, 1H), 7.54 – 7.47 (m, 1H), 7.19 (s, 1H), 2.90 (tt, *J* = 12.1, 3.4 Hz, 1H), 2.70 (s, 3H), 2.07 – 2.00 (m, 2H), 1.95 – 1.87 (m, 2H), 1.85 – 1.78 (m, 1H), 1.70 – 1.60 (m, 2H), 1.55 – 1.43 (m, 2H), 1.43 – 1.31 (m, 1H). **$^{13}$C NMR** (125 MHz, $CDCl_3$) δ 166.5, 147.7, 144.2, 129.5, 128.9, 127.1, 125.4, 123.6, 120.3, 47.6, 32.9, 26.6, 26.2, 18.9. **GC-MS** (EI, m/z) for $C_{16}H_{19}N$ Calcd: 225.2, found: 225.2. Spectra data are consistent with the reported literature.[14]

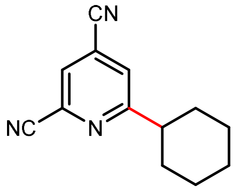


**6-cyclohexylpyridine-2,4-dicarbonitrile (49)**. Following the general procedure A, the product was isolated by preparative TLC with Hex/EtOAc (10:1) as the eluent to give a yellow oil (29.6 mg, 70%). **$^{1}$H NMR** (500 MHz, $CDCl_3$) δ 8.84 (s, 1H), 7.86 (d, *J* = 0.8 Hz, 1H), 3.04 (tt, *J* = 12.1, 3.1 Hz, 1H), 2.01 – 1.92 (m, 4H), 1.91 – 1.83 (m, 1H), 1.65 – 1.45 (m, 4H), 1.39 – 1.28 (m, 1H). **$^{13}$C NMR** (125 MHz, $CDCl_3$) δ 151.0, 148.6, 132.1, 129.9, 121.0, 115.8, 114.1, 41.8, 32.9, 26.2, 25.4. **HRMS** ($M+Na^+$) for $C_{13}H_{13}N_3Na$ Calcd: 234.1002, found: 234.0995. The compound was not reported.

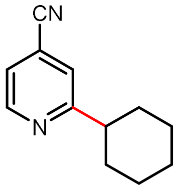


**2-cyclohexylisonicotinonitrile (50a)**. Following the general procedure A, the product was isolated by preparative TLC with Hex/EtOAc (10:1) as the eluent to give a yellow oil (8.9 mg, 24%). **$^{1}$H NMR** (500 MHz, $CDCl_3$) δ 8.72 (d, *J* = 5.0 Hz, 1H), 7.40 (s, 1H), 7.35 (dd, *J* = 4.9, 1.5 Hz, 1H), 2.78 (tt, *J* = 11.9, 3.4 Hz, 1H), 2.01 – 1.94 (m, 2H), 1.93 – 1.83 (m, 2H), 1.83 – 1.74 (m, 1H), 1.60 – 1.37 (m, 4H), 1.36 – 1.24 (m, 2H). **$^{13}$C NMR** (125 MHz, $CDCl_3$) δ 168.2, 150.1, 122.9, 122.4, 120.6, 116.9, 46.4, 32.6, 26.3, 25.9. **GC-MS** (EI, m/z) for $C_{12}H_{14}N_2$ Calcd: 186.1, found: 186.1. Spectra data are consistent with the reported literature.[15]

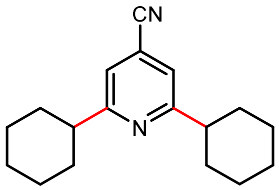

**2,6-dicyclohexylisonicotinonitrile (50b)**. Following the general procedure A, the product was isolated by preparative TLC with Hex/EtOAc (10:1) as the eluent to give a yellow oil (9.1 mg, 17%). **$^{1}$H NMR** (500 MHz, $CDCl_3$) δ 7.18 (s, 1H), 2.74 (tt, *J* = 11.7, 3.4 Hz, 1H), 2.01 – 1.93 (m, 2H), 1.92 – 1.84 (m, 2H), 1.82 – 1.75 (m, 1H), 1.56 – 1.38 (m, 4H), 1.36 – 1.26 (m, 1H). **$^{13}$C NMR** (125 MHz, $CDCl_3$) δ 167.2, 120.6, 119.6, 117.6, 46.4, 32.7, 26.4, 26.0. **GC-MS** (EI, m/z) for $C_{18}H_{24}N_2$ Calcd: 268.2, found: 268.1. Spectra data are consistent with the reported literature.[12]

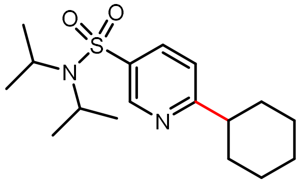

**6-cyclohexyl-*N*,*N*-diisopropylpyridine-3-sulfonamide (51)**. Following the general procedure A, the reaction was run for 36 h, and the product was isolated by preparative TLC with Hex/EtOAc (2:1) as the eluent to give a white solid (21.4 mg, 33%). **$^{1}$H NMR** (500 MHz, $CDCl_3$) δ 8.99 (d, *J* = 1.5 Hz, 1H), 8.05 (dd, *J* = 8.3, 2.4 Hz, 1H), 7.27 (d, *J* = 8.3 Hz, 1H), 3.74 (p, *J* = 6.8 Hz, 2H), 2.79 (tt, *J* = 11.9, 3.4 Hz, 1H), 2.00 – 1.93 (m, 2H), 1.92 – 1.85 (m, 2H), 1.82 – 1.75 (m, 1H), 1.60 – 1.49 (m, 2H), 1.49 – 1.25 (m, 15H). **$^{13}$C NMR** (125 MHz, $CDCl_3$) δ 170.2, 147.6, 136.4, 135.1, 120.8, 48.8, 46.6, 32.7, 26.4, 25.9, 22.0. **HRMS** ($M+Na^+$) for $C_{17}H_{28}N_2NaO_2S$ Calcd: 347.1764, found: 347.1755. The compound was not reported.

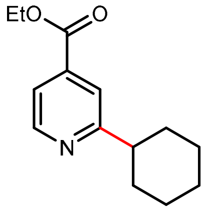

**ethyl 2-cyclohexylisonicotinate (52)**. Following the general procedure A, the product was isolated by preparative TLC with Hex/EtOAc (5:1) as the eluent to give a colorless oil (21.9 mg, 47%). **$^{1}$H NMR** (500 MHz, $CDCl_3$) δ 8.68 (d, *J* = 5.0 Hz, 1H), 7.73 (s, 1H), 7.66 (dd, *J* = 5.1, 1.6 Hz, 1H), 4.43 (q, *J* = 7.1 Hz, 2H), 2.81 (tt, *J* = 12.0, 3.4 Hz, 1H), 2.03 – 1.93 (m, 2H), 1.93 – 1.82 (m, 2H), 1.82 – 1.73 (m, 1H), 1.62 – 1.53 (m, 2H), 1.49 – 1.39 (m, 5H), 1.37 – 1.27 (m, 1H). **$^{13}$C NMR** (125 MHz, $CDCl_3$) δ 167.7, 165.6, 149.8, 138.0, 120.3, 120.2, 61.7, 46.6, 32.8, 26.5, 26.0, 14.2. **GC-MS** (EI, m/z) for $C_{14}H_{19}NO_2$ Calcd: 233.1, found: 233.1. Spectra data are consistent with the reported literature.[8]

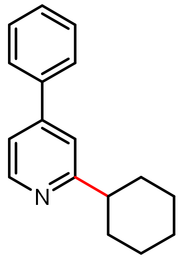


**2-cyclohexyl-4-phenylpyridine (53).** Following the general procedure A, the reaction was run at 55-60 °C for 36 h, and the product was isolated by preparative TLC with Hex/EtOAc (5:2) as the eluent to give a colorless oil (24.7 mg, 52%). **$^1$H NMR** (500 MHz, $CDCl_3$) δ 8.60 (dd, $J$ = 5.2, 0.8 Hz, 1H), 7.68 – 7.63 (m, 2H), 7.53 – 7.48 (m, 2H), 7.48 – 7.43 (m, 1H), 7.48 – 7.43 (m, 1H), 7.40 – 7.38 (m, 1H), 2.80 (tt, $J$ = 12.0, 3.4 Hz, 1H), 2.06 – 1.99 (m, 2H), 1.94 – 1.88 (m, 2H), 1.83 – 1.76 (m, 1H), 1.66 – 1.57 (m, 2H), 1.52 – 1.41 (m, 2H), 1.39 – 1.28 (m, 1H). **$^{13}$C NMR** (125 MHz, $CDCl_3$) δ 167.0, 149.5, 148.9, 138.8, 129.0, 128.8, 127.1, 119.2, 119.1, 46.7, 33.0, 26.6, 26.1. **GC-MS** (EI, m/z) for $C_{17}H_{19}N$ Calcd: 237.2, found: 237.1. Spectra data are consistent with the reported literature.[12]

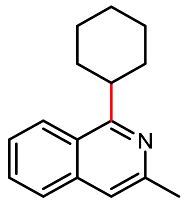


**1-cyclohexyl-3-methylisoquinoline (54)**. Following the general procedure A, the reaction was run for 36 h, and the product was isolated by preparative TLC with Hex/EtOAc (10:1) as the eluent to give a colorless oil (29.7 mg, 66%). **$^1$H NMR** (500 MHz, $CDCl_3$) δ 8.19 (d, $J$ = 8.4 Hz, 1H), 7.73 (d, $J$ = 7.8 Hz, 1H), 7.63 – 7.57 (m, 1H), 7.54 – 7.47 (m, 1H), 7.32 (s, 1H), 3.55 (tt, $J$ = 11.5, 3.3 Hz, 1H), 2.68 (s, 3H), 2.01 – 1.77 (m, 7H), 1.62 – 1.50 (m, 2H), 1.48 – 1.37 (m, 1H). **$^{13}$C NMR** (125 MHz, $CDCl_3$) δ 165.0, 150.5, 137.1, 129.3, 126.9, 125.6, 124.7, 124.3, 116.6, 41.6, 32.5, 26.9, 26.2, 24.5. **GC-MS** (EI, m/z) for $C_{16}H_{19}N$ Calcd: 225.2, found: 225.1. Spectra data are consistent with the reported literature.[16]

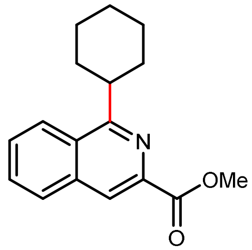


**methyl 1-cyclohexylisoquinoline-3-carboxylate (55)**. Following the general procedure A, the reaction was run for 36 h, and the product was isolated by preparative TLC with Hex/EtOAc (5:1) as the eluent to give a white solid (27.5 mg, 51%). **$^1$H NMR** (500 MHz, $CDCl_3$) δ 8.42 (s, 1H), 8.32 – 8.28 (m, 1H), 7.99 – 7.94 (m, 1H), 7.78 – 7.69 (m, 2H), 4.05 (s, 3H), 3.59 (tt, $J$ = 11.1, 3.8 Hz, 1H), 2.06 – 1.92 (m, 6H), 1.86 – 1.79 (m, 1H), 1.63 – 1.51 (m, 2H), 1.50 – 1.39 (m, 1H). **$^{13}$C NMR** (125 MHz, $CDCl_3$) δ 166.9, 166.1, 140.7, 136.0, 130.1, 129.1, 129.0, 127.8, 125.0, 122.4, 52.7, 42.1, 32.2, 26.8, 26.1. **GC-MS** (EI, m/z) for $C_{17}H_{19}NO_2$ Calcd: 269.1, found: 269.1. Spectra data are consistent with the reported literature.[12]

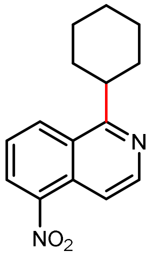


**1-cyclohexyl-5-nitroisoquinoline (56)**. Following the general procedure A, the reaction was run at 55-60 °C for 36 h, and the product was isolated by preparative TLC with Hex/EtOAc (1:1) as the eluent to give a yellow oil (15.4 mg, 30%). **$^1$H NMR** (500 MHz, $CDCl_3$) δ 8.71 (d, $J$ = 6.1 Hz, 1H), 8.60 (dd, $J$ = 8.5, 0.8 Hz, 1H), 8.45 (dd, $J$ = 7.7, 1.1 Hz, 1H), 8.24 (dd, $J$ = 6.1, 1.0 Hz, 1H), 7.74 – 7.68 (m, 1H), 3.60 (tt, $J$ = 11.6, 3.2 Hz, 1H), 2.04 – 1.94 (m, 4H), 1.93 – 1.81 (m, 3H), 1.60 – 1.50 (m, 2H), 1.47 – 1.36 (m, 1H). **$^{13}$C NMR** (125 MHz, $CDCl_3$) δ 166.5, 146.1, 145.3, 131.4, 128.9, 127.2, 126.9, 125.1, 113.4, 42.2, 32.7, 26.8, 26.1. **GC-MS** (EI, m/z) for $C_{15}H_{16}N_2O_2$ Calcd: 256.1, found: 256.1. Spectra data are consistent with the reported literature.[15]

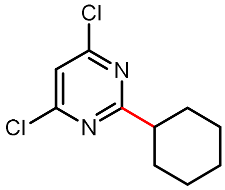


**4,6-dichloro-2-cyclohexylpyrimidine (57)**. Following the general procedure A, the reaction was run with 4 equiv of TFA for 36 h, and the product was isolated by preparative TLC with Hex/EtOAc (10:1) as the eluent to give a colorless oil (21.7 mg, 47%). **$^1$H NMR** (500 MHz, $CDCl_3$) δ 7.24 (s, 1H), 2.87 (tt, $J$ = 11.8, 3.5 Hz, 1H), 2.03 – 1.98 (m, 2H), 1.89 – 1.85 (m, 2H), 1.78 – 1.73 (m, 1H), 1.68 – 1.57 (m, 2H), 1.46 – 1.25 (m, 3H). **$^{13}$C NMR** (125 MHz, $CDCl_3$) δ 176.5, 161.7, 118.5, 47.2, 31.5, 25.9, 25.7. **GC-MS** (EI, m/z) for $C_{10}H_{12}Cl_2N_2$ Calcd: 230.0, 232.0, found: 230.0, 232.0. Spectra data are consistent with the reported literature.[8]

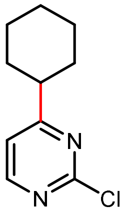


**2-chloro-4-cyclohexylpyrimidine (58a)**. Following the general procedure A, the reaction was run with 4 equiv of TFA for 36 h, the product was isolated by preparative TLC with Hex/EtOAc (10:1) as the eluent to give a colorless oil (20.8 mg, 53%). **$^1$H NMR** (500 MHz, $CDCl_3$) δ 8.51 (d, $J$ = 5.1 Hz, 1H), 7.13 (d, $J$ = 5.1 Hz, 1H), 2.69 (tt, $J$ = 11.9, 3.4 Hz, 1H), 2.01 – 1.94 (m, 2H), 1.93 – 1.85 (m, 2H), 1.81 – 1.74 (m, 1H), 1.57 – 1.46 (m, 2H), 1.46 – 1.35 (m, 2H), 1.34 – 1.23 (m, 1H). **$^{13}$C NMR** (125 MHz, $CDCl_3$) δ 178.6, 161.2, 159.3, 117.0, 45.9, 31.9, 26.0, 25.7. **GC-MS** (EI, m/z) for $C_{10}H_{13}ClN_2$ Calcd: 196.1, 198.1, found: 196.1, 198.1. Spectra data are consistent with the reported literature.[17]

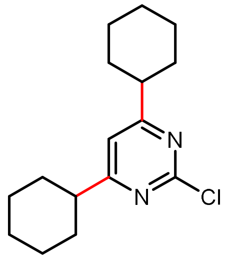

**2-chloro-4,6-dicyclohexylpyrimidine (58b)**. Following the general procedure A, the reaction was run with 4 equiv of TFA for 36 h, and the product was isolated by preparative TLC with Hex/EtOAc (10:1) as the eluent to give a colorless oil (5 mg, 9%). **$^{1}$H NMR** (500 MHz, $CDCl_3$) δ 6.94 (s, 1H), 2.70 – 2.60 (m, 2H), 2.00 – 1.92 (m, 4H), 1.92 – 1.83 (m, 4H), 1.80 – 1.73 (m, 2H), 1.56 – 1.46 (m, 4H), 1.46 – 1.35 (m, 4H), 1.35 – 1.24 (m, 2H). **$^{13}$C NMR** (125 MHz, $CDCl_3$) δ 178.2, 160.7, 114.0, 45.9, 32.0, 26.1, 25.7. **GC-MS** (EI, m/z) for $C_{16}H_{23}ClN_2$ Calcd: 278.2, 280.2, found: 278.1, 280.1. Spectra data are consistent with the reported literature.[12]

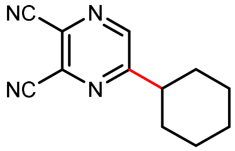

**5-cyclohexylpyrazine-2,3-dicarbonitrile (59)**. Following the general procedure A, the reaction was run with 4 equiv of TFA for 36 h, and the product was isolated by preparative TLC with Hex/EtOAc (1:1) as the eluent to give a brown oil (24.2 mg, 57%). **$^{1}$H NMR** (500 MHz, $CDCl_3$) δ 8.76 (s, 1H), 2.94 (tt, *J* = 11.9, 3.3 Hz, 1H), 2.01 – 1.90 (m, 4H), 1.86 – 1.80 (m, 1H), 1.68 – 1.58 (m, 2H), 1.52 – 1.40 (m, 2H), 1.39 – 1.31 (m, 1H). **$^{13}$C NMR** (125 MHz, $CDCl_3$) δ 165.7, 146.4, 133.1, 131.0, 113.1, 113.1, 44.3, 31.9, 25.9, 25.4. **HRMS** ($M+Na^+$) for $C_{12}H_{12}N_4Na$ Calcd: 235.0954, found: 235.0943. The compound was not reported.

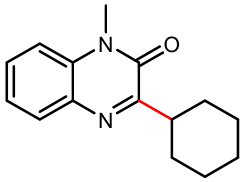

**3-cyclohexyl-1-methylquinoxalin-2(1*H*)-one (60)**. Following the general procedure A, the product was isolated by preparative TLC with Hex/EtOAc (5:1) as the eluent to give a white solid (32.5 mg, 67%). **$^{1}$H NMR** (500 MHz, $CDCl_3$) δ 7.86 (dd, *J* = 8.1, 1.5 Hz, 1H), 7.56 – 7.50 (m, 1H), 7.37 – 7.32 (m, 1H), 7.32 – 7.29 (m, 1H), 3.72 (s, 3H), 3.36 (tt, *J* = 11.6, 3.3 Hz, 1H), 2.02 – 1.94 (m, 2H), 1.92 – 1.86 (m, 2H), 1.82 – 1.76 (m, 1H), 1.65 – 1.55 (m, 2H), 1.55 – 1.43 (m, 2H), 1.39 – 1.27 (m, 1H). **$^{13}$C NMR** (125 MHz, $CDCl_3$) δ 164.3, 154.6, 132.9, 132.9, 129.8, 129.4, 123.4, 113.5, 40.8, 30.5, 29.1, 26.3, 26.2. **GC-MS** (EI, m/z) for $C_{15}H_{18}N_2O$ Calcd: 242.1, found: 242.1. Spectra data are consistent with the reported literature.[18]

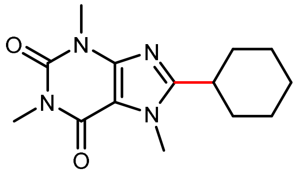

**8-cyclohexyl-1,3,7-trimethyl-3,7-dihydro-1*H*-purine-2,6-dione (61)**. Following the general procedure A, the reaction was run with 4 equiv of TFA, and the product was isolated by preparative TLC with Hex/EtOAc (1:1) as the eluent to give a white solid (35.9 mg, 65%). **$^1$H NMR** (500 MHz, $CDCl_3$) δ 3.94 (s, 3H), 3.58 (s, 3H), 3.40 (s, 3H), 2.72 (tt, *J* = 11.6, 3.5 Hz, 1H), 1.93 – 1.83 (m, 4H), 1.80 – 1.66 (m, 3H), 1.45 – 1.29 (m, 3H). **$^{13}$C NMR** (125 MHz, $CDCl_3$) δ 158.0, 155.5, 151.8, 148.1, 107.0, 35.8, 31.4, 30.9, 29.7, 27.8, 26.0, 25.6. **GC-MS** (EI, m/z) for $C_{14}H_{20}$N4O2 Calcd: 276.2, found: 276.1. Spectra data are consistent with the reported literature.[19]

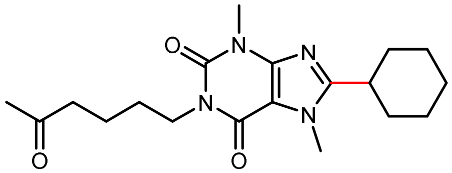

**8-cyclohexyl-7-methyl-1-(4-oxopentyl)-3,7-dihydro-1*H*-purine-2,6-dione (62)**. Following the general procedure A, the reaction was run with 4 equiv of TFA, and the product was isolated by preparative TLC with Hex/EtOAc (1:1) as the eluent to give a white solid (38.2 mg, 53%). **$^1$H NMR** (500 MHz, $CDCl_3$) δ 4.01 (t, *J* = 7.0 Hz, 2H), 3.93 (s, 3H), 3.57 (s, 3H), 2.72 (tt, *J* = 11.6, 3.4 Hz, 1H), 2.51 (t, *J* = 7.1 Hz, 2H), 2.15 (s, 3H), 1.94 – 1.84 (m, 4H), 1.81 – 1.61 (m, 6H), 1.45 – 1.32 (m, 3H). **$^{13}$C NMR** (125 MHz, $CDCl_3$) δ 208.8, 158.0, 155.3, 151.5, 148.2, 107.0, 43.3, 40.6, 35.8, 31.4, 30.9, 29.9, 29.7, 27.5, 26.0, 25.6, 21.0. **HRMS** ($M+Na^+$) for $C_{19}H_{28}N_4NaO_3$ Calcd: 383.2054, found: 383.2042. The compound was not reported.

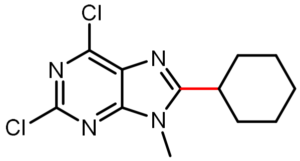

**2,6-dichloro-8-cyclohexyl-9-methyl-9*H*-purine (63)**. Following the general procedure A, the reaction was run with 5 equiv of TFA, and the product was isolated by preparative TLC with Hex/EtOAc (1:1) as the eluent to give a white solid (39.9 mg, 70%). **$^1$H NMR** (500 MHz, $CDCl_3$) δ 3.83 (s, 3H), 2.90 (tt, *J* = 11.8, 3.5 Hz, 1H), 2.05 – 1.91 (m, 4H), 1.90 – 1.76 (m, 3H), 1.51 – 1.34 (m, 3H). **$^{13}$C NMR** (125 MHz, $CDCl_3$) δ 163.1, 154.7, 151.7, 149.5, 130.2, 36.9, 30.8, 29.2, 25.9, 25.5. **GC-MS** (EI, m/z) for $C_{12}H_{14}Cl_2N_4$ Calcd: 284.1, 286.1, found: 284.0, 286.0. Spectra data are consistent with the reported literature.[13]

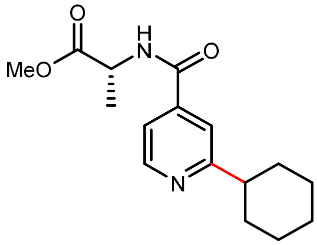

**(*R*)-methyl-2-(2-cyclohexylisonicotinamido)propanoate (64a)**. Following the general procedure A, the reaction was run for 36 h, and the product was isolated by preparative TLC with Hex/EtOAc (1:1) as the eluent to give a white oil (20.3 mg, 35%). **$^1$H NMR** (500 MHz, $CDCl_3$) δ 8.66 (d, *J* = 5.8 Hz, 1H), 7.52 (s, 1H), 7.42 (dd, *J* = 5.1, 1.7 Hz, 1H), 6.88 (d, *J* = 9.5 Hz, 1H), 4.81 (p, *J* = 7.2 Hz, 1H), 3.82 (s, 3H), 2.78 (tt, *J* = 12.0, 3.5 Hz, 1H), 2.00 – 1.93 (m, 2H), 1.92 – 1.84 (m, 2H), 1.81 – 1.74 (m, 1H), 1.62 – 1.51 (m, 5H), 1.49 – 1.38 (m, 2H), 1.36 – 1.24 (m, 1H). **$^{13}$C NMR** (125 MHz, $CDCl_3$) δ 173.4, 167.9, 165.4, 149.8, 141.4,

118.5, 118.1, 52.7, 48.6, 46.6, 32.8, 26.5, 26.0, 18.5. **HRMS** (M+Na$^+$) for $C_{16}H_{22}N_2NaO_3$ Calcd: 313.1523, found: 313.1514. The compound was not reported.

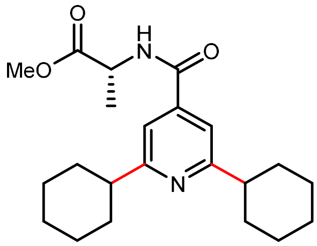


**(*R*)-methyl-2-(2,6-dicyclohexylisonicotinamido)propanoate (64b)**. Following the general procedure A, the reaction was run for 36 h, and the product was isolated by preparative TLC with Hex/EtOAc (5:2) as the eluent to give a white solid (6.7 mg, 9%). **$^1$H NMR** (500 MHz, $CDCl_3$) δ 7.28 (s, 2H), 6.80 (d, *J* = 6.8 Hz, 1H), 4.81 (p, *J* = 7.2 Hz, 1H), 3.82 (s, 3H), 2.75 (tt, *J* = 11.8, 3.4 Hz, 2H), 1.99 – 1.94 (m, 4H), 1.89 – 1.84 (m, 4H), 1.79 – 1.73 (m, 2H), 1.59 – 1.48 (m, 7H), 1.48 – 1.37 (m, 4H), 1.36 – 1.23 (m, 2H). **$^{13}$C NMR** (125 MHz, $CDCl_3$) δ 173.5, 166.9, 166.2, 141.8, 115.1, 52.7, 48.5, 46.7, 32.9, 26.5, 26.1, 18.6. **GC-MS** (EI, m/z) for $C_{22}H_{32}N_2O_3$ Calcd: 372.2, found: 372.2. Spectra data are consistent with the reported literature.[8]

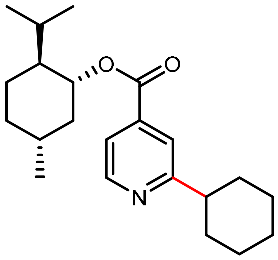


**(1*R*,2*S*,5*R*)-2-isopropyl-5-methylcyclohexyl 2-cyclohexylisonicotinate (65)**. Following the general procedure A, the reaction was run for 36 h, and the product was isolated by preparative TLC with Hex/EtOAc (10:1) as the eluent to give a colorless oil (28.2 mg, 41%). **$^1$H NMR** (500 MHz, $CDCl_3$) δ 8.68 (dd, *J* = 5.0, 0.9 Hz, 1H), 7.72 (s, 1H), 7.66 (dd, *J* = 5.1, 1.6 Hz, 1H), 4.98 (td, *J* = 10.9, 4.4 Hz, 1H), 2.81 (tt, *J* = 11.9, 3.4 Hz, 1H), 2.15 – 2.09 (m, 1H), 2.01 – 1.86 (m, 5H), 1.82 – 1.72 (m, 3H), 1.64 – 1.53 (m, 4H), 1.51 – 1.39 (m, 2H), 1.37 – 1.27 (m, 1H), 1.21 – 1.10 (m, 2H), 1.00 – 0.90 (m, 7H), 0.82 (d, *J* = 6.9 Hz, 3H). **$^{13}$C NMR** (125 MHz, $CDCl_3$) δ 167.6, 165.1, 149.8, 138.4, 120.4, 120.2, 75.8, 47.2, 46.6, 40.8, 34.2, 32.8, 32.8, 31.5, 26.6, 26.5, 26.0, 23.6, 22.0, 20.7, 16.5. **HRMS** (M+H$^+$) for $C_{22}H_{34}NO_2$ Calcd: 344.2584, found: 344.2571. The compound was not reported.

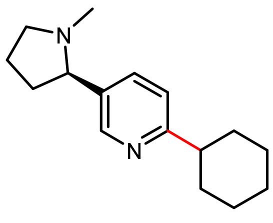


**(*R*)-2-cyclohexyl-5-(1-methylpyrrolidin-2-yl)pyridine (66)**. Following the general procedure A, the reaction was run with 4 equiv of TFA, and the product was isolated by preparative TLC with Hex/EtOAc/MeOH (2:1:0.5) as the eluent to give a yellow oil (20.5 mg, 42%). **$^1$H NMR** (500 MHz, $CDCl_3$) δ 8.43 (d, *J* = 1.8 Hz, 1H), 7.64 (dd, *J* = 8.1, 2.3 Hz, 1H), 7.14 (d, *J* = 8.0 Hz, 1H), 3.26 (t, *J* = 9.6 Hz, 1H),

3.07 (t, $J$ = 8.3 Hz, 1H), 2.71 (tt, $J$ = 11.9, 3.4 Hz, 1H), 2.34 – 2.26 (m, 1H), 2.23 – 2.14 (m, 4H), 2.02 – 1.93 (m, 2H), 1.90 – 1.71 (m, 5H), 1.58 – 1.24 (m, 6H). **$^{13}$C NMR** (125 MHz, $CDCl_3$) δ 165.5, 148.8, 135.2, 120.9, 68.7, 57.1, 46.3, 40.4, 35.0, 33.0, 33.0, 26.6, 26.1, 22.6. **GC-MS** (EI, m/z) for $C_{16}H_{24}N_2$ Calcd: 244.2, found: 244.1. Spectra data are consistent with the reported literature.[8]

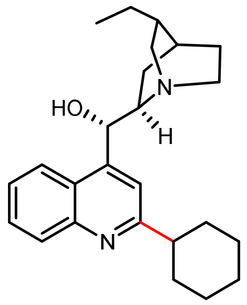


**(*S*)-(2-cyclohexylquinolin-4-yl)((1*S*,2*S*,4*S*,5*R*)-5-ethylquinuclidin-2-yl)methanol (67)**. Following the general procedure A, the reaction was run at 55-60 °C, and the product was isolated by preparative TLC with Hex/EtOAc/MeOH (1:1:1) as the eluent to give a white solid (40.1 mg, 53%). **$^1$H NMR** (500 MHz, $CDCl_3$) δ 8.05 (d, $J$ = 7.0 Hz, 1H), 7.77 (d, $J$ = 9.8 Hz, 1H), 7.65 – 7.57 (m, 2H), 7.35 – 7.30 (m, 1H), 6.06 (s, 1H), 3.62 (d, $J$ = 8.6 Hz, 1H), 3.24 – 3.06 (m, 3H), 3.00 – 2.84 (m, 2H), 2.18 – 2.05 (m, 1H), 2.02 – 1.94 (m, 2H), 1.93 – 1.85 (m, 2H), 1.85 – 1.75 (m, 2H), 1.70 – 1.25 (m, 11H), 1.04 – 0.96 (m, 1H), 0.92 (t, $J$ = 7.2 Hz, 3H). **$^{13}$C NMR** (125 MHz, $CDCl_3$) δ 166.6, 147.8, 147.0, 129.9, 128.9, 126.0, 123.8, 122.1, 117.0, 68.9, 60.3, 50.8, 49.7, 47.7, 36.3, 32.8, 32.7, 26.5, 26.0, 25.8, 25.4, 24.7, 19.1, 11.8. **HRMS** ($M+H^+$) for $C_{25}H_{35}N_2O$ Calcd: 379.2744, found: 379.2731. The compound was not reported.

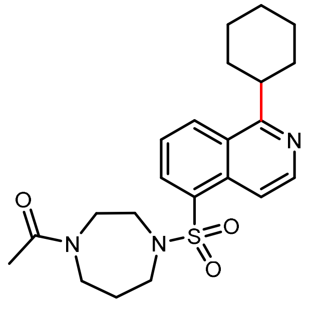


**1-(4-((1-cyclohexylisoquinolin-5-yl)sulfonyl)-1,4-diazepan-1-yl)ethan-1-one, undefined ratio of conformational isomers (68)**. Following the general procedure A, the reaction was run with 4 equiv of TFA at 55-60 °C for 36 h, and the product was isolated by preparative TLC with Hex/EtOAc/MeOH (1:1:0.5) as the eluent to give a white solid (29.1 mg, 35%). **$^1$H NMR** (500 MHz, $CDCl_3$) δ 8.66 – 8.62 (m, 1H), 8.54 – 8.48 (m, 1H), 8.35 – 8.28 (m, 1H), 8.23 (d, $J$ = 6.0 Hz, 1H), 7.70 – 7.64 (m, 1H), 3.76 – 3.76 (m, 1H), 3.70 – 3.55 (m, 4H), 3.56 – 3.49 (m, 1H), 3.49 – 3.38 (m, 3H), 2.09 – 2.05 (m, 3H), 2.05 – 1.91 (m, 6H), 1.91 – 1.79 (m, 3H), 1.62 – 1.48 (m, 2H), 1.48 – 1.33 (m, 1H). **$^{13}$C NMR** (125 MHz, $CDCl_3$) δ 170.2, 170.0, 166.8, 166.8, 144.1, 144.0, 134.7, 132.4, 132.4, 132.2, 130.6, 130.6, 126.9, 125.2, 115.2, 115.2, 50.9, 50.1, 49.2, 48.3, 48.0, 47.6, 46.8, 44.4, 42.1, 42.1, 32.7, 28.9, 27.6, 26.8, 26.1, 21.6, 21.1. **GC-MS** (EI, m/z) for $C_{22}H_{29}N_3S_3$ Calcd: 415.2, found: 415.1. Spectra data are consistent with the reported literature.[20]

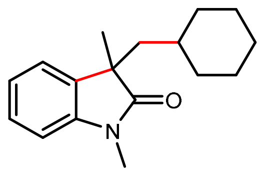

**3-(cyclohexylmethyl)-1,3-dimethylindolin-2-one (71)**. Colorless oil. **$^{1}$H NMR** (500 MHz, $CDCl_3$) δ 7.31 – 7.26 (m, 1H), 7.18 (d, *J* = 6.0 Hz, 1H), 7.10 – 7.05 (m, 1H), 6.87 (d, *J* = 7.7 Hz, 1H), 3.24 (s, 3H), 1.95 (dd, *J* = 14.1, 7.0 Hz, 1H), 1.75 (dd, *J* = 14.0, 5.2 Hz, 1H), 1.57 – 1.45 (m, 3H), 1.39 – 1.31 (m, 4H), 1.26 – 1.19 (m, 1H), 1.05 – 0.91 (m, 4H), 0.89 – 0.71 (m, 2H). **$^{13}$C NMR** (125 MHz, $CDCl_3$) δ 181.2, 143.1, 134.4, 127.5, 122.7, 122.3, 108.0, 47.9, 45.4, 34.7, 34.5, 33.5, 26.2, 26.2, 26.1, 26.0. **GC-MS** (EI, m/z) for $C_{17}H_{23}NO$ Calcd: 257.2, found: 257.1. Spectra data are consistent with the reported literature.[21]

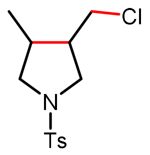

**3-(chloromethyl)-4-methyl-1-tosylpyrrolidine, 1:0.5 diastereomers (73)**. Yellow oil. **$^{1}$H NMR** (500 MHz, $CDCl_3$) δ 7.77 – 7.68 (m, 2H+1H), 7.40 – 7.30 (m, 2H+1H), 3.57 – 3.39 (m, 3H+1.5H), 3.34 (dd, *J* = 11.1, 7.5 Hz, 0.5H), 3.25 – 3.11 (m, 2H+0.5H), 3.04 (dd, *J* = 9.8, 4.9 Hz, 1H), 2.83 (dd, *J* = 9.8, 8.0 Hz, 0.5H), 2.48 – 2.28 (m, 3H+3.5H), 2.04 – 1.94 (m, 1H), 0.98 (d, *J* = 6.4 Hz, 1.5H), 0.86 (d, *J* = 6.9 Hz, 3H). **$^{13}$C NMR** (125 MHz, $CDCl_3$) δ 143.6, 143.5, 133.8, 133.4, 129.7, 127.6, 127.5, 54.8, 54.4, 51.3, 50.1, 47.8, 44.9, 44.4, 42.7, 36.4, 34.9, 21.6, 16.7, 12.8. **GC-MS** (EI, m/z) for $C_{22}H_{29}N_3S_3$ Calcd: 287.1, 289.1, found: 287.0, 289.0. Spectra data are consistent with the reported literature.[22]

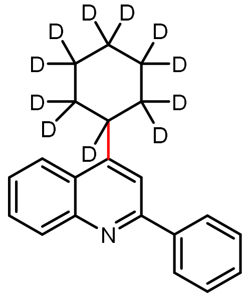

**4-(cyclohexyl-*d*$_{11}$)-2-phenylquinoline (3-*d*$_{11}$)**. White solid. **$^{1}$H NMR** (500 MHz, $CDCl_3$) δ 8.22 (dd, *J* = 8.4, 1.3 Hz, 1H), 8.17 – 8.15 (m, 2H), 8.13 – 8.09 (m, 1H), 7.78 (s, 1H), 7.75 – 7.70 (m, 1H), 7.59 – 7.52 (m, 3H), 7.51 – 7.46 (m, 1H). **$^{13}$C NMR** (125 MHz, $CDCl_3$) δ 157.4, 154.0, 148.6, 140.3, 130.7, 129.1, 129.0, 128.8, 127.6, 125.9, 125.9, 122.9, 115.5. **HRMS** ($M+H^+$) for $C_{12}H_{11}D_{11}N$ Calcd: 299.2437, found: 299.2426. The compound was not reported.

## 5. Supplementary References


1. McCallum, T. *et al.* The Photochemical Alkylation and Reduction of Heteroarenes. *Chem. Sci.* ***8***, 7412–7418 (2017).

2. Fu, N., Sauer, G. S. & Lin, S. Electrocatalytic Radical Dichlorination of Alkenes with Nucleophilic Chlorine Sources. *J. Am. Chem. Soc.* ***139***, 15548–15553 (2017).
3. Xu, P., Chen, P.-Y. & Xu, H.-C. Scalable Photoelectrochemical Dehydrogenative Cross-Coupling of Heteroarenes with Aliphatic C–H Bonds. *Angew. Chem. Int. Ed.* ***59***, 14275–14280 (2020).
4. Luo, G.-G. *et al.* Noble-Metal-Free BODIPY–Cobaloxime Photocatalysts for Visible-Light-Driven Hydrogen Production. *Phys. Chem. Chem. Phys.* ***16***, 23884–23894 (2014).
5. Cismesia, M. A. & Yoon, T. P. Characterizing Chain Processes in Visible Light Photoredox Catalysis. *Chem. Sci.* ***6***, 5426–5434 (2015).
6. Zhang, L. & Jiao, L. Visible-Light-Induced Organocatalytic Borylation of Aryl Chlorides. *J. Am. Chem. Soc.* ***141***, 9124–9128 (2019).
7. Wang, Z. *et al.* Bromide-Promoted Visible-Light-Induced Reductive Minisci Reaction with Aldehydes. *ACS Catal.* ***10***, 154–159 (2020).
8. Huang, C.-Y., Li, J., Liu, W. & Li, C.-J. Diacetyl as a "Traceless" Visible Light Photosensitizer in Metal-Free Cross-Dehydrogenative Coupling Reactions. *Chem. Sci.* ***10***, 5018–5024 (2019).
9. Xu, T. *et al.* Pd-Catalyzed Tandem Reaction of 2-Aminostyryl Nitriles with Arylboronic Acids: Synthesis of 2-Arylquinolines. *J. Org. Chem.* ***84***, 13604–13614 (2019).
10. Ramaraju, A. *et al.* Cu-Catalyzed Coupling of *O*-Acyl Oximes with Isatins: Domino Rearrangement Strategy for Direct Access to Quinoline-4-Carboxamides by C–N Bond Cleavage. *Eur. J. Org. Chem.* ***2018***, 2963–2971 (2018).
11. Ikarashi, G., Morofuji, T. & Kano, N. Terminal-Oxidant-Free Photocatalytic C–H Alkylations of Heteroarenes with Alkylsilicates as Alkyl Radical Precursors. *Chem. Commun.* ***56***, 10006–10009 (2020).
12. Shao, X., Wu, X., Wu, S. & Zhu, C. Metal-Free Radical-Mediated C($sp^3$)–H Heteroarylation of Alkanes. *Org. Lett.* ***22***, 7450–7454 (2020).
13. Zhao, H. & Jin, J. Visible Light-Promoted Aliphatic C–H Arylation Using Selectfluor as a Hydrogen Atom Transfer Reagent. *Org. Lett.* ***21***, 6179–6184 (2019).
14. Fu, M.-C. *et al.* Photocatalytic Decarboxylative Alkylations Mediated by Triphenylphosphine and Sodium Iodide. *Science* ***363***, 1429–1434 (2019).
15. Zhou, J. *et al.* Copper-Catalyzed Versatile C($sp^3$)–H Arylation: Synthetic Scope and Regioselectivity Investigations. *Org. Chem. Front.* ***6***, 1594–1598 (2019).
16. Sutherland, D. R., Veguillas, M., Oates, C. L. & Lee, A.-L. Metal-, Photocatalyst-, and Light-Free, Late-Stage C–H Alkylation of Heteroarenes and 1,4-Quinones Using Carboxylic Acids. *Org. Lett.* ***20***, 6863–6867 (2018).
17. Perry, I. B. *et al.* Direct Arylation of Strong Aliphatic C–H Bonds. *Nature* ***560***, 70–75 (2018).
18. He, X.-K. *et al.* Bi-OAc-Accelerated C3–H Alkylation of Quinoxalin-2(1*H*)-ones under Visible-Light Irradiation. *Org. Lett.* ***22***, 5984–5989 (2020).
19. Antonchick, A. P. & Burgmann, L. Direct Selective Oxidative Cross-Coupling of Simple Alkanes with Heteroarenes. *Angew. Chem. Int. Ed.* ***52***, 3267–3271 (2013).
20. Garza-Sanchez, R. A., Tlahuext-Aca, A., Tavakoli, G. & Glorius, F. Visible Light-Mediated Direct Decarboxylative C–H Functionalization of Heteroarenes. *ACS Catal.* *7*, 4057–4061 (2017).
21. Ling, A., Zhang, L., Tan, R. X. & Liu, Z.-Q. Molecular Oxygen-Promoted General and Site-Specific Alkylation with Organoboronic Acid. *J. Org. Chem.* ***83***, 14489–14497 (2018).
22. Taniguchi, T., Goto, N., Nishibata, A. & Ishibashi, H. Iron-Catalyzed Redox Radical Cyclizations of 1,6-Dienes and Enynes. *Org. Lett.* ***12***, 112–115 (2010).

# 6. NMR spectra

**Supplementary Figure 9a | $^{1}H$ NMR (500 MHz, $CDCl_3$) of 4-cyclohexyl-2-phenylquinoline (3)**

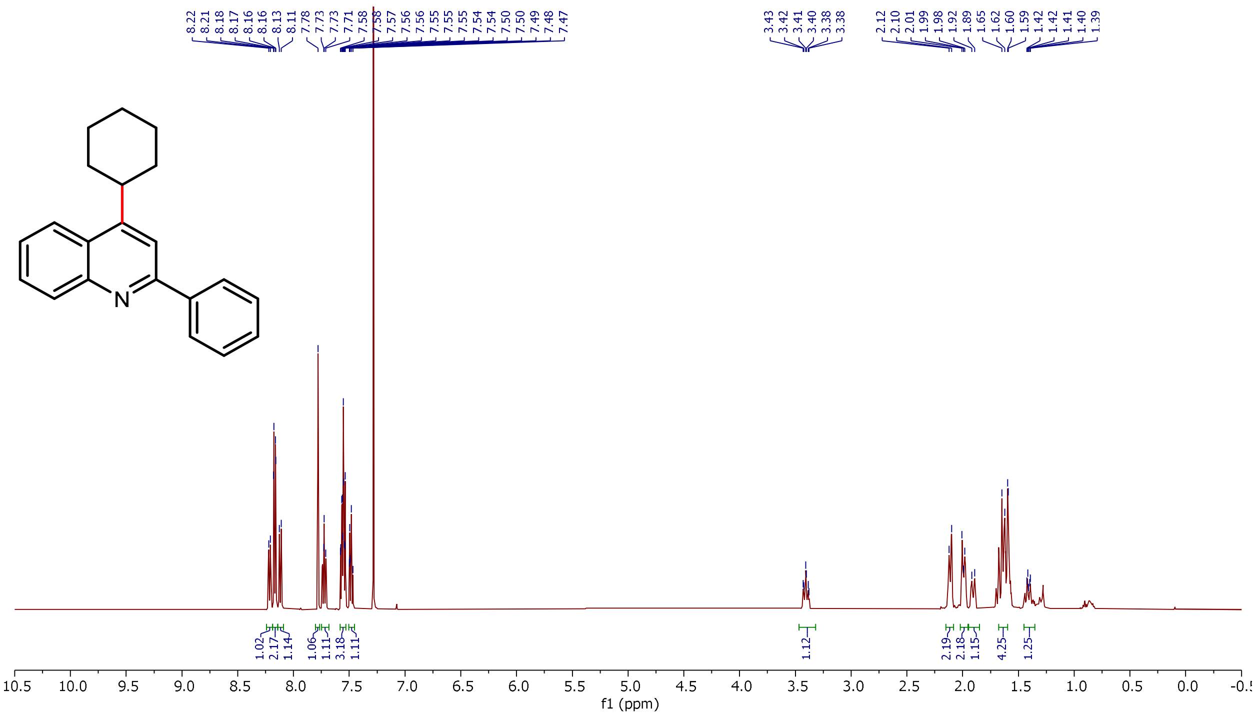

**Supplementary Figure 9b | $^{13}C$ NMR (125 MHz, $CDCl_3$) of 4-cyclohexyl-2-phenylquinoline (3)**

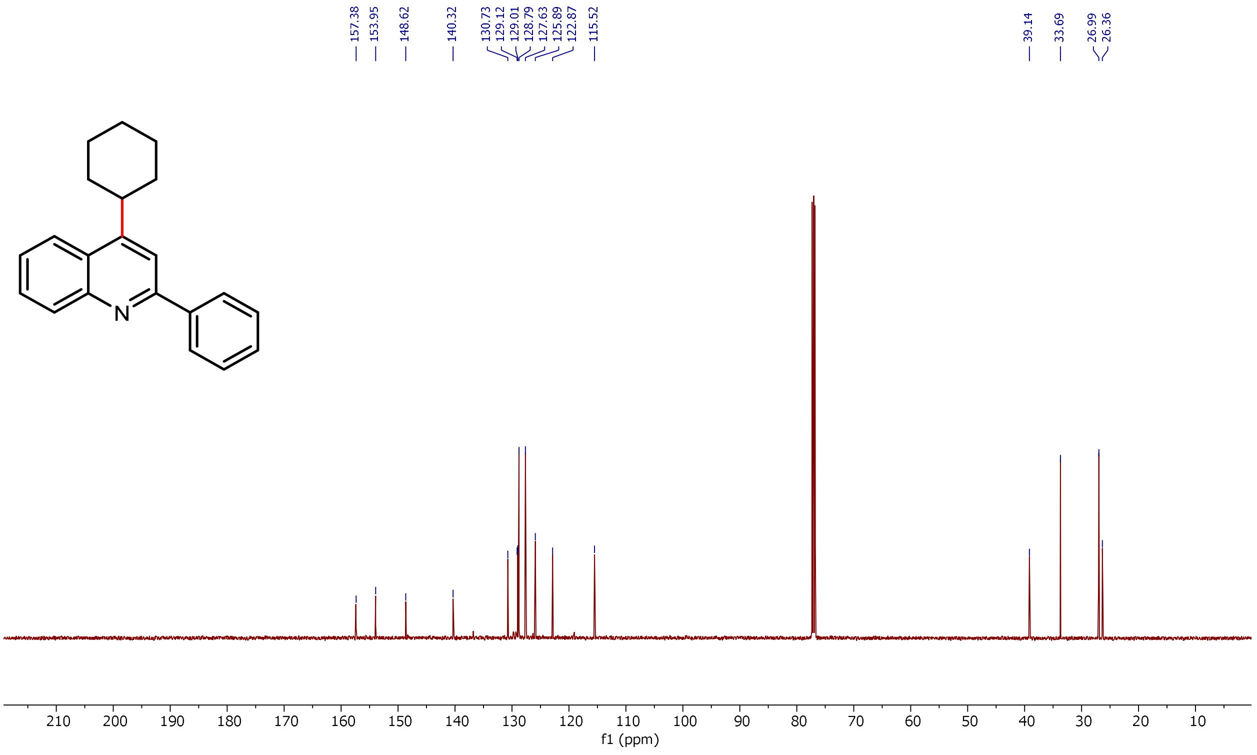

## Supplementary Figure 10a | $^{1}$H NMR (500 MHz, $CDCl_3$) of 4-cyclopentyl-2-phenylquinoline (4)

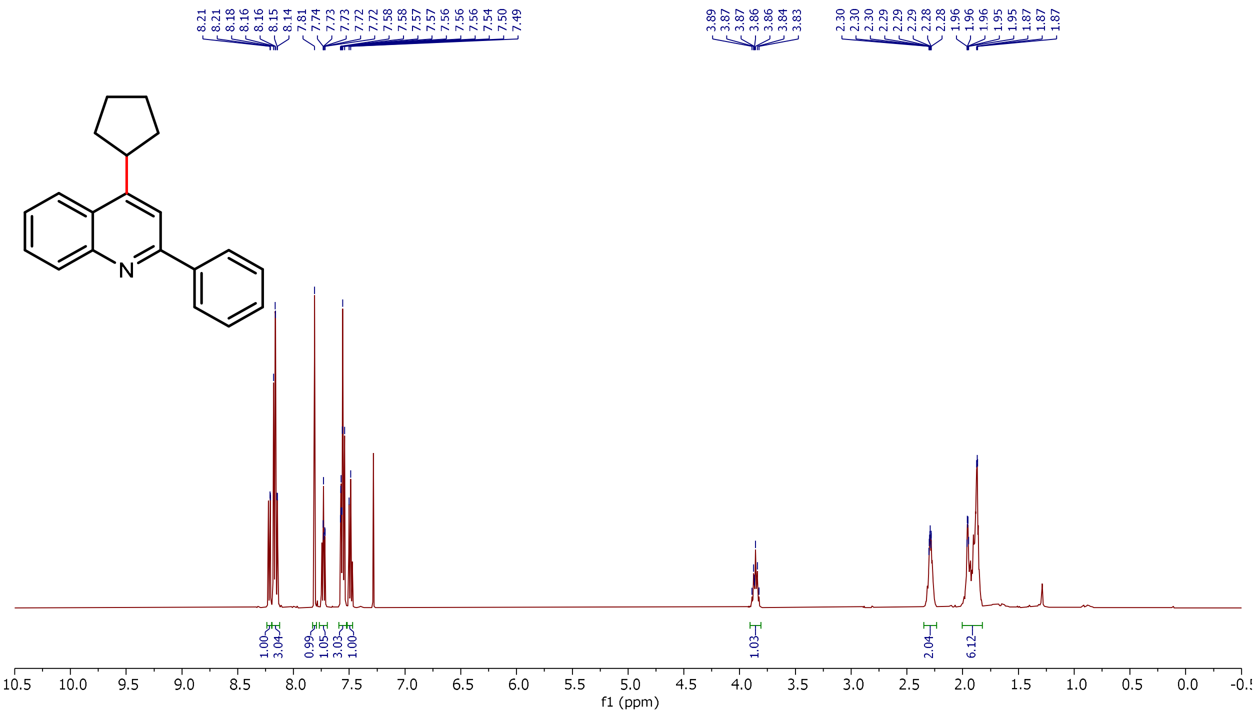

## Supplementary Figure 10b | $^{13}$C NMR (125 MHz, $CDCl_3$) of 4-cyclopentyl-2-phenylquinoline (4)

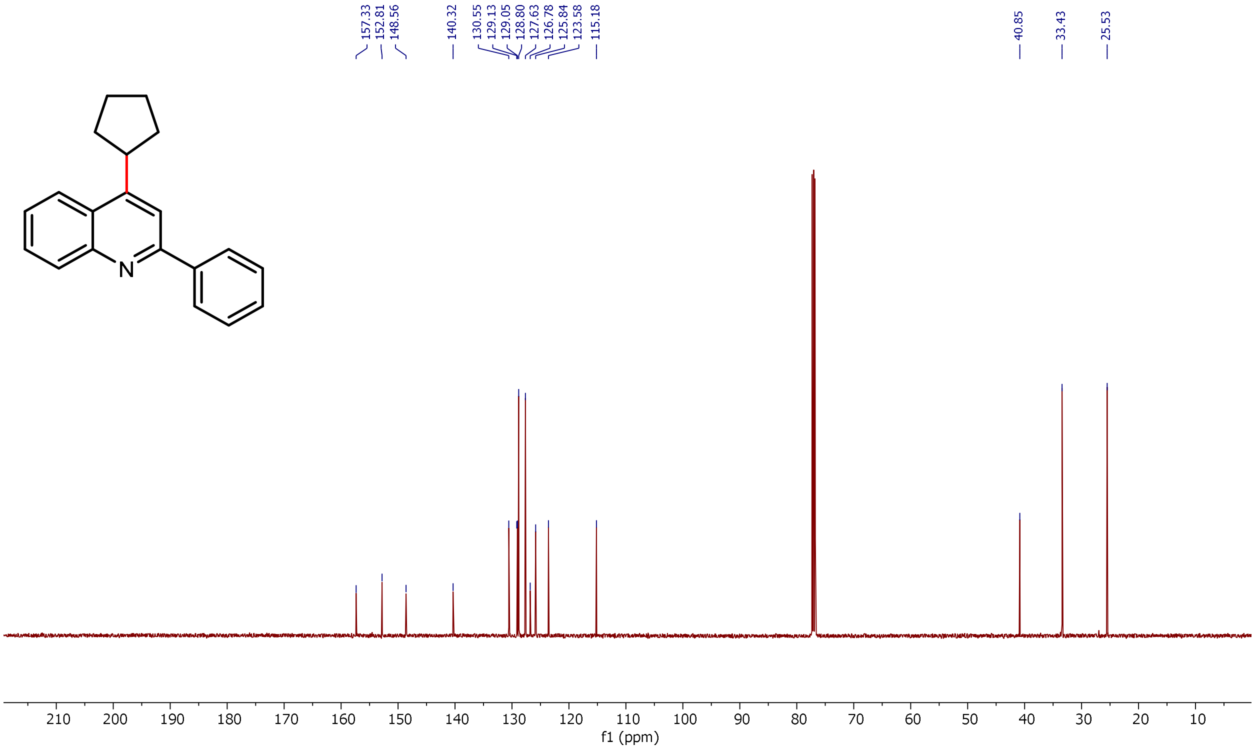

**Supplementary Figure 11a | $^1$H NMR (500 MHz, CDCl$_3$) of 4-cycloheptyl-2-phenylquinoline (5)**

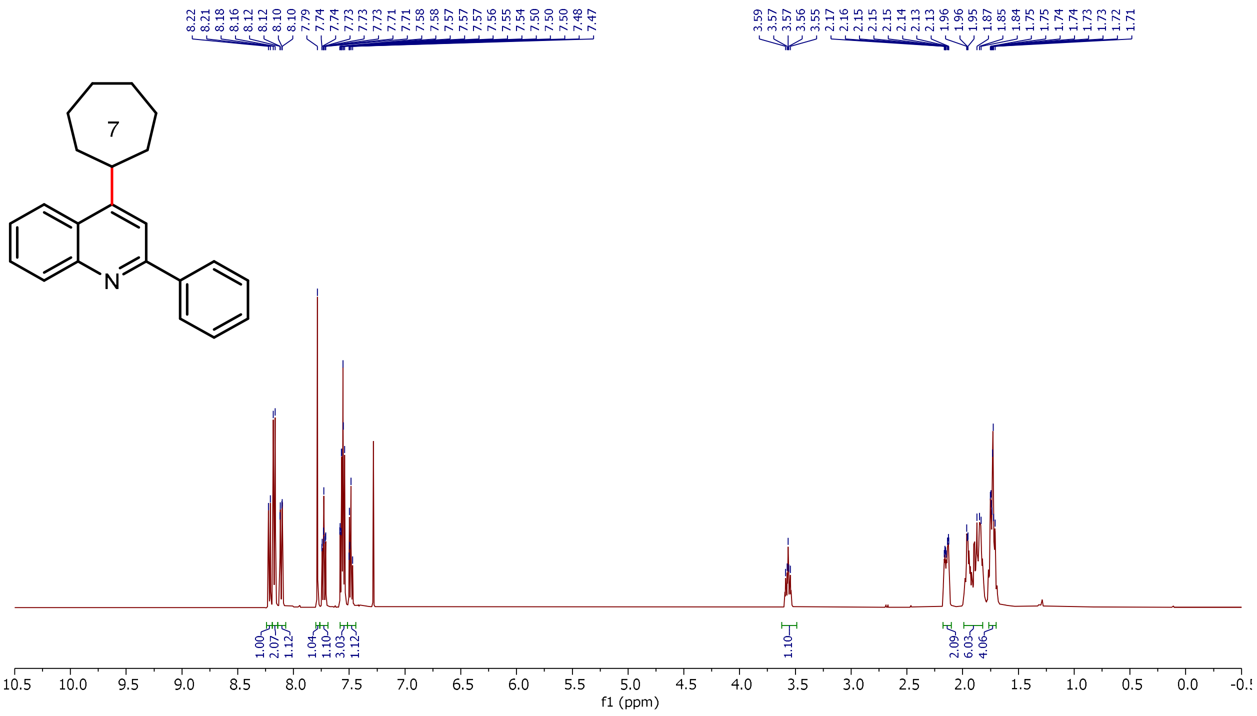

**Supplementary Figure 11b | $^{13}$C NMR (125 MHz, CDCl$_3$) of 4-cycloheptyl-2-phenylquinoline (5)**

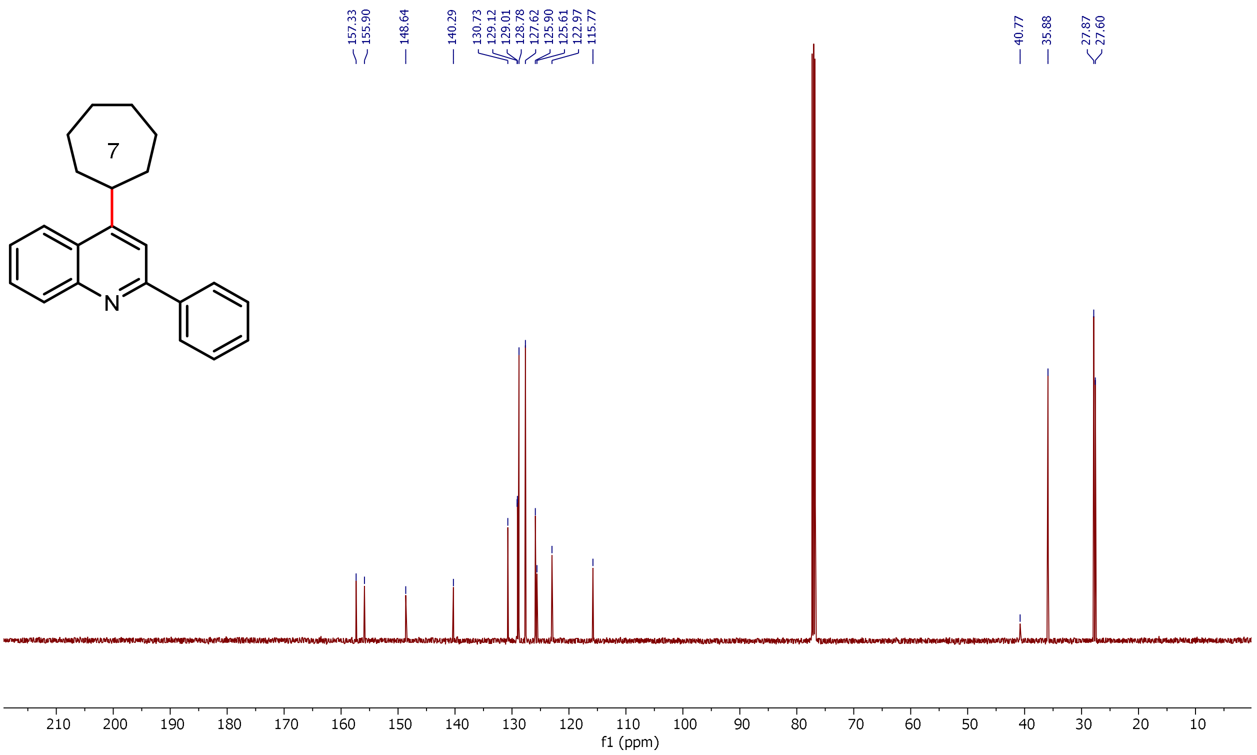

## Supplementary Figure 12a | $^1$H NMR (500 MHz, $CDCl_3$) of 4-cyclooctyl-2-phenylquinoline (6)

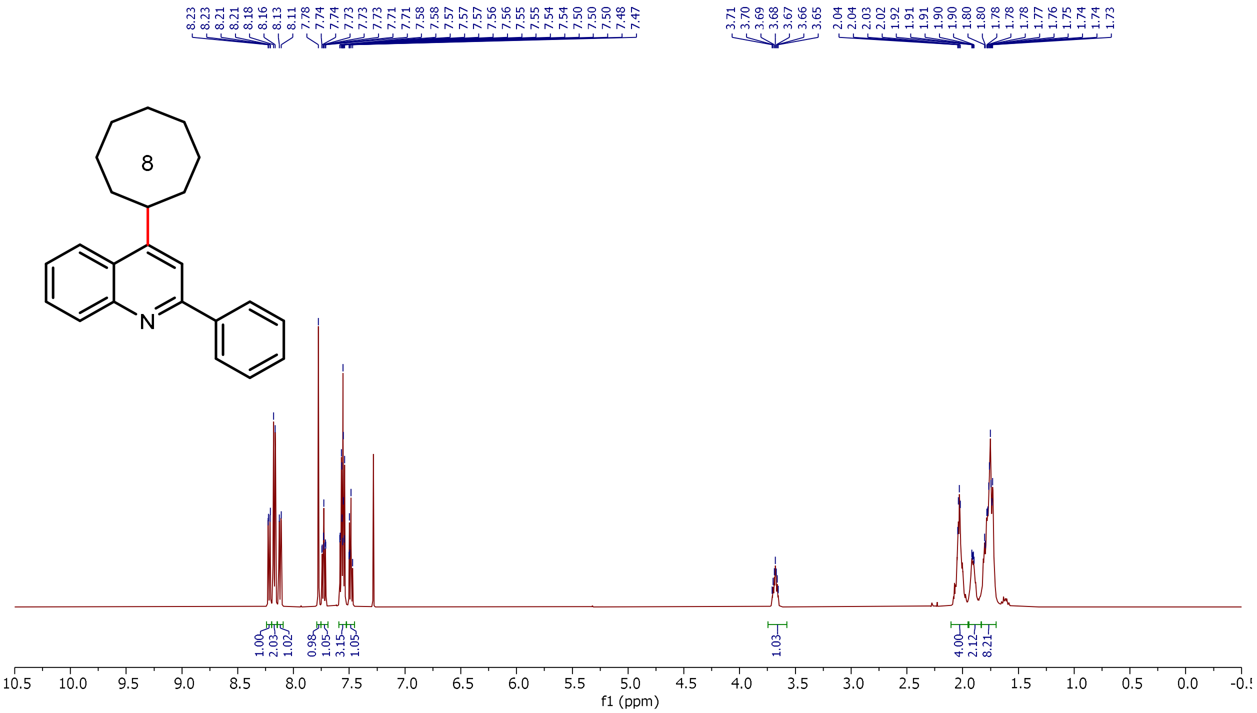


## Supplementary Figure 12b | $^{13}$C NMR (125 MHz, $CDCl_3$) of 4-cyclooctyl-2-phenylquinoline (6)

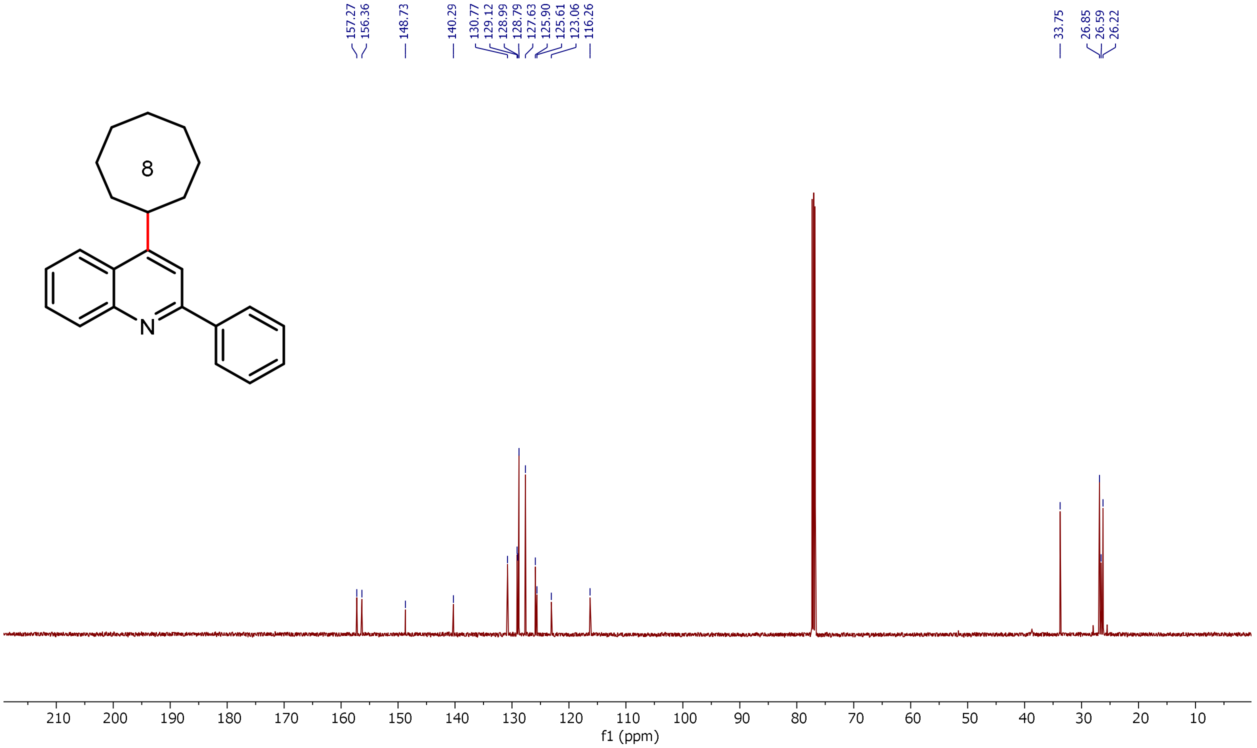

**Supplementary Figure 13a | $^1$H NMR (500 MHz, $CDCl_3$) of 4-cyclododecyl-2-phenylquinoline (7)**

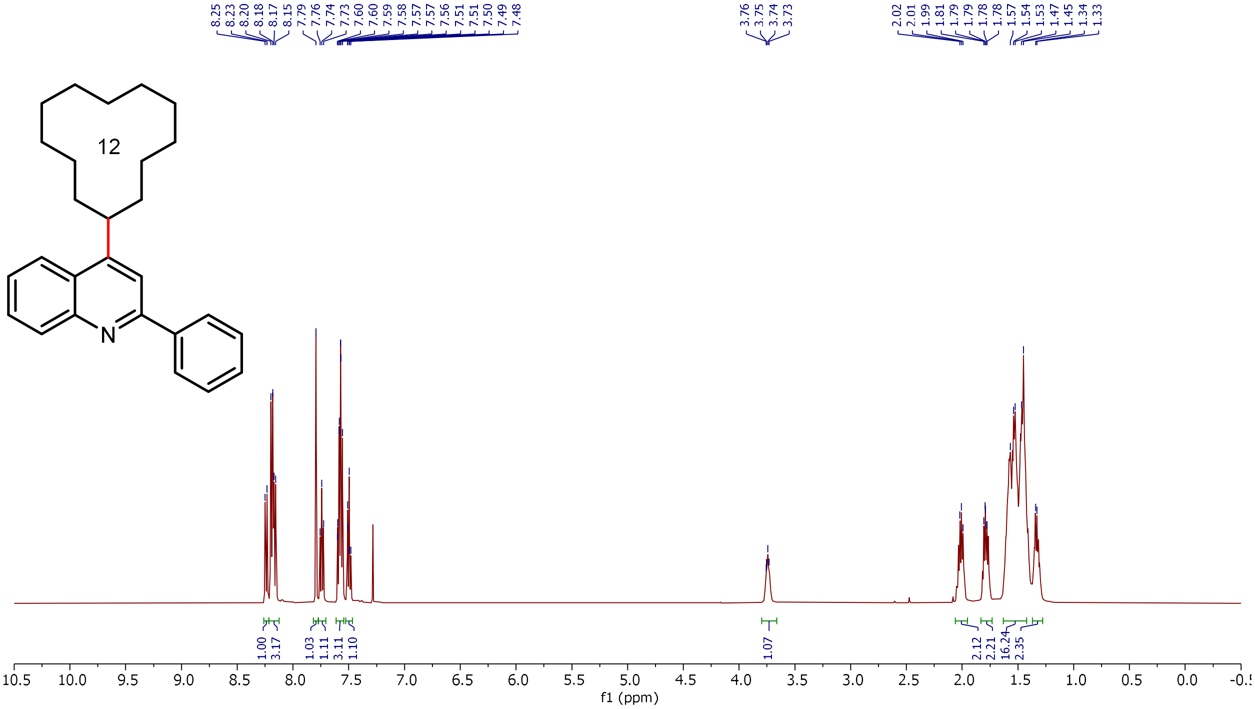

**Supplementary Figure 13b | $^{13}$C NMR (125 MHz, $CDCl_3$) of 4-cyclododecyl-2-phenylquinoline (7)**

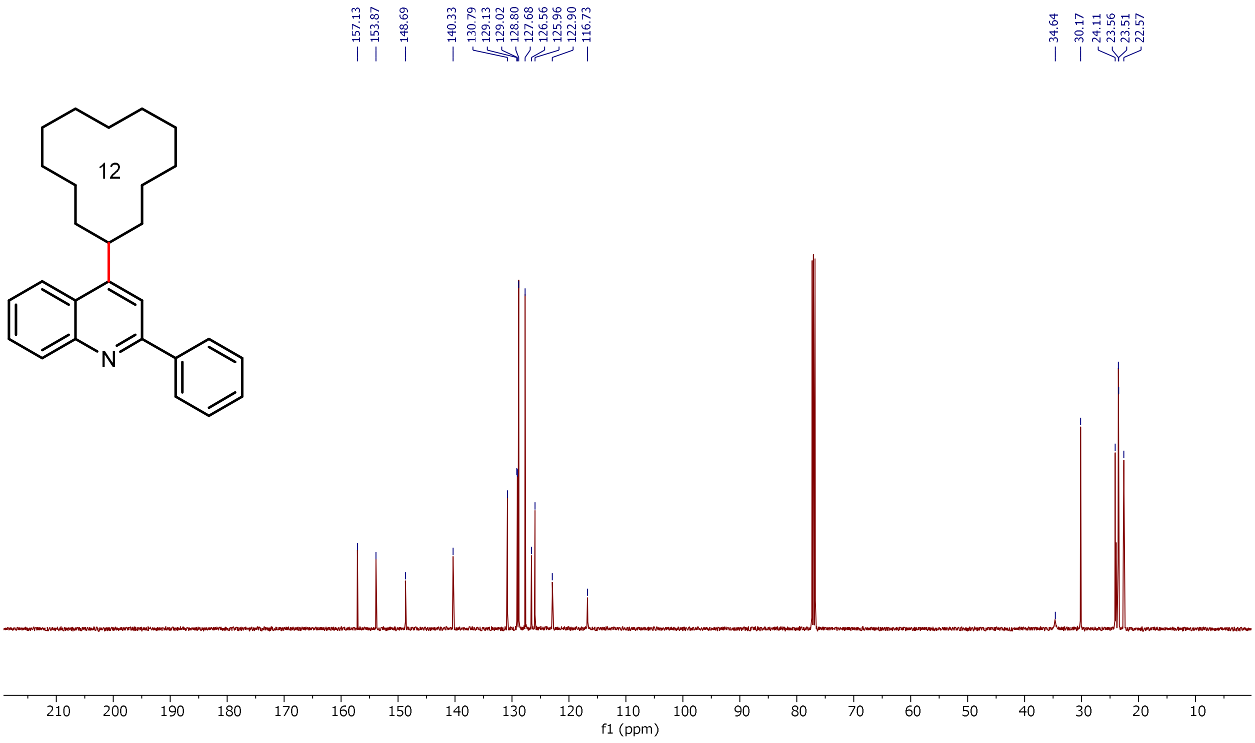

**Supplementary Figure 14a | $^1$H NMR (500 MHz, $CDCl_3$) of 4-bicyclo[2.2.1]heptan-2-yl)-2-phenylquinoline (8)**

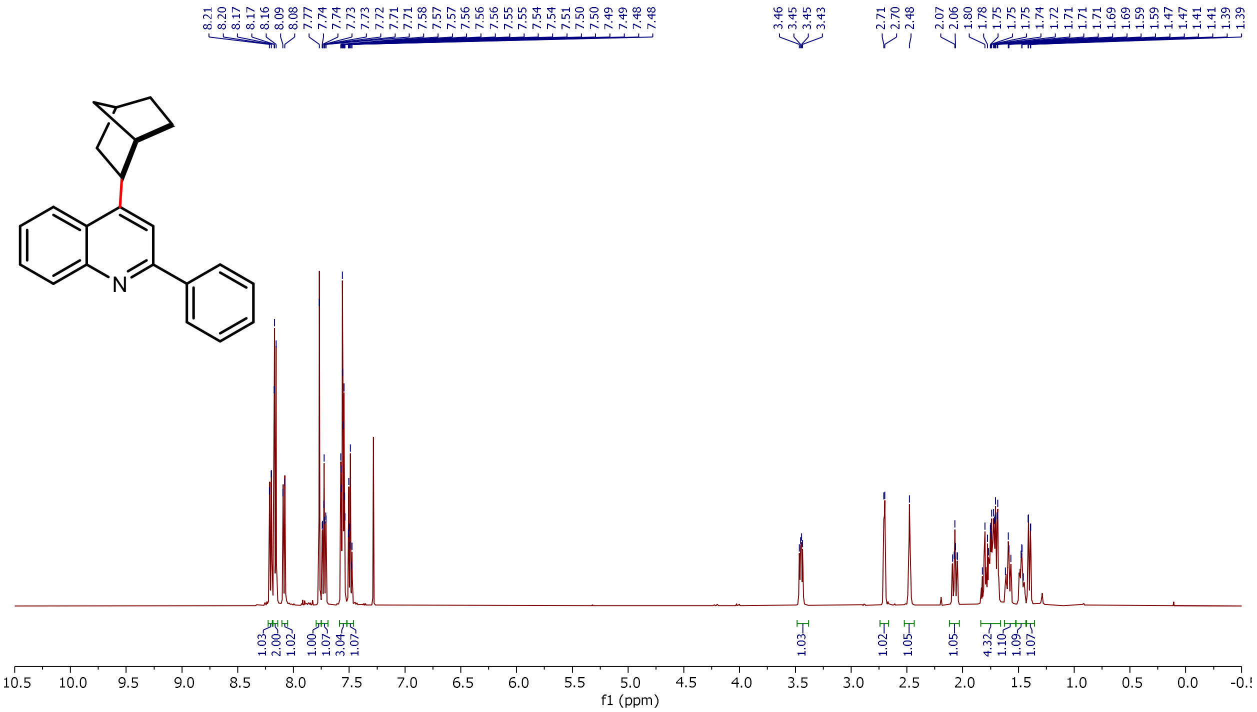

**Supplementary Figure 14b, $^{13}$C NMR (125 MHz, $CDCl_3$) of 4-bicyclo[2.2.1]heptan-2-yl)-2-phenylquinoline (8)**

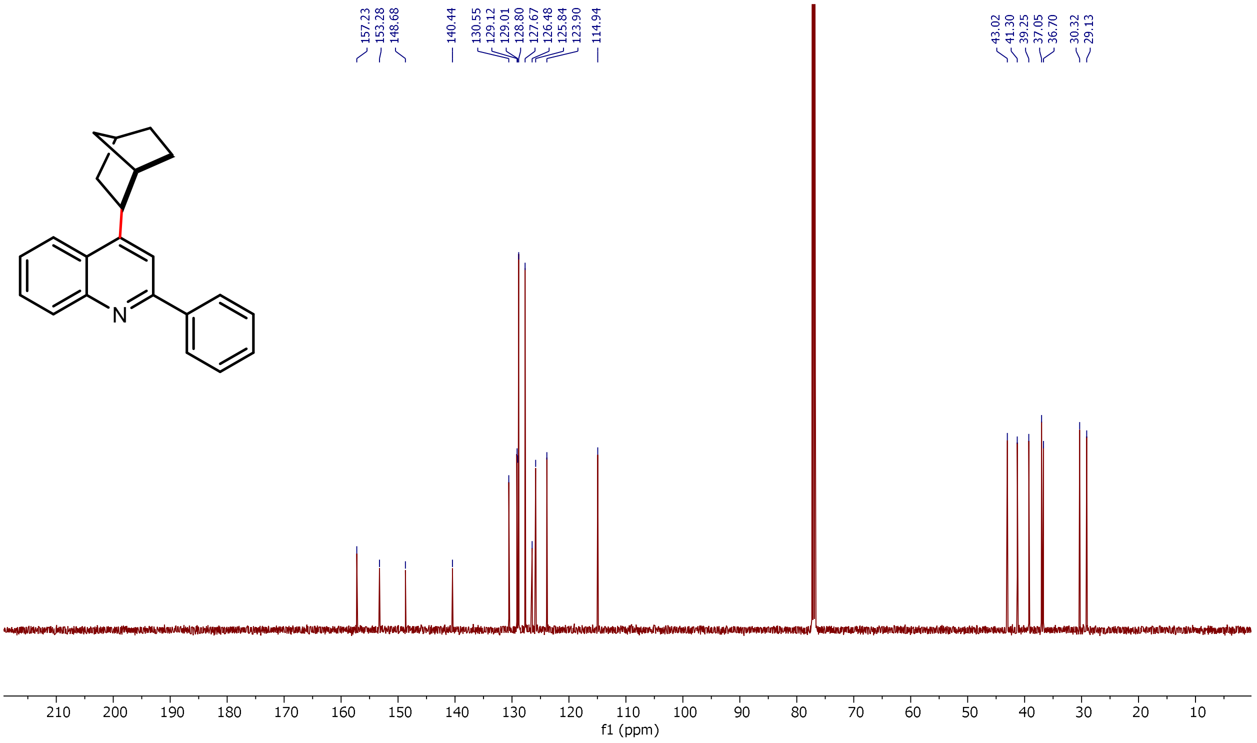

**Supplementary Figure 15a | $^{1}$H NMR (500 MHz, $CDCl_3$) of (4-adamantan-1-yl)-2-phenylquinoline (9a) and 4-adamantan-2-yl)-2-phenylquinoline (9b), 1:0.9 congeners**

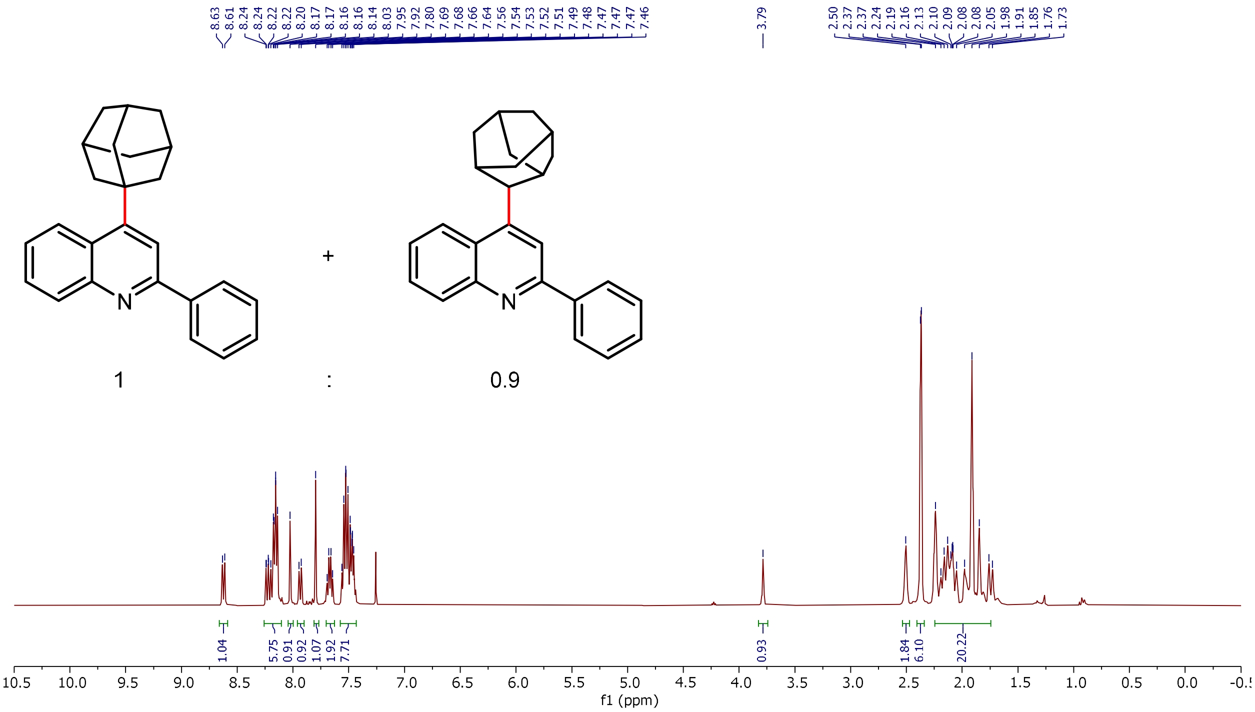


**Supplementary Figure 15b | $^{13}$C NMR (125 MHz, $CDCl_3$) of (4-adamantan-1-yl)-2-phenylquinoline (9a) and 4-adamantan-2-yl)-2-phenylquinoline (9b), 1:0.9 congeners**

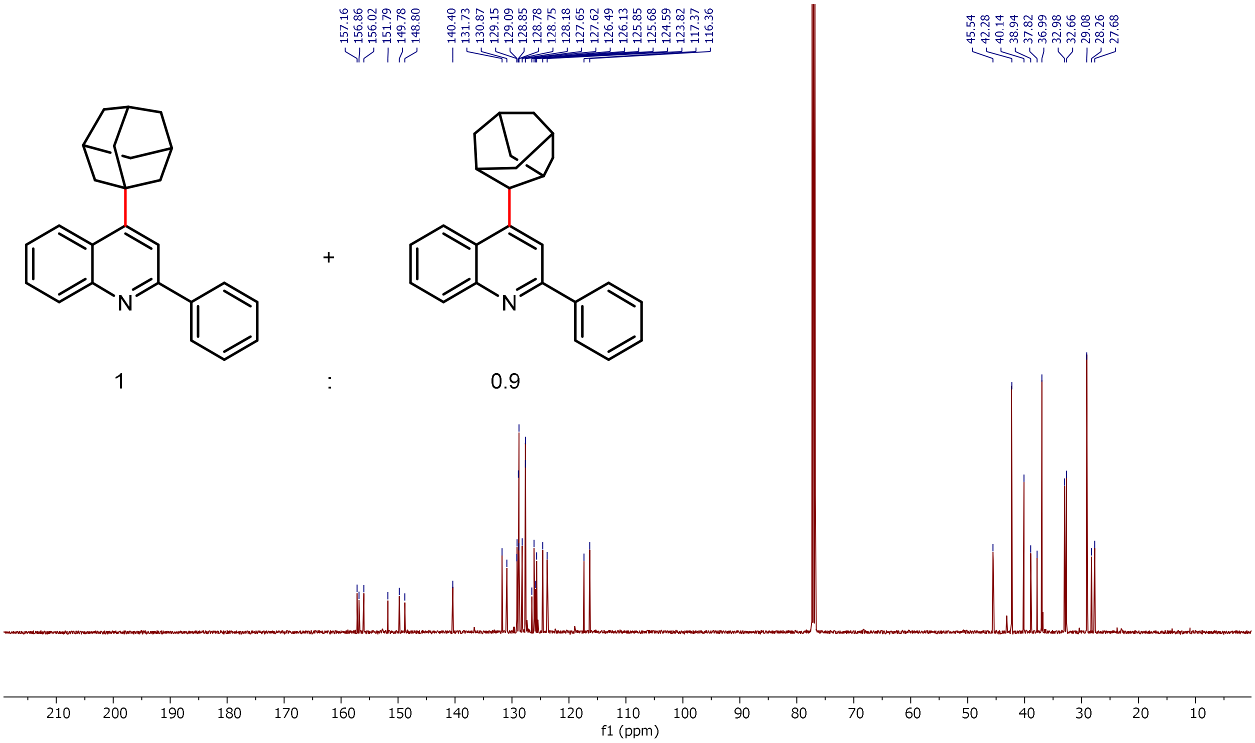

**Supplementary Figure 16a | $^{1}$H NMR (500 MHz, $CDCl_3$) of 3-(2-phenylquinolin-4-yl)cyclopentan-1-one (10)**

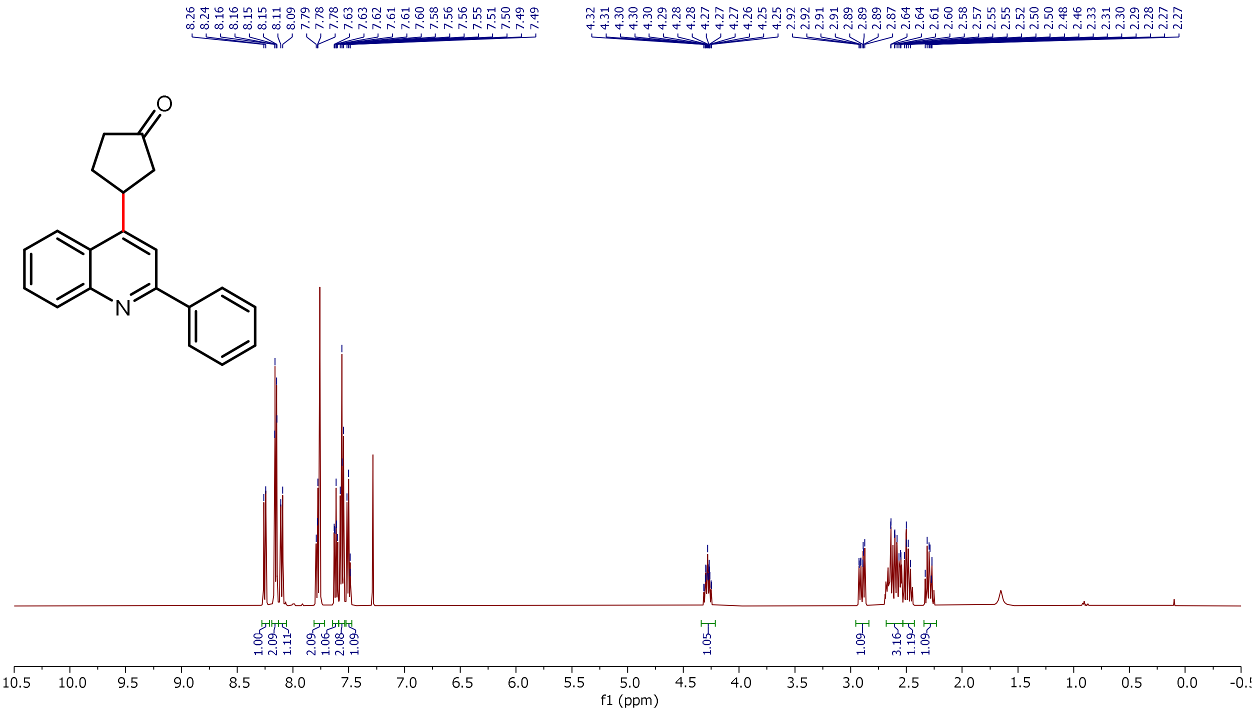

**Supplementary Figure 16b | $^{13}$C NMR (125 MHz, $CDCl_3$) of 3-(2-phenylquinolin-4-yl)cyclopentan-1-one (10)**

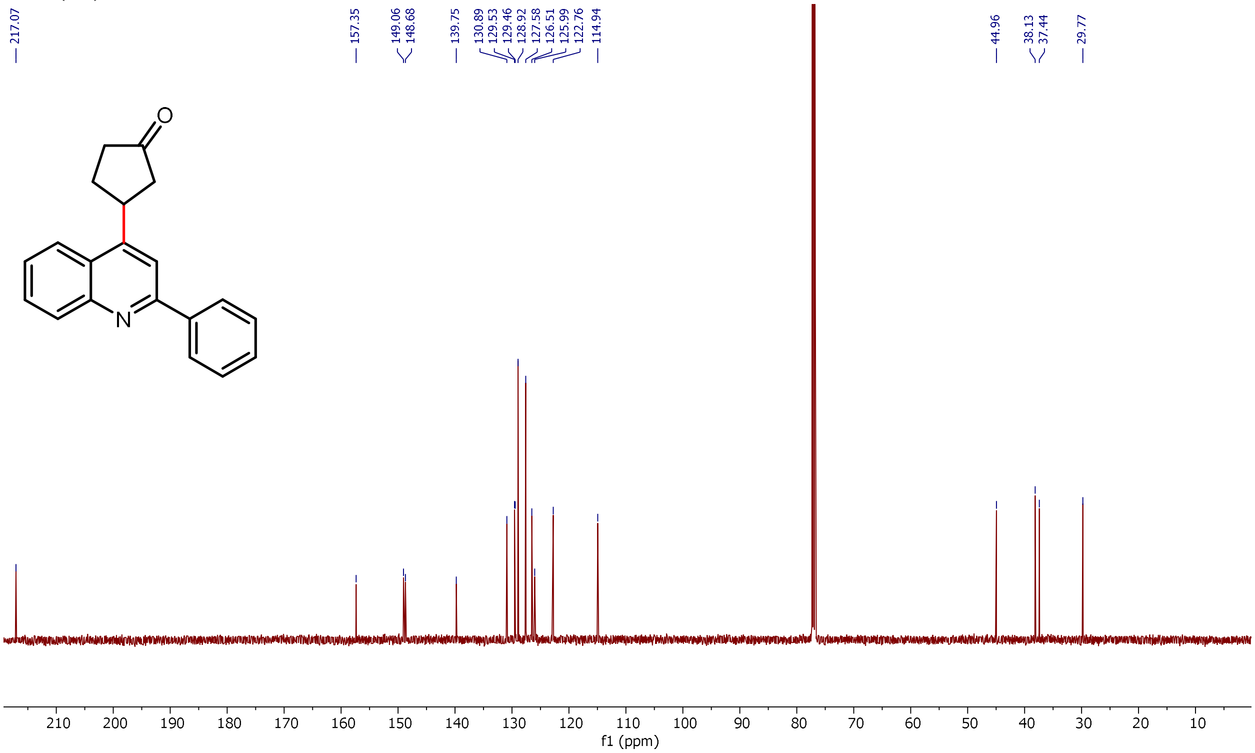

**Supplementary Figure 17a | $^1$H NMR (500 MHz, $CDCl_3$) of 4-(3,5-dimethylbenzyl)-2-phenylquinoline (11)**

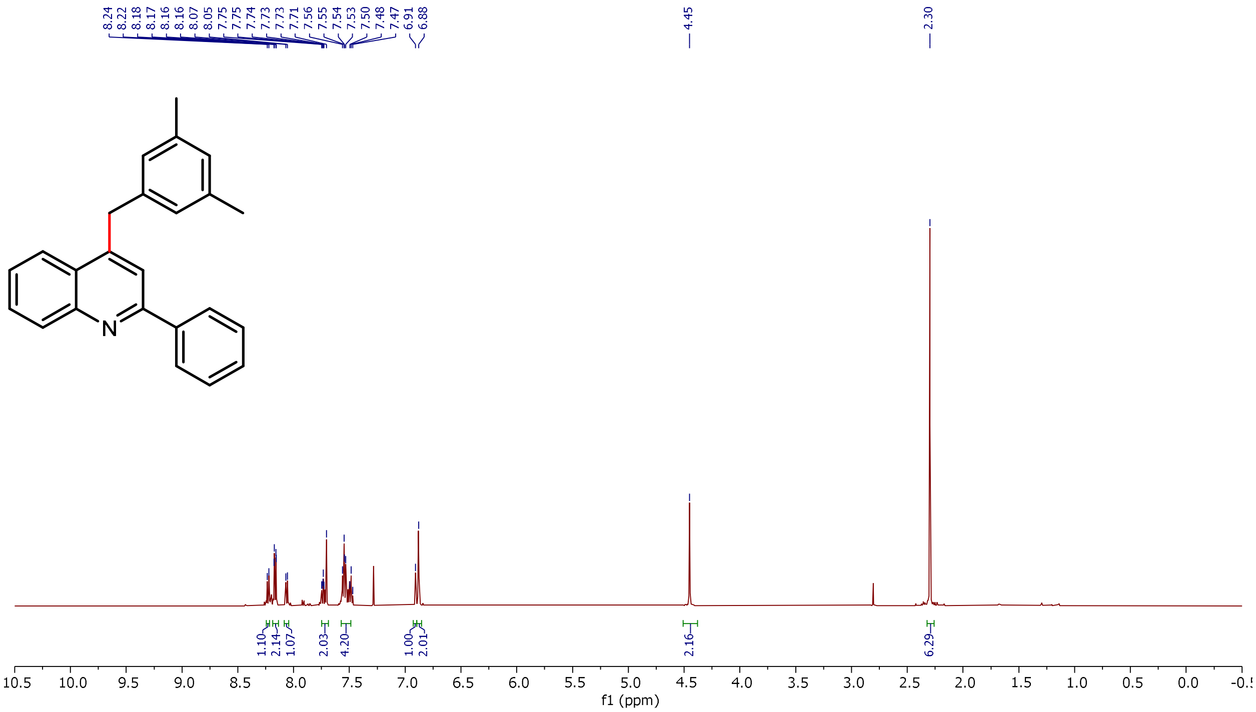


**Supplementary Figure 17b | $^{13}$C NMR (125 MHz, $CDCl_3$) of 4-(3,5-dimethylbenzyl)-2-phenylquinolin (11)**

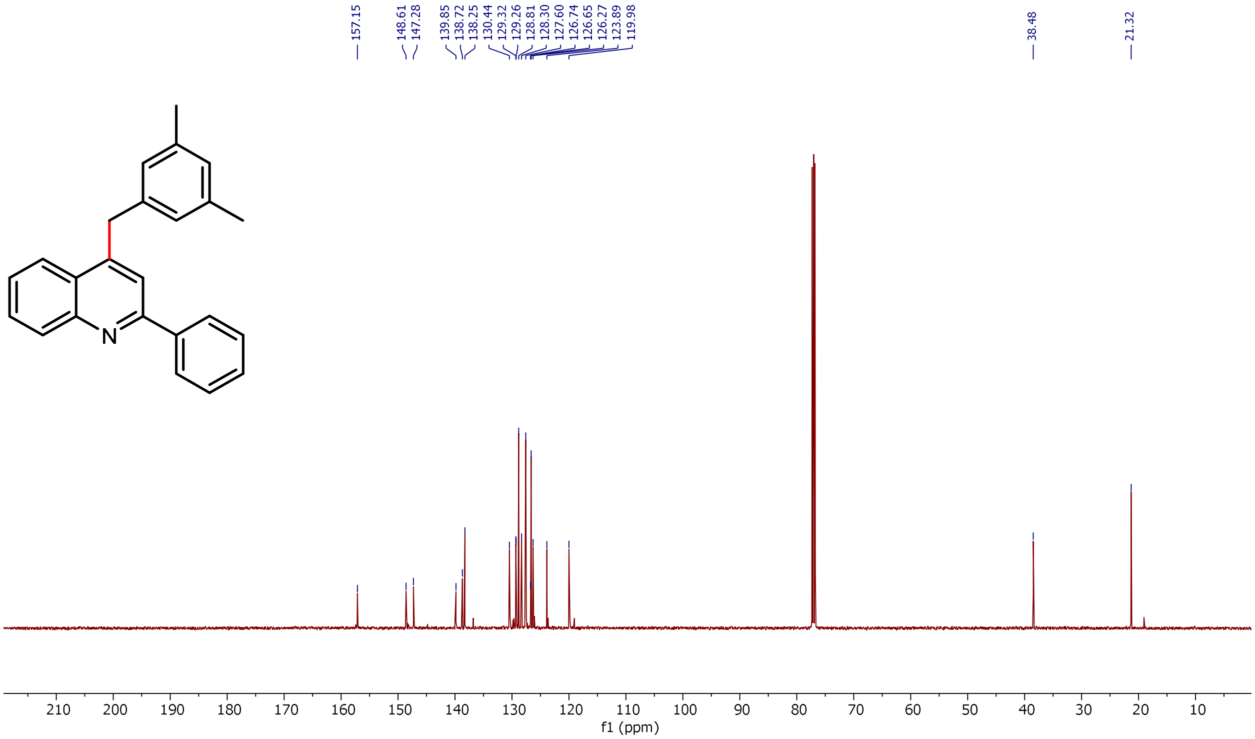

**Supplementary Figure 18a | $^{1}$H NMR (500 MHz, $CDCl_3$) of 4-(4-methylbenzyl)-2-phenylquinoline (12)**

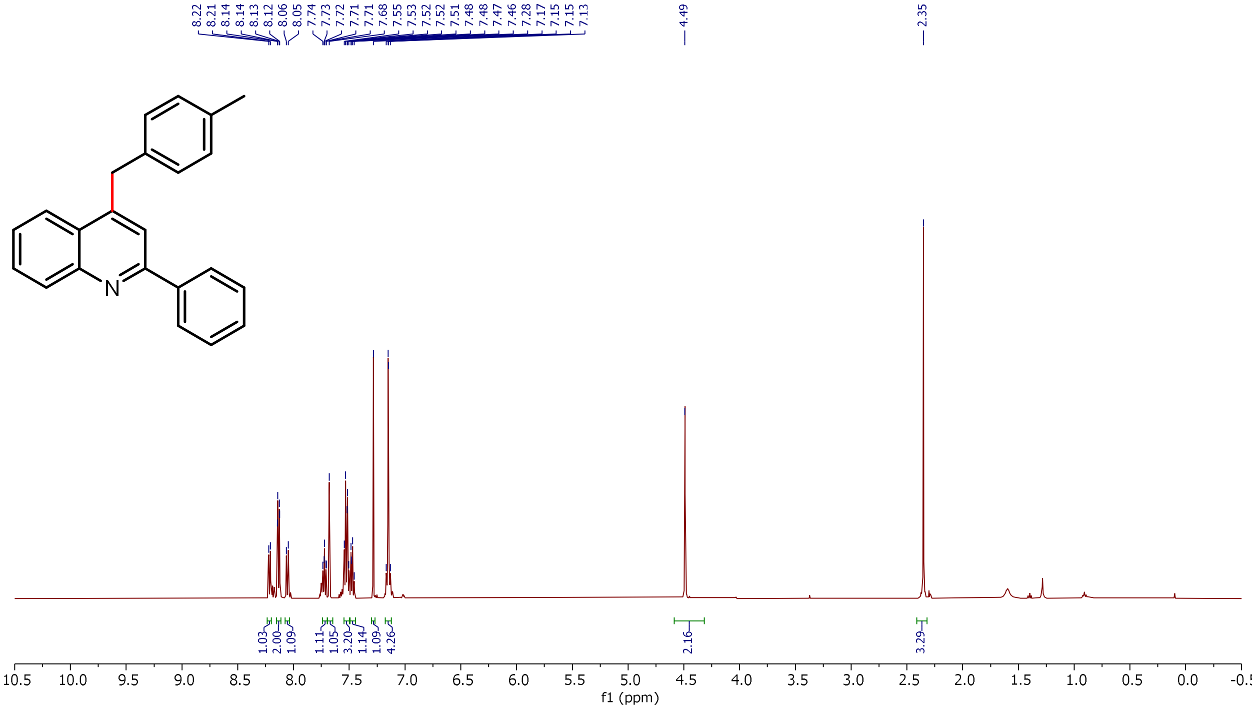


**Supplementary Figure 18b | $^{13}$C NMR (125 MHz, $CDCl_3$) of 4-(4-methylbenzyl)-2-phenylquinoline (12)**

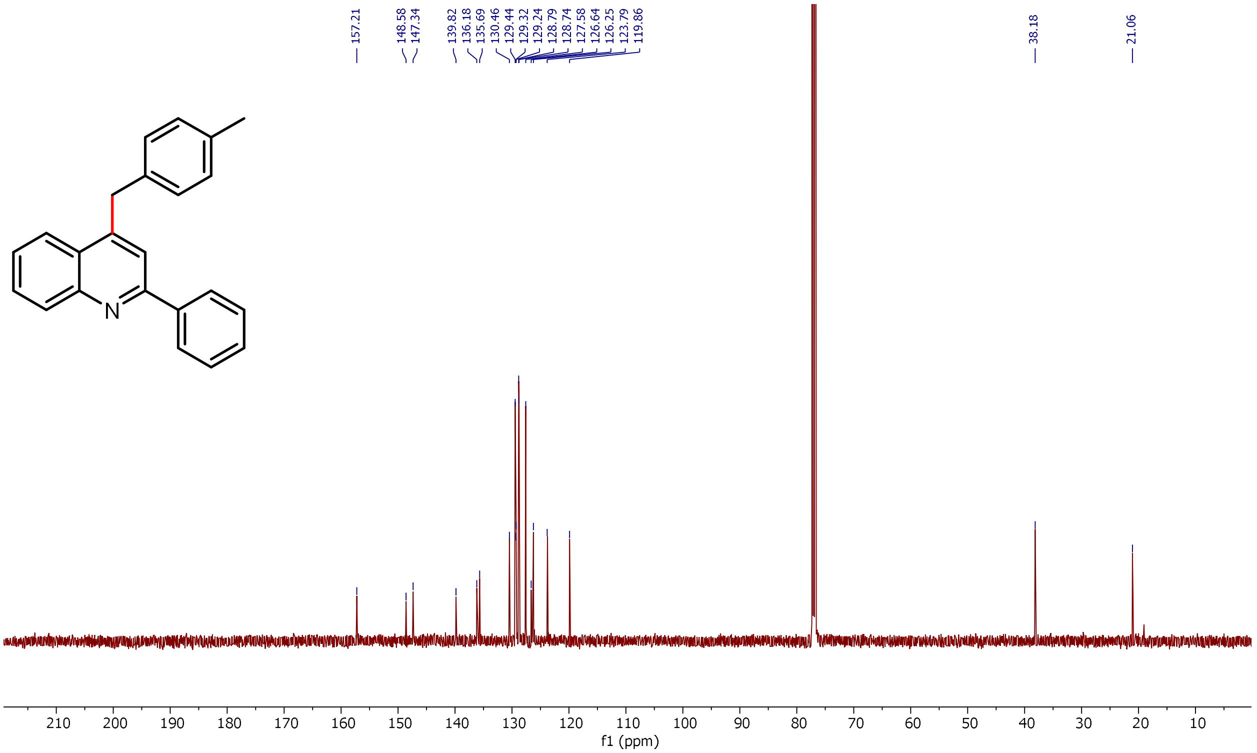

**Supplementary Figure 19a | $^{1}H$ NMR (500 MHz, $CDCl_3$) of 4-(4-methoxylbenzyl)-2-phenylquinoline (13)**

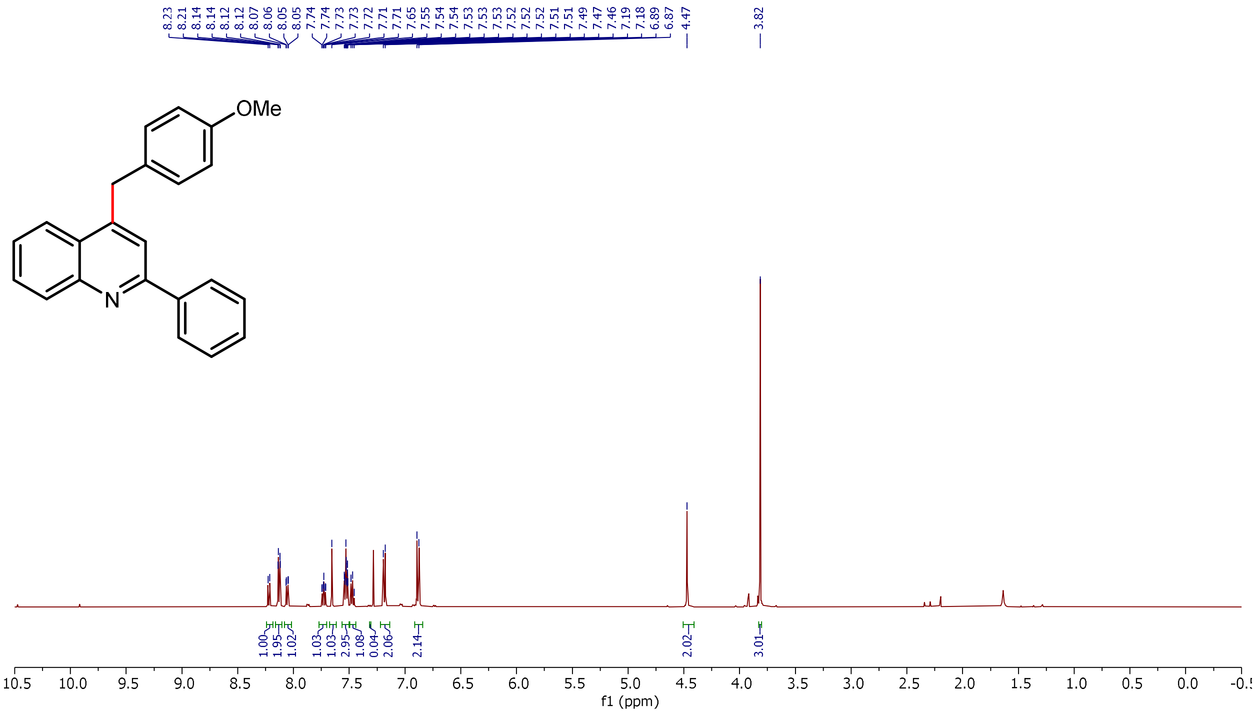


**Supplementary Figure 19b | $^{13}C$ NMR (125 MHz, $CDCl_3$) of 4-(4-methoxylbenzyl)-2-phenylquinoline (13)**

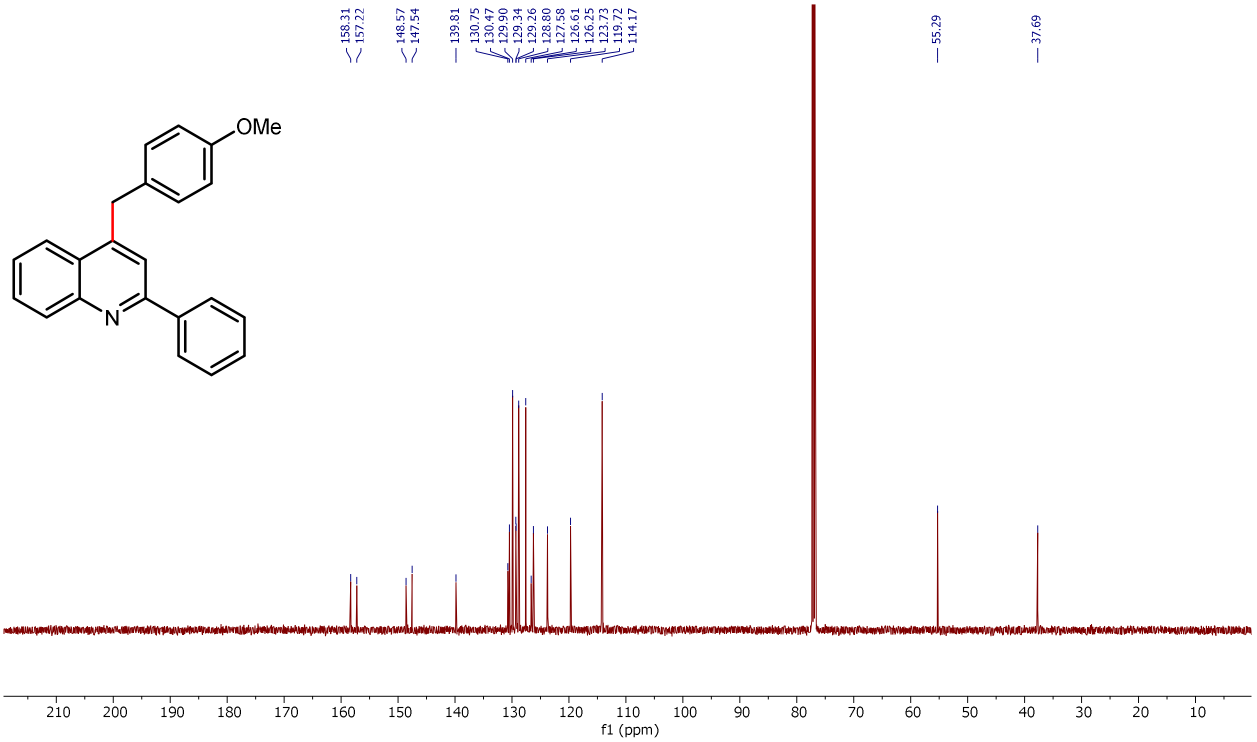

**Supplementary Figure 20a | $^{1}$H NMR (500 MHz, $CDCl_3$) of 4-([1,1'-biphenyl]-4-ylmethyl)-2-phenylquinoline (14)**

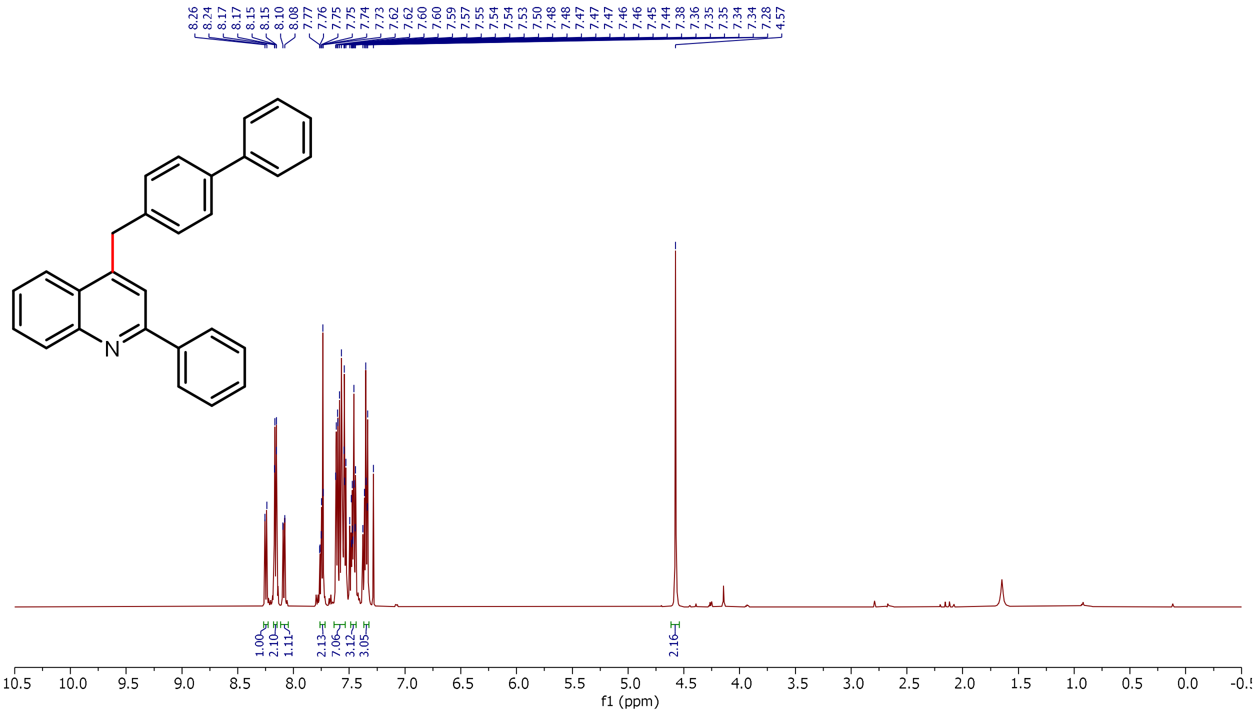


**Supplementary Figure 20b | $^{13}$C NMR (125 MHz, $CDCl_3$) of 4-([1,1'-biphenyl]-4-ylmethyl)-2-phenylquinoline (14)**

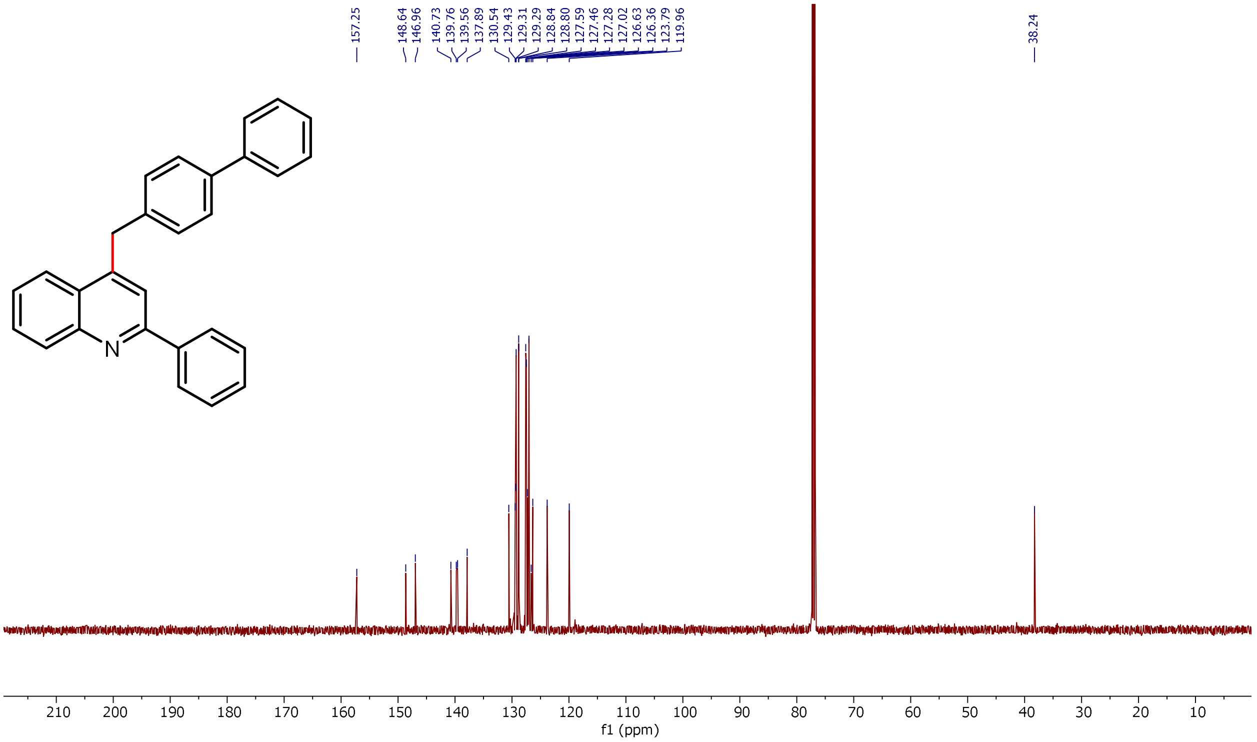

## Supplementary Figure 21a | $^1$H NMR (500 MHz, $CDCl_3$) of 4-ethyl-2-phenylquinoline (15)

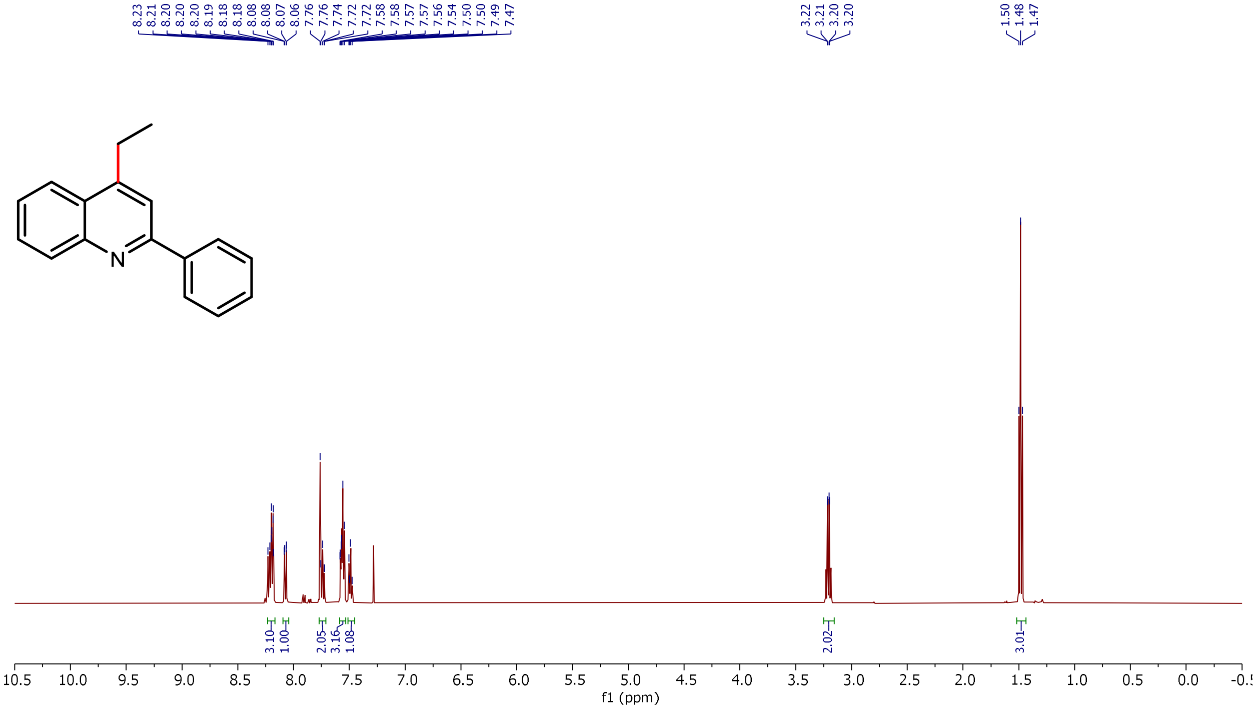


## Supplementary Figure 21b | $^{13}$C NMR (125 MHz, $CDCl_3$) of 4-ethyl-2-phenylquinoline (15)

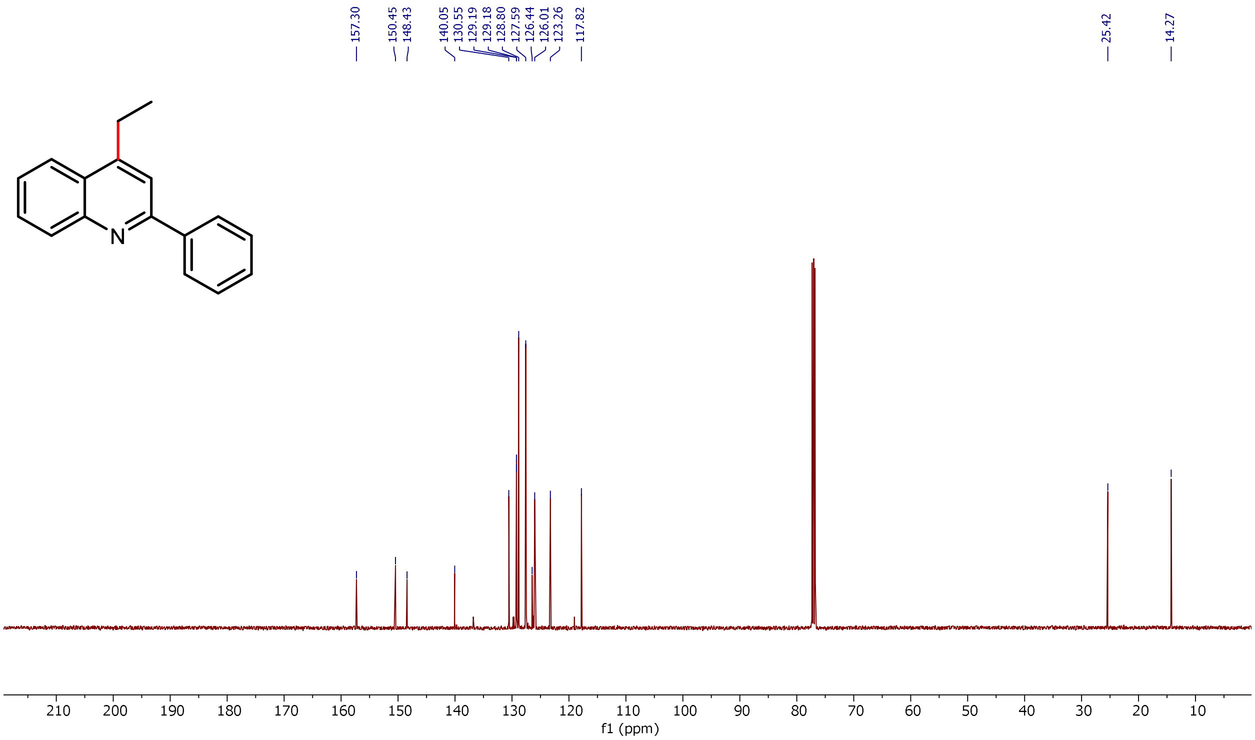

**Supplementary Figure 22a | $^{1}$H NMR (500 MHz, $CDCl_3$) of 4-(2-methoxyethyl)-2-phenylquinoline (16)**

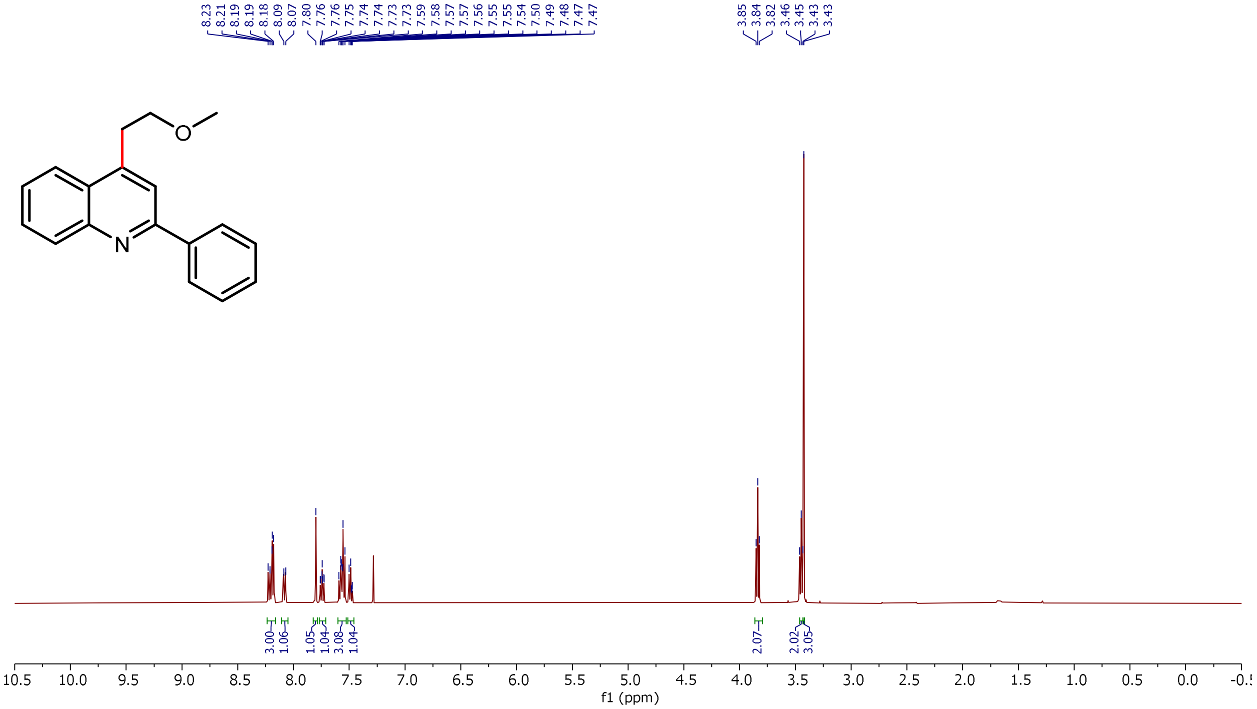


**Supplementary Figure 22b | $^{13}$C NMR (125 MHz, $CDCl_3$) of 4-(2-methoxyethyl)-2-phenylquinoline (16)**

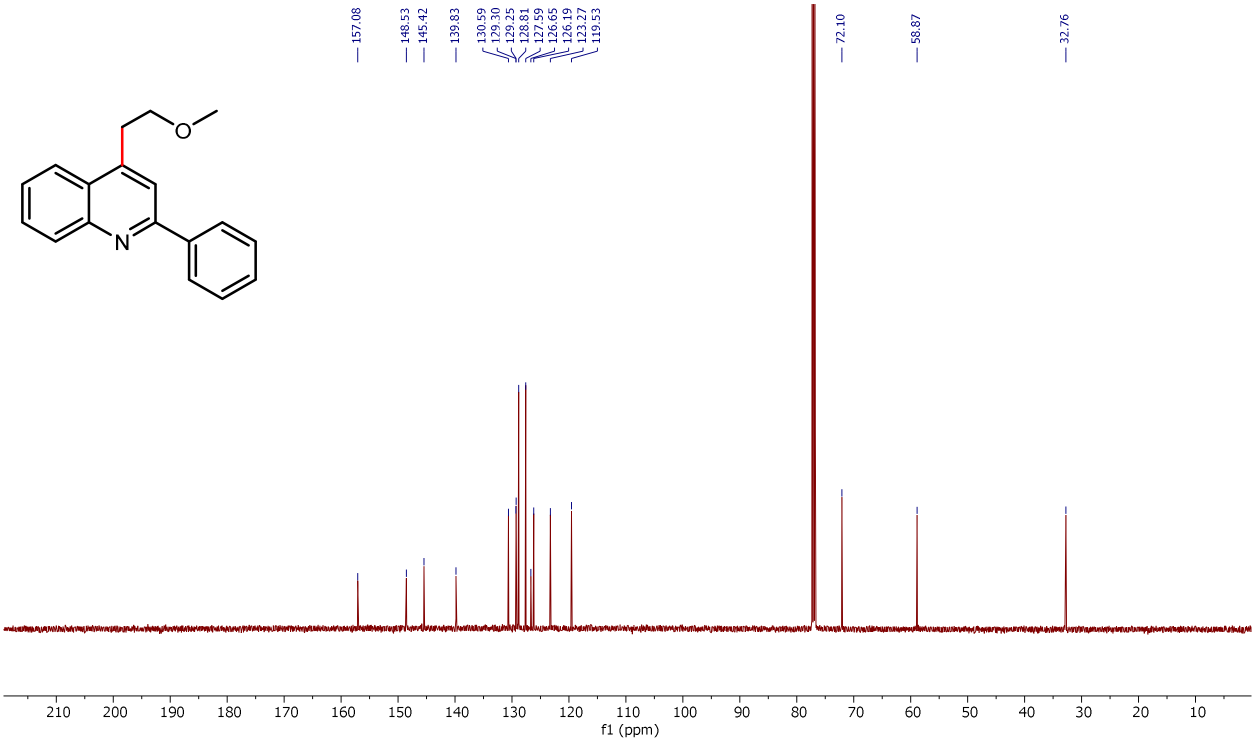

## Supplementary Figure 23a | $^{1}$H NMR (500 MHz, CDCl$_3$) of 4-(1-ethoxyethyl)-2-phenylquinoline (17)

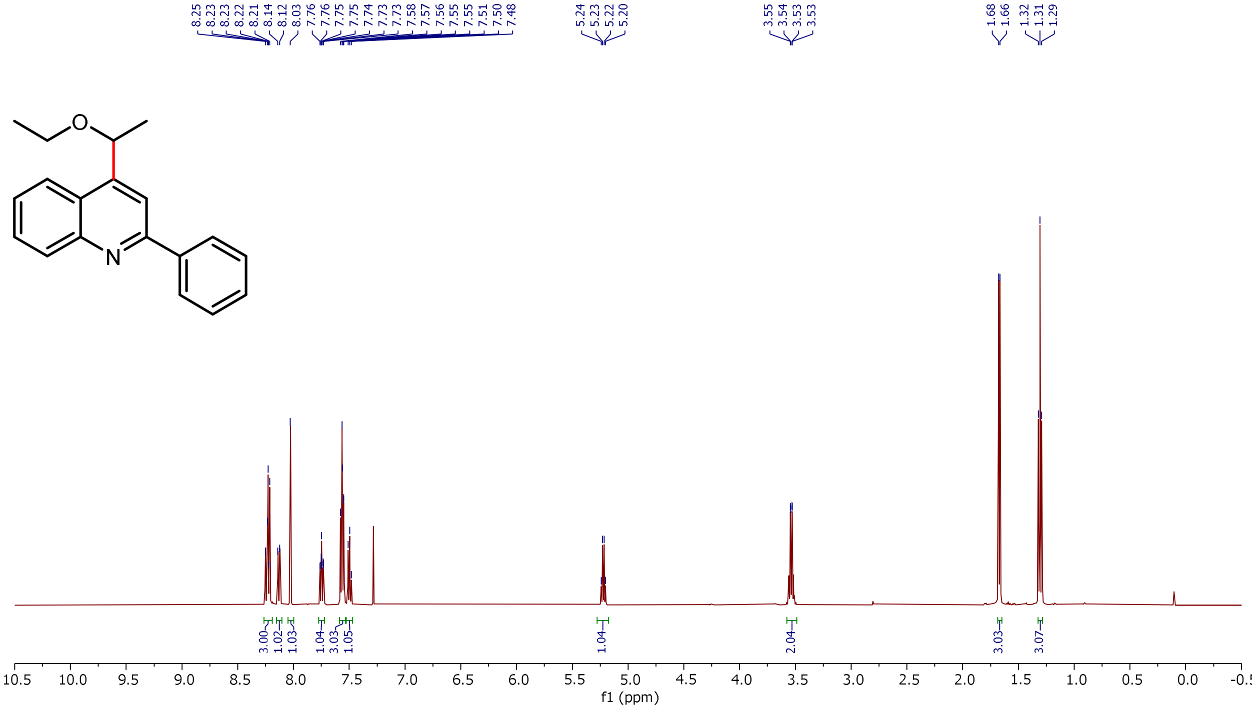


## Supplementary Figure 23b | $^{13}$C NMR (125 MHz, CDCl$_3$) of 4-(1-ethoxyethyl)-2-phenylquinoline (17)

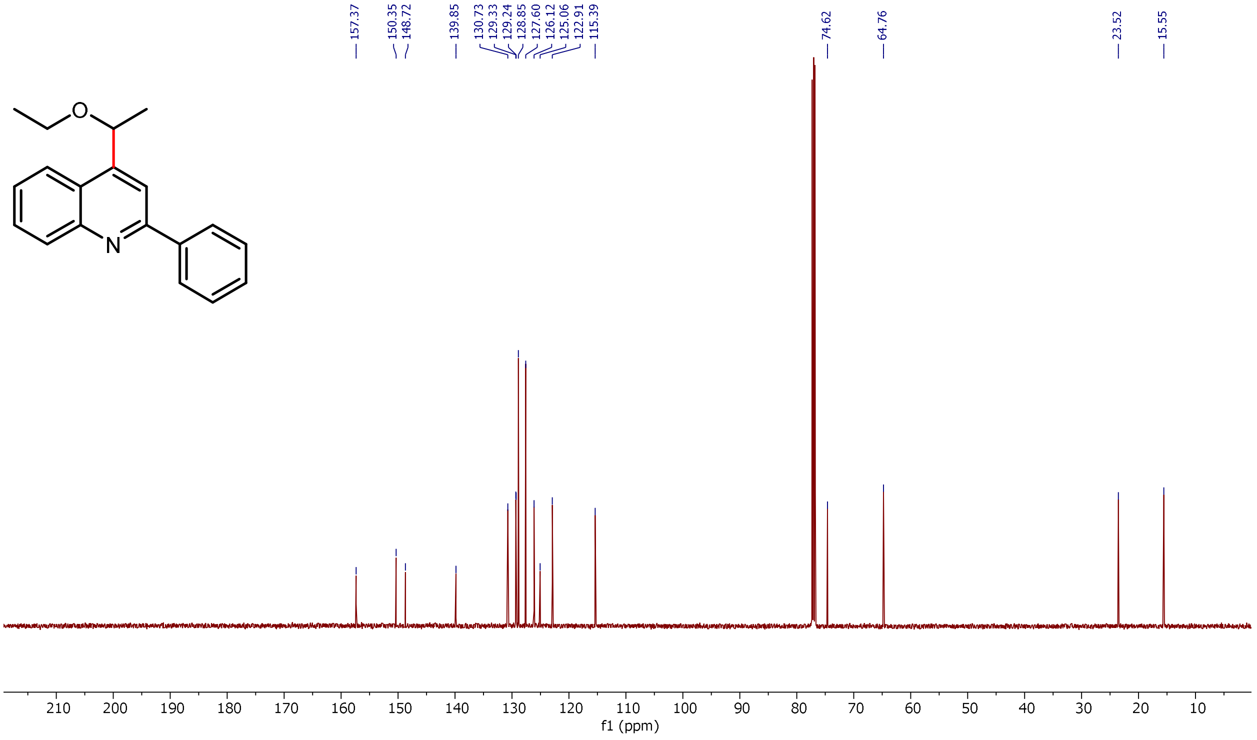

**Supplementary Figure 24a | $^{1}$H NMR (500 MHz, $CDCl_3$) of 4-(1,2-dimethoxyethyl)-2-phenylquinoline (18)**

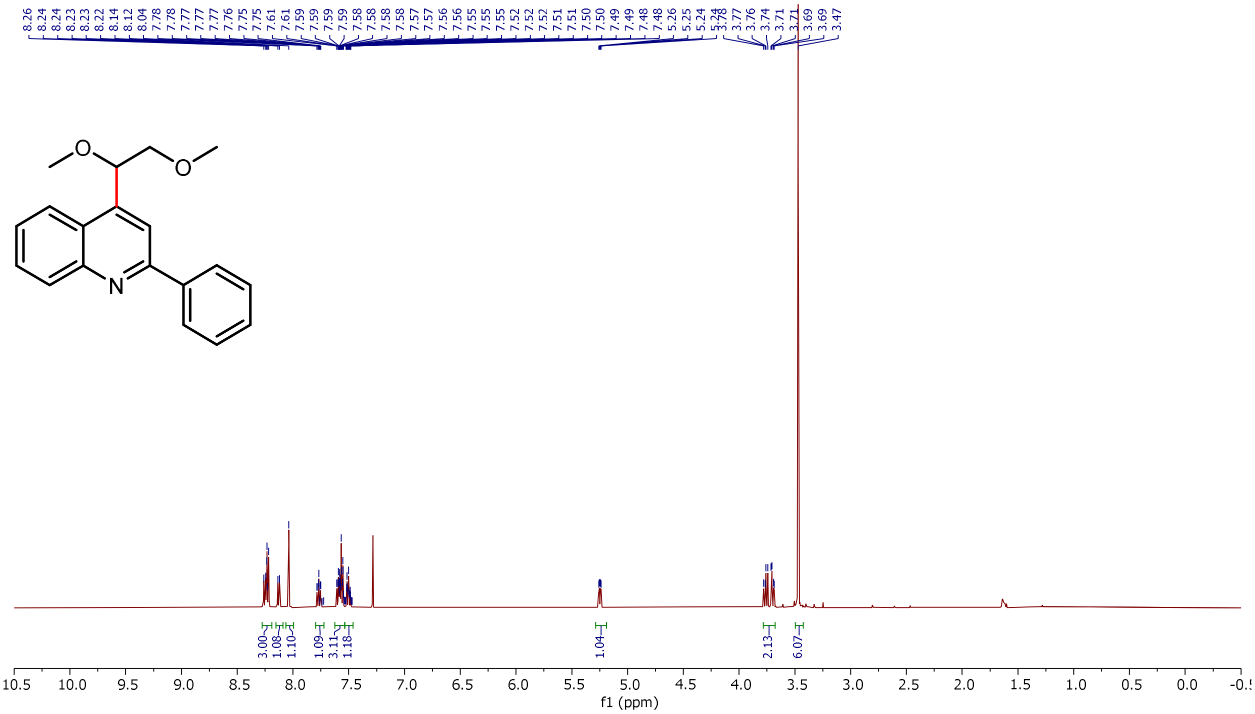


**Supplementary Figure 24b | $^{13}$C NMR (125 MHz, $CDCl_3$) of 4-(1,2-dimethoxyethyl)-2-phenylquinoline (18)**

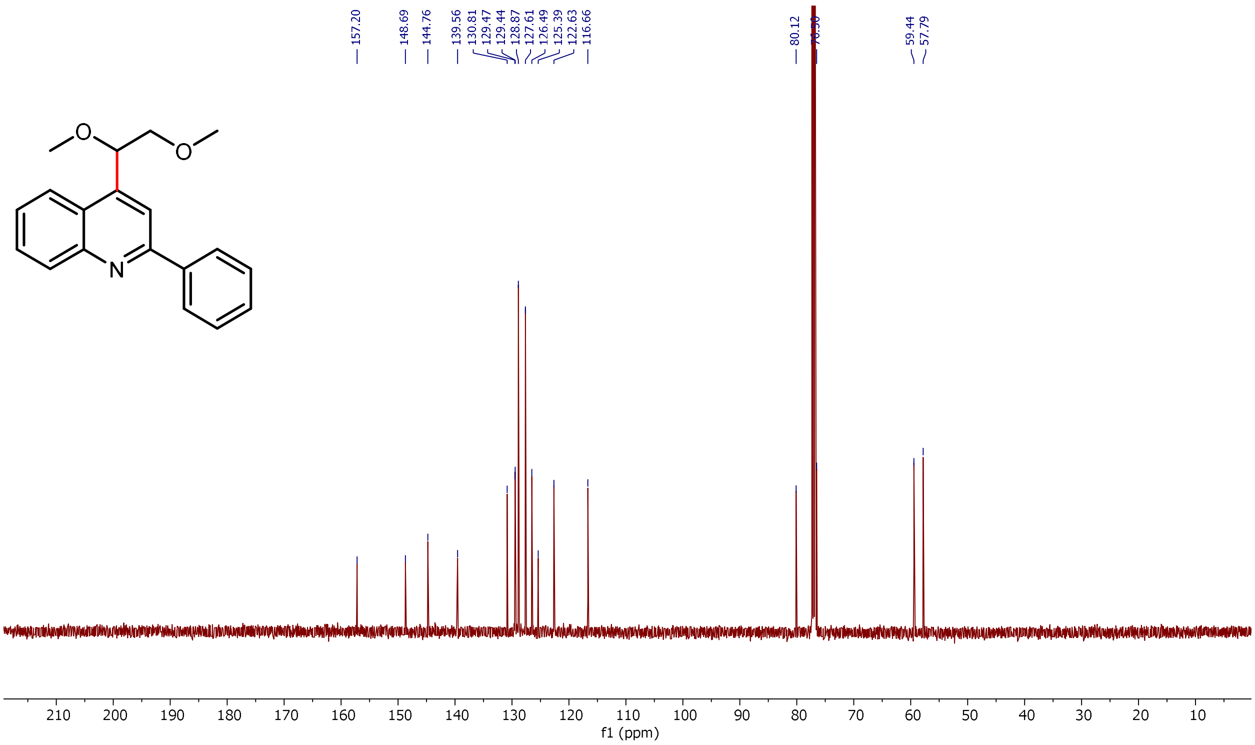

**Supplementary Figure 25a | $^{1}$H NMR (500 MHz, $CDCl_3$) of 4-(2-phenylquinolin-4-yl)butan-1-ol (19)**

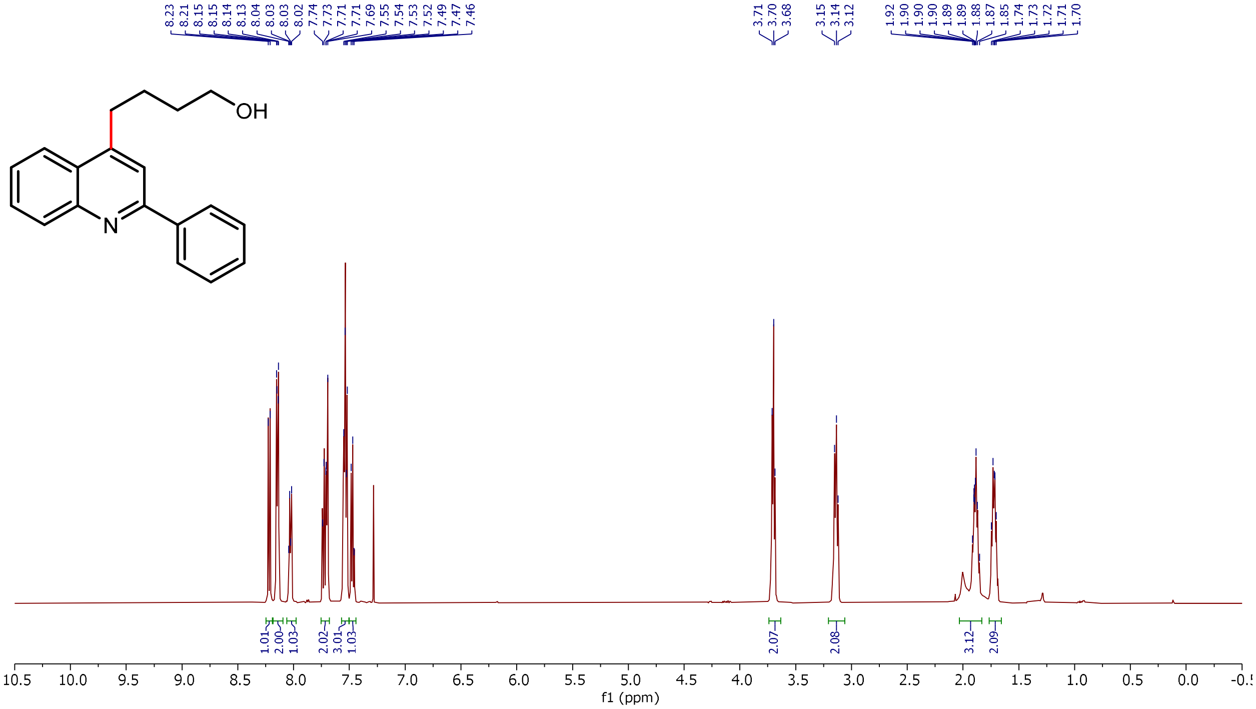


**Supplementary Figure 25b | $^{13}$C NMR (125 MHz, $CDCl_3$) of 4-(2-phenylquinolin-4-yl)butan-1-ol (19)**

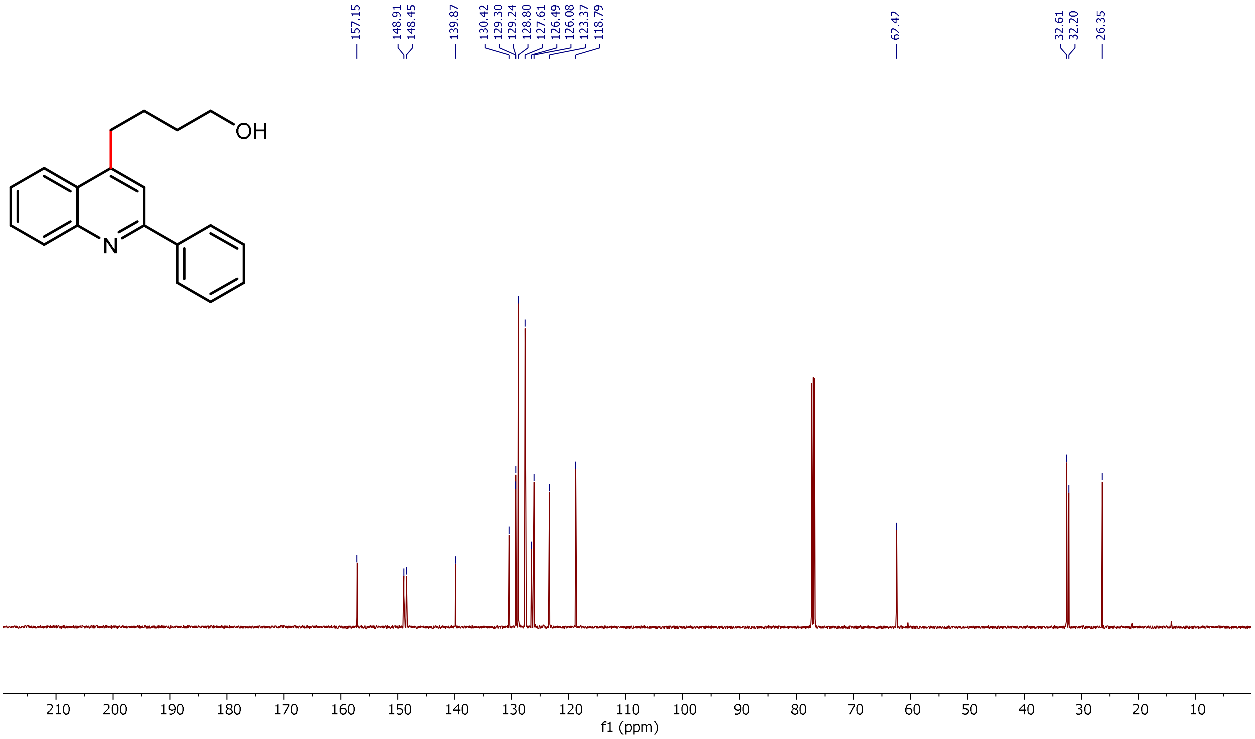

**Supplementary Figure 26a | $^1$H NMR (500 MHz, $CDCl_3$) of 5-(2-phenylquinolin-4-yl)pentan-2-ol (20)**

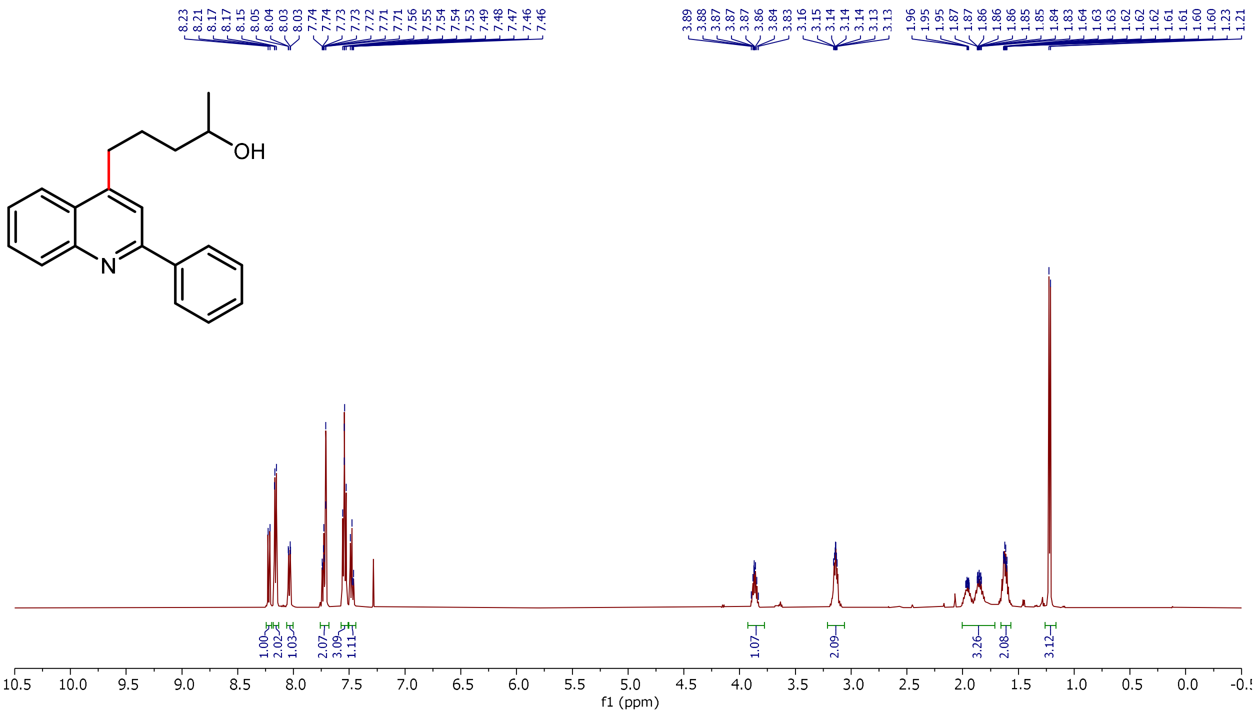

**Supplementary Figure 26b | $^{13}$C NMR (125 MHz, $CDCl_3$) of 5-(2-phenylquinolin-4-yl)pentan-2-ol (20)**

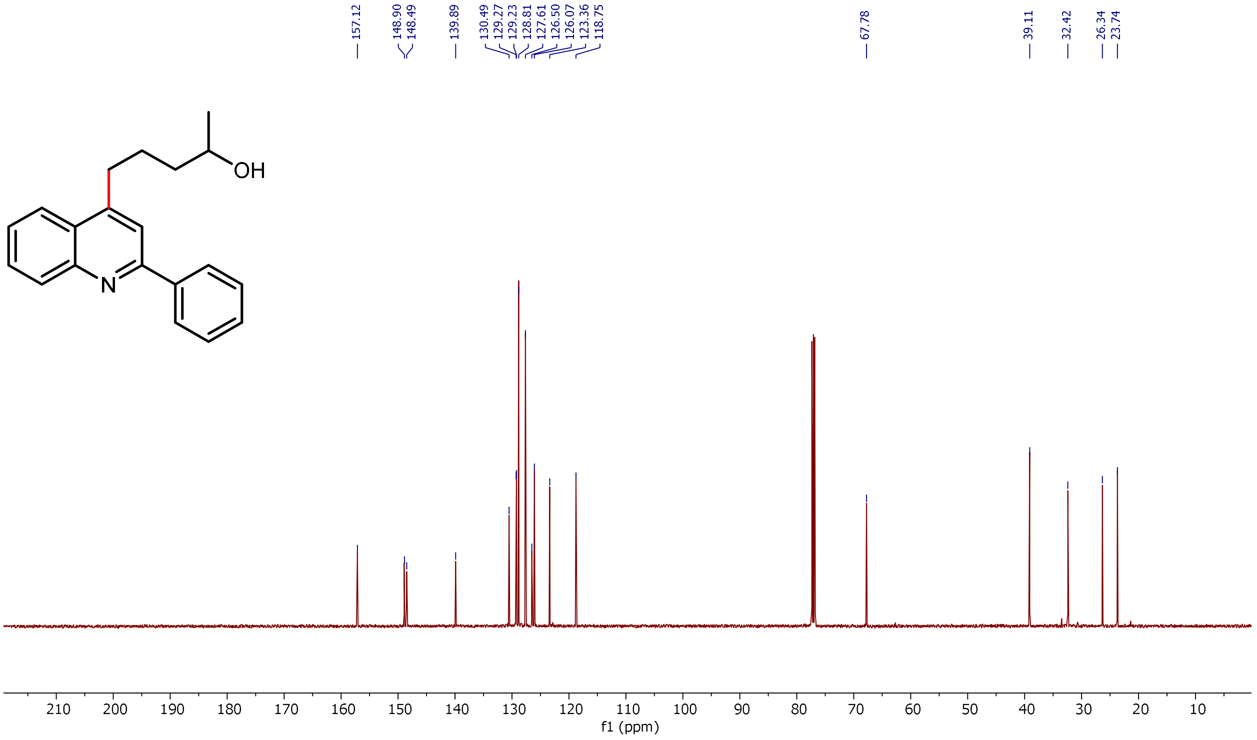

**Supplementary Figure 27a | $^1$H NMR (500 MHz, $CDCl_3$) of 4-methyl-2-phenylquinoline (21)**

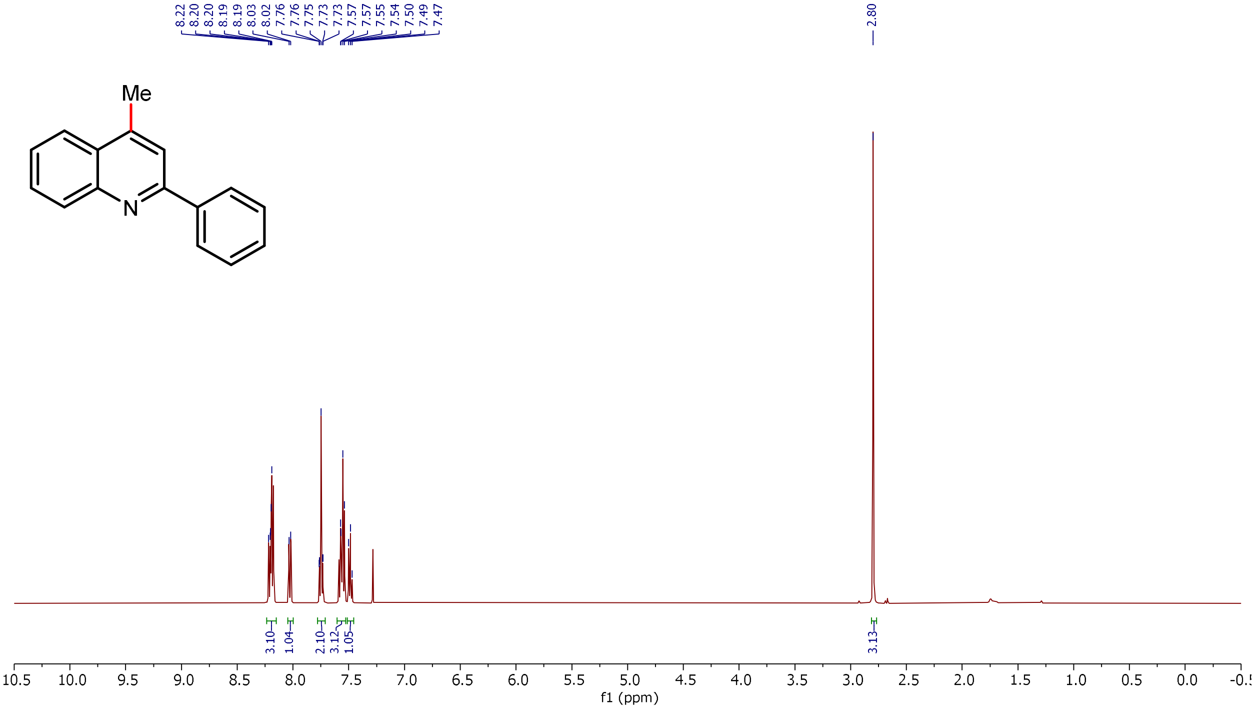


**Supplementary Figure 27b | $^{13}$C NMR (125 MHz, $CDCl_3$) of 4-methyl-2-phenylquinoline (21)**

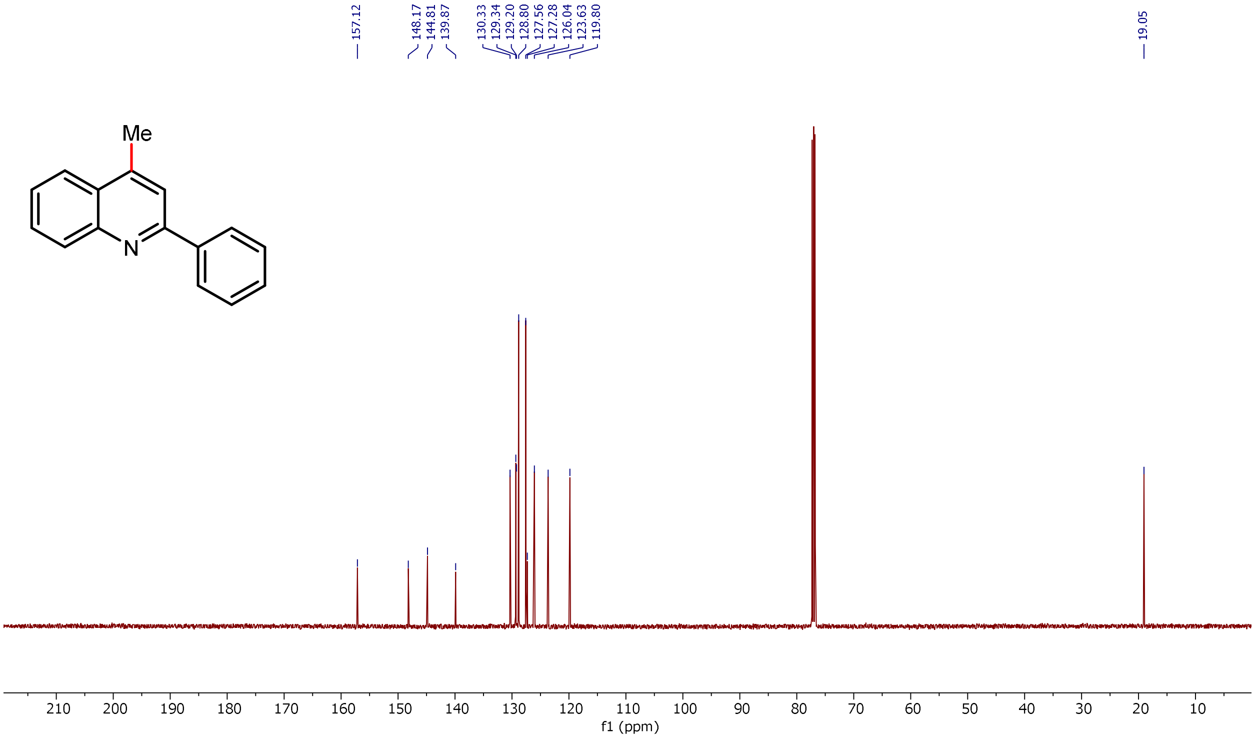

**Supplementary Figure 28a | $^1$H NMR (500 MHz, $CDCl_3$) of 4-(methyl-$d_3$)-2-phenylquinoline (22)**

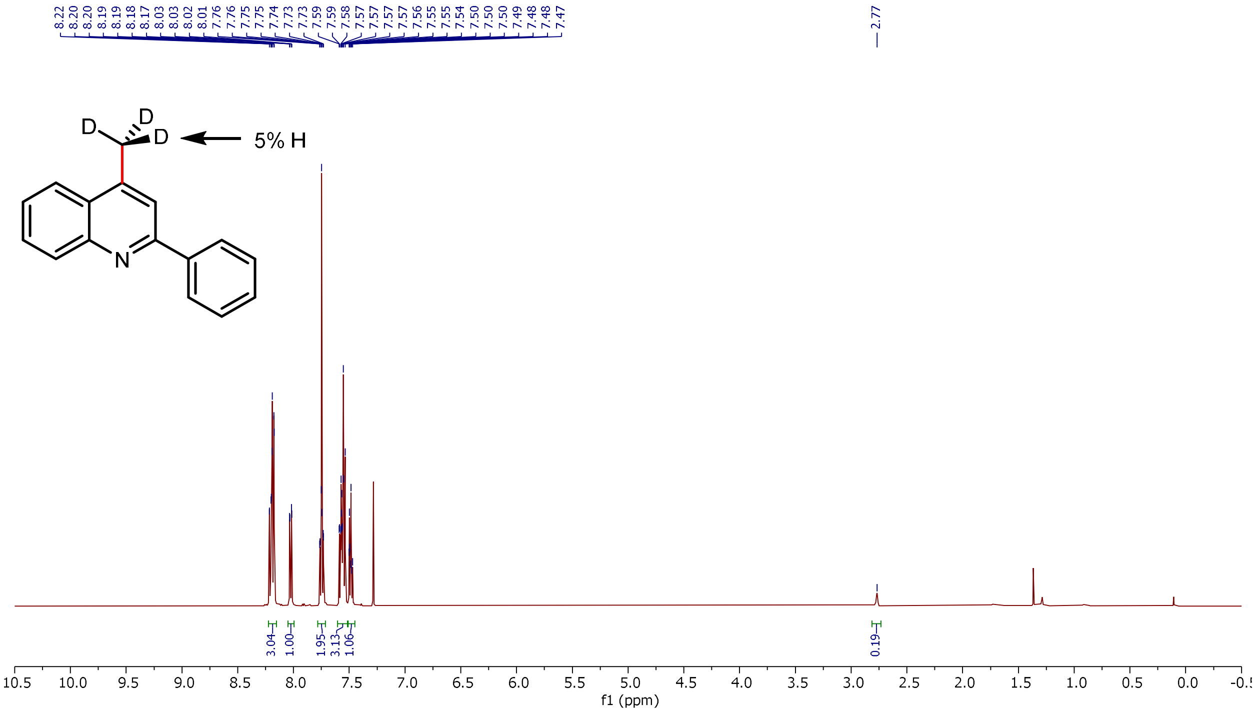


**Supplementary Figure 28b | $^{13}$C NMR (125 MHz, $CDCl_3$) of 4-(methyl-$d_3$)-2-phenylquinoline (22)**

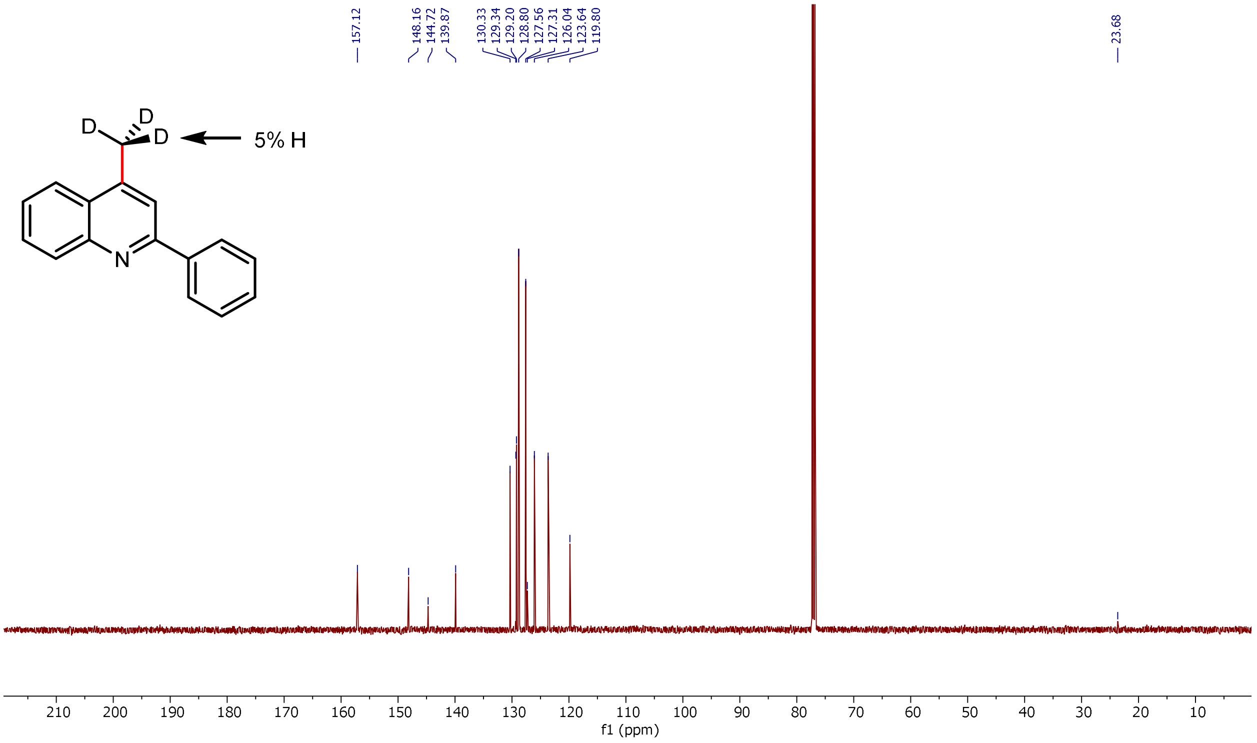

**Supplementary Figure 28c | $^2$H NMR (77 MHz, $CDCl_3$) of 4-(methyl-$d_3$)-2-phenylquinoline (22)**

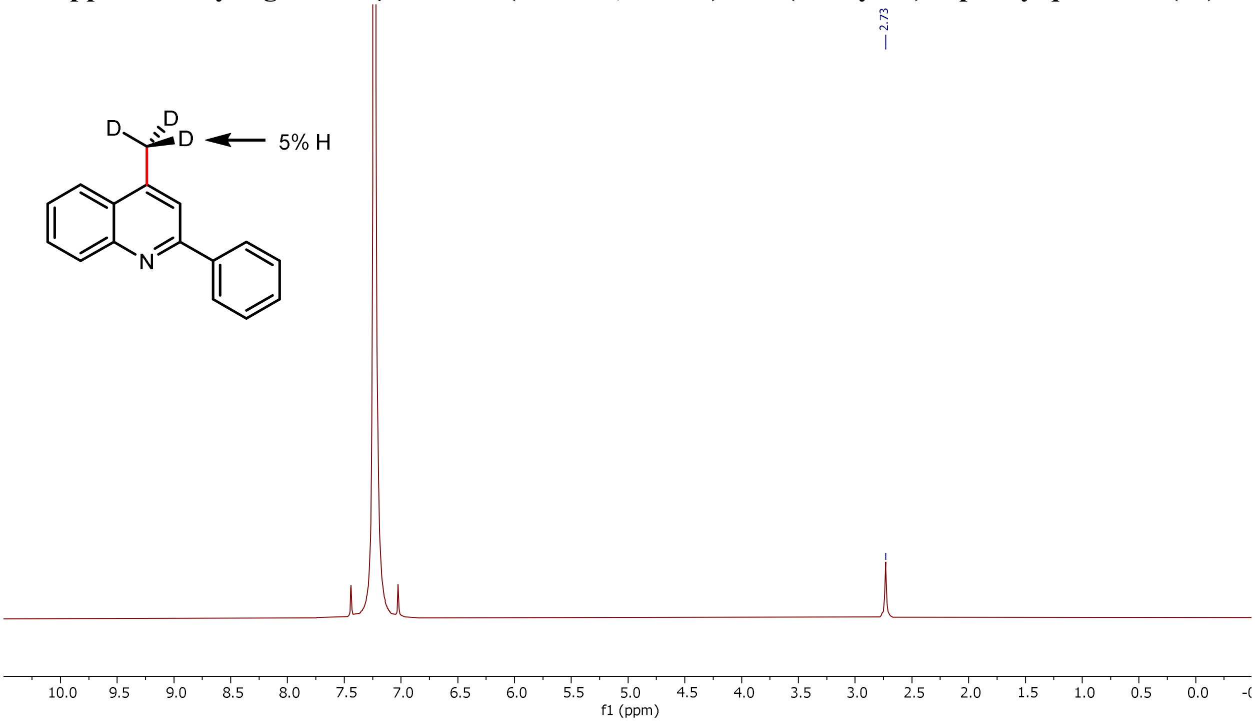

**Supplementary Figure 29a | $^{1}$H NMR (500 MHz, $CDCl_3$) of 4-(1,4-dioxan-2-yl)-2-phenylquinoline (23)**

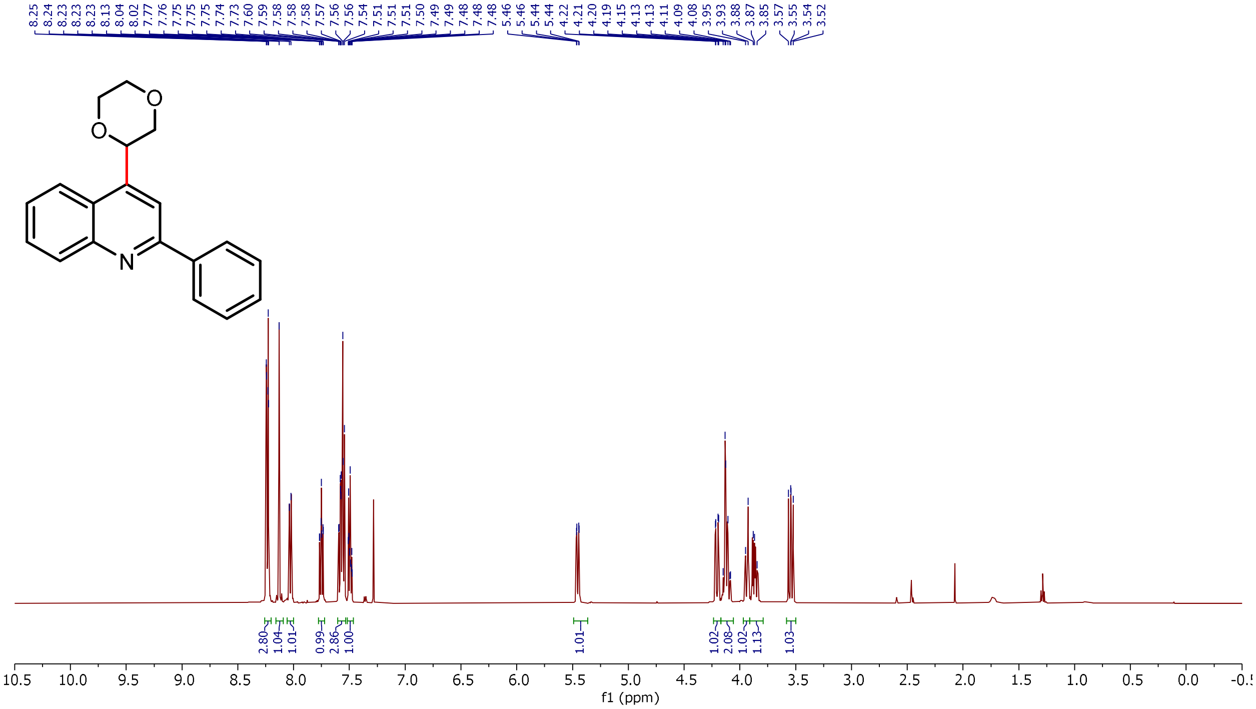


**Supplementary Figure 29b | $^{13}$C NMR (125 MHz, $CDCl_3$) of 4-(1,4-dioxan-2-yl)-2-phenylquinoline (23)**

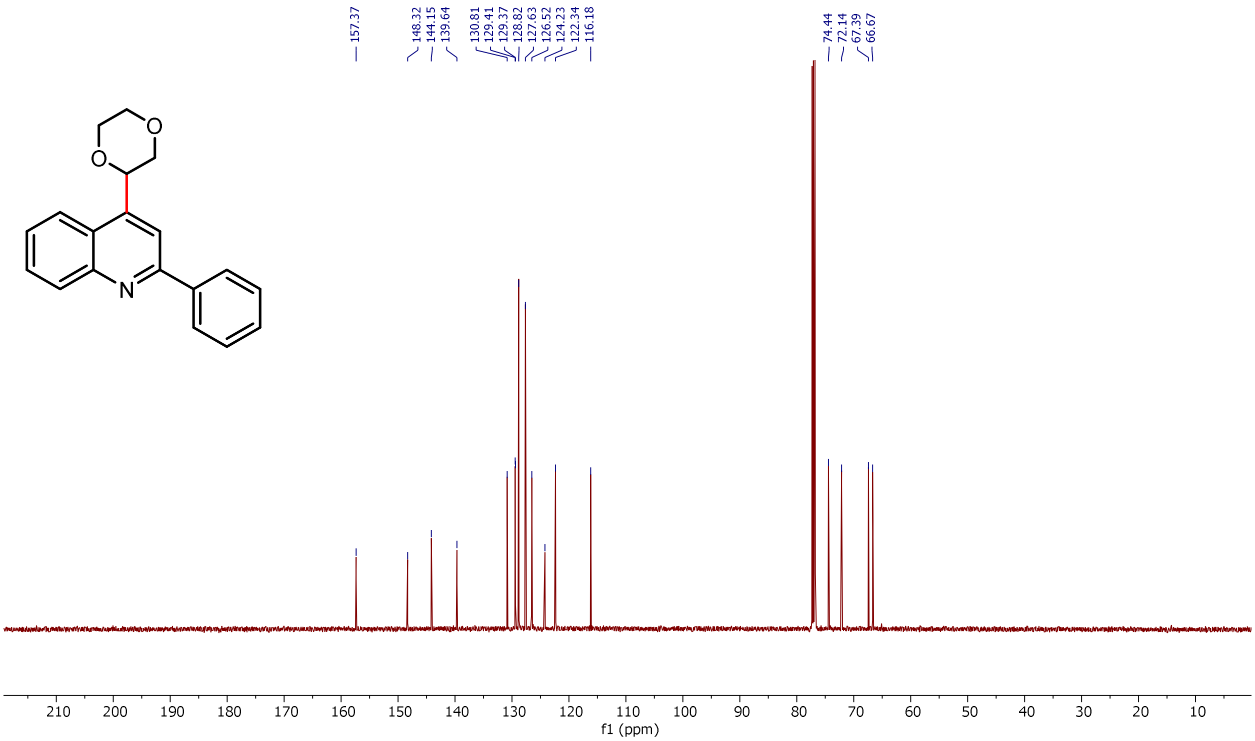

**Supplementary Figure 30a | $^{1}$H NMR (500 MHz, $CDCl_3$) of 2-phenyl-4-(tetrahydro-2*H*-pyran-2-yl)quinoline (24)**

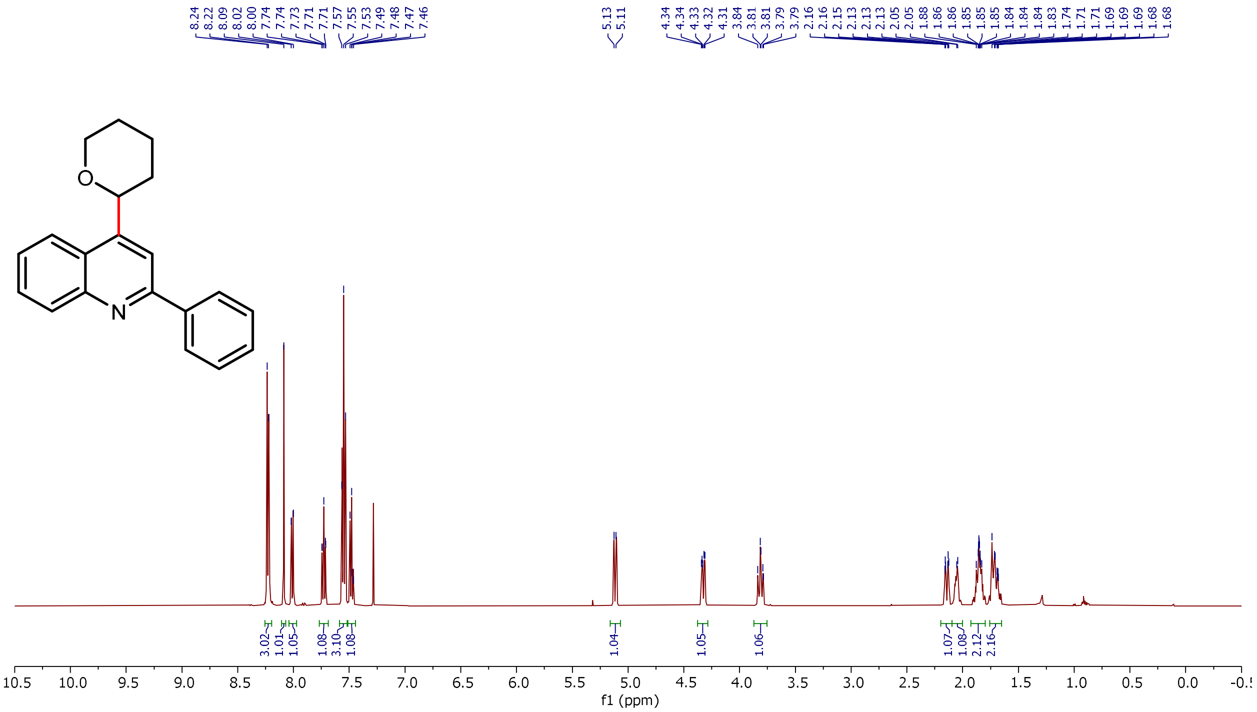

**Supplementary Figure 30b | $^{13}$C NMR (125 MHz, $CDCl_3$) of 2-phenyl-4-(tetrahydro-2*H*-pyran-2-yl)quinoline (24)**

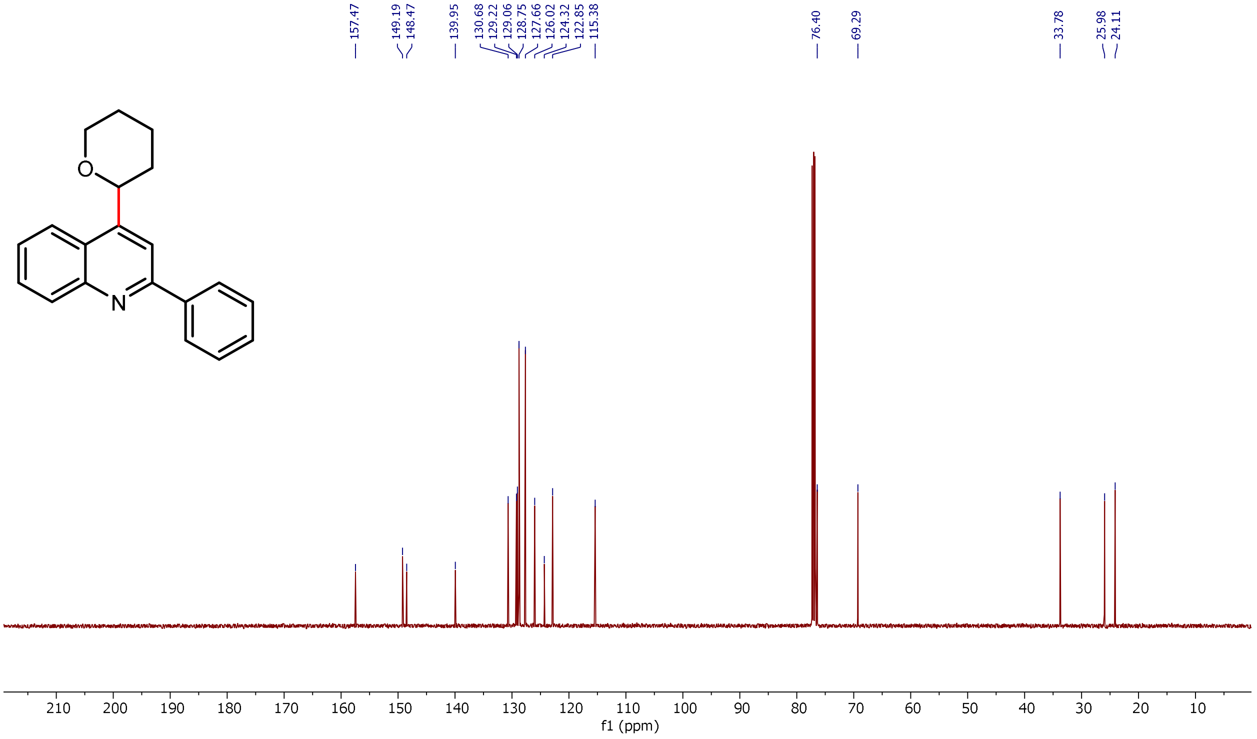

**Supplementary Figure 31a | $^{1}$H NMR (500 MHz, $CDCl_3$) of methyl 2-(*trans*-2-phenylquinolin-4-yl)tetrahydro-2*H*-pyran-4-carboxylate (25)**

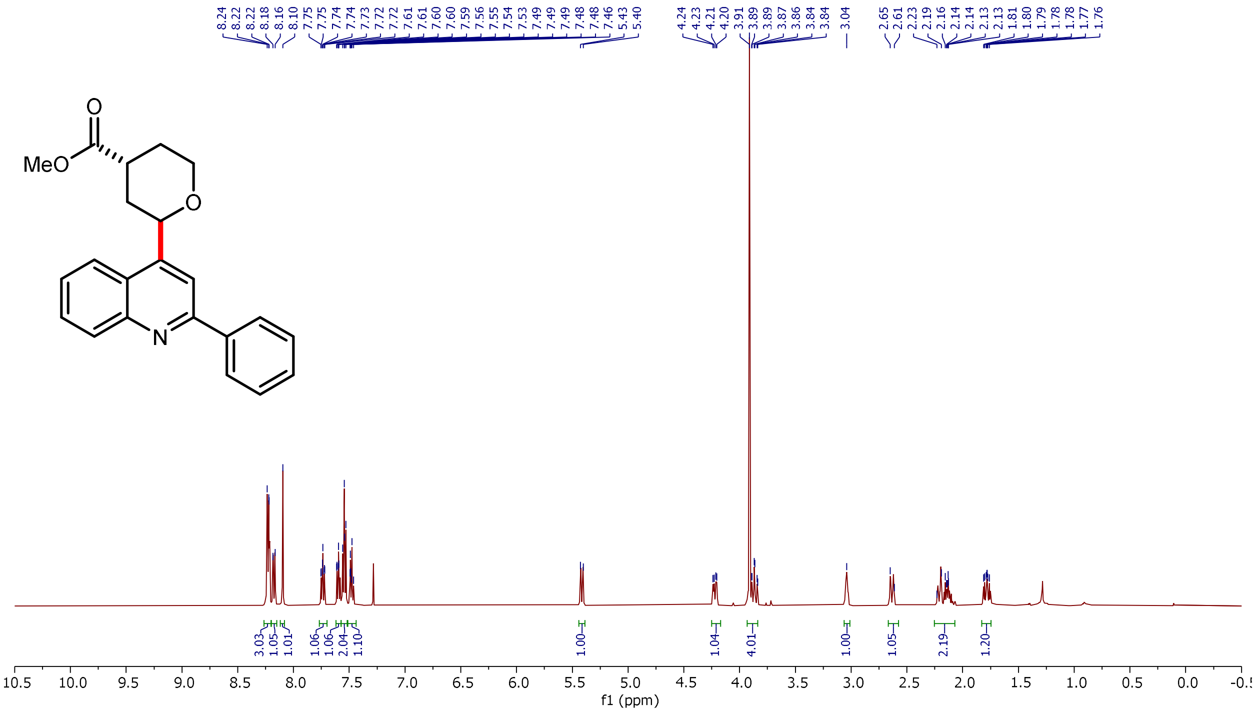


**Supplementary Figure 31b | $^{13}$C NMR (125 MHz, $CDCl_3$) of methyl 2-(*trans*-2-phenylquinolin-4-yl)tetrahydro-2*H*-pyran-4-carboxylate (25)**

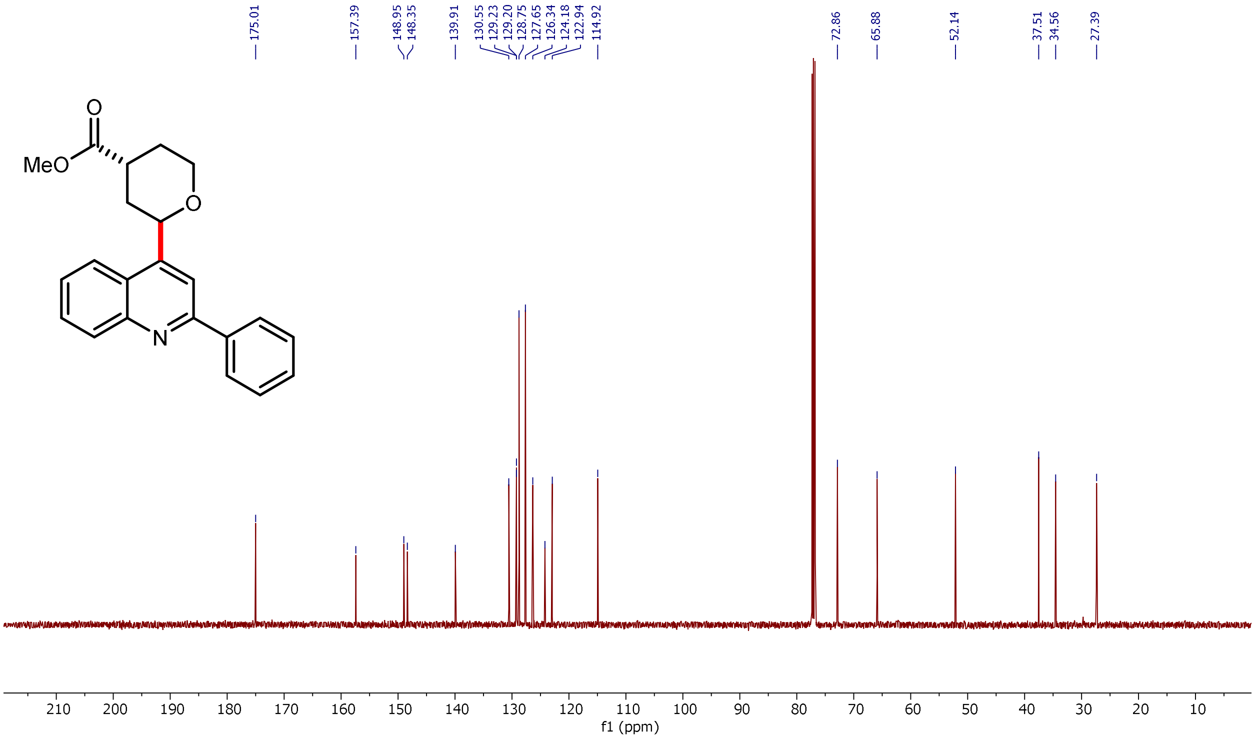

**Supplementary Figure 32a | $^{1}$H NMR (500 MHz, $CDCl_3$) of 2-(2-phenylquinolin-4-yl)tetrahydro-4*H*-pyran-4-one (26)**

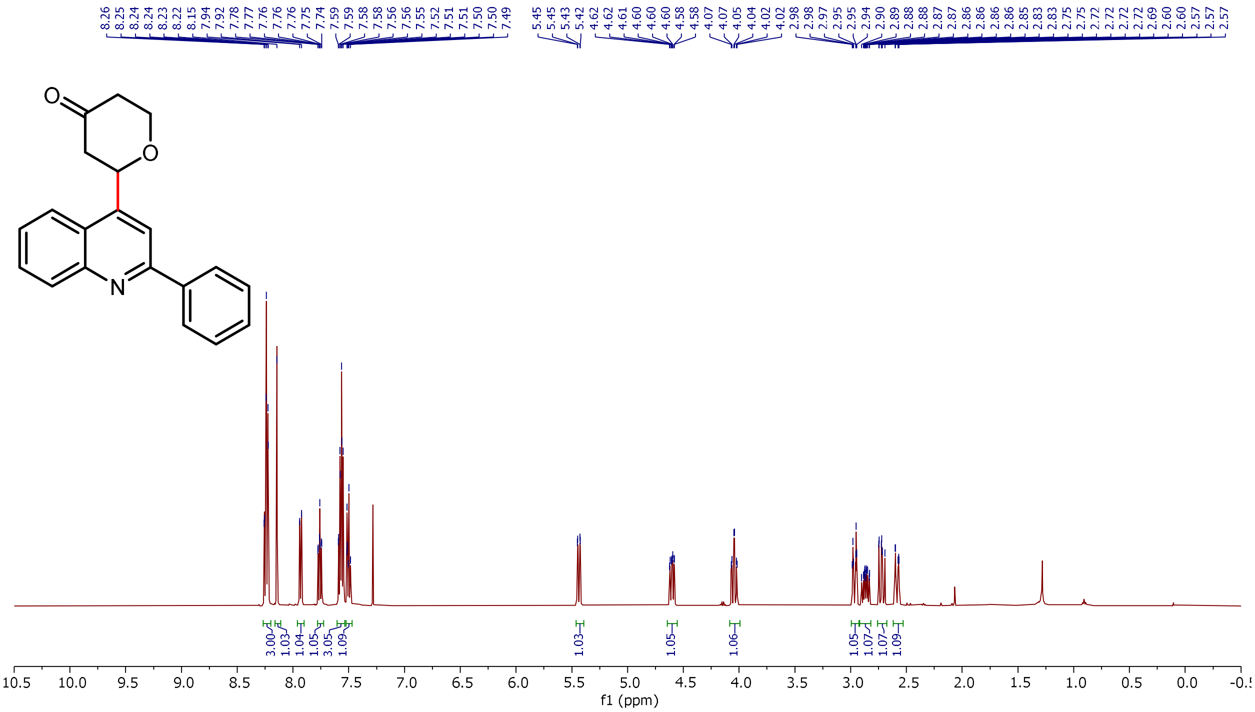


**Supplementary Figure 32b | $^{13}$C NMR (125 MHz, $CDCl_3$) of 2-(2-phenylquinolin-4-yl)tetrahydro-4*H*-pyran-4-one (26)**

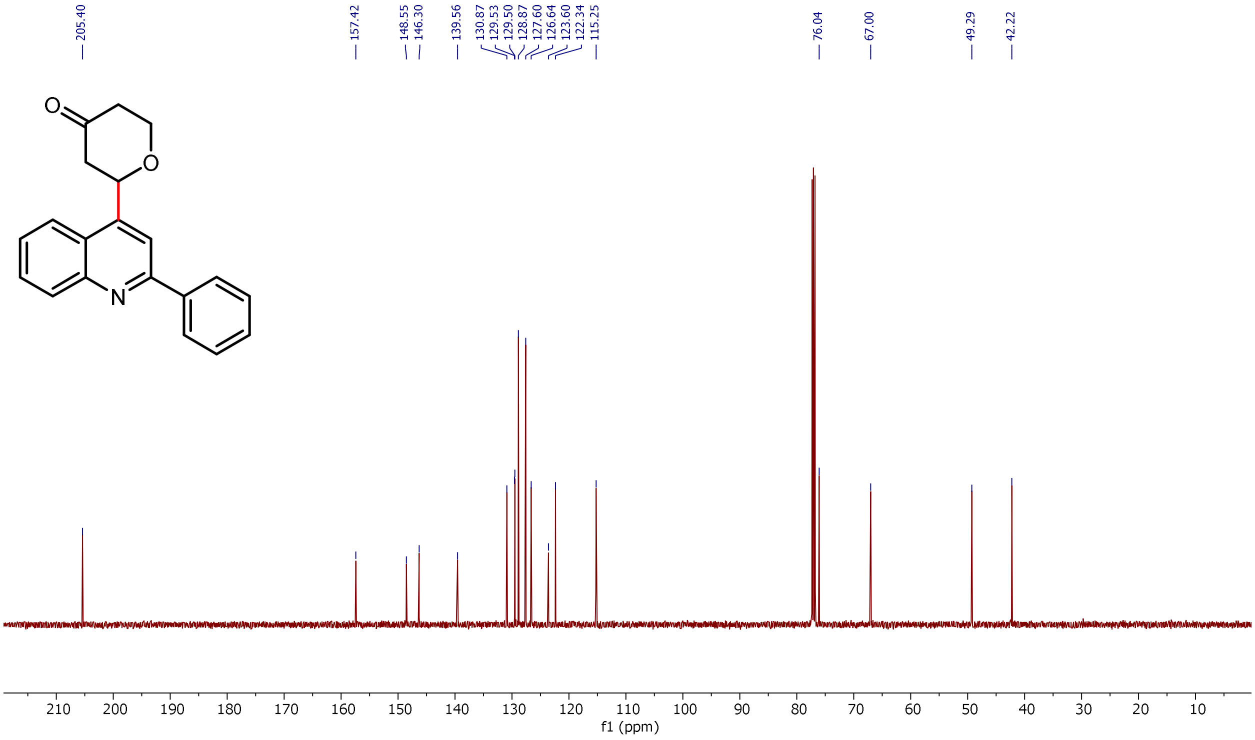

**Supplementary Figure 33a | $^{1}$H NMR (500 MHz, $CDCl_3$) of 1-(2-(2-phenylquinolin-4-yl)pyrrolidin-1-yl)ethan-1-one, 1:0.65 rotamers (27)**

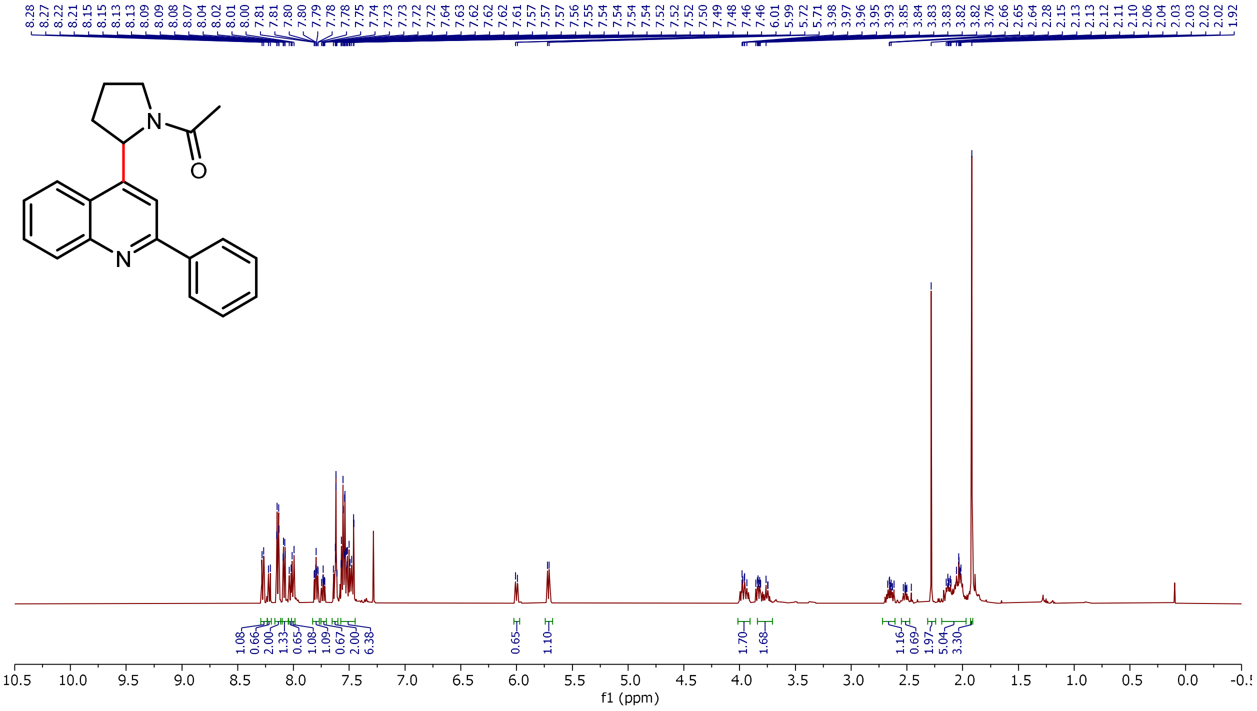


**Supplementary Figure 33b | $^{13}$C NMR (125 MHz, $CDCl_3$) of 1-(2-(2-phenylquinolin-4-yl)pyrrolidin-1-yl)ethan-1-one, 1:0.65 rotamers (27)**

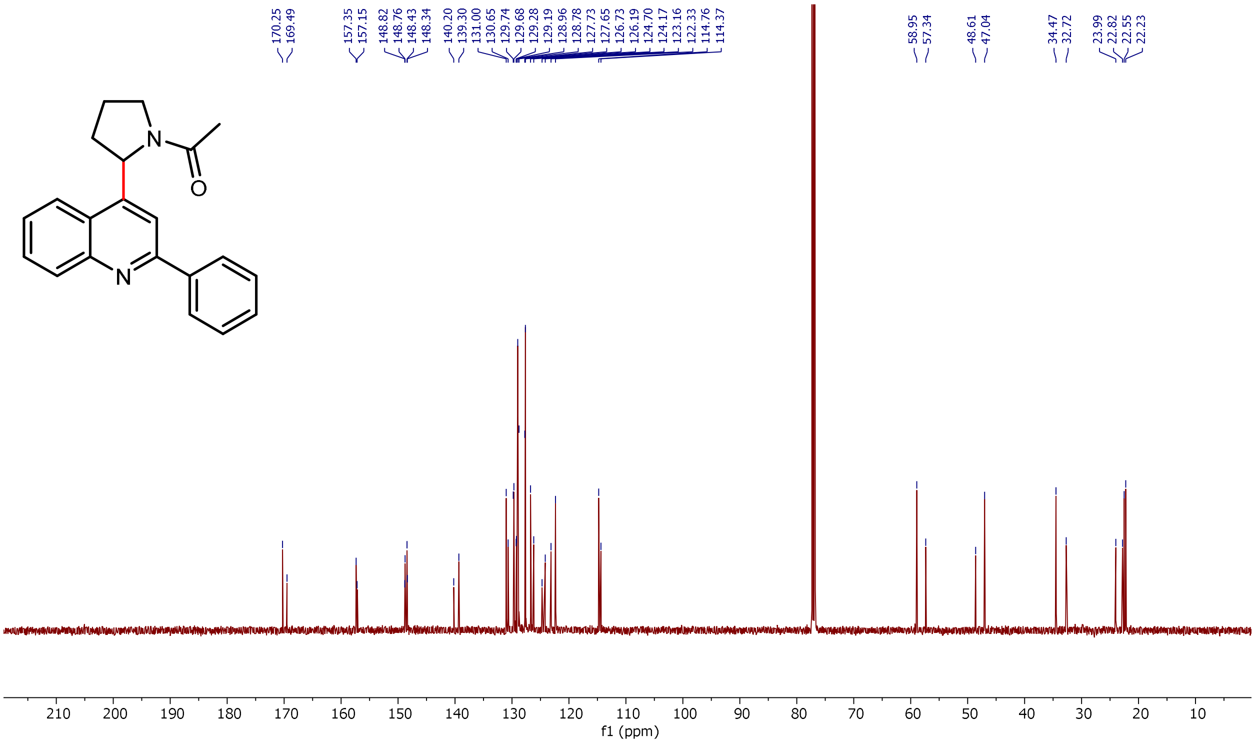

**Supplementary Figure 34a | $^{1}$H NMR (500 MHz, CDCl$_3$) of 5-(2-phenylquinolin-4-yl)pyrrolidin-2-one (28)**

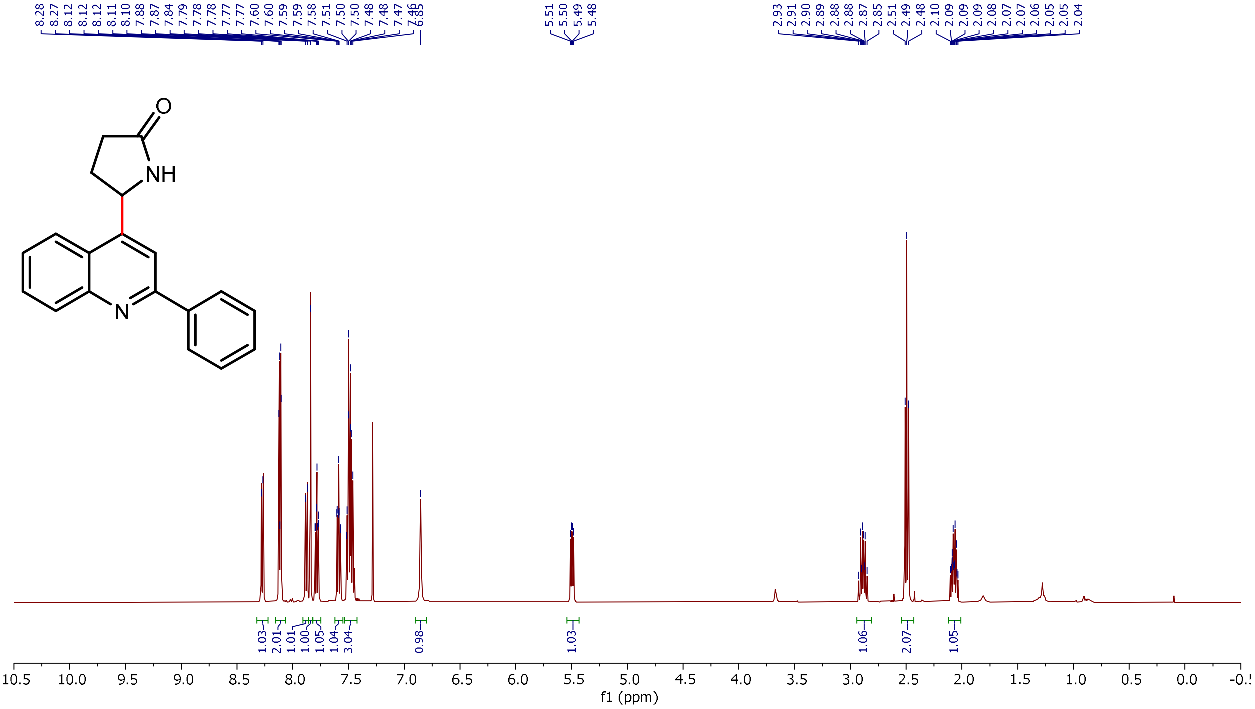


**Supplementary Figure 34b | $^{13}$C NMR (125 MHz, CDCl$_3$) of 5-(2-phenylquinolin-4-yl)pyrrolidin-2-one (28)**

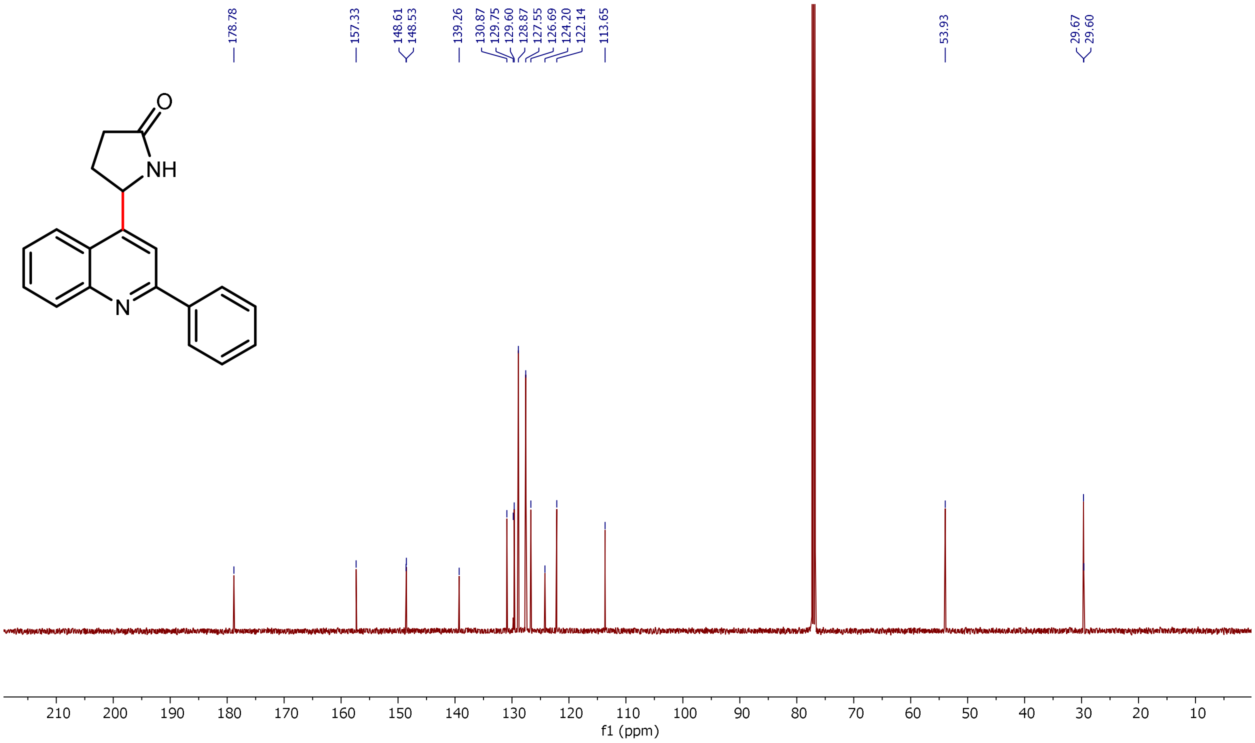

**Supplementary Figure 35a | $^{1}$H NMR (500 MHz, $CDCl_3$) of *N*-methyl-*N*-((2-phenylquinolin-4-yl)methyl)formamide, 1:0.5 rotamers (29)**

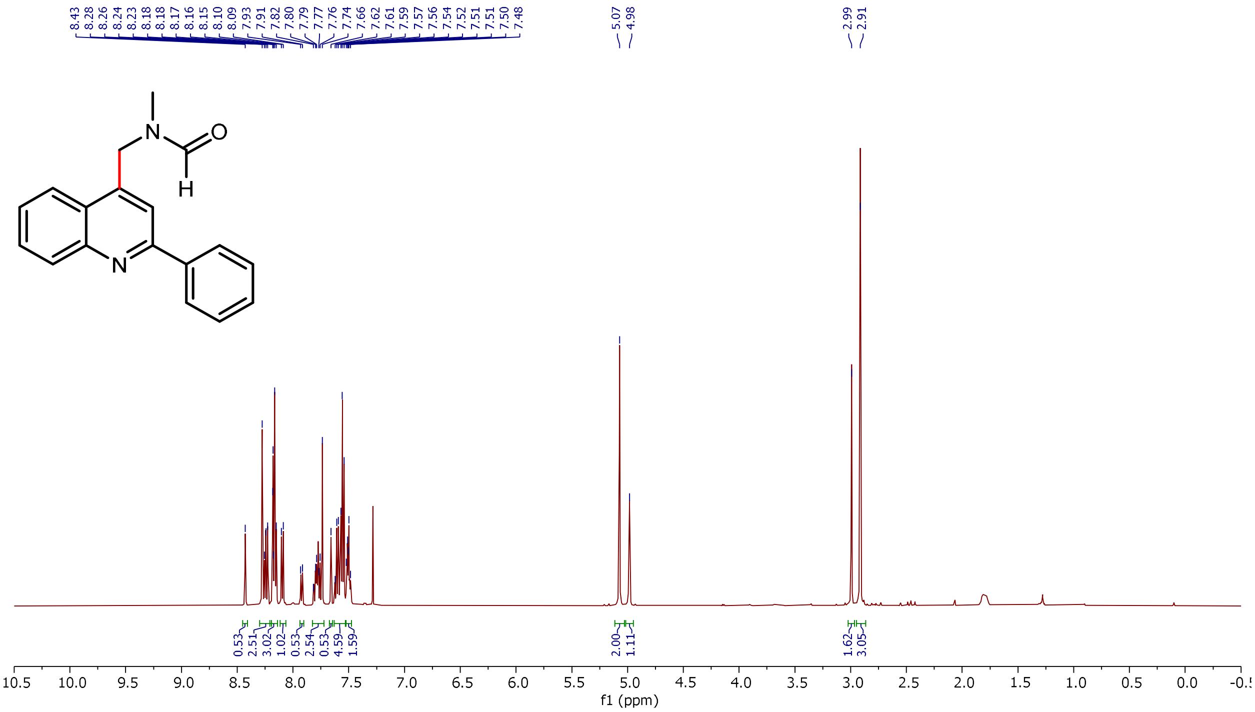


**Supplementary Figure 35b | $^{13}$C NMR (125 MHz, $CDCl_3$) of *N*-methyl-*N*-((2-phenylquinolin-4-yl)methyl)formamide, 1:0.5 rotamers (29)**

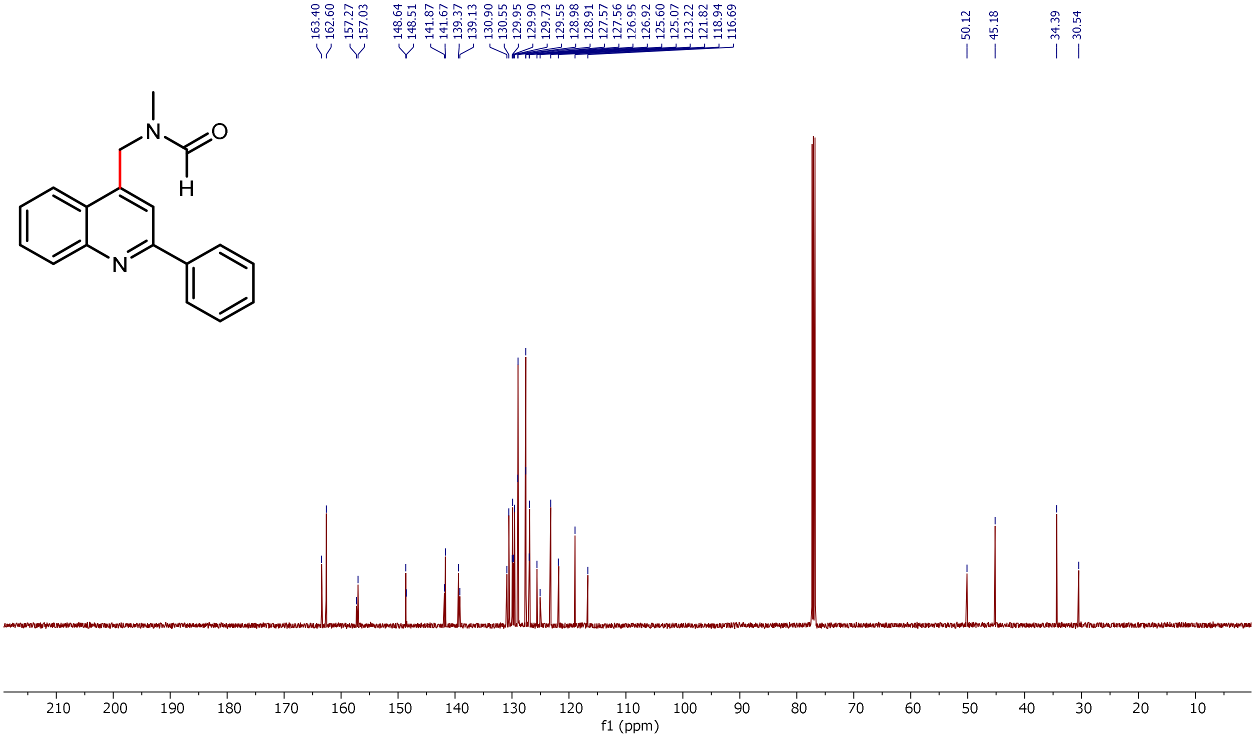

**Supplementary Figure 36a | $^{1}$H NMR (500 MHz, $CDCl_3$) of *N*-methyl-*N*-((2-phenylquinolin-4-yl)methyl)acetamide, 1:0.5 rotamers (30)**

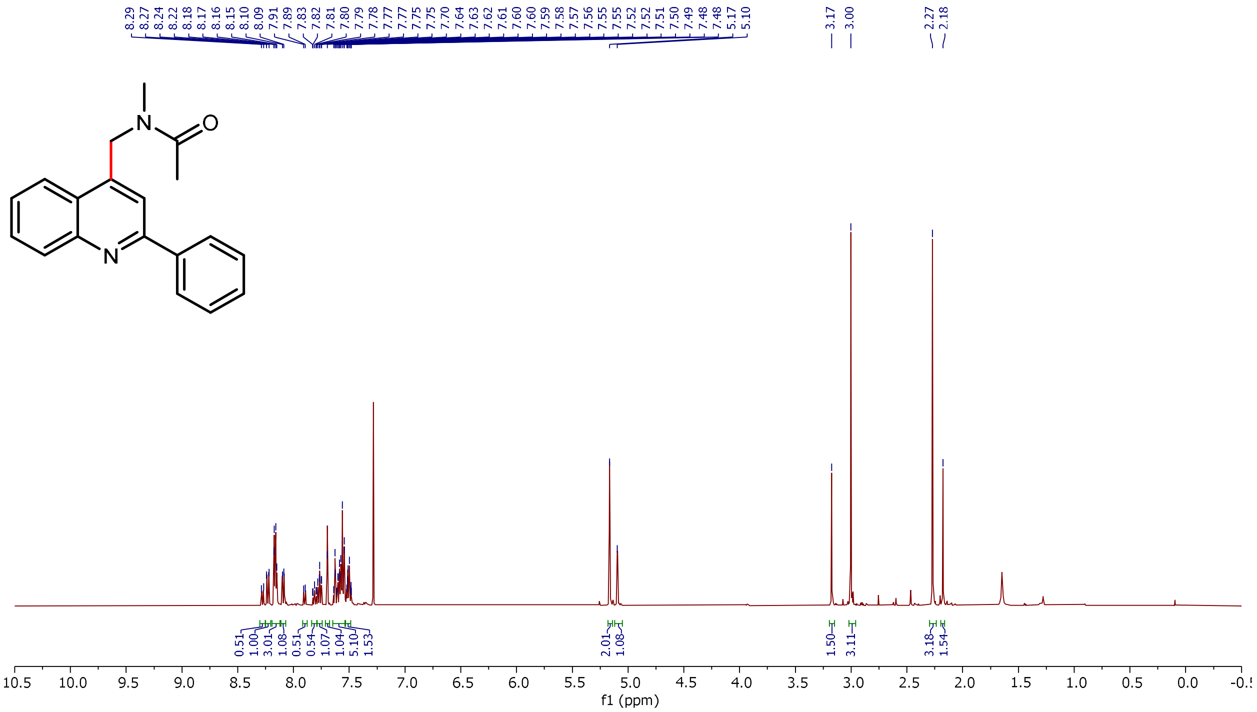


**Supplementary Figure 36b | $^{13}$C NMR (125 MHz, $CDCl_3$) of *N*-methyl-*N*-((2-phenylquinolin-4-yl)methyl)acetamide, 1:0.5 rotamers (30)**

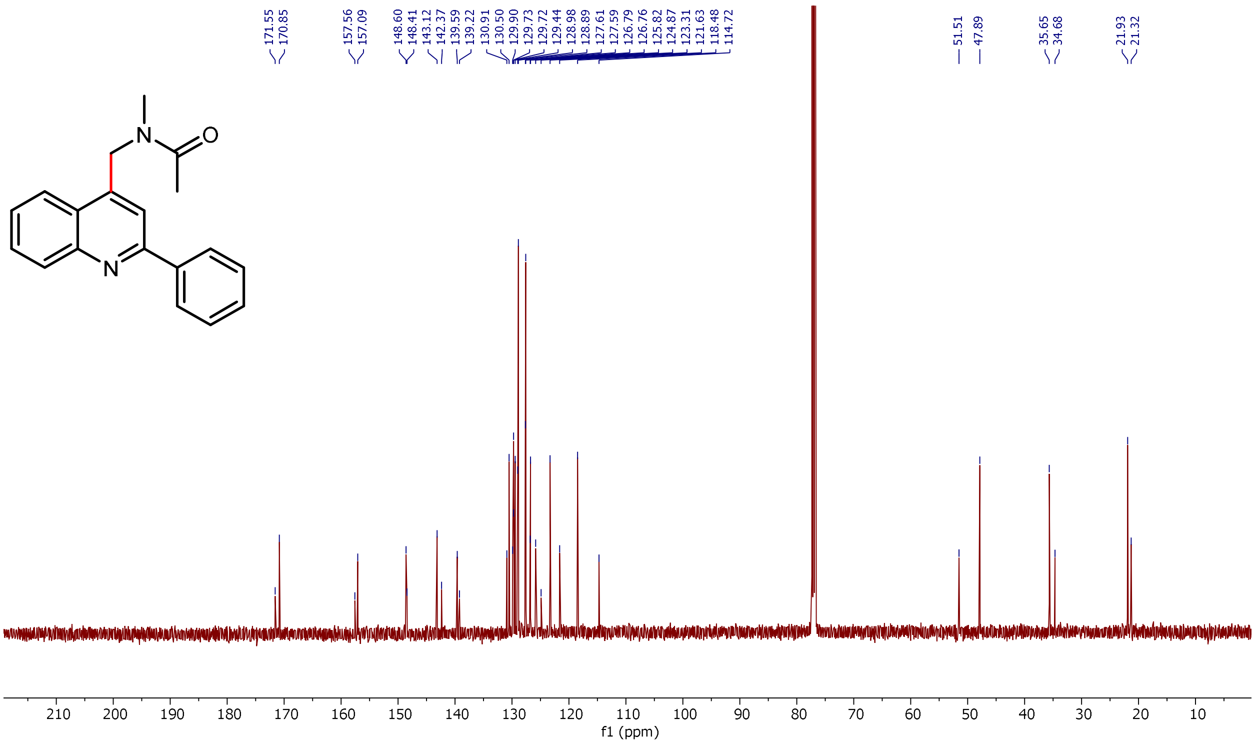

**Supplementary Figure 37a | $^{1}$H NMR (500 MHz, $CDCl_3$) of 1-methyl-5-(2-phenylquinolin-4-yl)pyrrolidin-2-one (31a) and 1-((2-phenylquinolin-4-yl)methyl)pyrrolidin-2-one (31b), 1:0.26 congeners (better purity could not be obtained)**

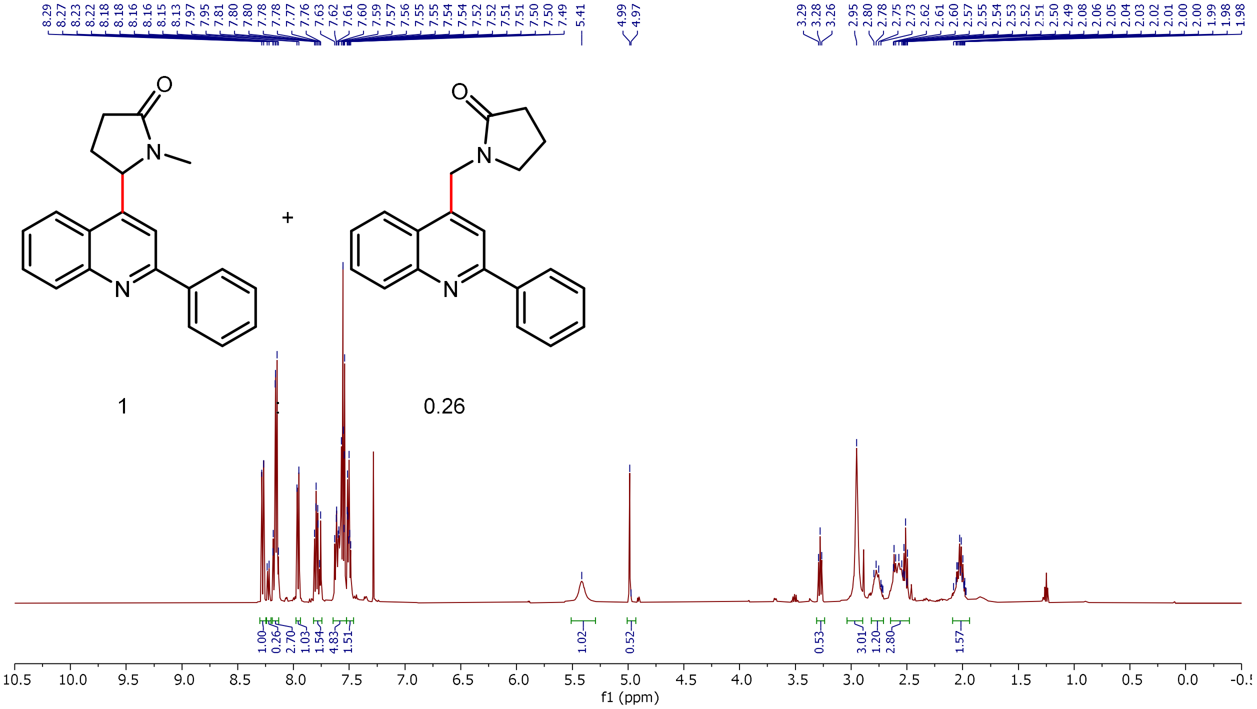


**Supplementary Figure 37b | $^{13}$C NMR (125 MHz, $CDCl_3$) of 1-methyl-5-(2-phenylquinolin-4-yl)pyrrolidin-2-one (31a) and 1-((2-phenylquinolin-4-yl)methyl)pyrrolidin-2-one (31b), 1:0.26 congeners (better purity could not be obtained)**

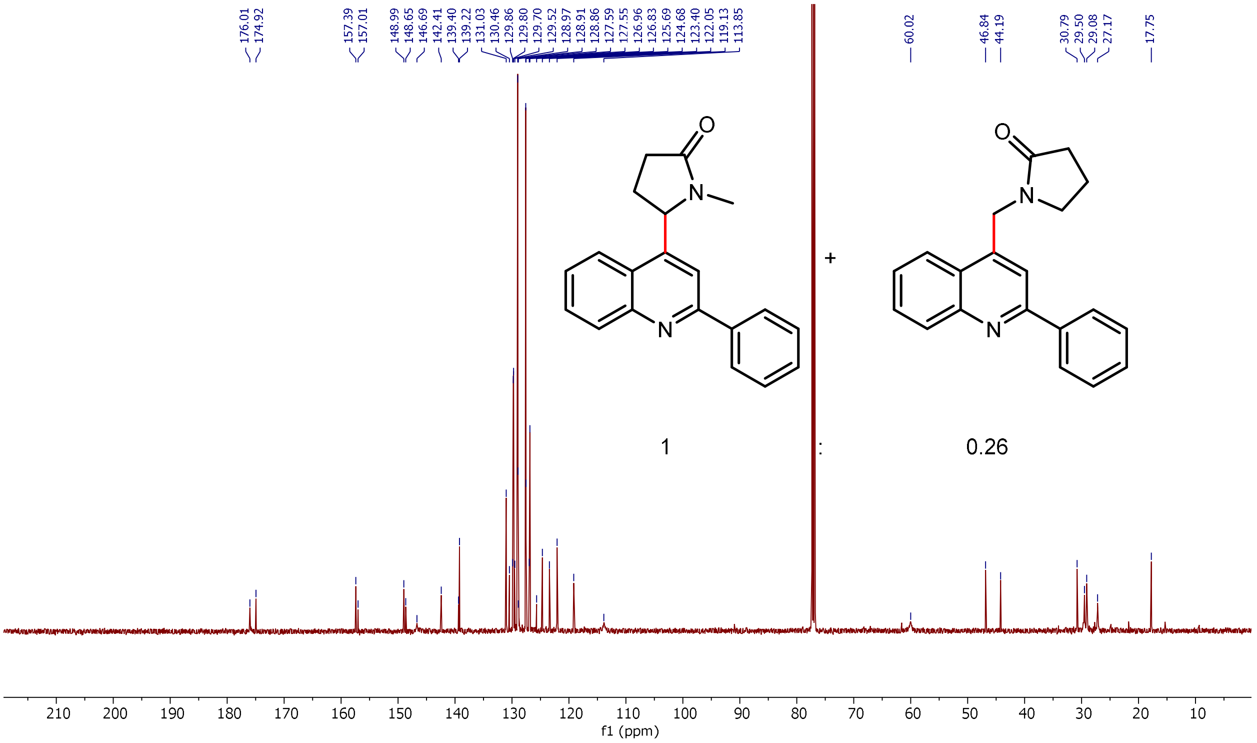

**Supplementary Figure 38a | $^{1}$H NMR (500 MHz, $CDCl_3$) of *N*-((2-phenylquinolin-4-yl)methyl)methanesulfonamide (32)**

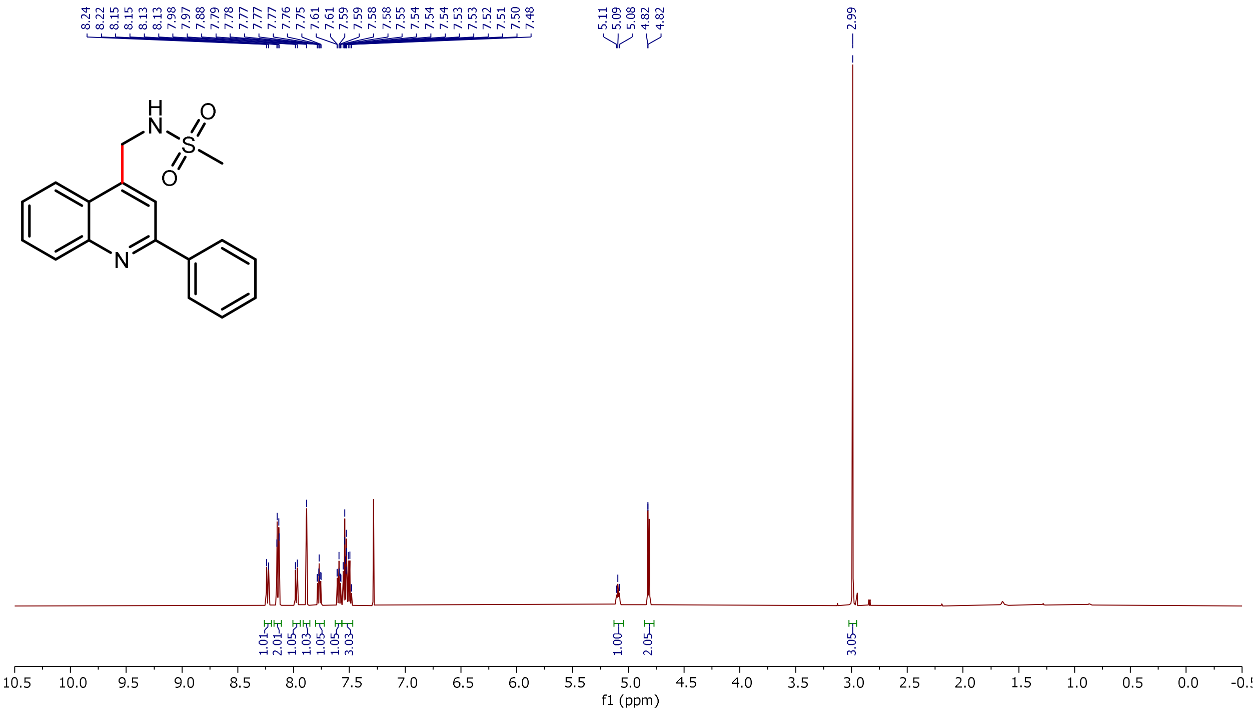


**Supplementary Figure 38b | $^{13}$C NMR (125 MHz, $CDCl_3$) of *N*-((2-phenylquinolin-4-yl)methyl)methanesulfonamide (32)**

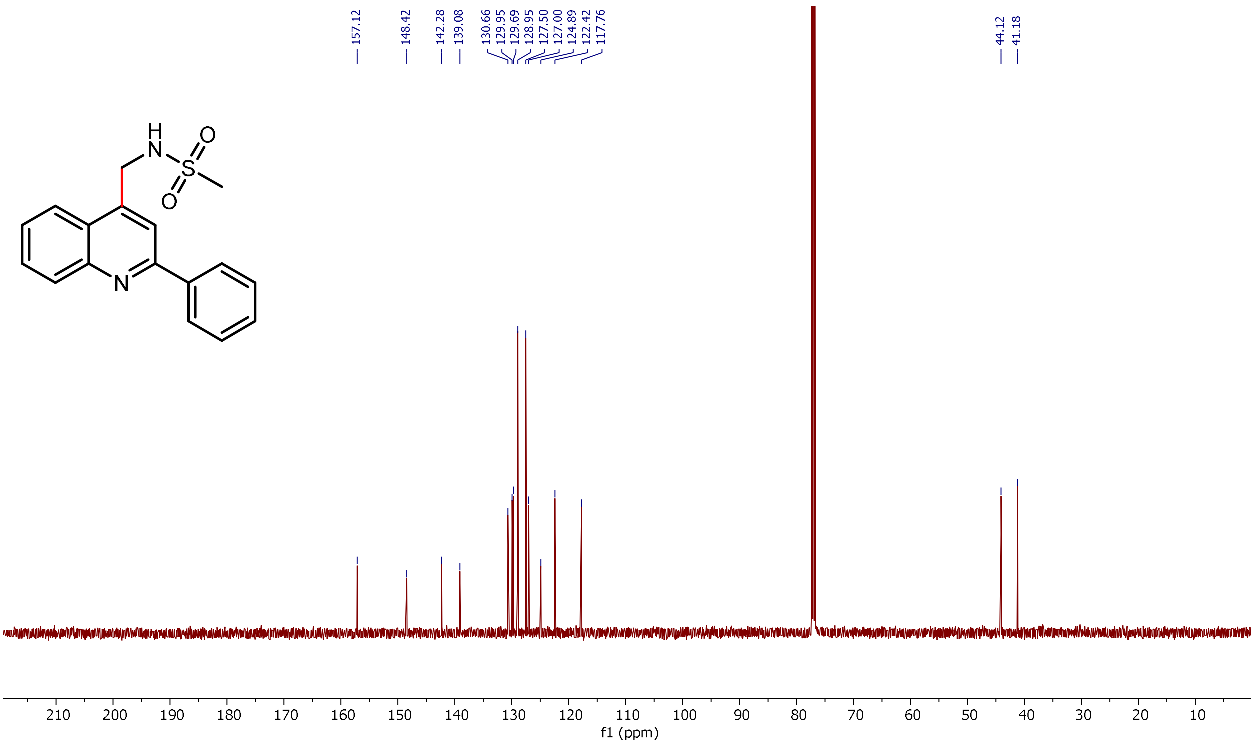

**Supplementary Figure 39a | $^{1}$H NMR (500 MHz, $CDCl_3$) of *N*, *N*', *N*',*N*'',*N*''-pentamethyl-*N*-((2-phenylquinolin-4-yl)methyl)phosphoramide (33)**

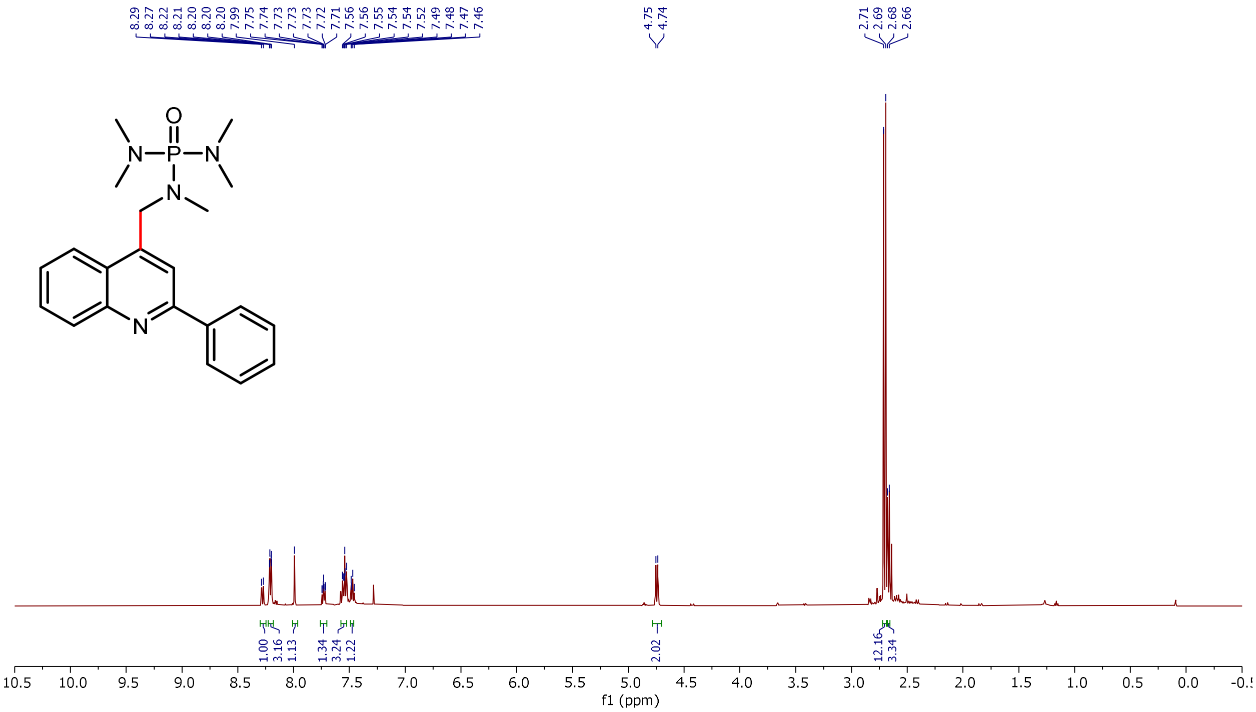


**Supplementary Figure 39b | $^{13}$C NMR (125 MHz, $CDCl_3$) of *N*, *N*', *N*',*N*'',*N*''-pentamethyl-*N*-((2-phenylquinolin-4-yl)methyl)phosphoramide (33)**

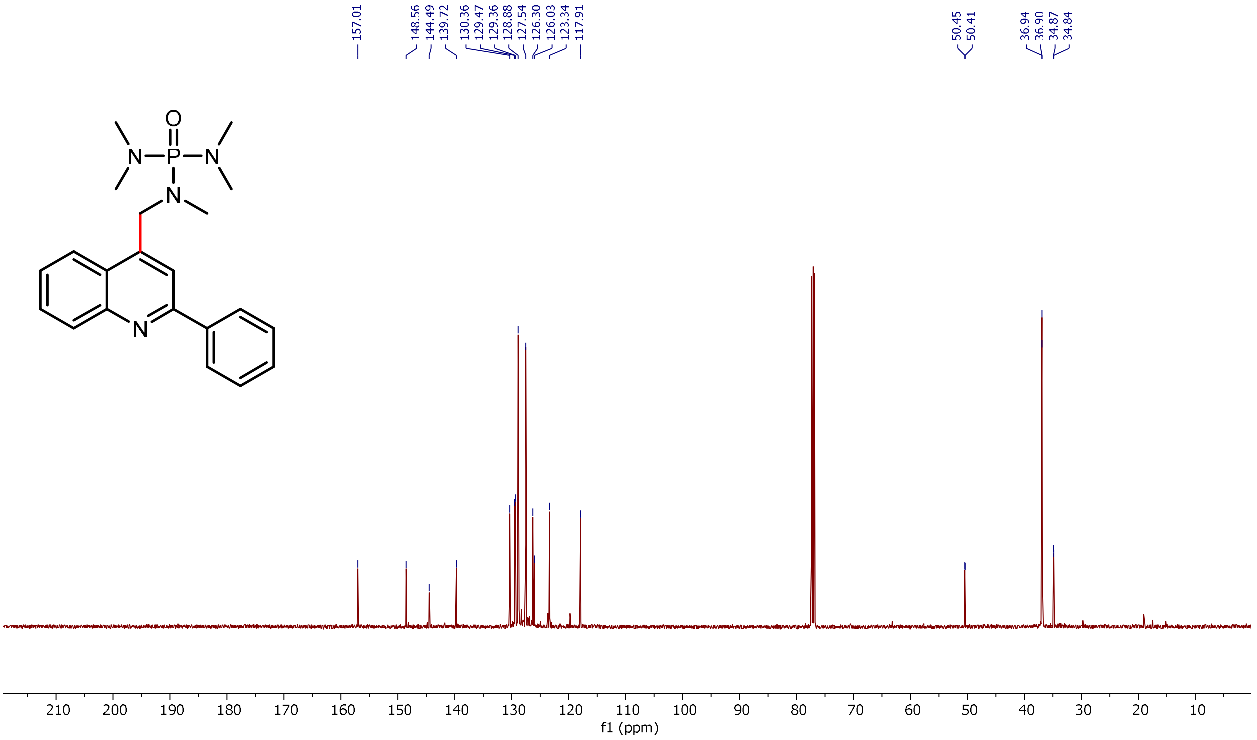

**Supplementary Figure 39c | $^{31}$P NMR (203 MHz, CDCl$_3$) of *N*, *N'*, *N'*,*N''*,*N''*-pentamethyl-*N*-((2-phenylquinolin-4-yl)methyl)phosphoramide (33)**

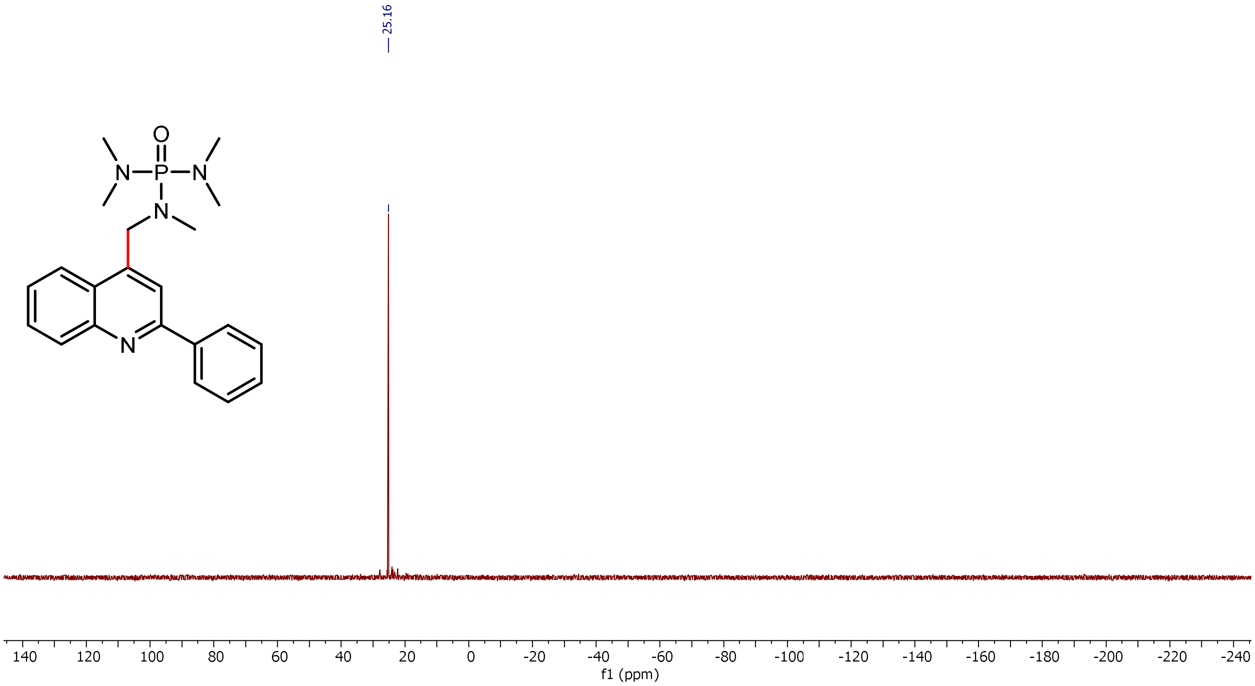

**Supplementary Figure 40a | $^{1}$H NMR (500 MHz, $CDCl_3$) of 2-phenylquinoline-4-carboxamide (34)**

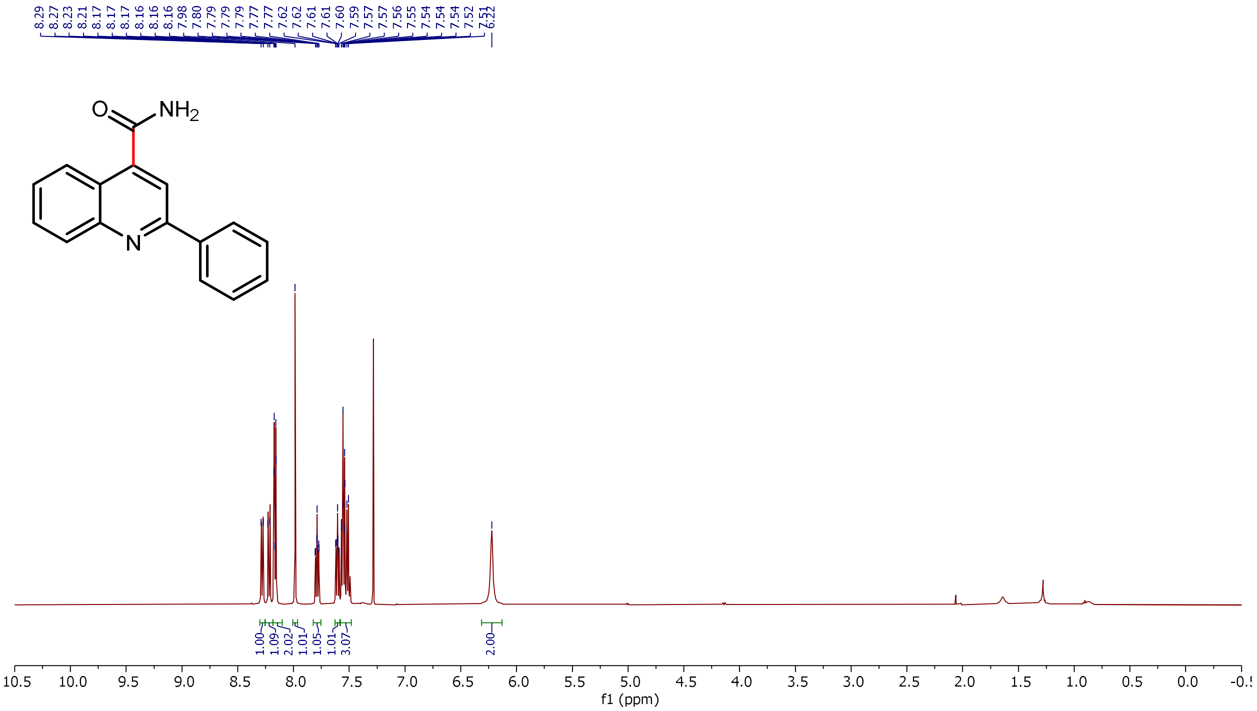

**Supplementary Figure 40b | $^{13}$C NMR (125 MHz, $CDCl_3$) of 2-phenylquinoline-4-carboxamide (34)**

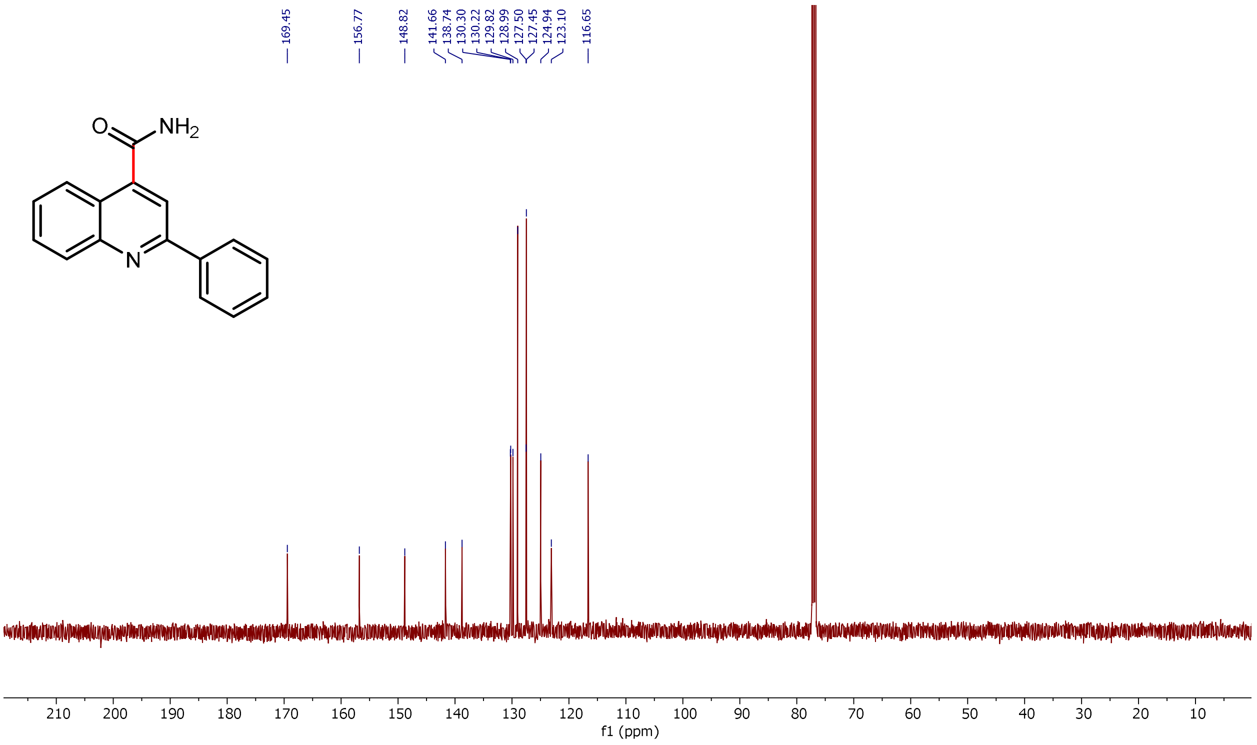

**Supplementary Figure 41a | $^{1}$H NMR (500 MHz, $CDCl_3$) of 4-cyclohexyl-2-(4-fluorophenyl)quinoline (35)**

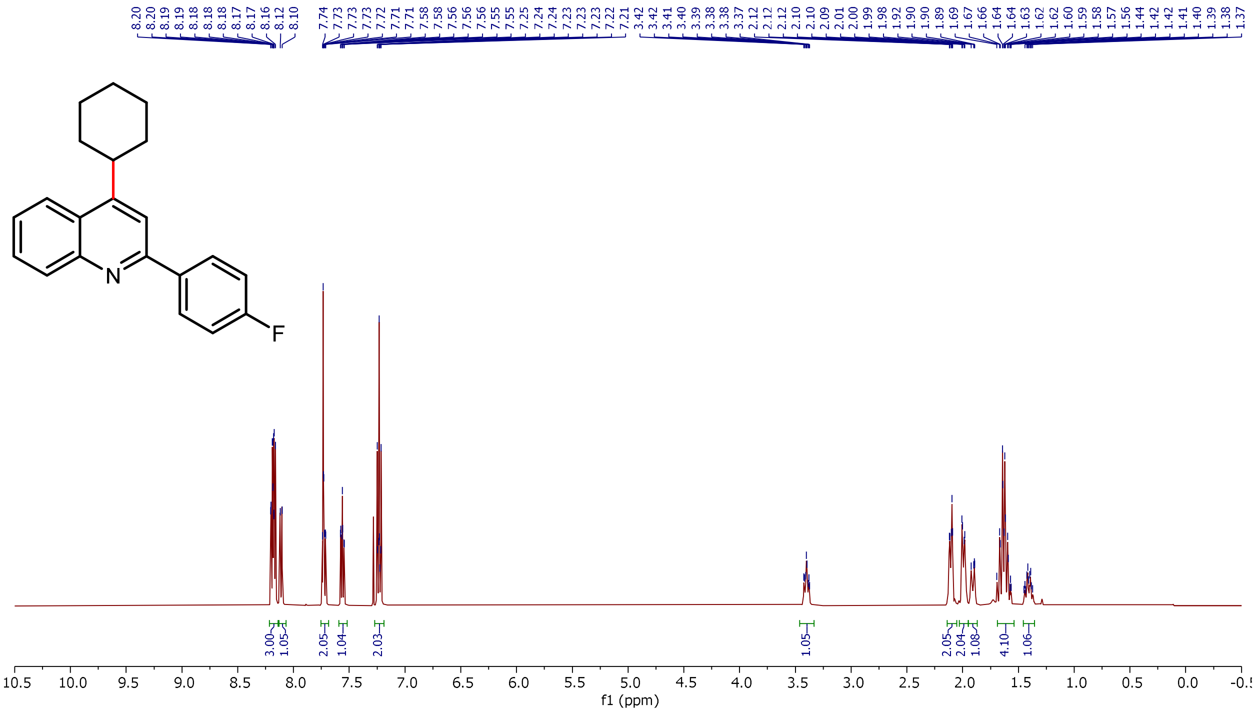

**Supplementary Figure 41b | $^{13}$C NMR (125 MHz, $CDCl_3$) of 4-cyclohexyl-2-(4-fluorophenyl)quinoline (35)**

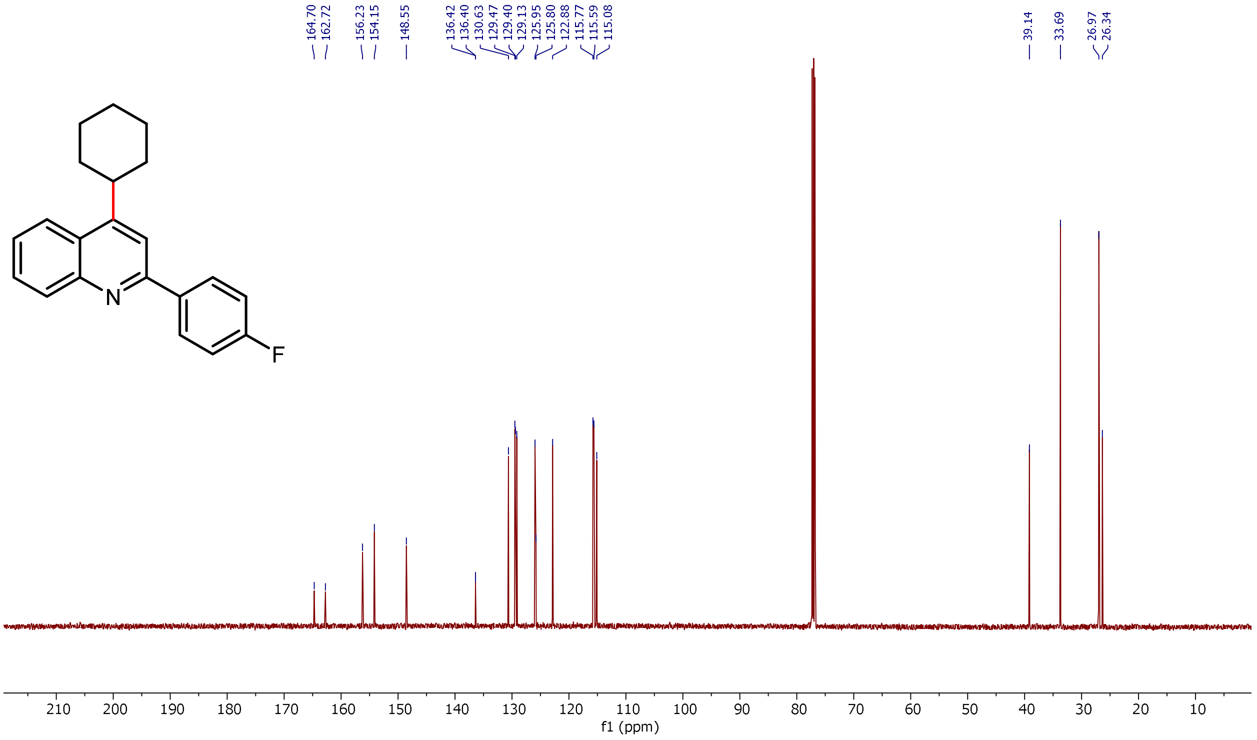

**Supplementary Figure 41c | $^{19}$F NMR (470 MHz, $CDCl_3$) of 4-cyclohexyl-2-(4-fluorophenyl)quinoline (35)**

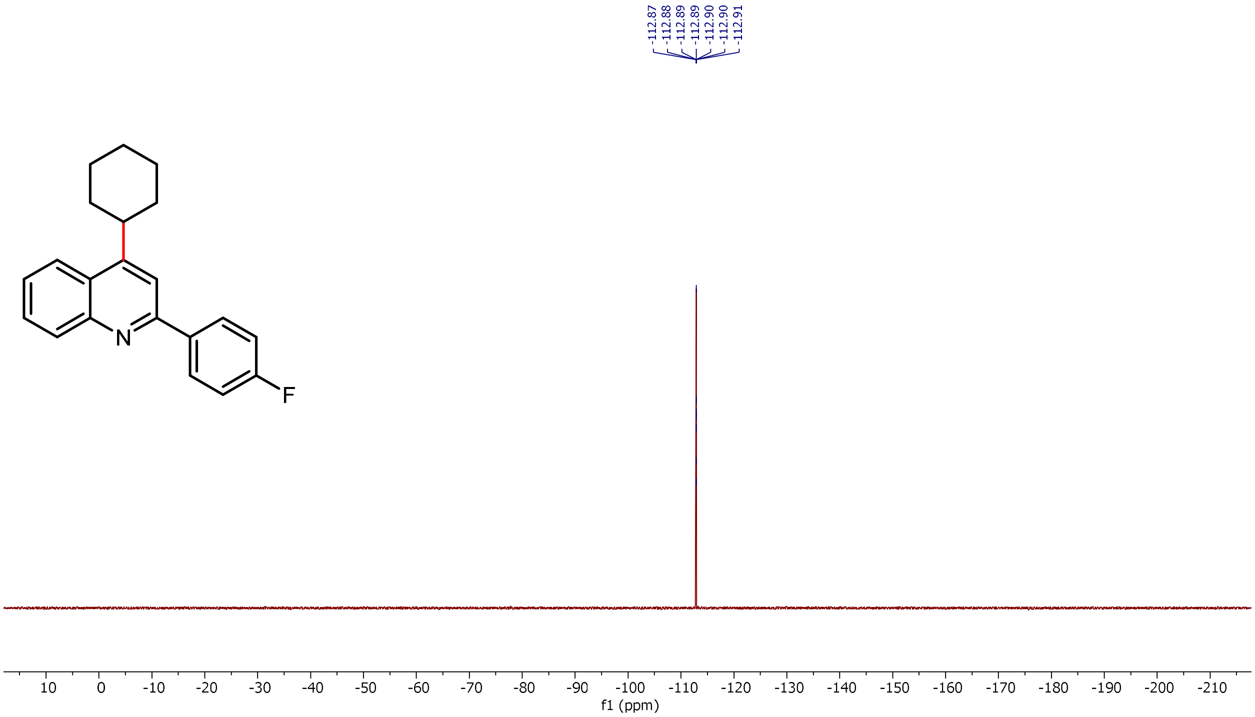

**Supplementary Figure 42a | $^1$H NMR (500 MHz, $CDCl_3$) of 2-(4-chlorophenyl)-4-cyclohexylquinoline (36)**

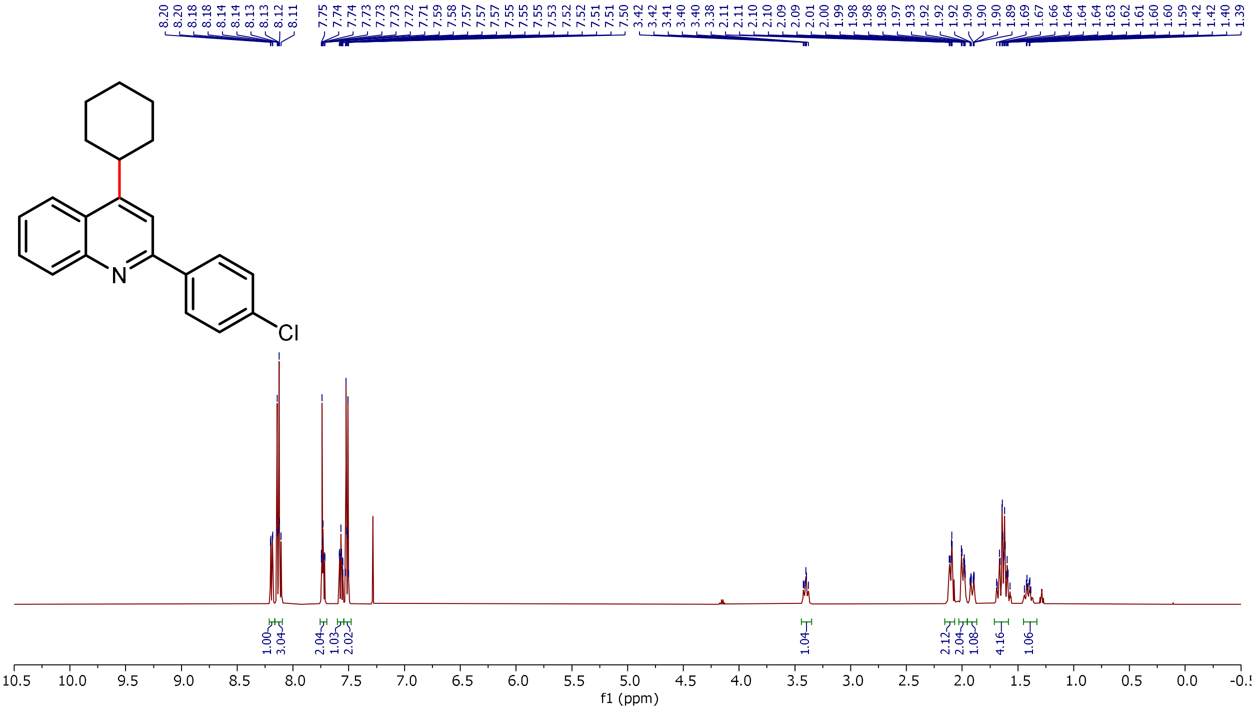

**Supplementary Figure 42b | $^{13}$C NMR (125 MHz, $CDCl_3$) of 2-(4-chlorophenyl)-4-cyclohexylquinoline (36)**

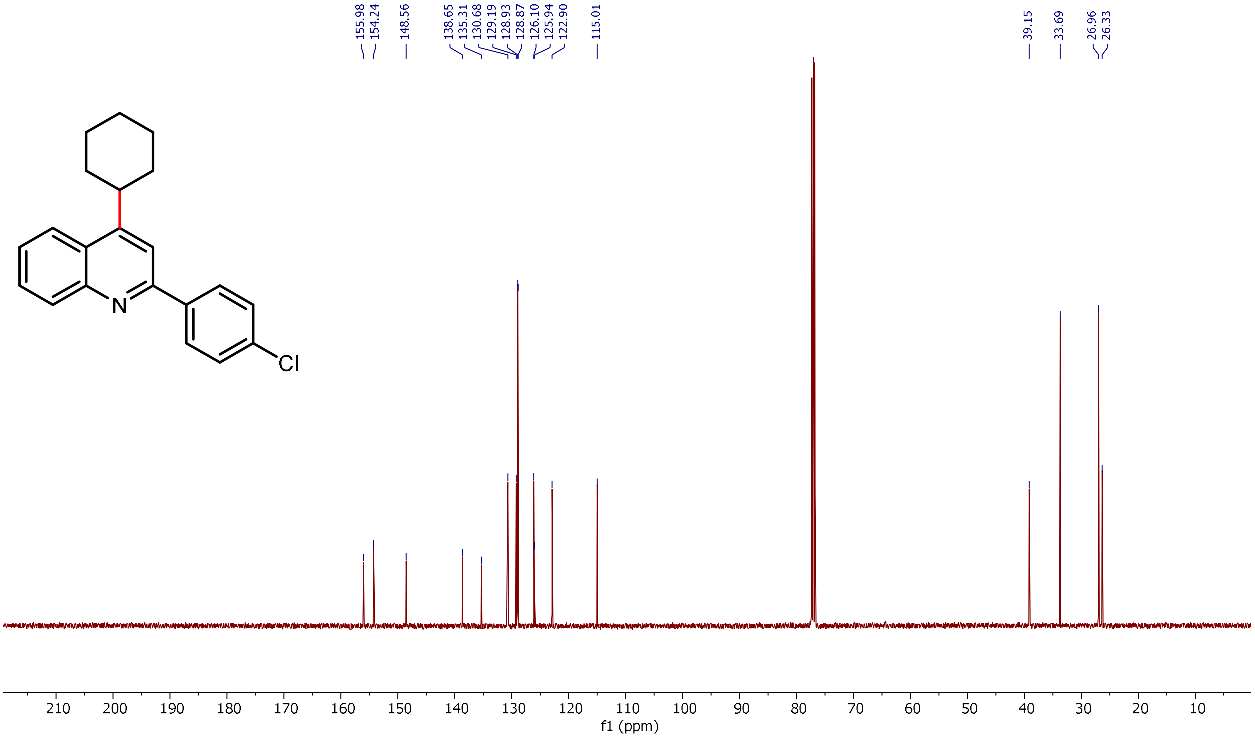

**Supplementary Figure 43a | $^{1}$H NMR (500 MHz, $CDCl_3$) of 2-(4-bromophenyl)-4-cyclohexylquinoline (37)**

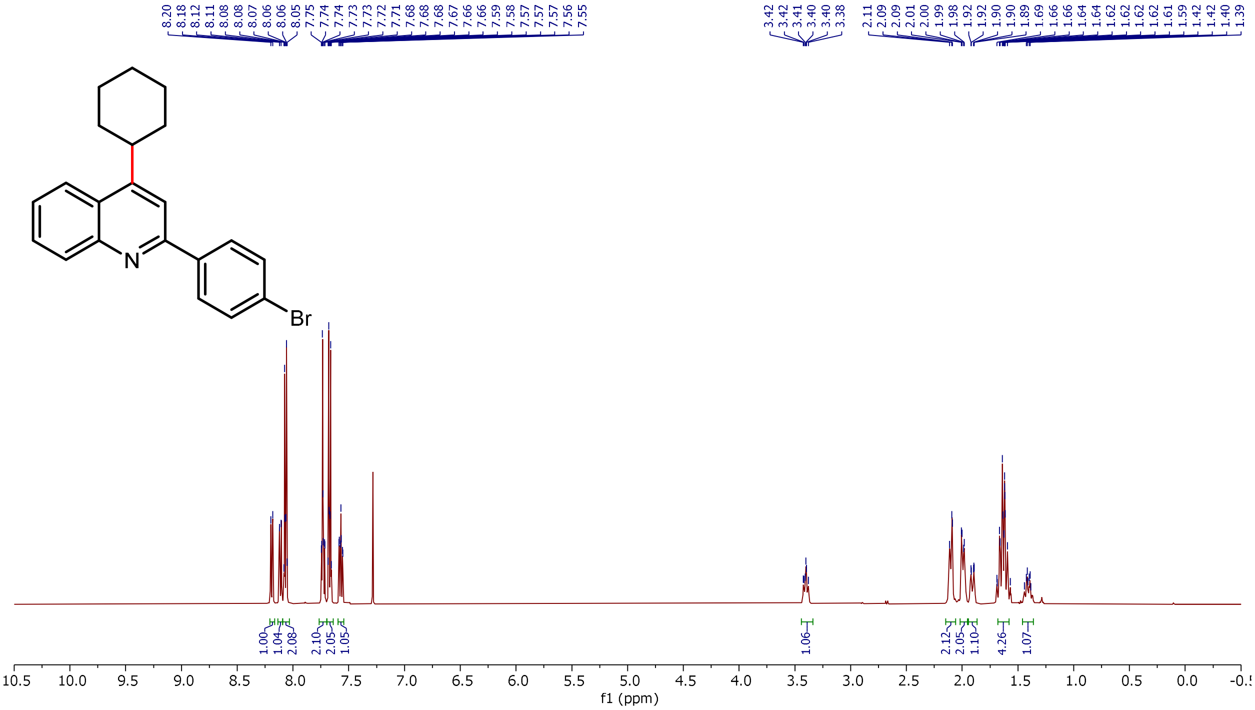


**Supplementary Figure 43b | $^{13}$C NMR (125 MHz, $CDCl_3$) of 2-(4-bromophenyl)-4-cyclohexylquinoline (37)**

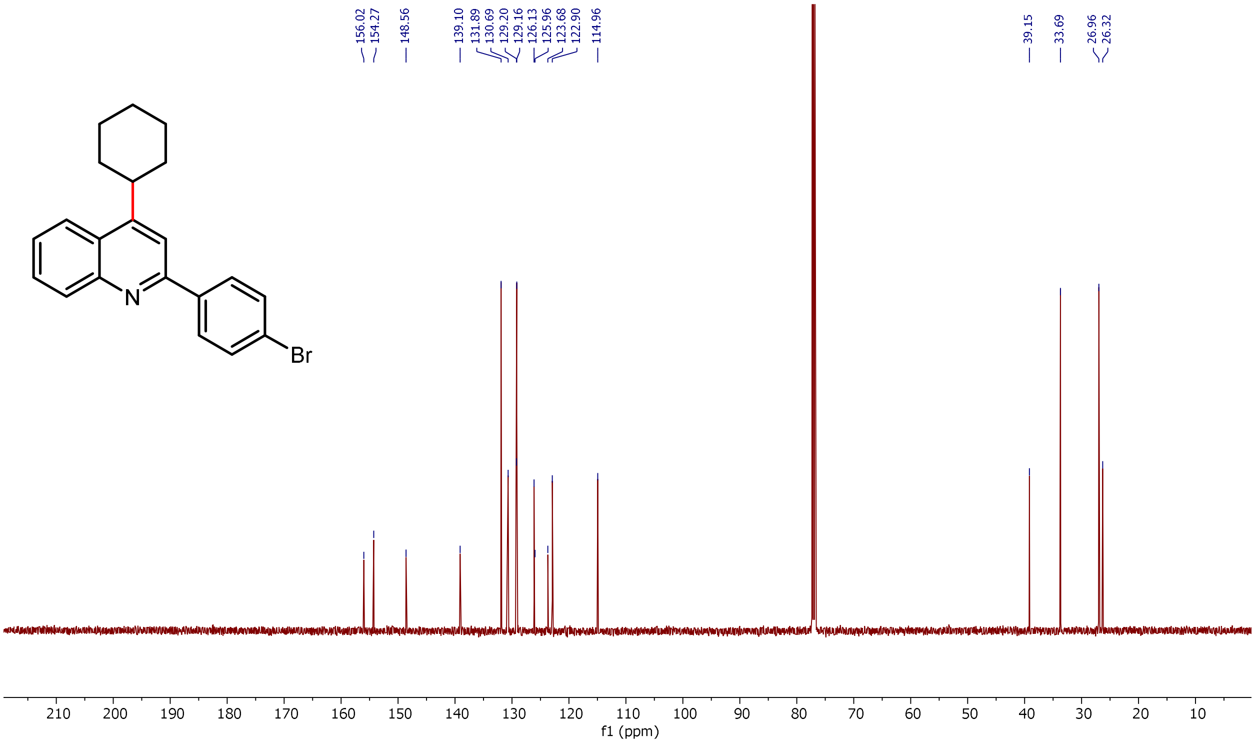

**Supplementary Figure 44a | $^{1}$H NMR (500 MHz, $CDCl_3$) of 4-(4-cyclohexylquinolin-2-yl)benzonitrile (38)**

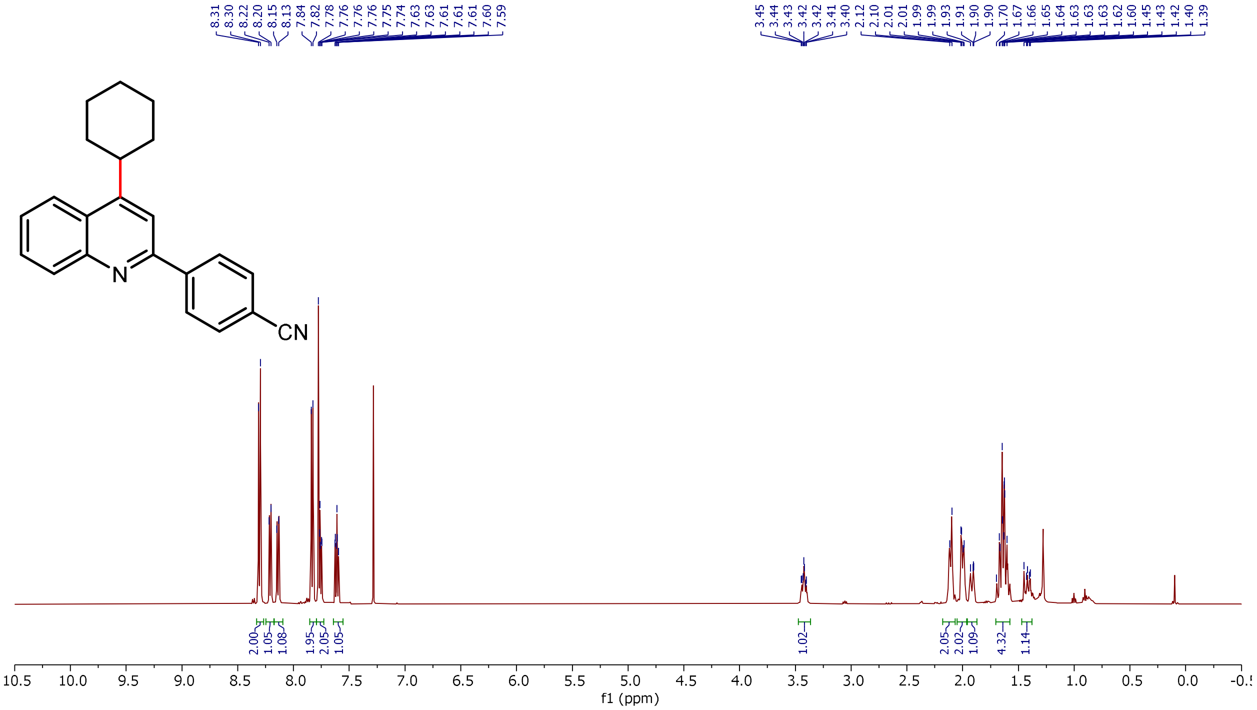


**Supplementary Figure 44b | $^{13}$C NMR (125 MHz, $CDCl_3$) of 4-(4-cyclohexylquinolin-2-yl)benzonitrile (38)**

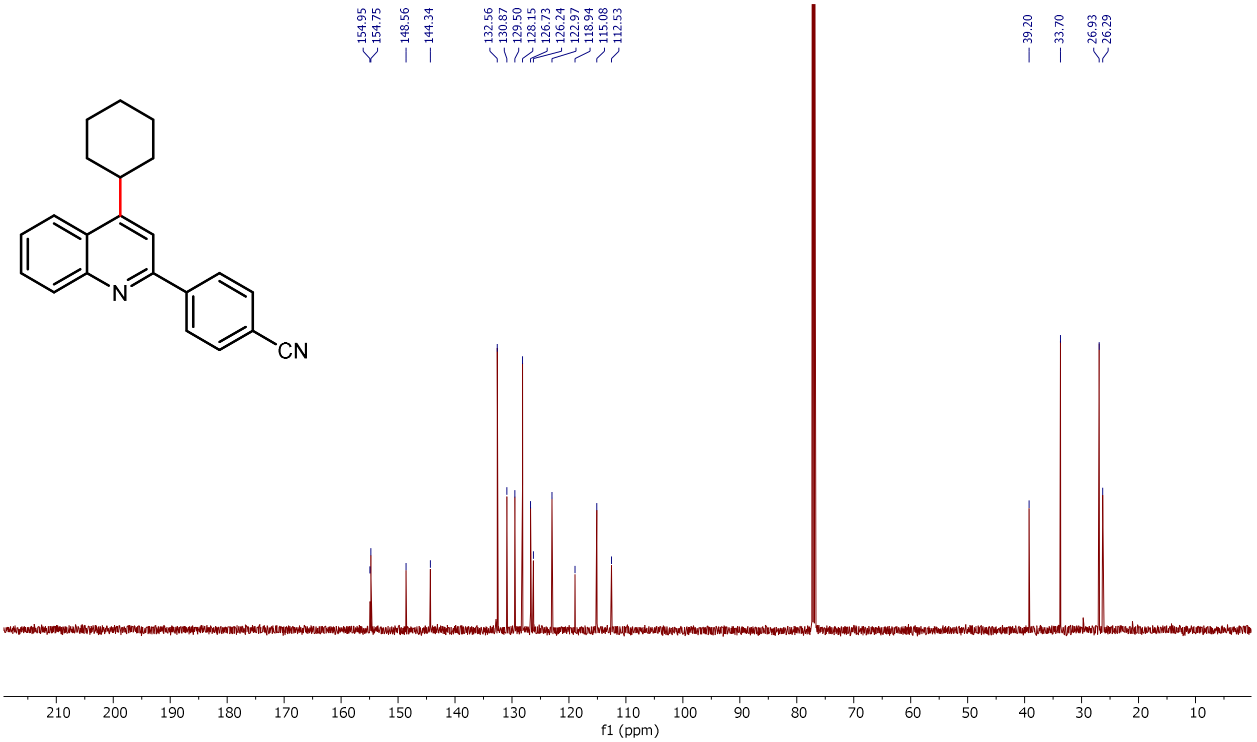

**Supplementary Figure 45a | $^{1}H$ NMR (500 MHz, $CDCl_3$) of 1-(4-(4-cyclohexylquinolin-2-yl)phenyl)ethan-1-one (39)**

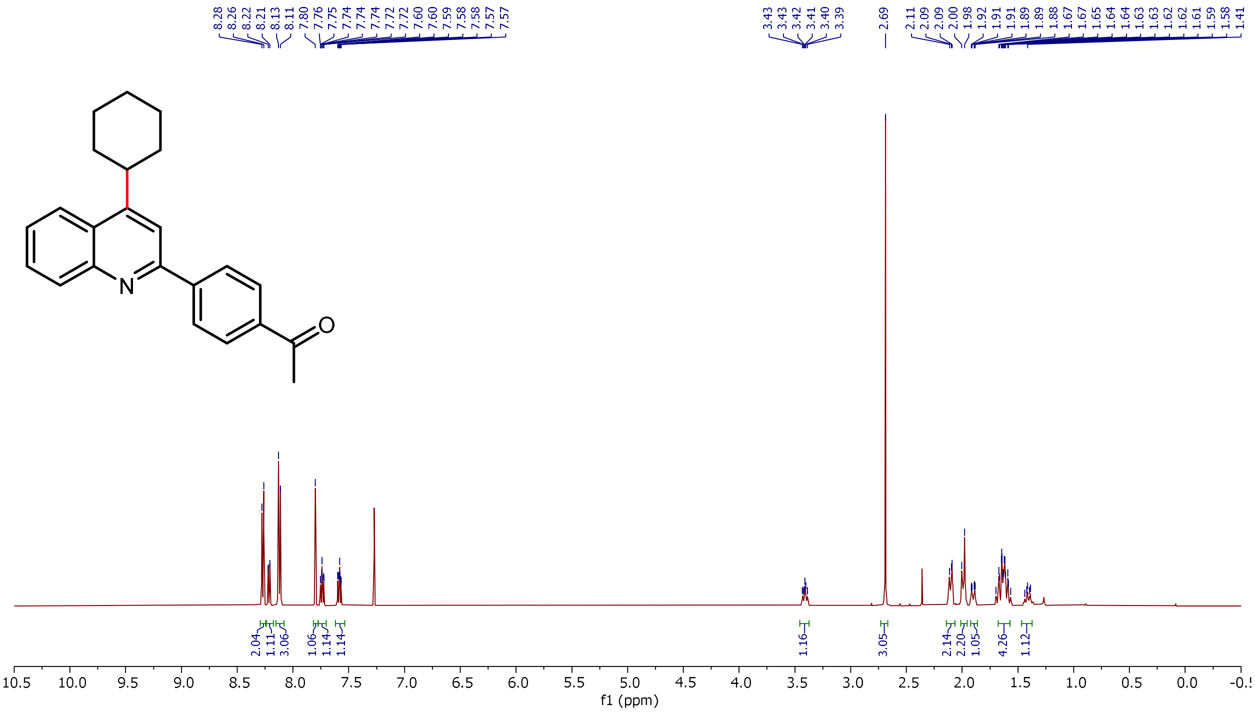


**Supplementary Figure 45b | $^{13}C$ NMR (125 MHz, $CDCl_3$) of 1-(4-(4-cyclohexylquinolin-2-yl)phenyl)ethan-1-one (39)**

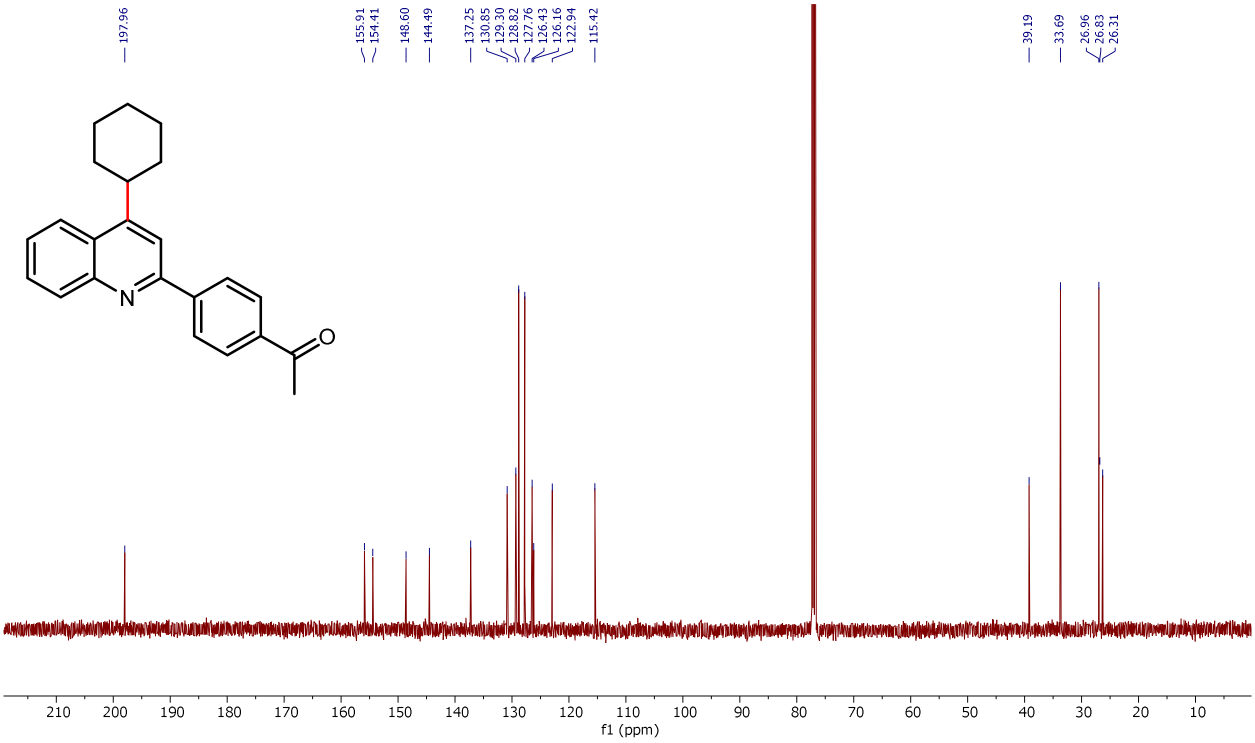

**Supplementary Figure 46a | $^{1}$H NMR (500 MHz, $CDCl_3$) of 4-cyclohexyl-2-(*p*-tolyl)quinoline (40)**

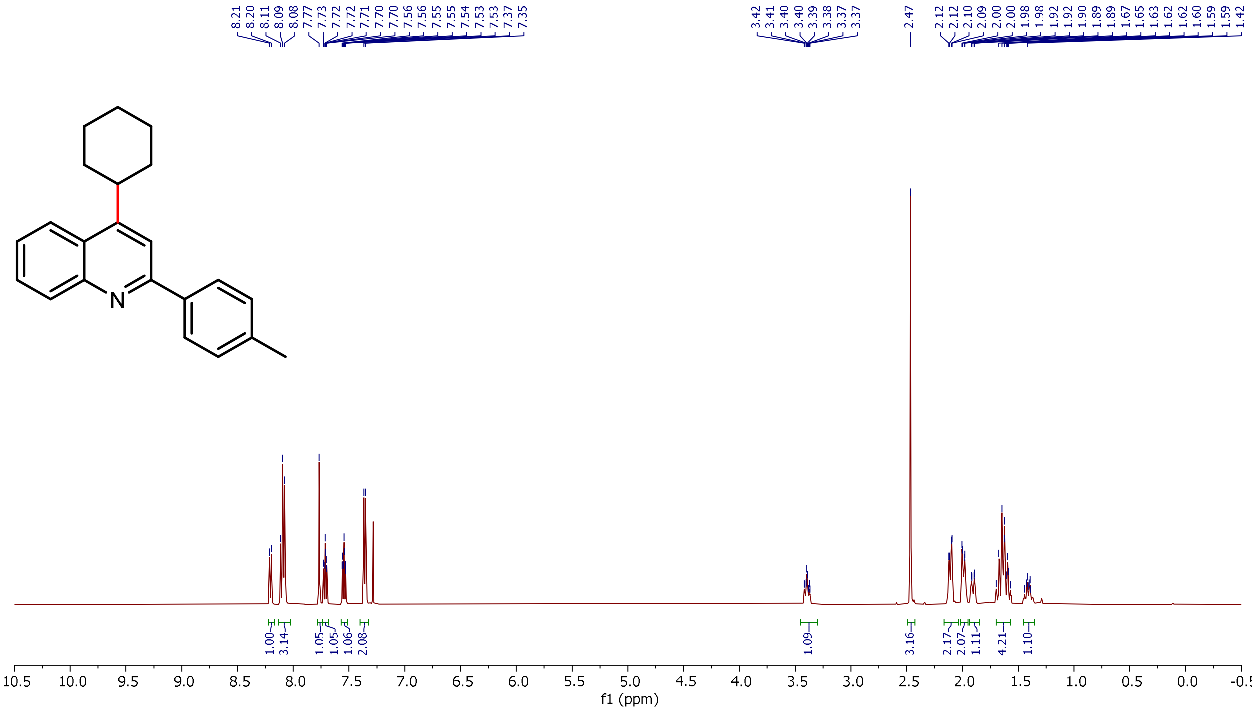


**Supplementary Figure 46b | $^{13}$C NMR (125 MHz, $CDCl_3$) of 4-cyclohexyl-2-(*p*-tolyl)quinoline (40)**

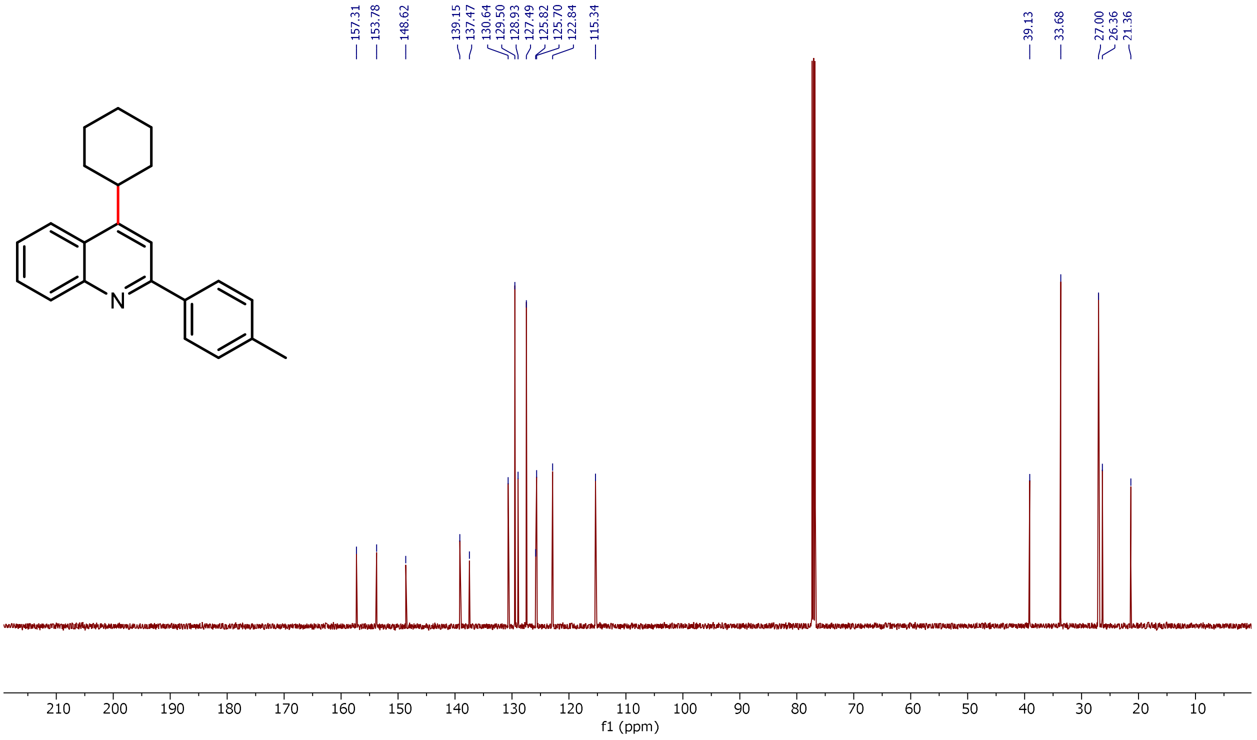

**Supplementary Figure 47a | $^{1}$H NMR (500 MHz, $CDCl_3$) of 2-([1,1'-biphenyl]-4-yl)-4-cyclohexylquinoline (41)**

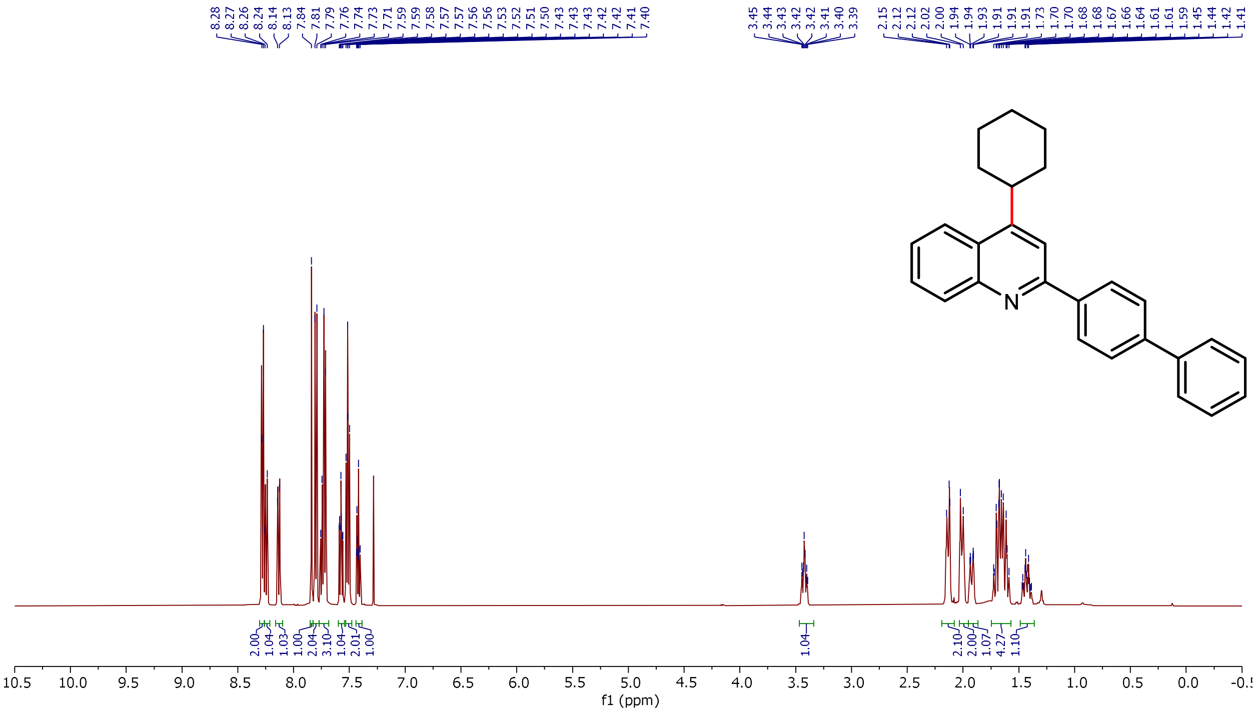


**Supplementary Figure 47b | $^{13}$C NMR (125 MHz, $CDCl_3$) of 2-([1,1'-biphenyl]-4-yl)-4-cyclohexylquinoline (41)**

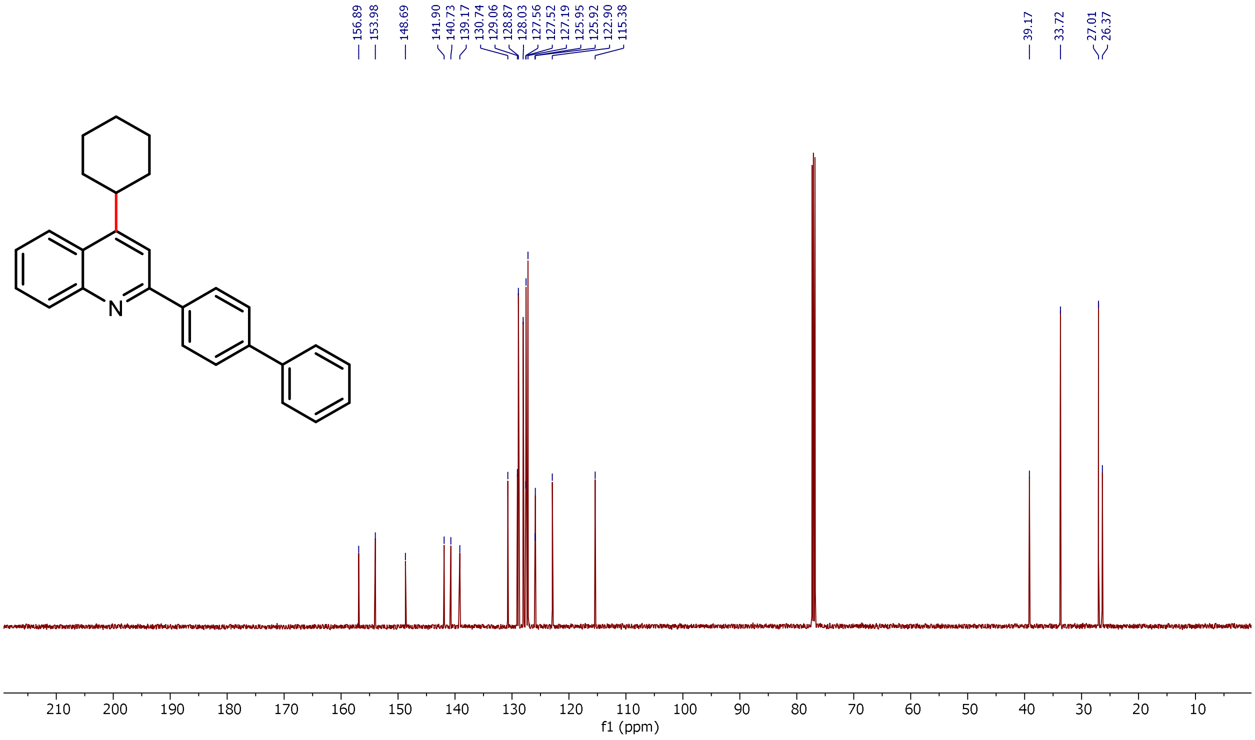

**Supplementary Figure 48a | $^{1}H$ NMR (500 MHz, $CDCl_3$) of 4-cyclohexyl-2-(4-methoxyphenyl)quinoline (42)**

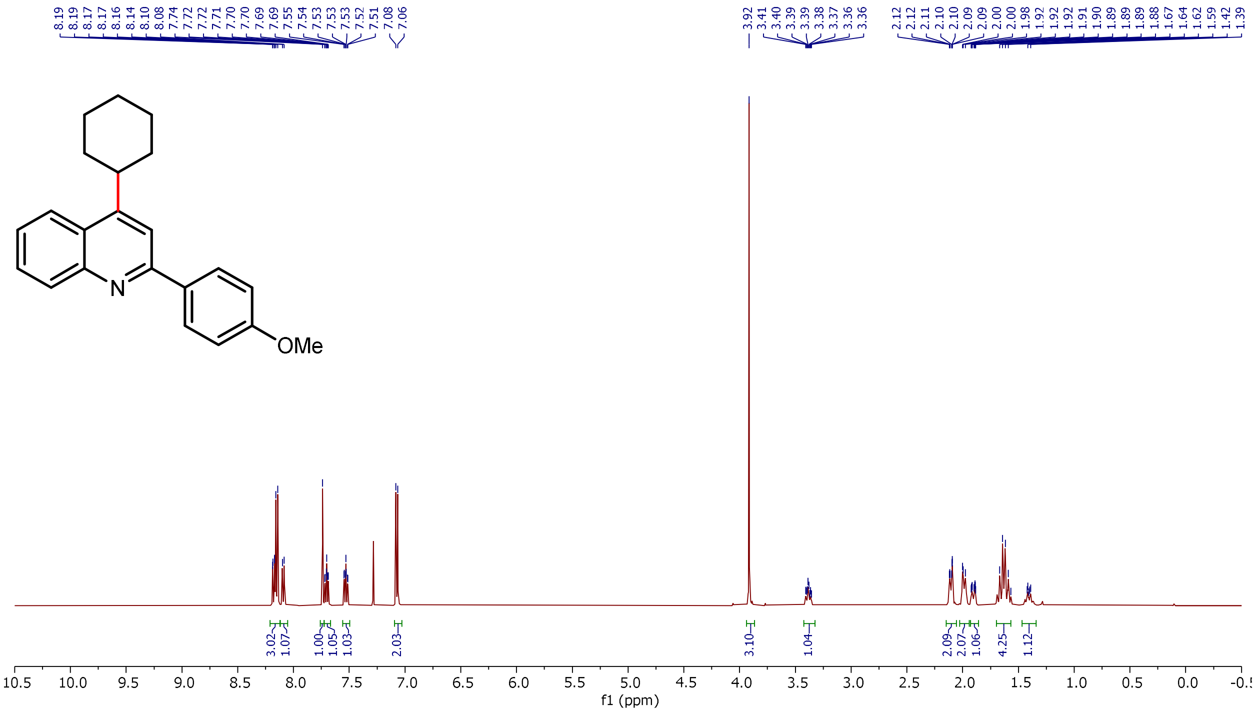


**Supplementary Figure 48b | $^{13}C$ NMR (125 MHz, $CDCl_3$) of 4-cyclohexyl-2-(4-methoxyphenyl)quinoline (42)**

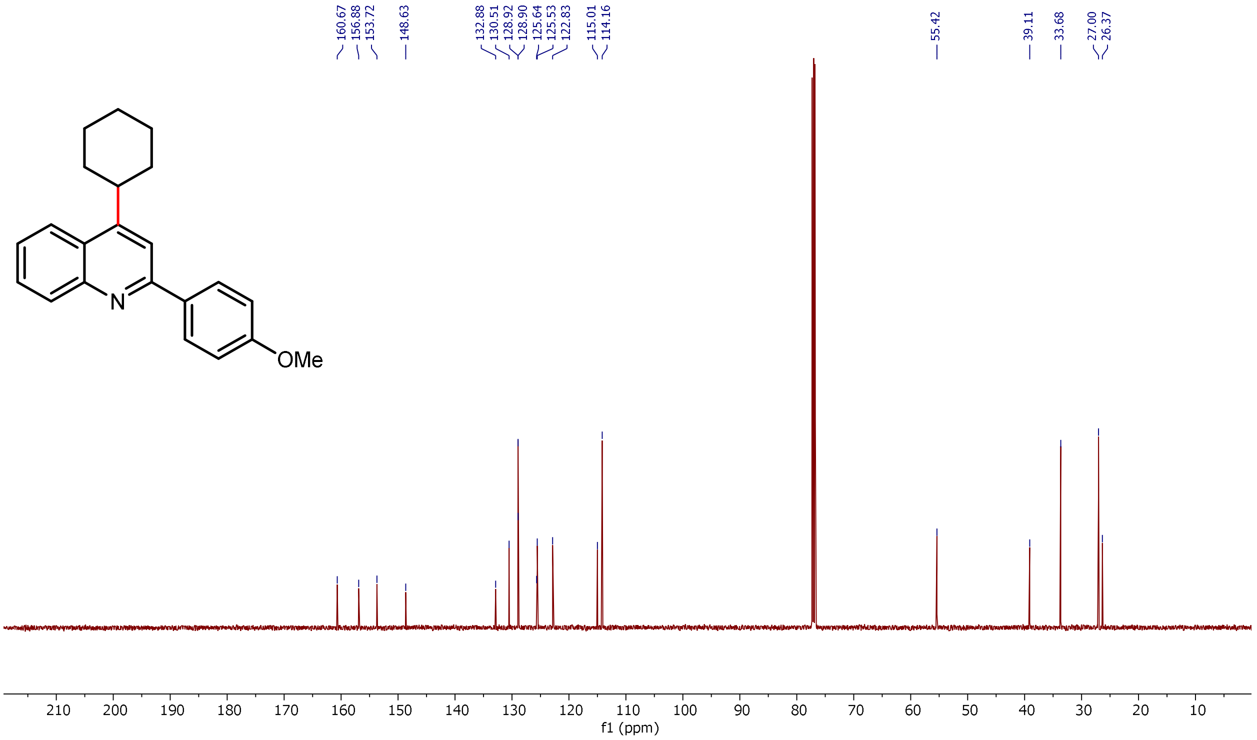

## Supplementary Figure 49a | $^{1}$H NMR (500 MHz, $CDCl_3$) of 4-cyclohexyl-2-methylquinoline (43)

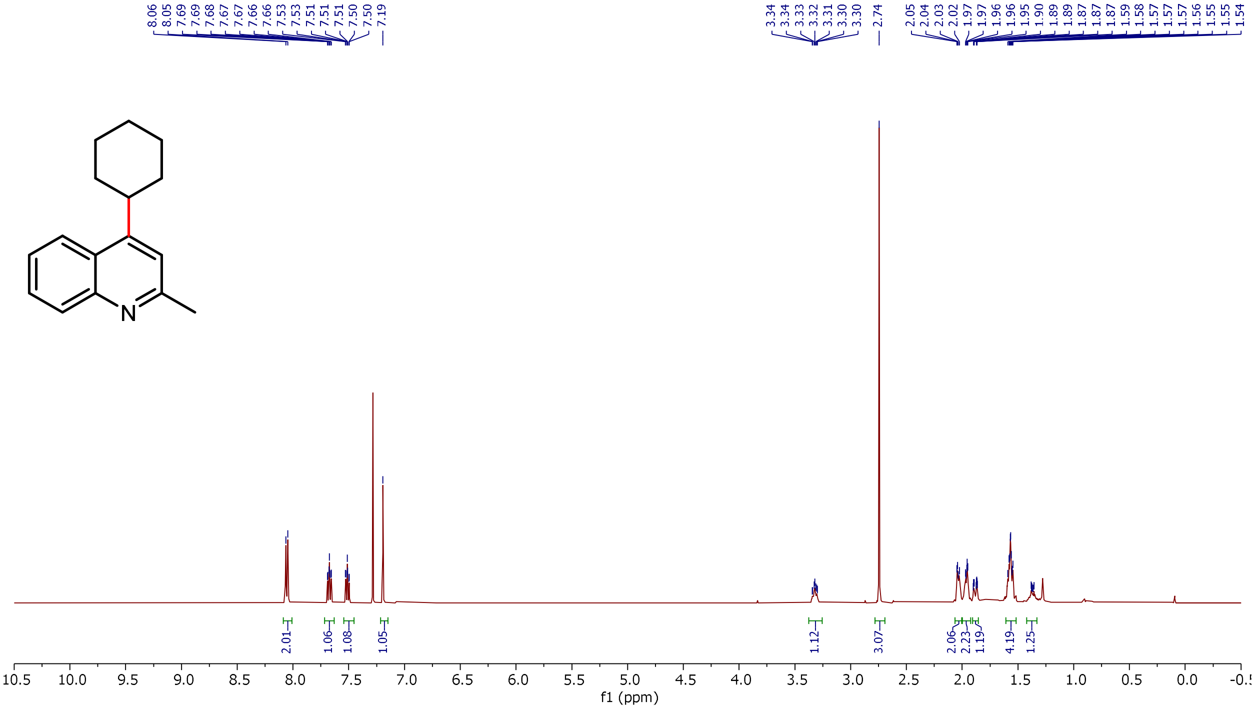


## Supplementary Figure 49b | $^{13}$C NMR (125 MHz, $CDCl_3$) of 4-cyclohexyl-2-methylquinoline (43)

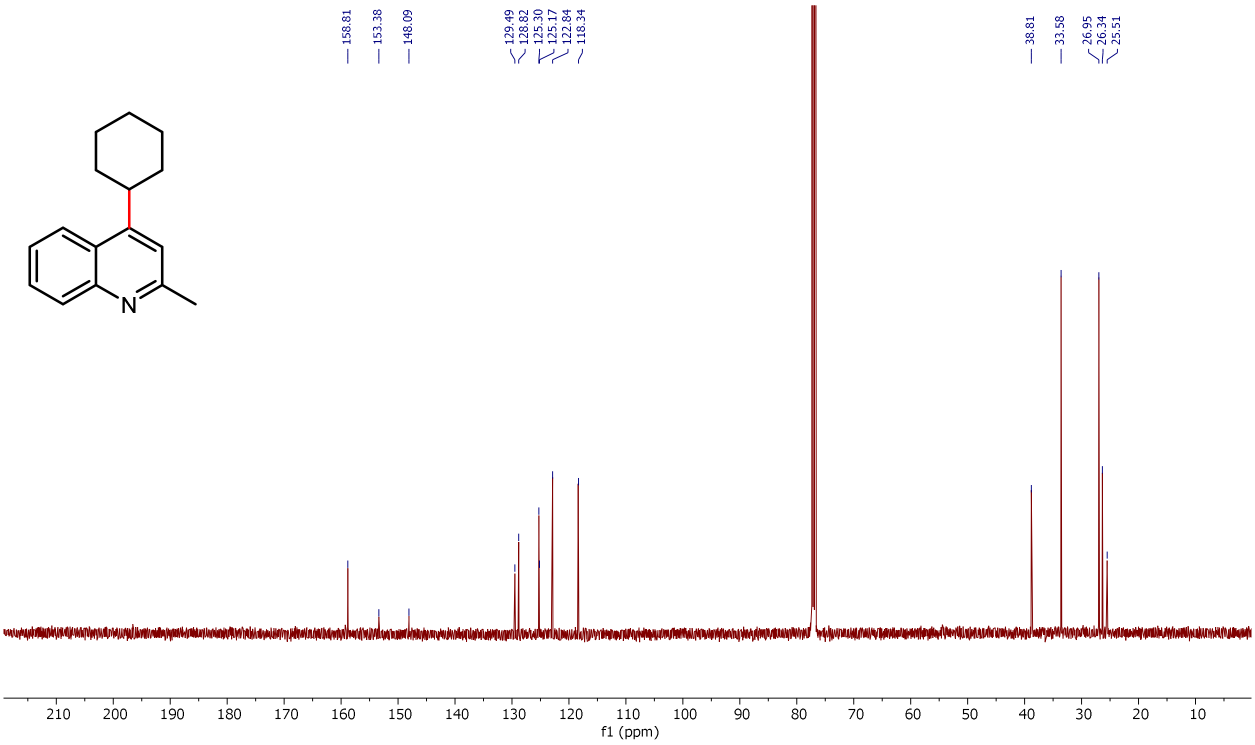

## Supplementary Figure 50a | $^{1}$H NMR (500 MHz, $CDCl_3$) of 2-chloro-4-cyclohexylquinoline (44)

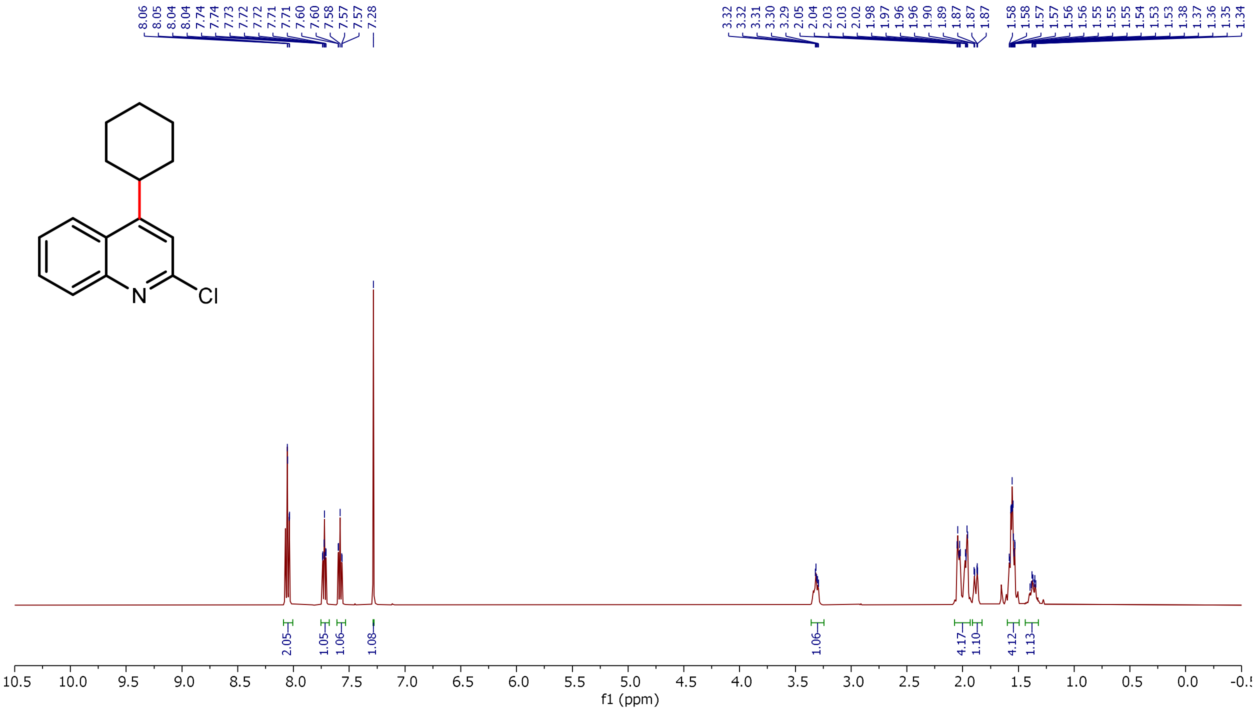


## Supplementary Figure 50b | $^{13}$C NMR (125 MHz, $CDCl_3$) of 2-chloro-4-cyclohexylquinoline (44)

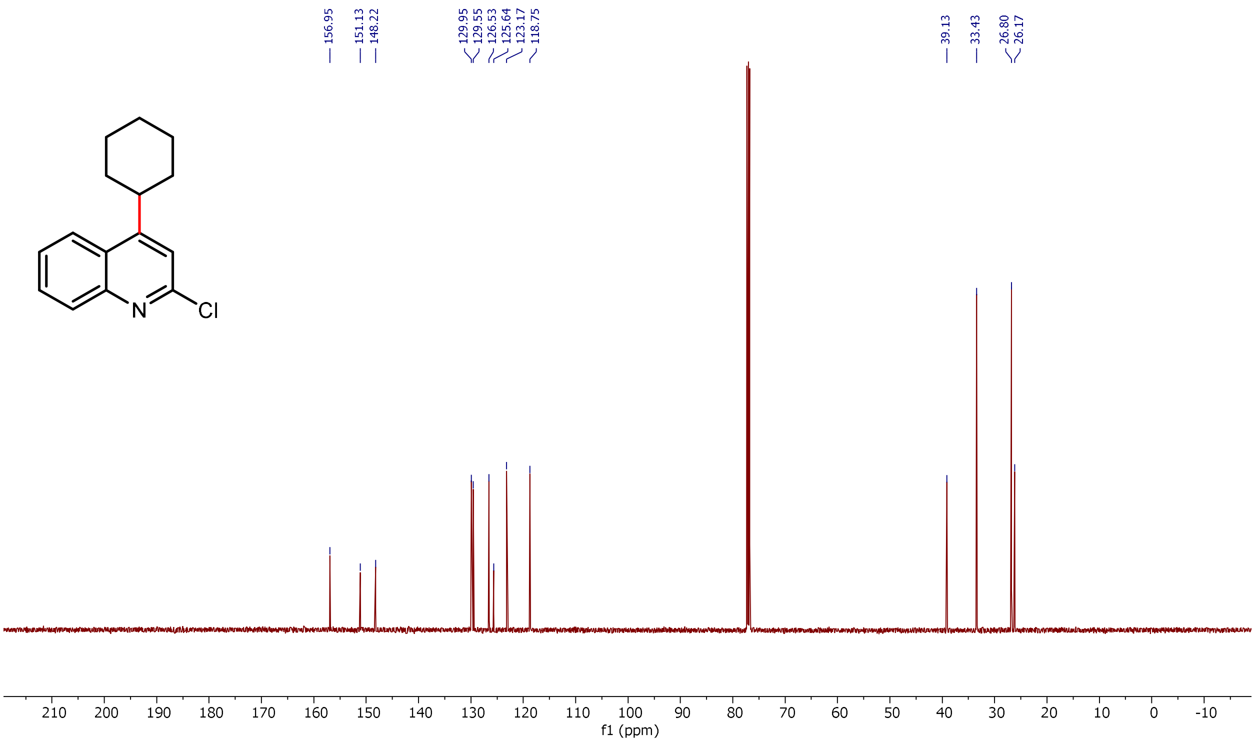

## Supplementary Figure 51a | $^{1}$H NMR (500 MHz, $CDCl_3$) of 4-cyclohexylquinoline-2-carbonitrile (45)

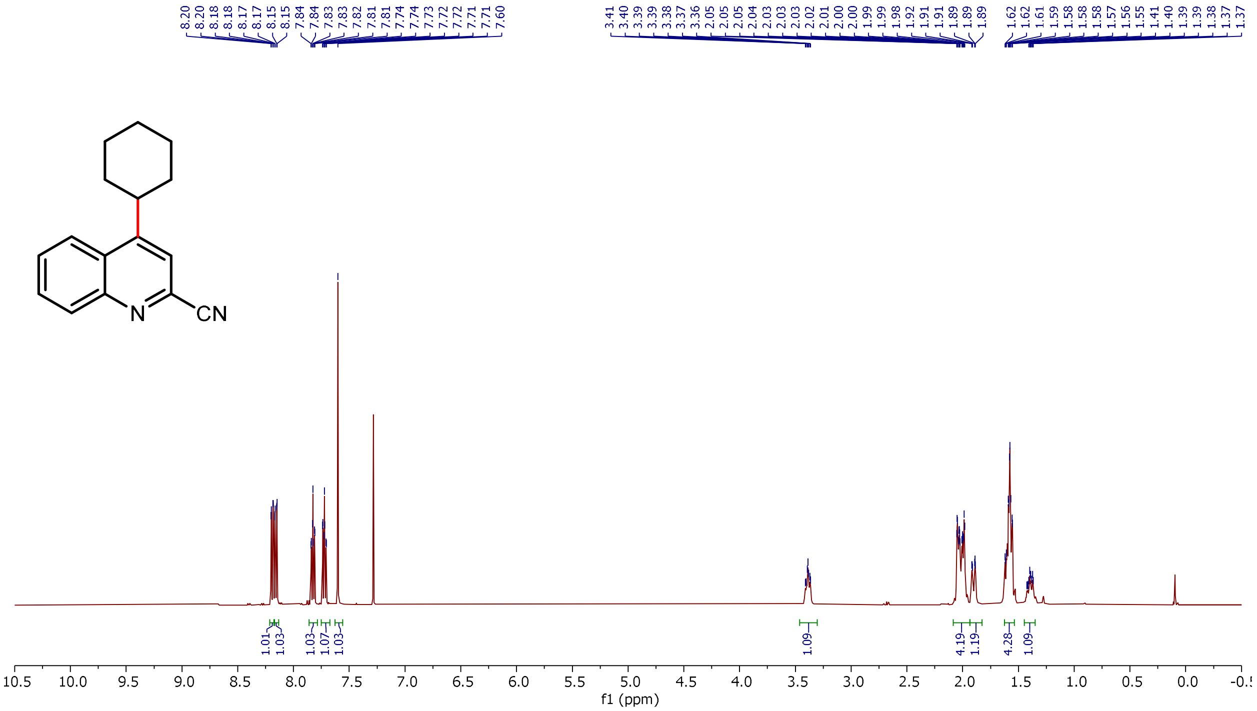


## Supplementary Figure 51b | $^{13}$C NMR (125 MHz, $CDCl_3$) of 4-cyclohexylquinoline-2-carbonitrile (45)

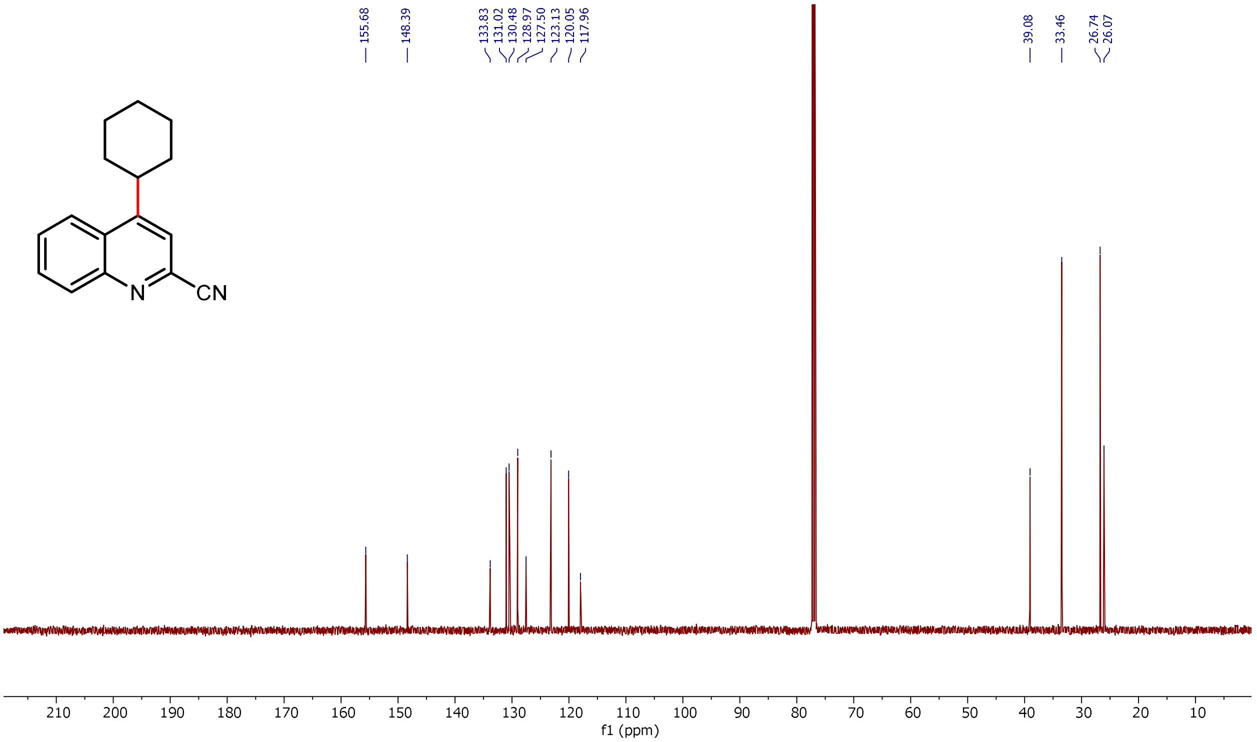

**Supplementary Figure 52a | $^1$H NMR (500 MHz, $CDCl_3$) of 4-cyclohexyl-2-phenethylquinoline (46)**

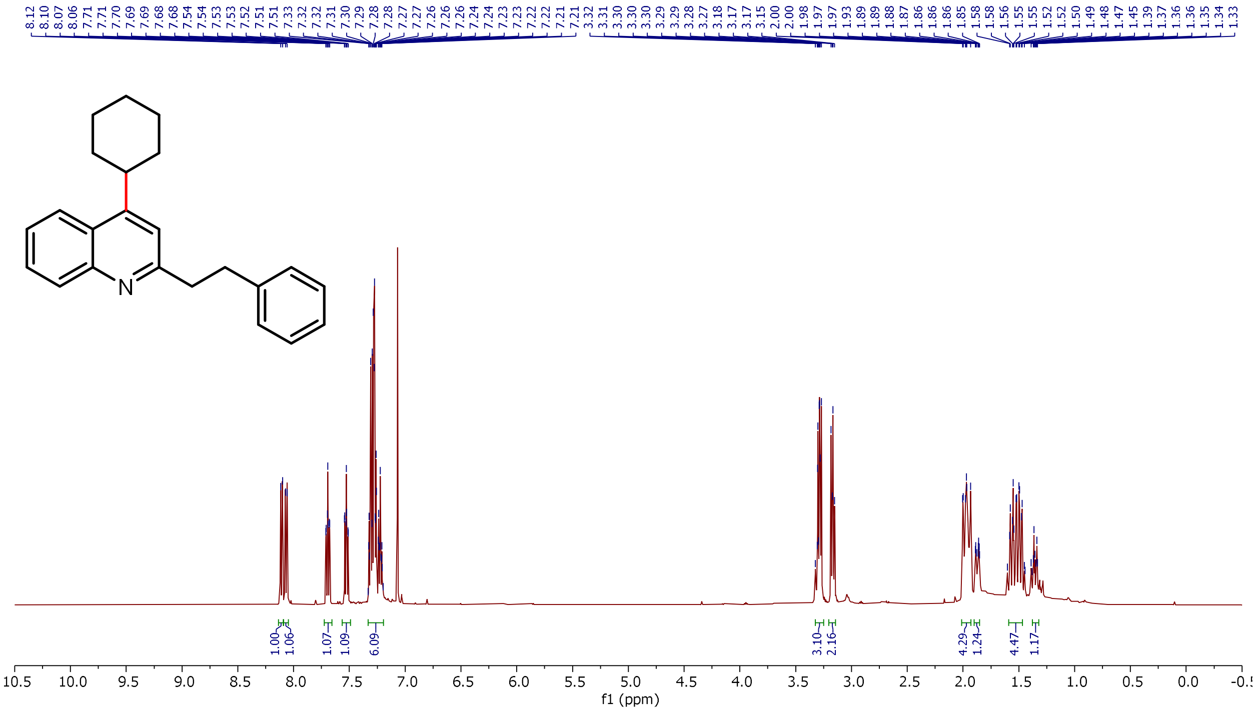


**Supplementary Figure 52b | $^{13}$C NMR (125 MHz, $CDCl_3$) of 4-cyclohexyl-2-phenethylquinoline (46)**

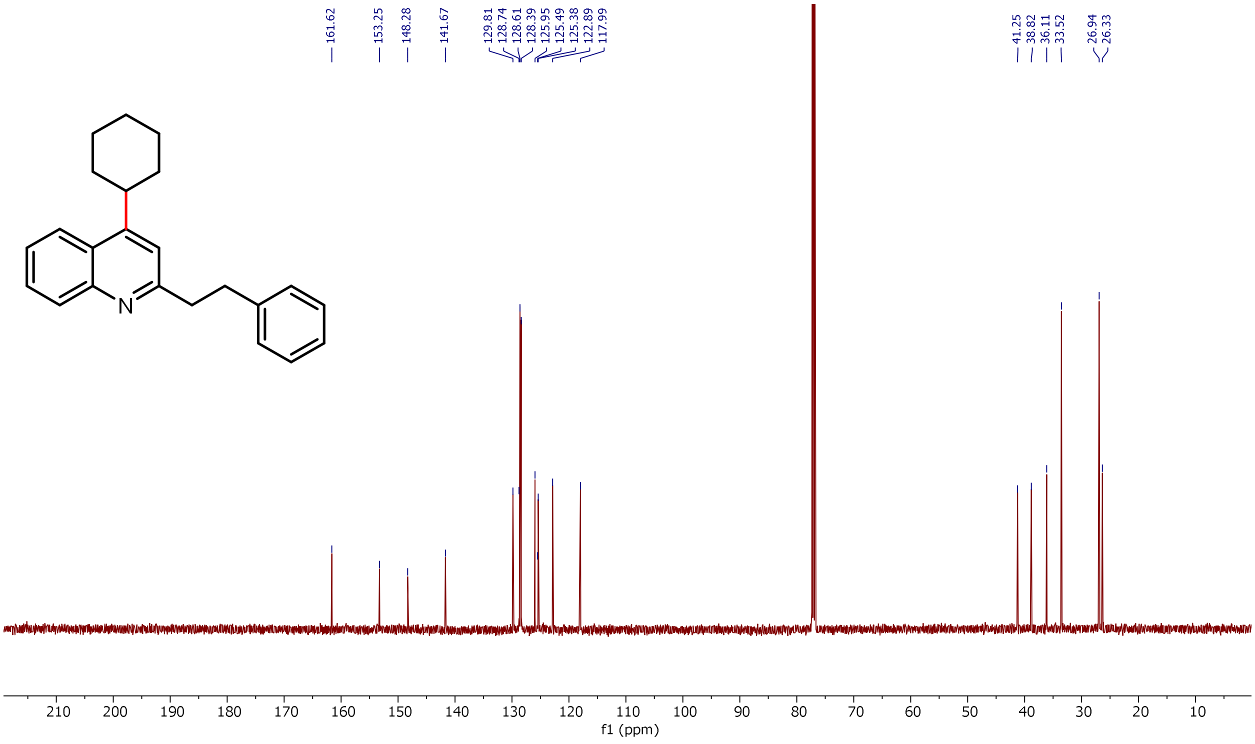

## Supplementary Figure 53a | $^{1}$H NMR (500 MHz, $CDCl_3$) of 2-cyclohexyl-4-phenylquinoline (47)

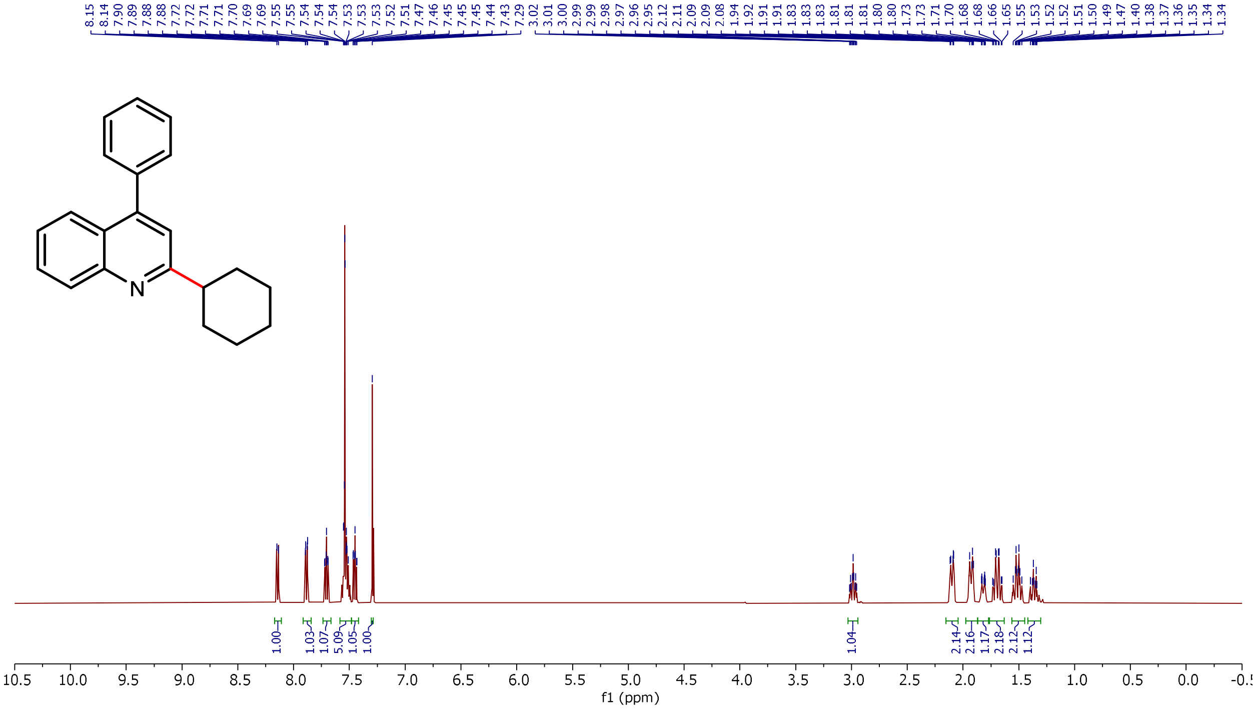


## Supplementary Figure 53b | $^{13}$C NMR (125 MHz, $CDCl_3$) of 2-cyclohexyl-4-phenylquinoline (47)

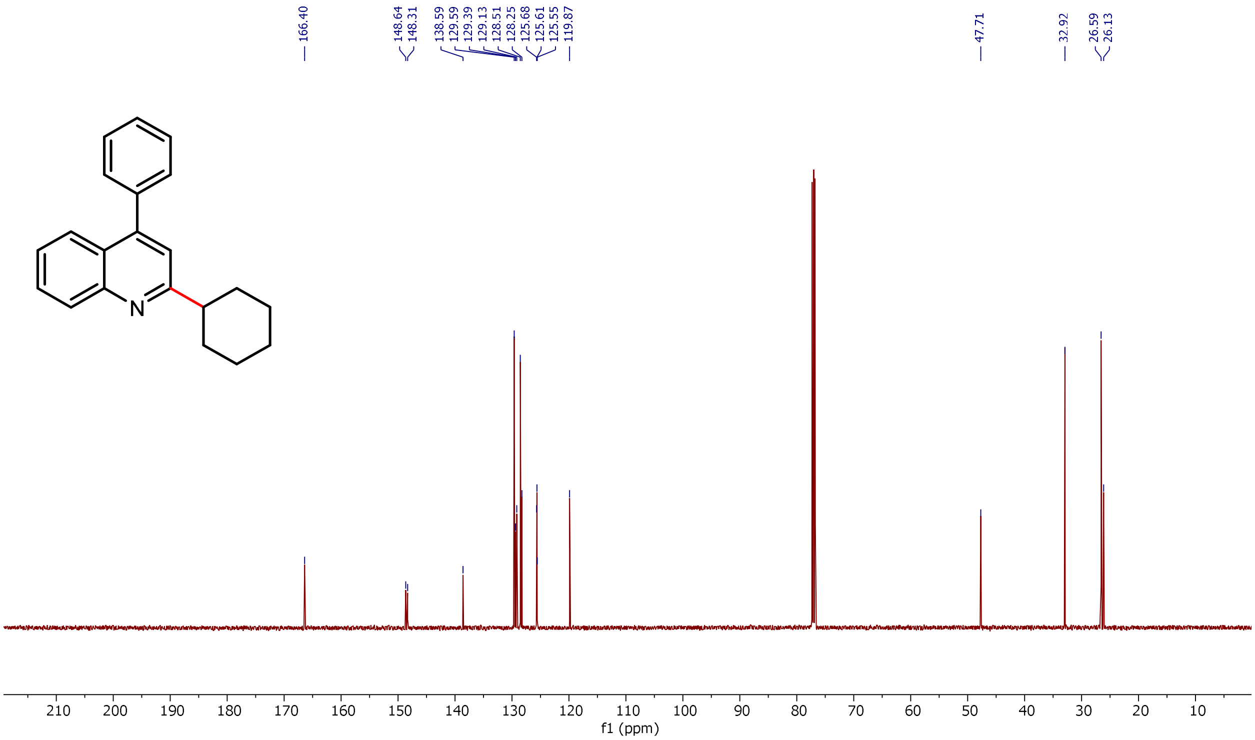

## Supplementary Figure 54a | $^1$H NMR (500 MHz, $CDCl_3$) of 2-cyclohexyl-4-methylquinoline (48)

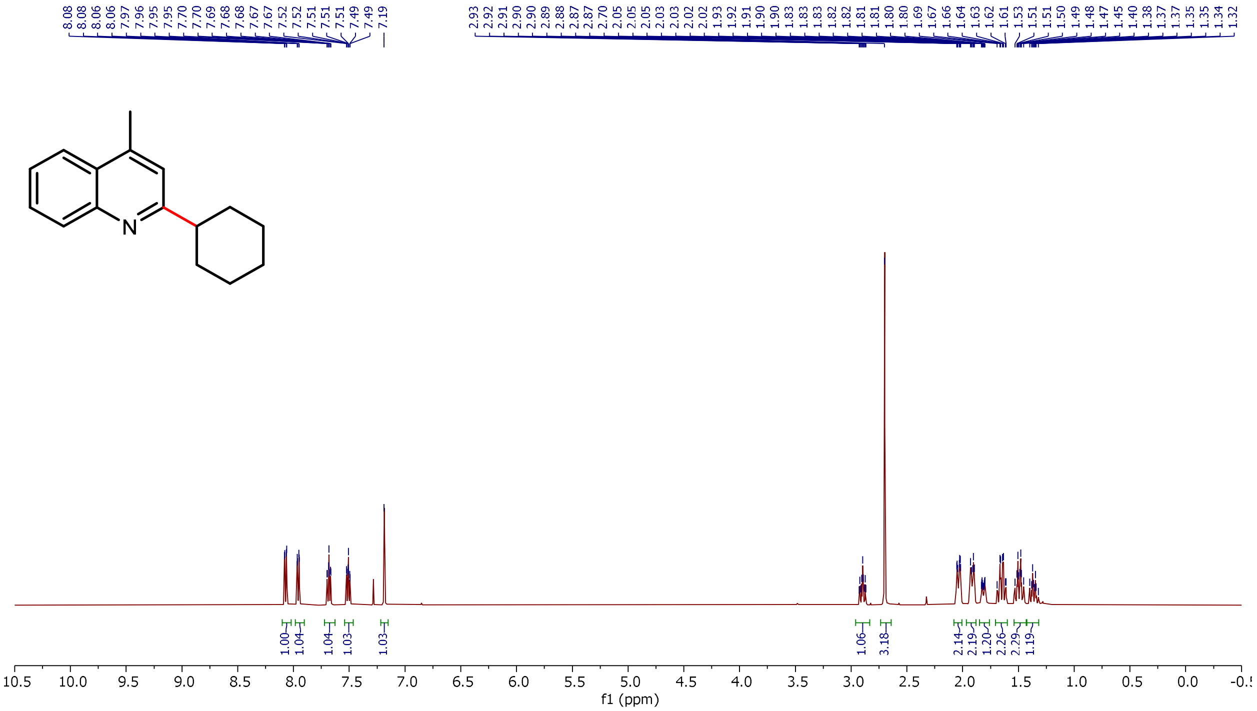


## Supplementary Figure 54b | $^{13}$C NMR (125 MHz, $CDCl_3$) of 2-cyclohexyl-4-methylquinoline (48)

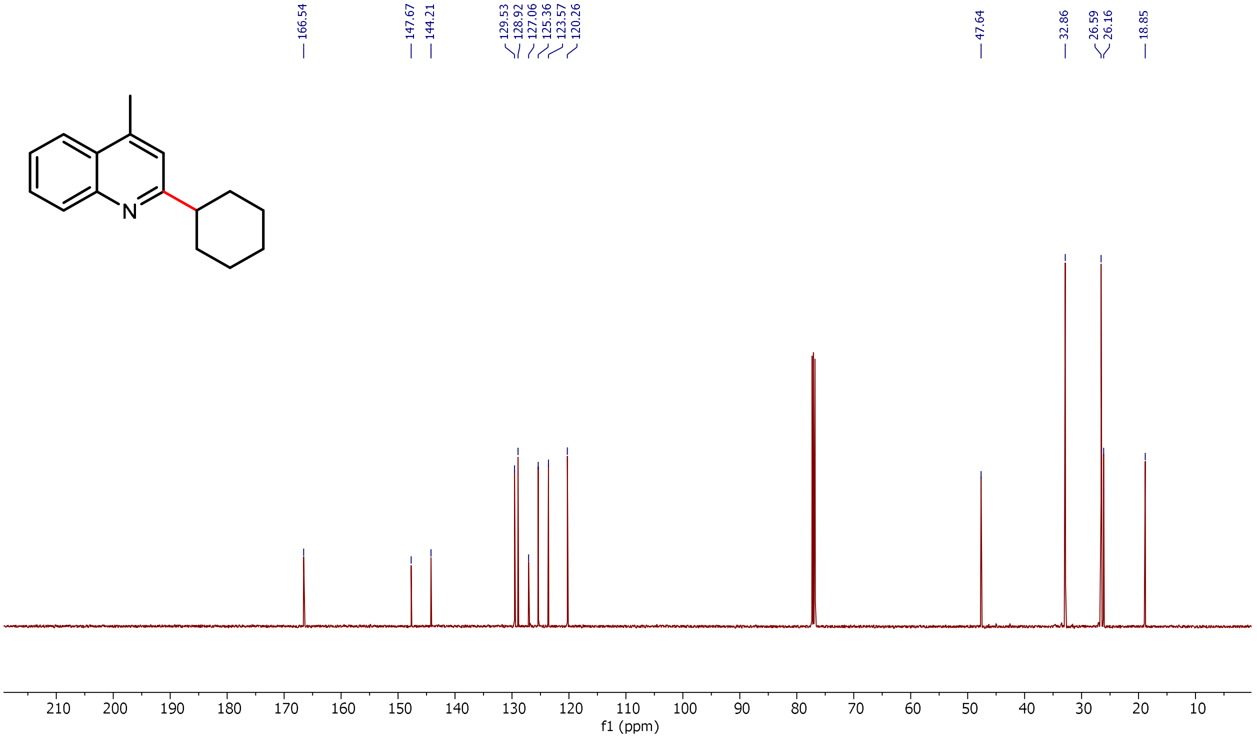

**Supplementary Figure 55a | ¹H NMR (500 MHz, $CDCl_3$) of 6-cyclohexylpyridine-2,4-dicarbonitrile (49)**

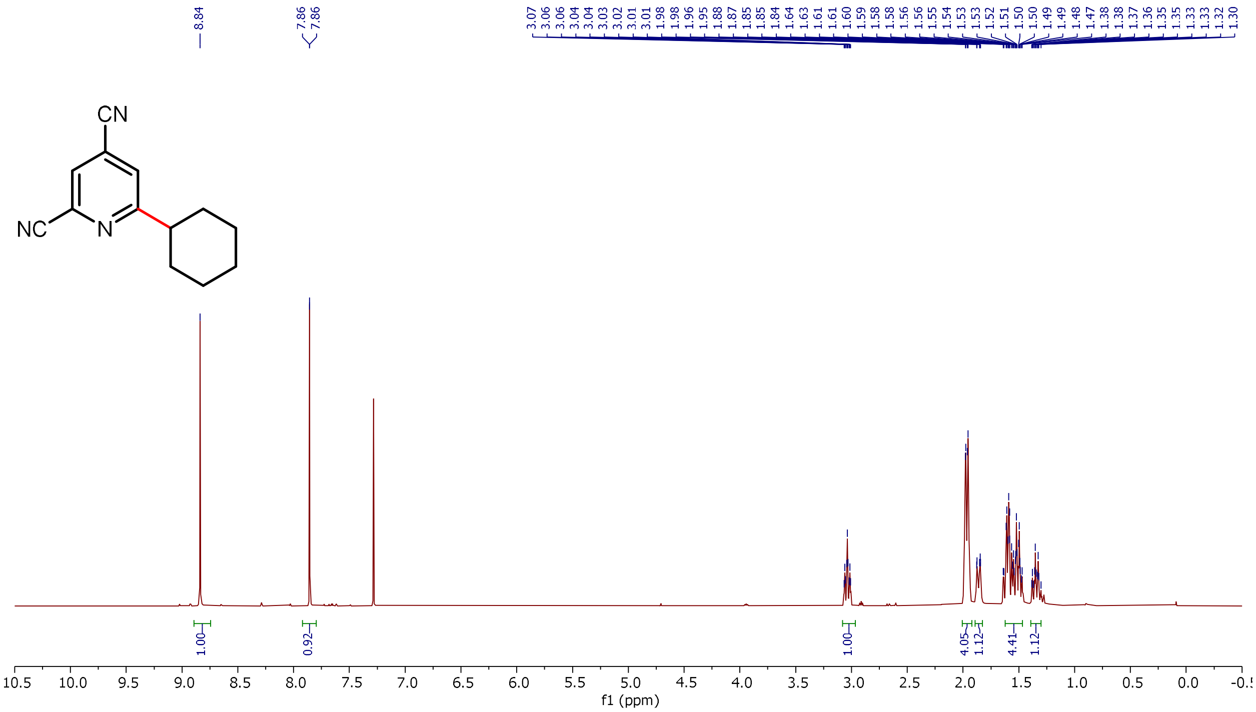


**Supplementary Figure 55b | ¹³C NMR (125 MHz, $CDCl_3$) of 6-cyclohexylpyridine-2,4-dicarbonitrile (49)**

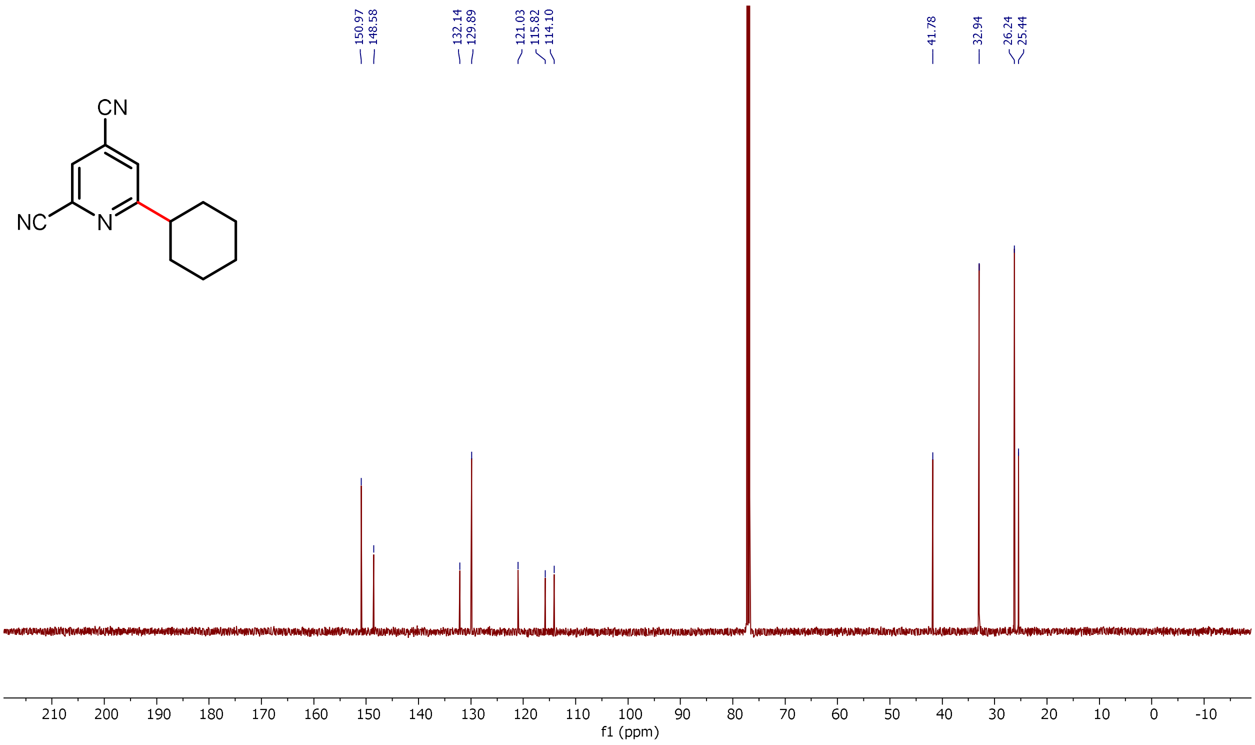

Supplementary Figure 56a | $^{1}$H NMR (500 MHz, $CDCl_3$) of 2-cyclohexylisonicotinonitrile (50a)

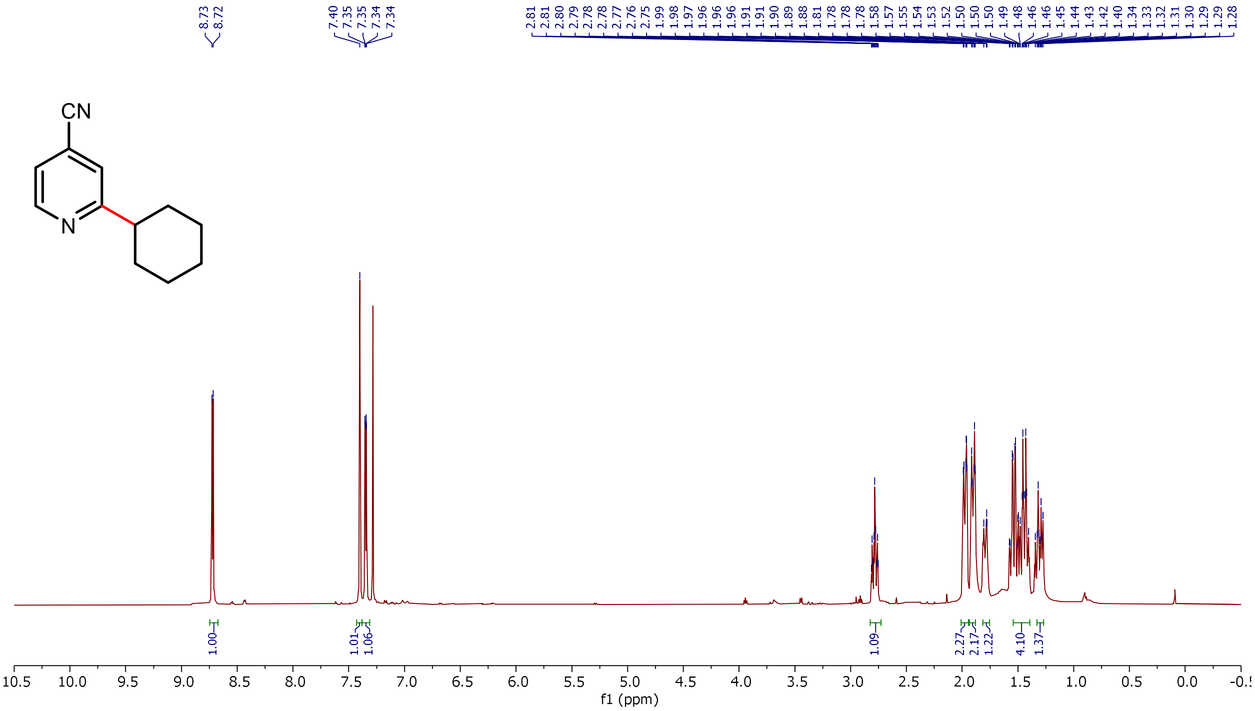


Supplementary Figure 56b | $^{13}$C NMR (125 MHz, $CDCl_3$) of 2-cyclohexylisonicotinonitrile (50a)

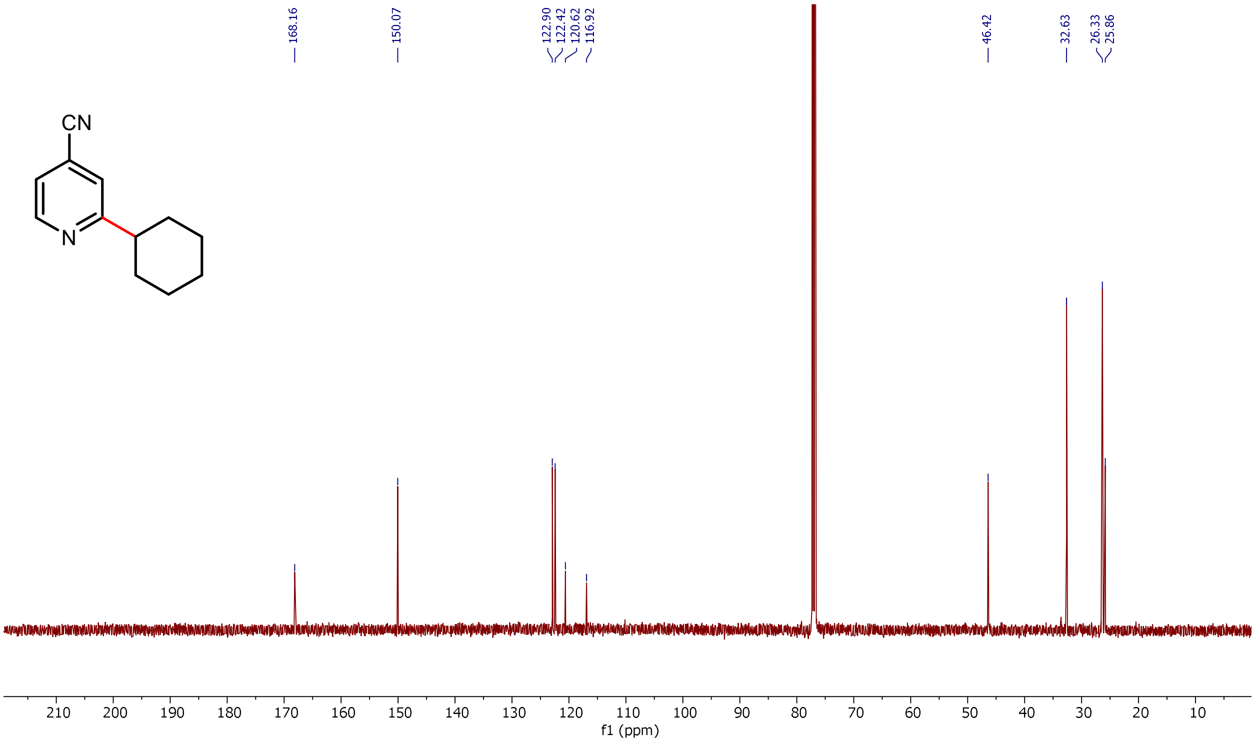

## Supplementary Figure 57a | $^{1}$H NMR (500 MHz, $CDCl_3$) of 2,6-dicyclohexylisonicotinonitrile (50b)

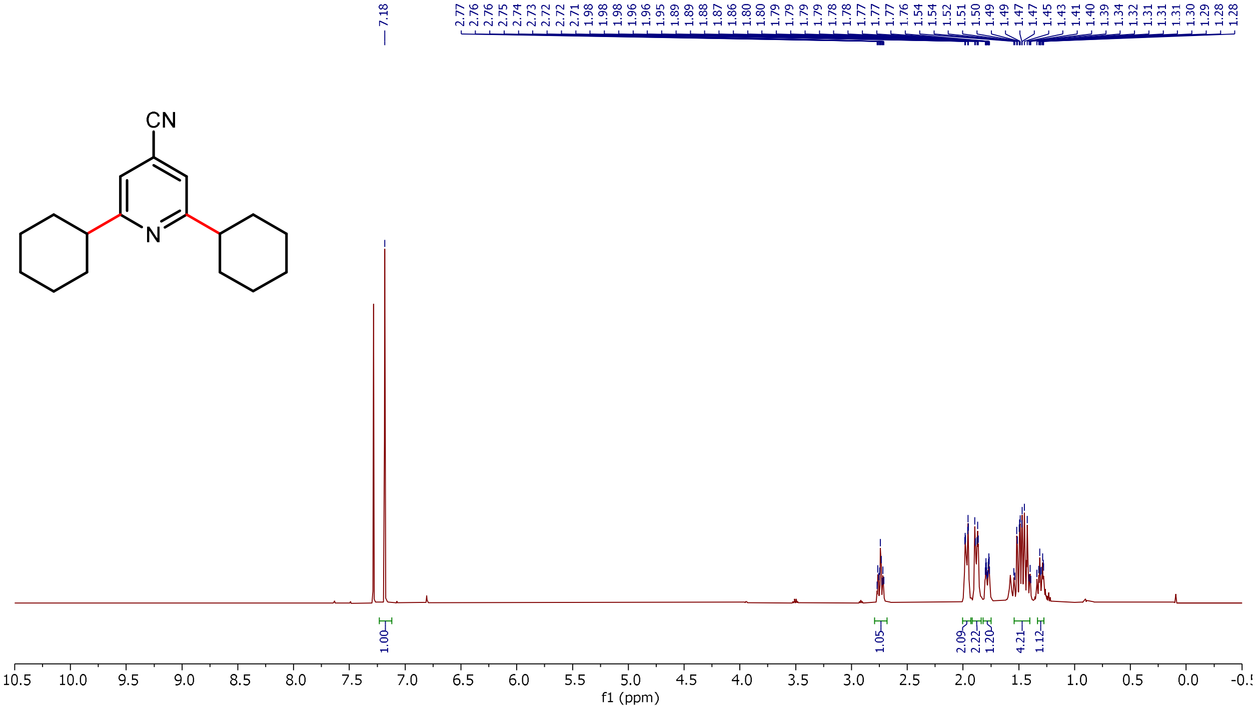


## Supplementary Figure 57b | $^{13}$C NMR (125 MHz, $CDCl_3$) of 2,6-dicyclohexylisonicotinonitrile (50b)

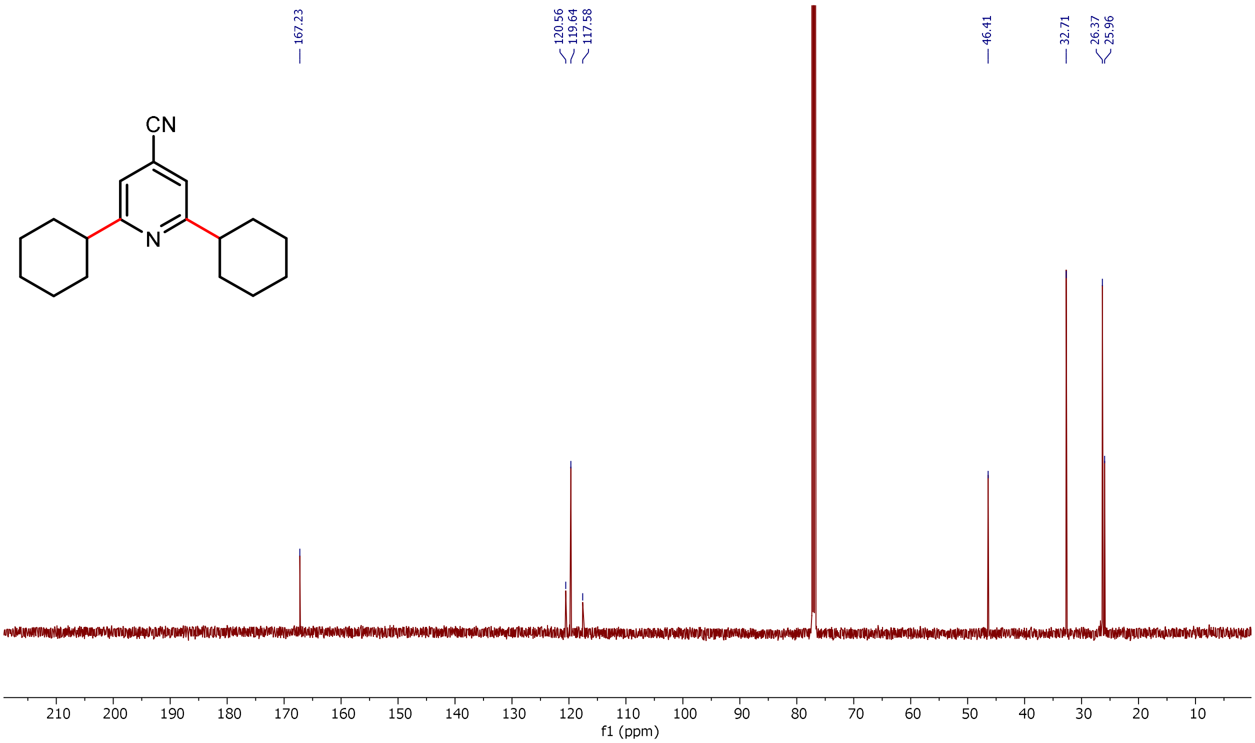

**Supplementary Figure 58a | $^{1}$H NMR (500 MHz, $CDCl_3$) of 6-cyclohexyl-*N*,*N*-diisopropylpyridine-3-sulfonamide (51)**

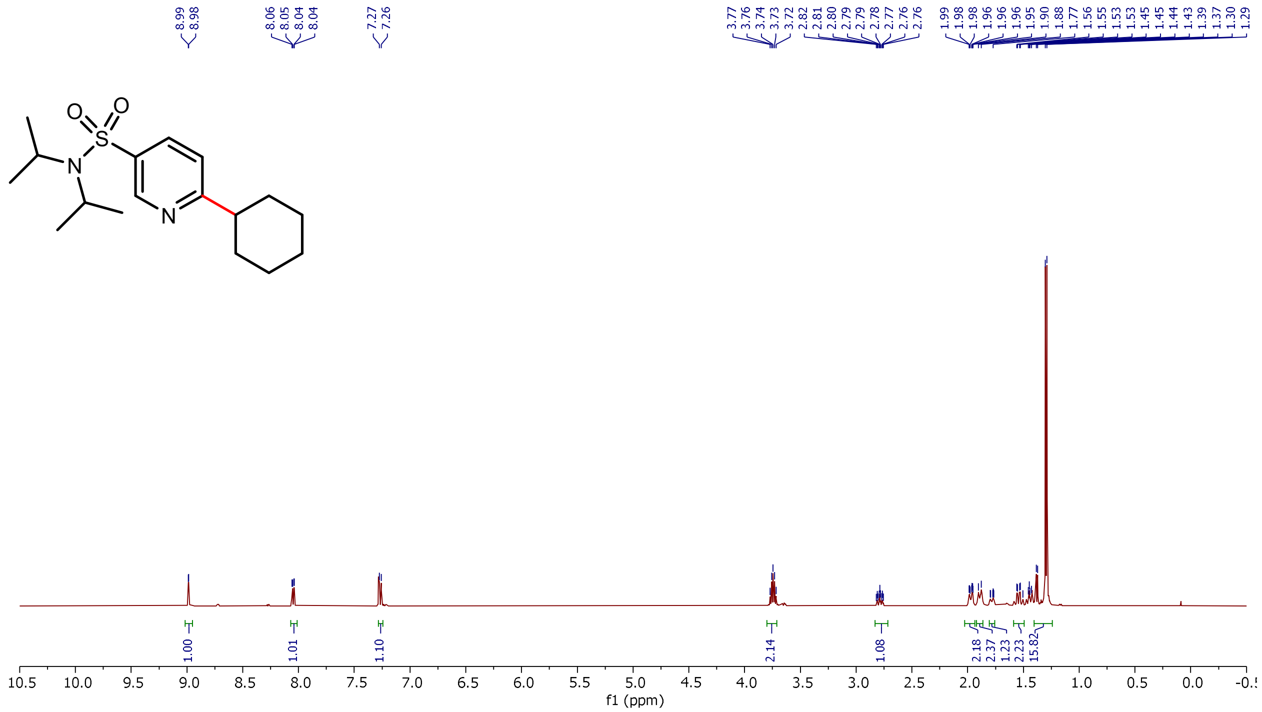


**Supplementary Figure 58b | $^{13}$C NMR (125 MHz, $CDCl_3$) of 6-cyclohexyl-*N*,*N*-diisopropylpyridine-3-sulfonamide (51)**

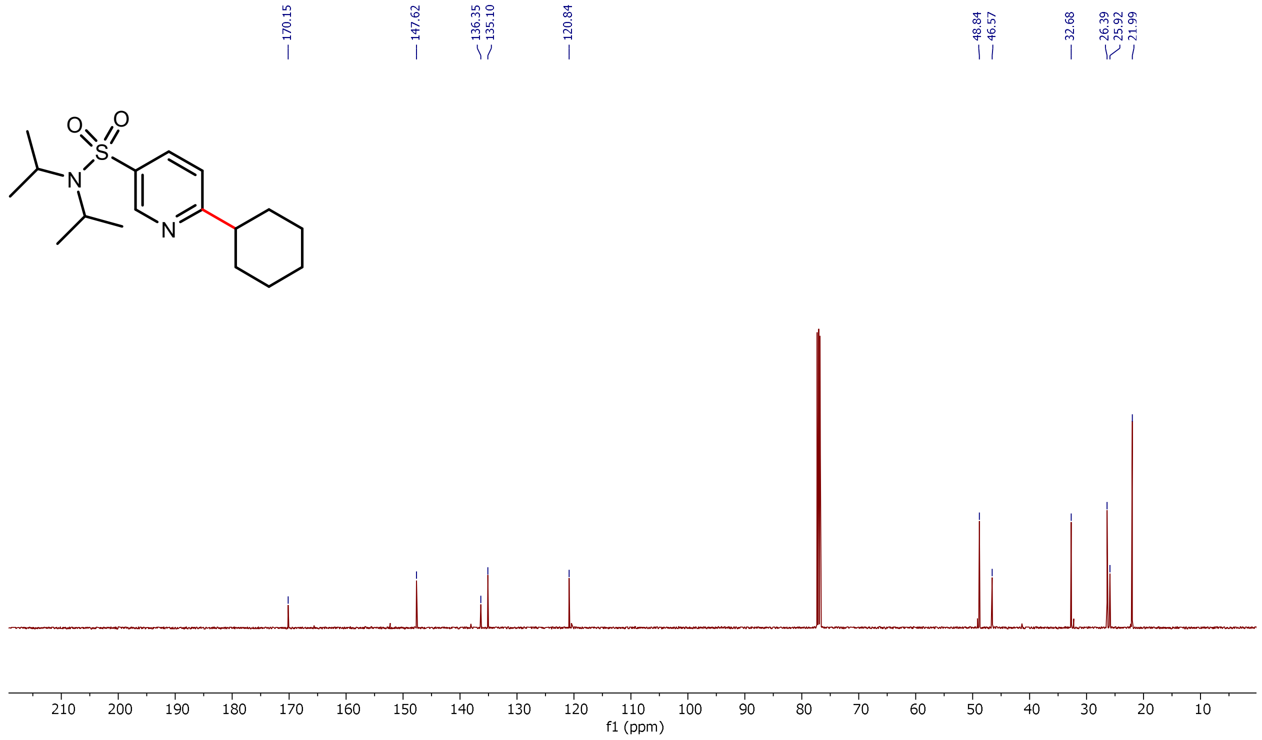

**Supplementary Figure 59a | $^{1}$H NMR (500 MHz, $CDCl_3$) of ethyl 2-cyclohexylisonicotinate (52)**

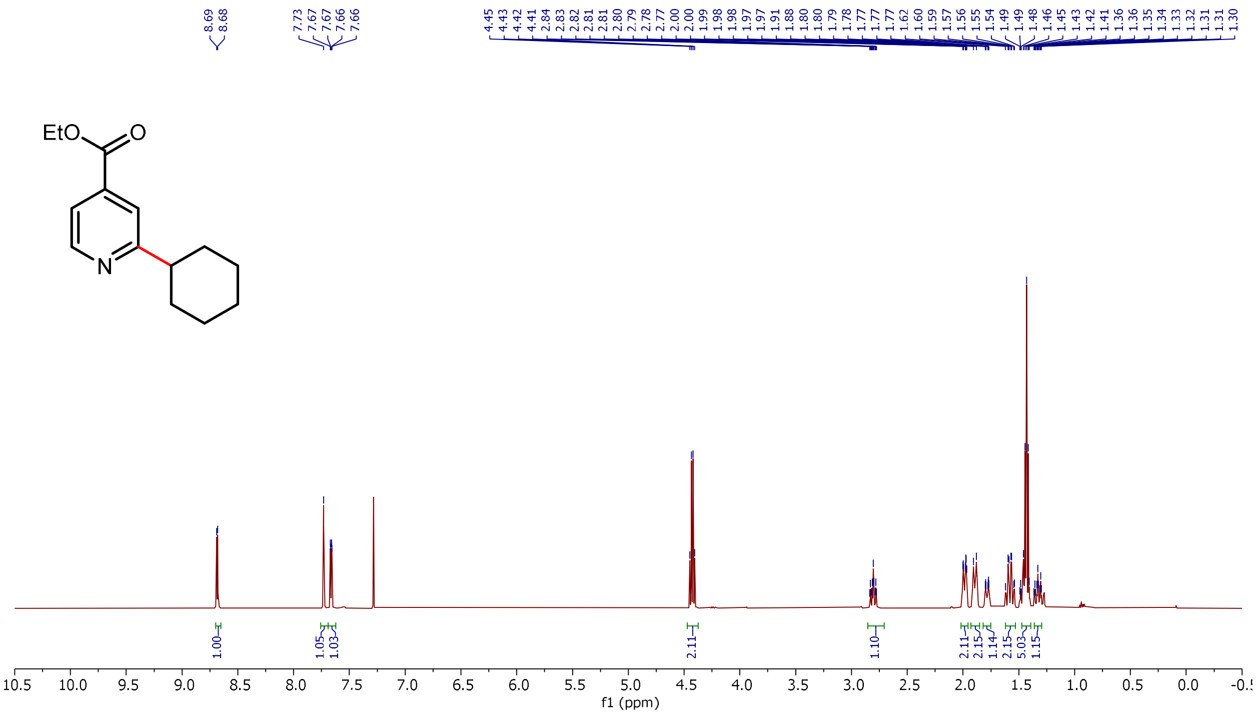

**Supplementary Figure 59b | $^{13}$C NMR (125 MHz, $CDCl_3$) of ethyl 2-cyclohexylisonicotinate (52)**

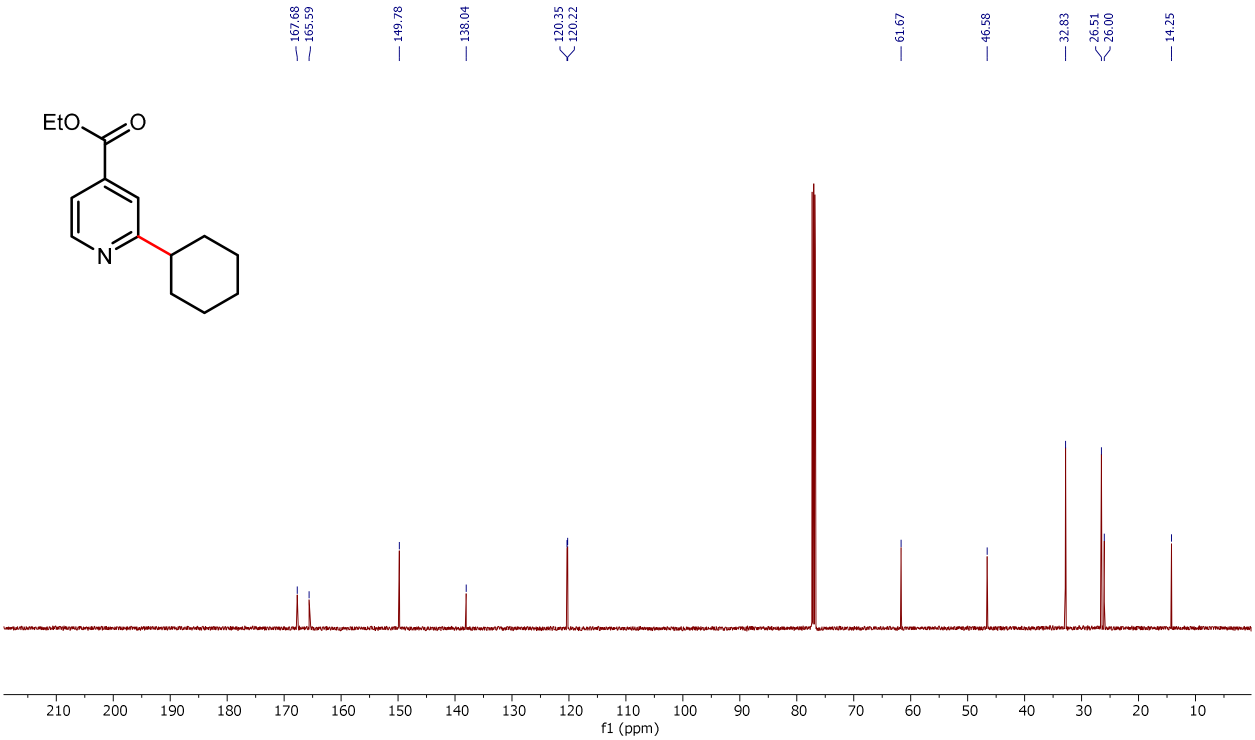

**Supplementary Figure 60a | $^{1}$H NMR (500 MHz, $CDCl_3$) of 2-cyclohexyl-4-phenylpyridine (53)**

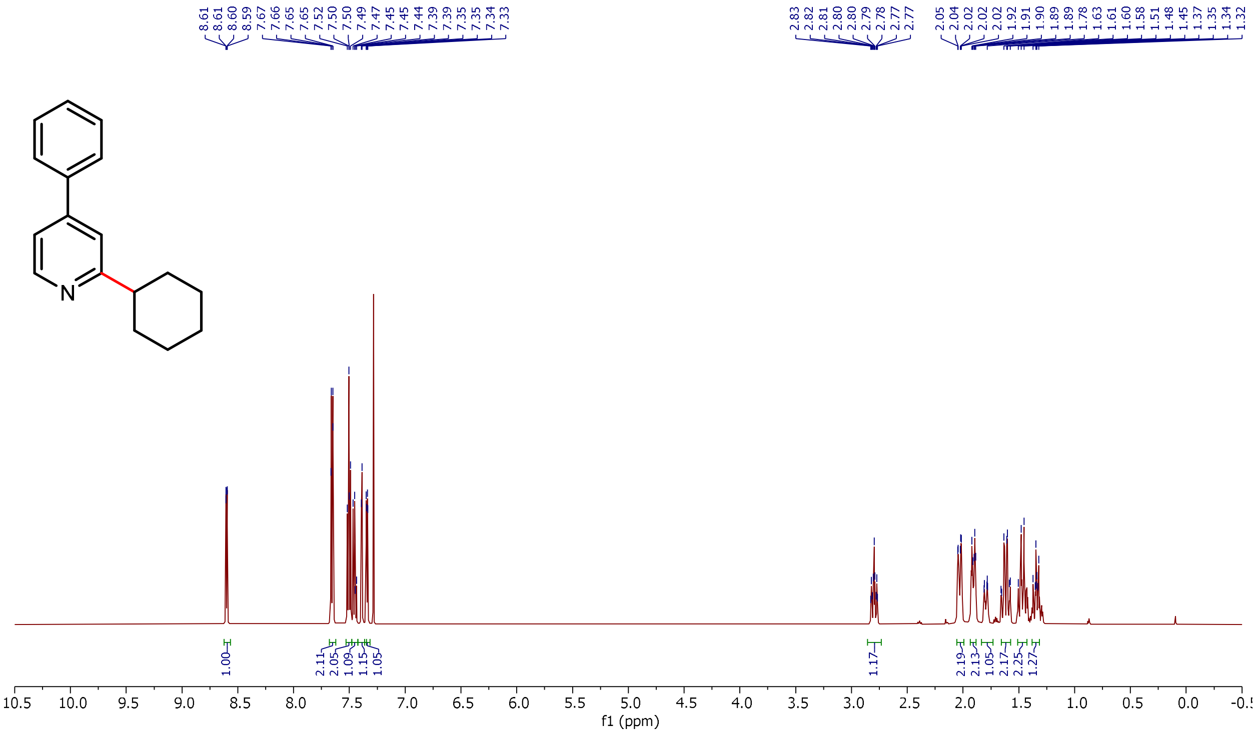


**Supplementary Figure 60b | $^{13}$C NMR (125 MHz, $CDCl_3$) of 2-cyclohexyl-4-phenylpyridine (53)**

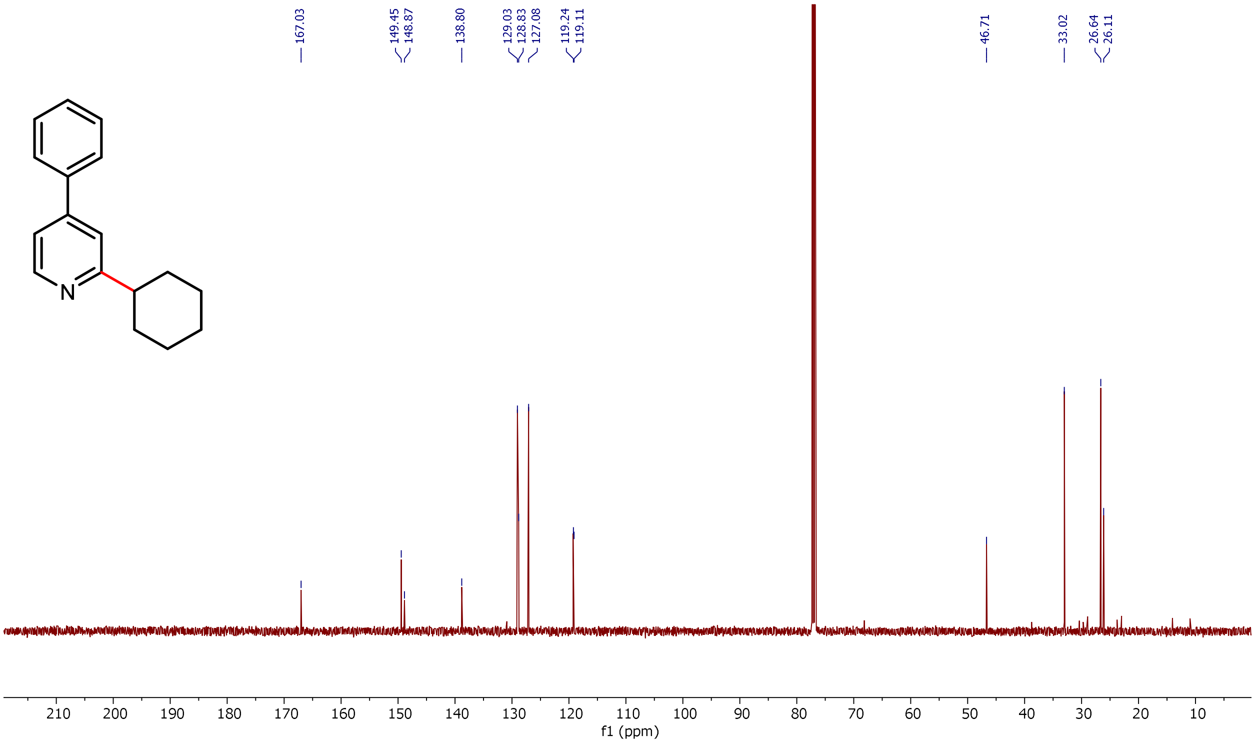

Supplementary Figure 61a | $^{1}$H NMR (500 MHz, $CDCl_3$) of 1-cyclohexyl-3-methylisoquinoline (54)

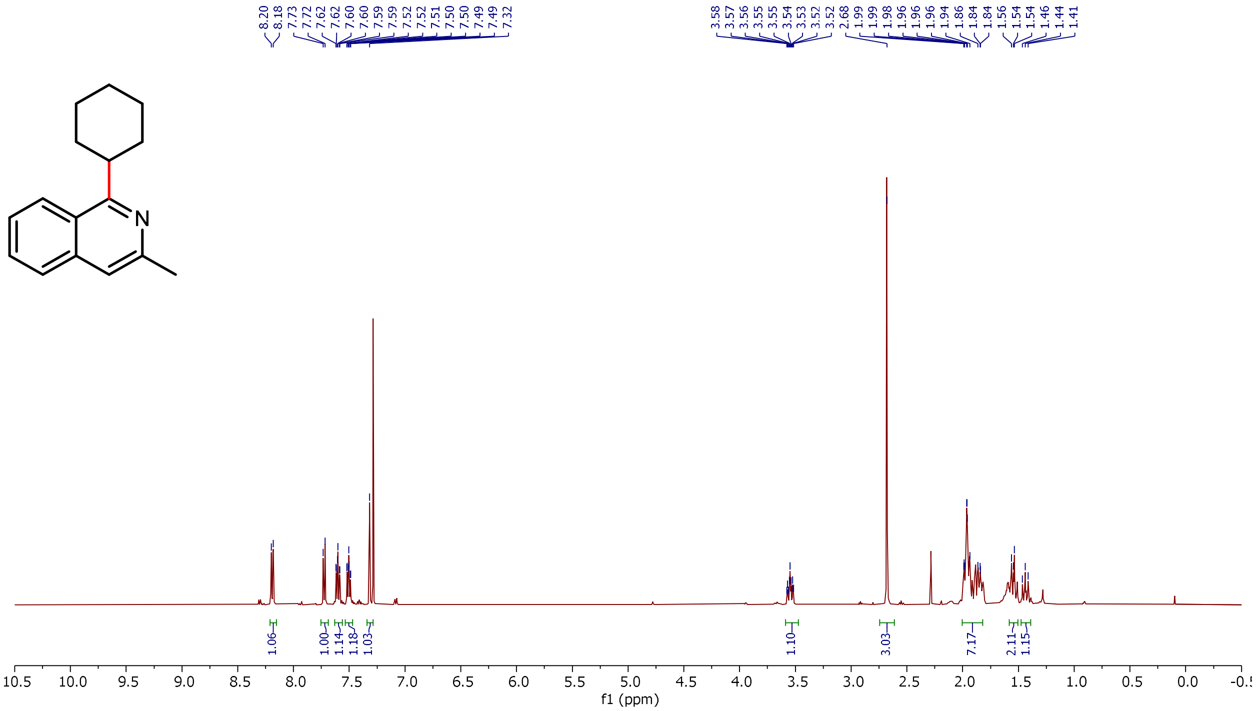

Supplementary Figure 61b | $^{13}$C NMR (125 MHz, $CDCl_3$) of 1-cyclohexyl-3-methylisoquinoline (54)

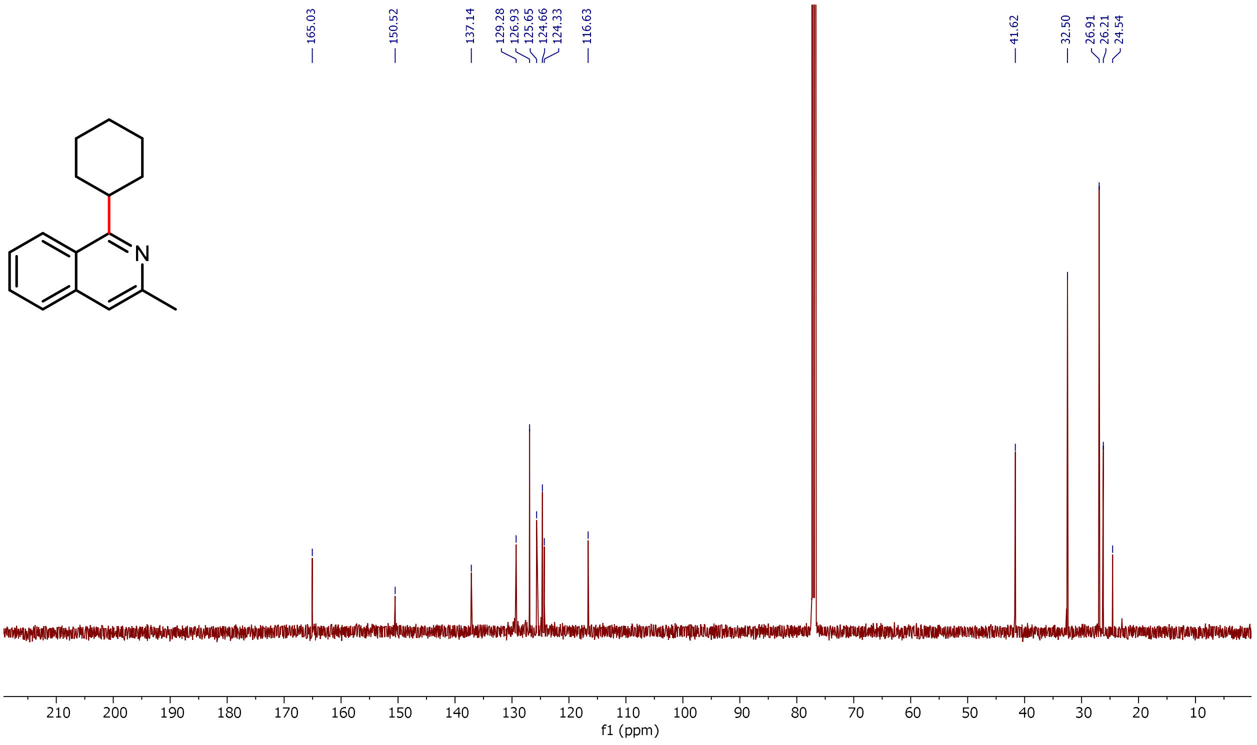

**Supplementary Figure 62a | $^{1}$H NMR (500 MHz, $CDCl_3$) of methyl 1-cyclohexylisoquinoline-3-carboxylate (55)**

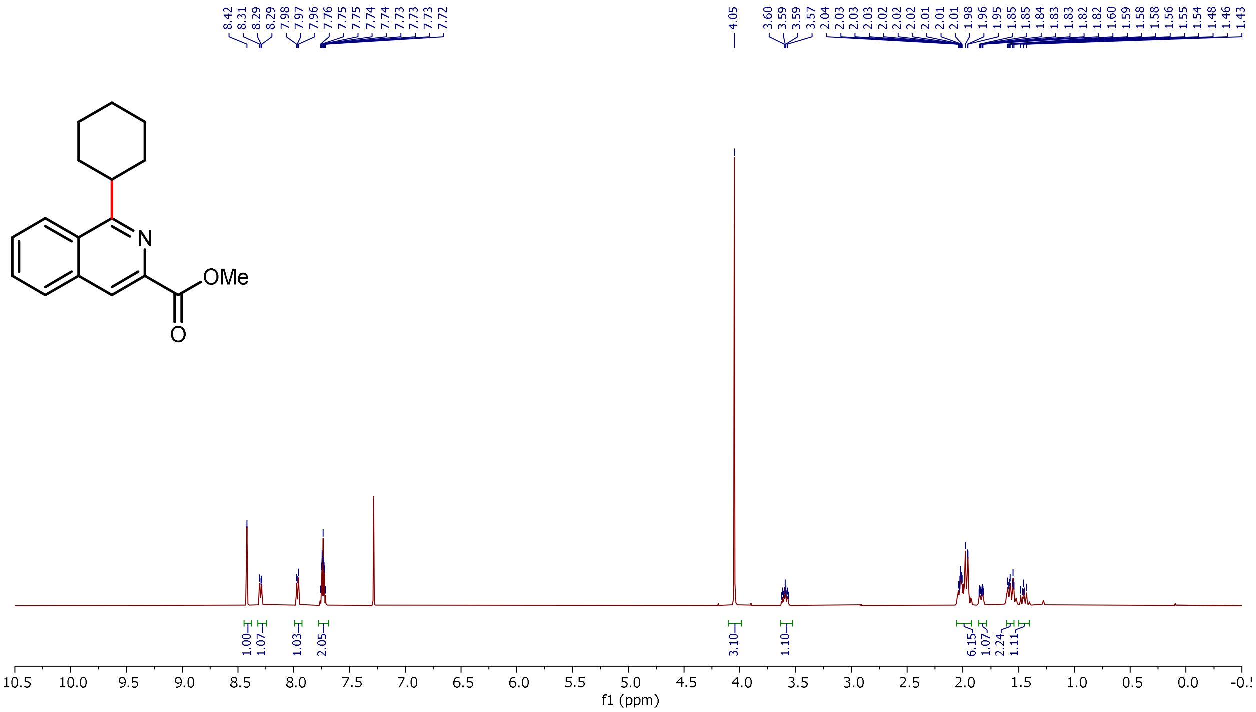


**Supplementary Figure 62b | $^{13}$C NMR (125 MHz, $CDCl_3$) of methyl 1-cyclohexylisoquinoline-3-carboxylate (55)**

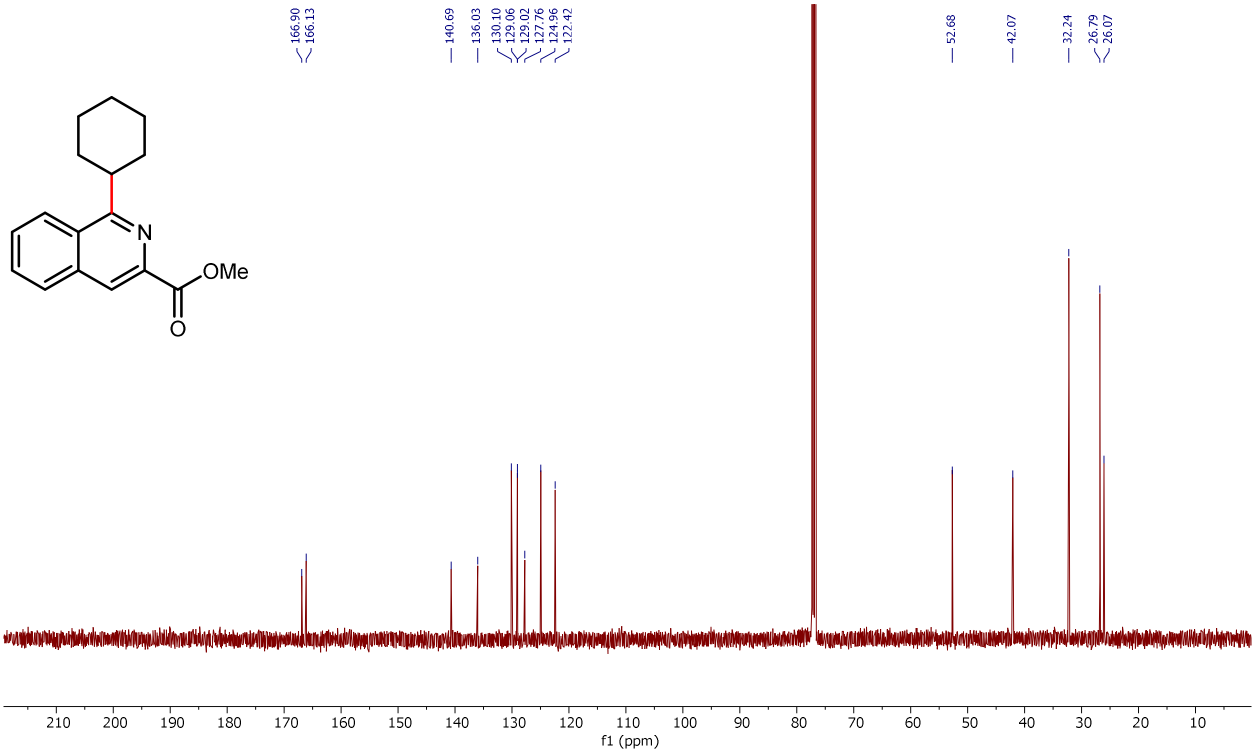

**Supplementary Figure 63a | $^{1}$H NMR (500 MHz, $CDCl_3$) of 1-cyclohexyl-5-nitroisoquinoline (56)**

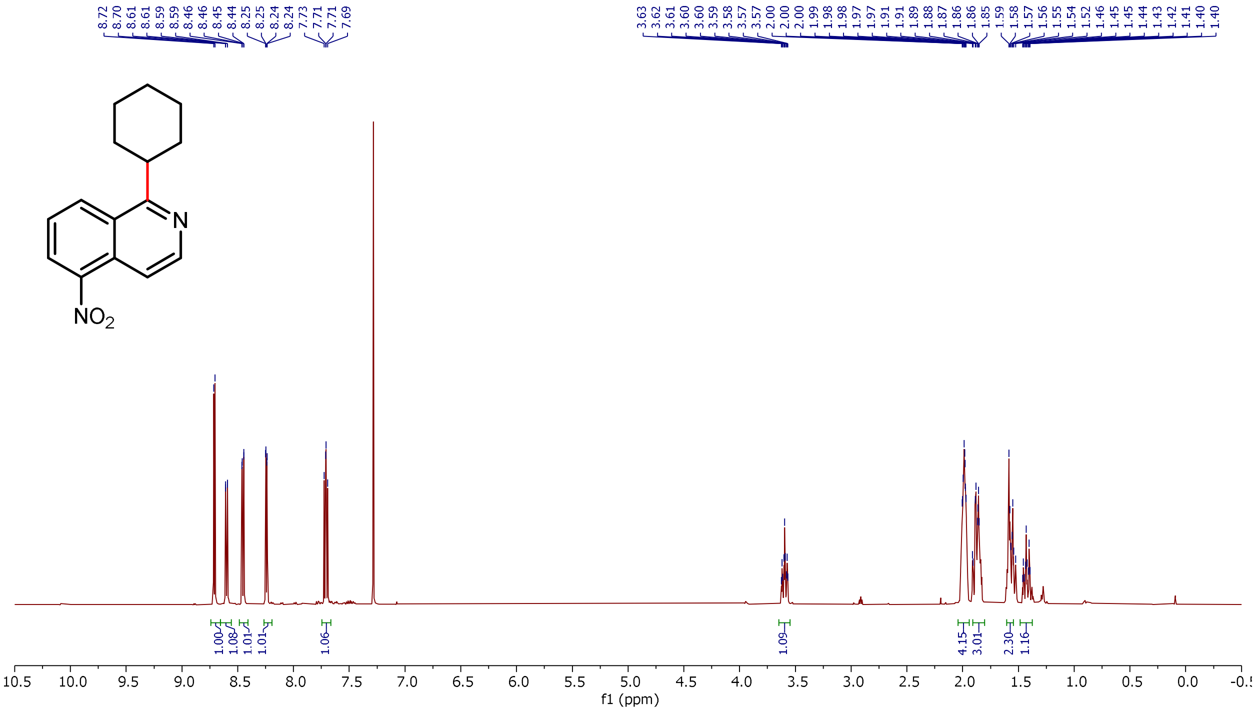


**Supplementary Figure 63b | $^{13}$C NMR (125 MHz, $CDCl_3$) of 1-cyclohexyl-5-nitroisoquinoline (56)**

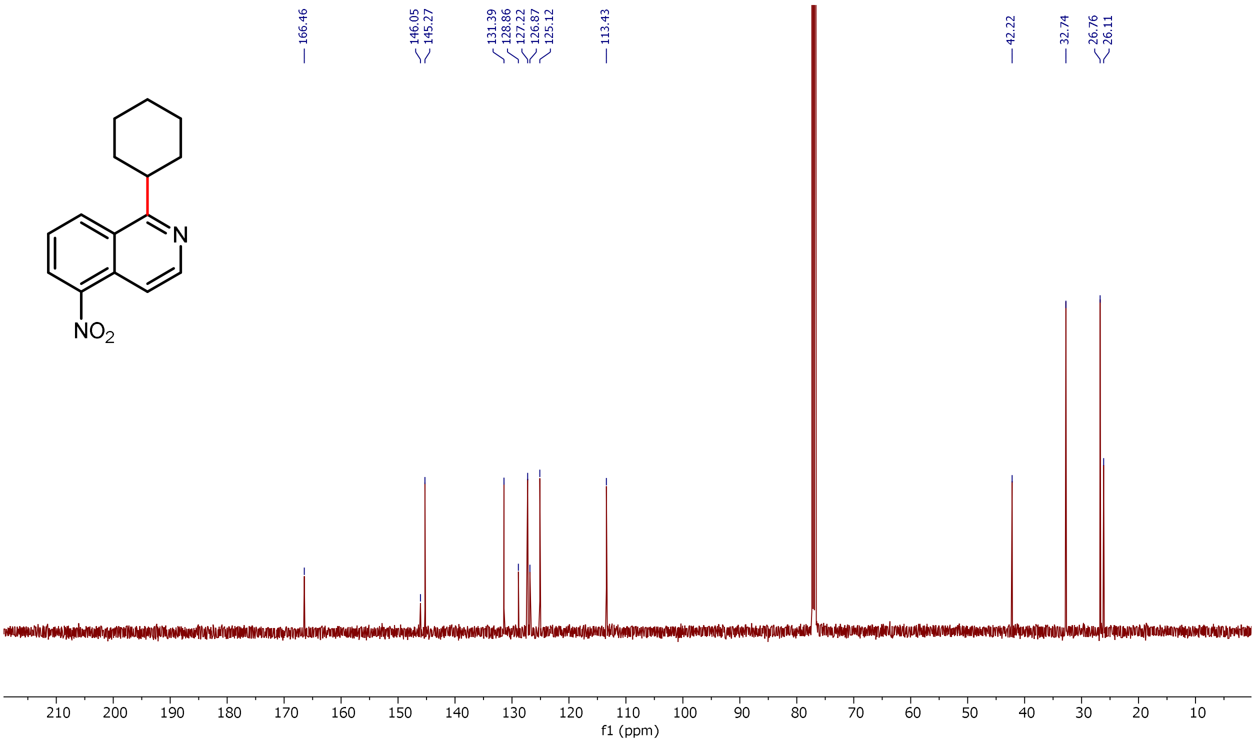

**Supplementary Figure 64a | $^1$H NMR (500 MHz, $CDCl_3$) of 4,6-dichloro-2-cyclohexylpyrimidine (57)**

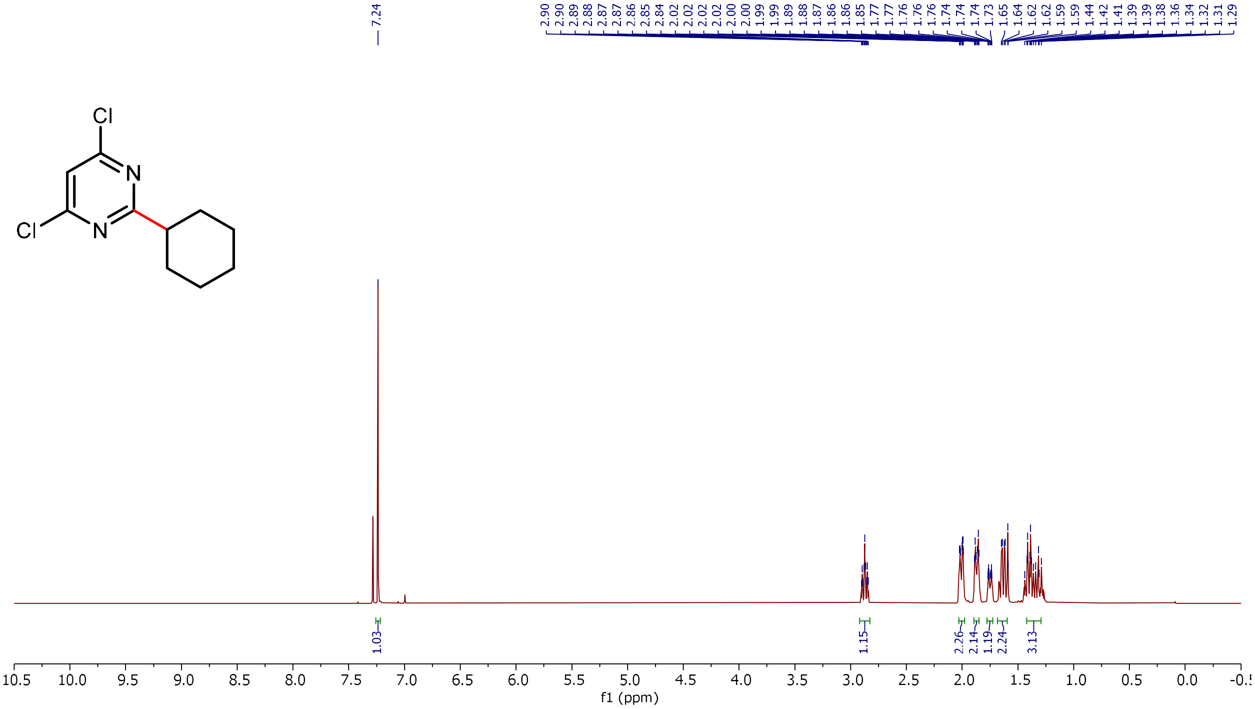

**Supplementary Figure 64b | $^{13}$C NMR (125 MHz, $CDCl_3$) of 4,6-dichloro-2-cyclohexylpyrimidine (57)**

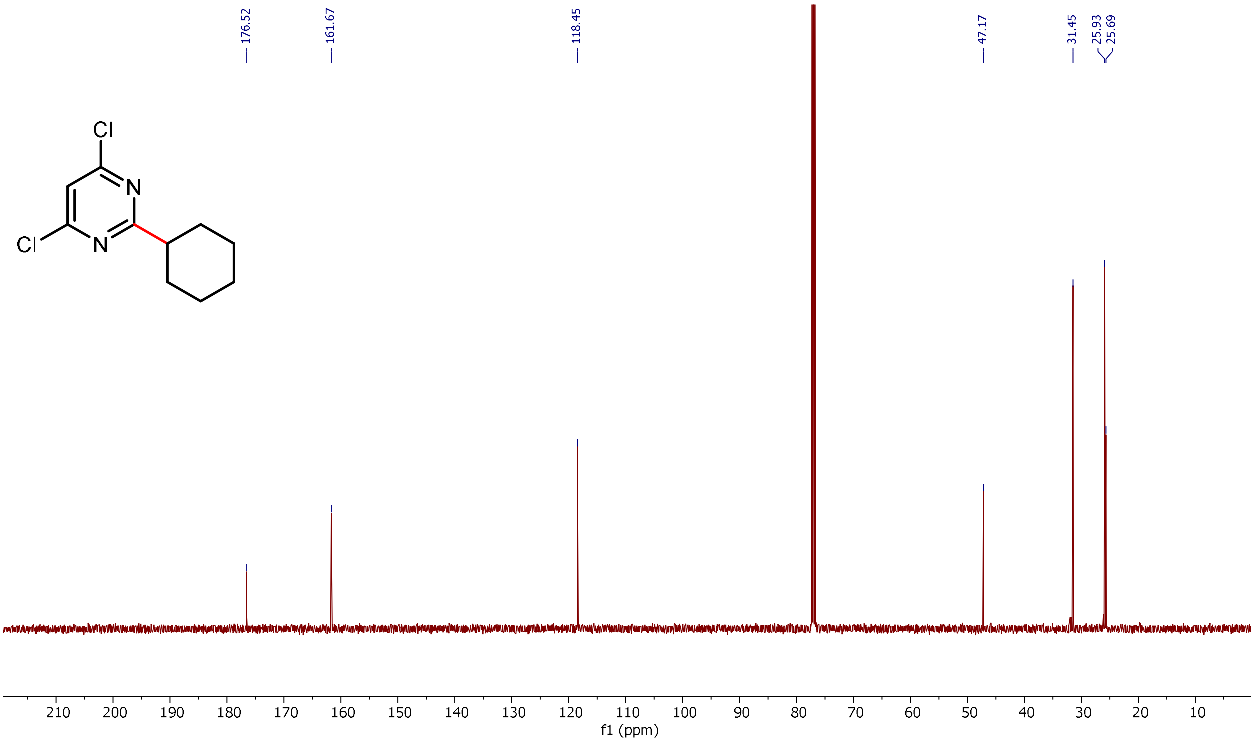

Supplementary Figure 65a | $^{1}$H NMR (500 MHz, $CDCl_3$) of 2-chloro-4-cyclohexylpyrimidine (58a)

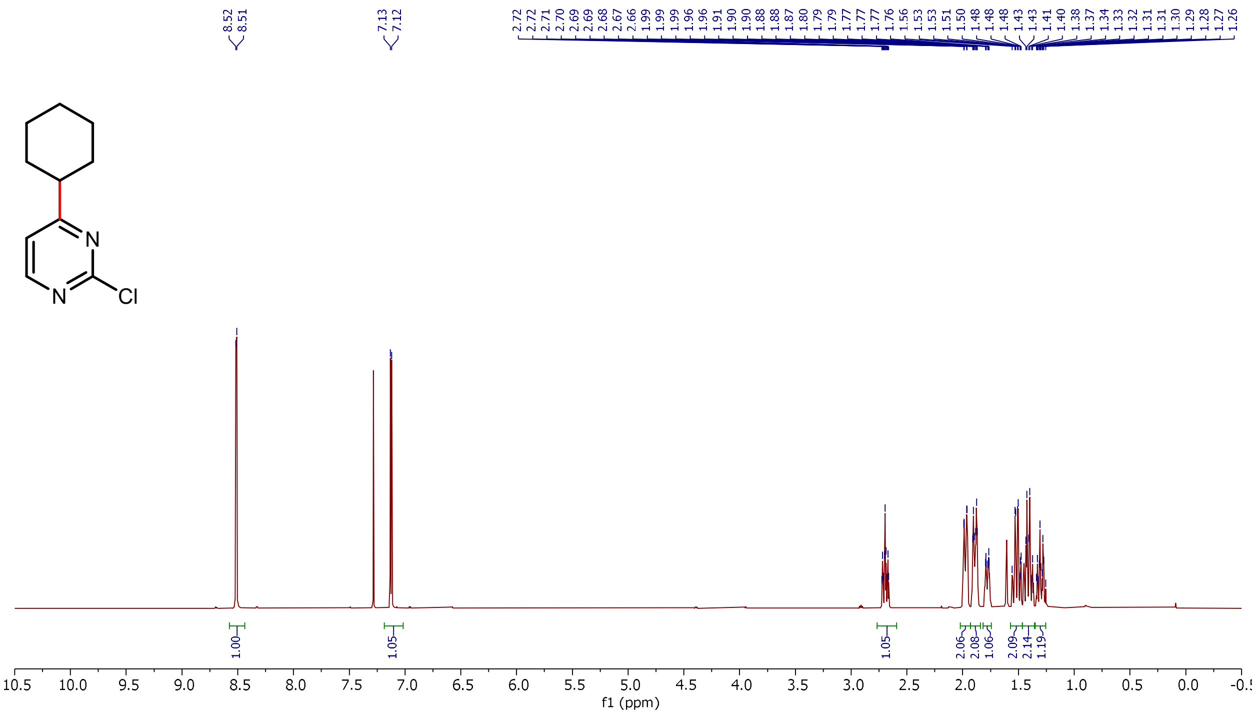

Supplementary Figure 65b | $^{13}$C NMR (125 MHz, $CDCl_3$) of 2-chloro-4-cyclohexylpyrimidine (58a)

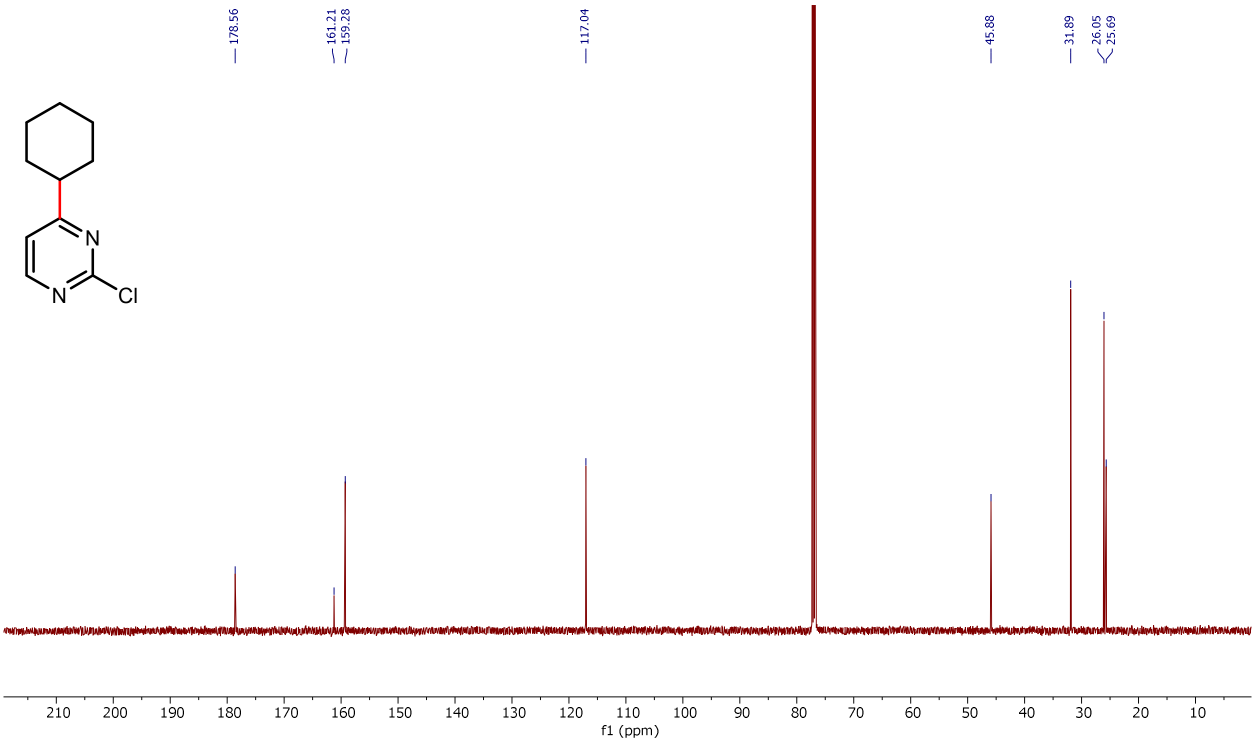

Supplementary Figure 66a | $^{1}$H NMR (500 MHz, $CDCl_3$) of 2-chloro-4,6-dicyclohexylpyrimidine (58b)

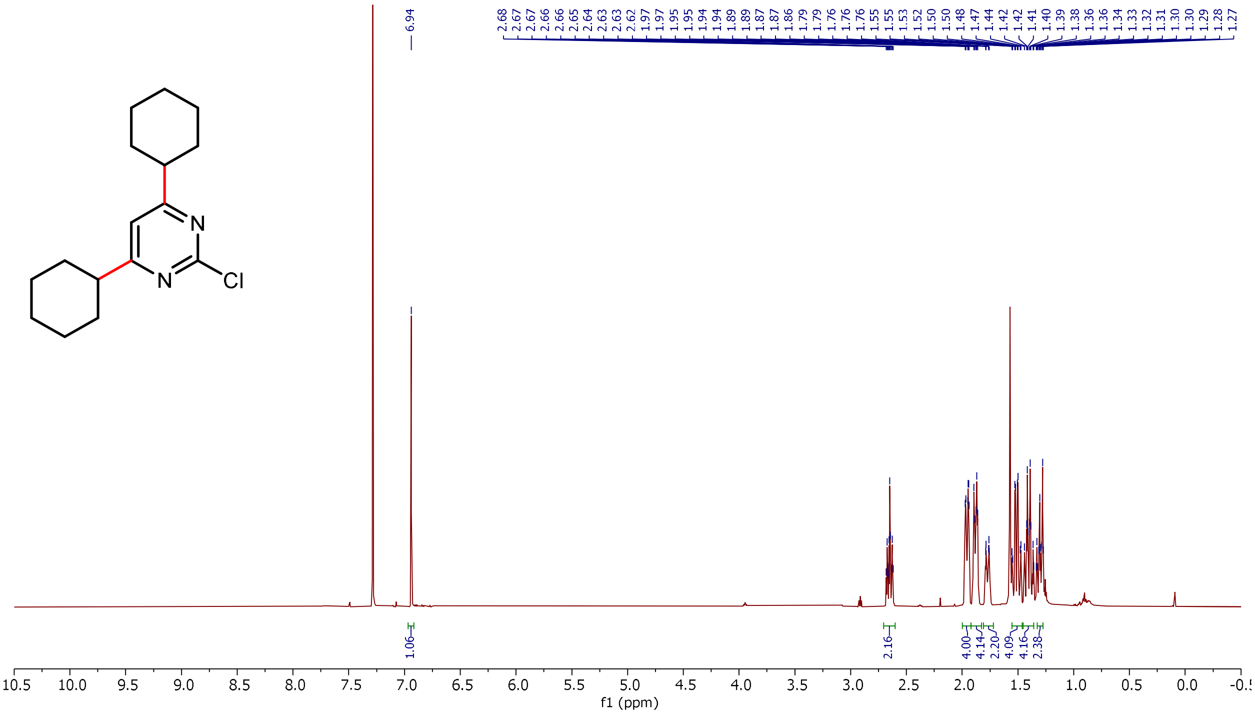

Supplementary Figure 66b | $^{13}$C NMR (125 MHz, $CDCl_3$) of 2-chloro-4,6-dicyclohexylpyrimidine (58b)

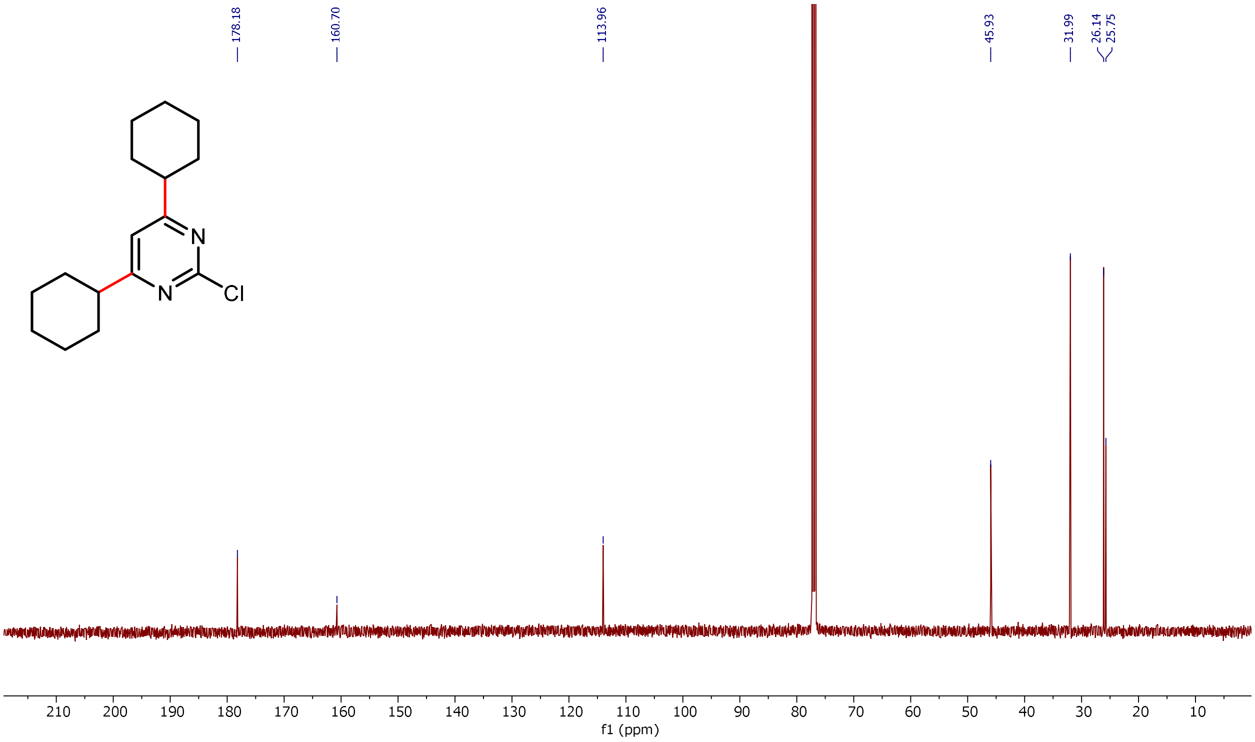

**Supplementary Figure 67a | $^1$H NMR (500 MHz, CDCl$_3$) of 5-cyclohexylpyrazine-2,3-dicarbonitrile (59)**

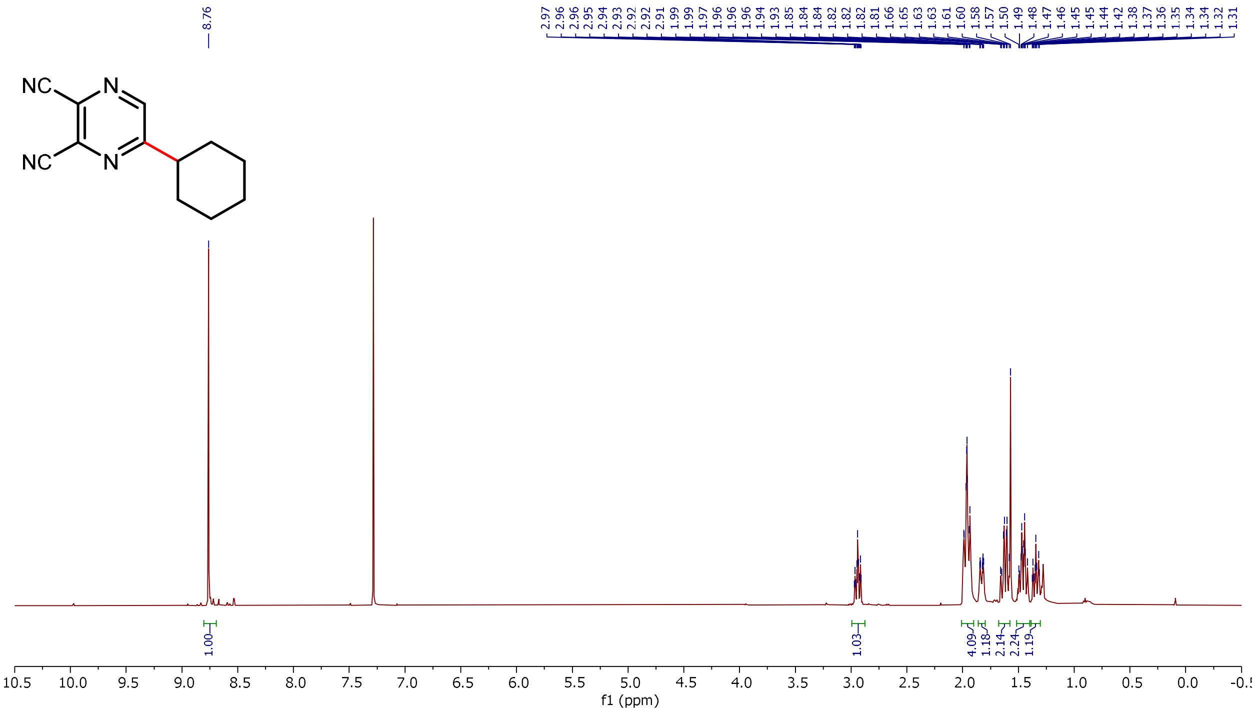


**Supplementary Figure 67b | $^{13}$C NMR (125 MHz, CDCl$_3$) of 5-cyclohexylpyrazine-2,3-dicarbonitrile (59)**

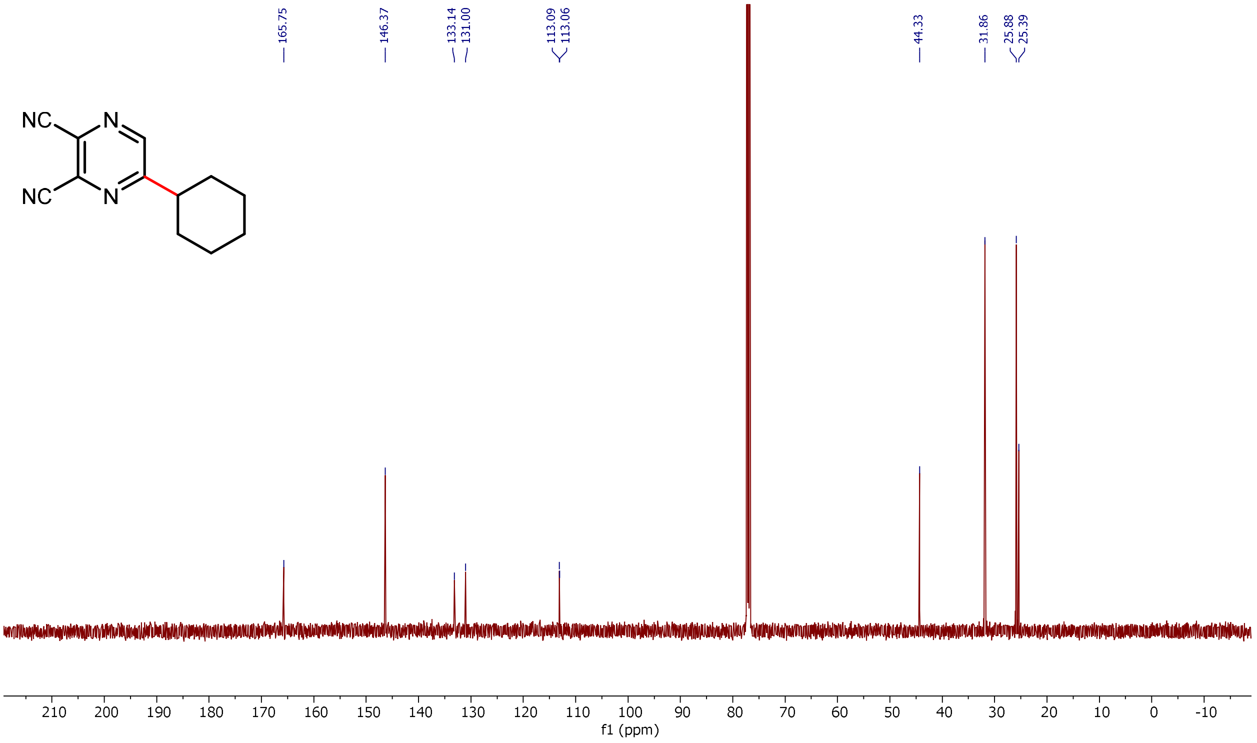

**Supplementary Figure 68a | $^{1}$H NMR (500 MHz, $CDCl_3$) of 3-cyclohexyl-1-methylquinoxalin-2(1*H*)-one (60)**

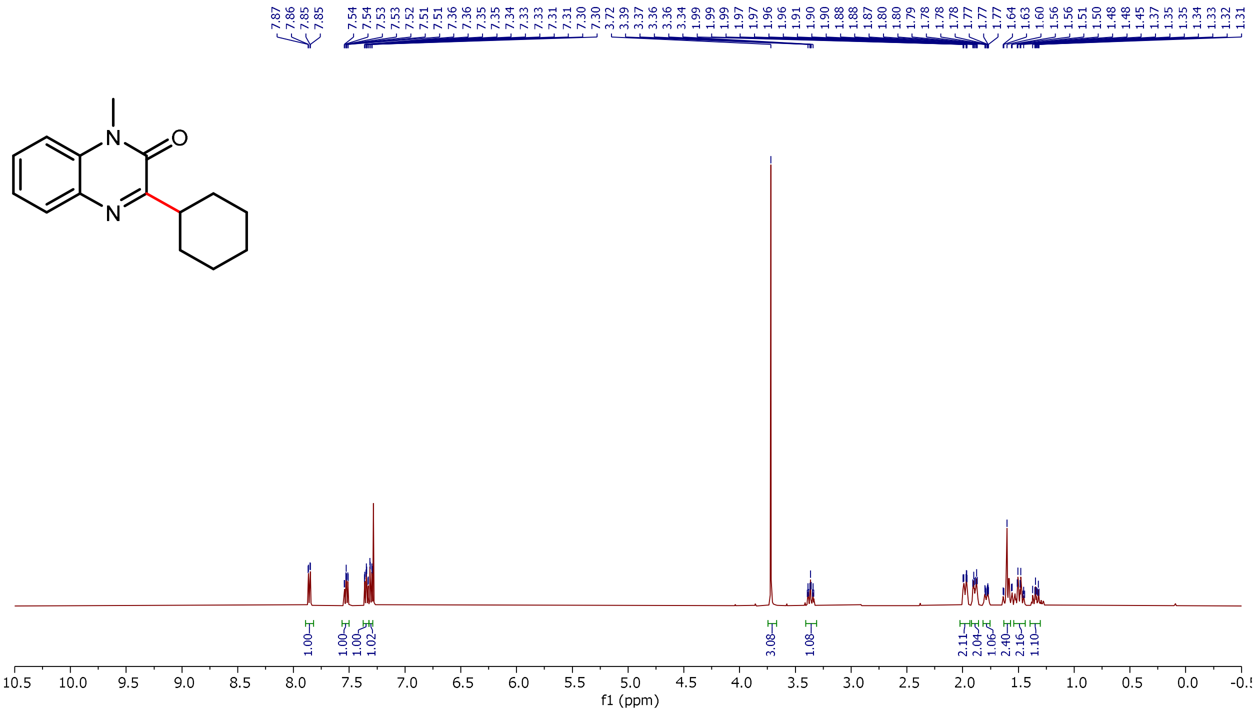


**Supplementary Figure 68b | $^{13}$C NMR (125 MHz, $CDCl_3$) of 3-cyclohexyl-1-methylquinoxalin-2(1*H*)-one (60)**

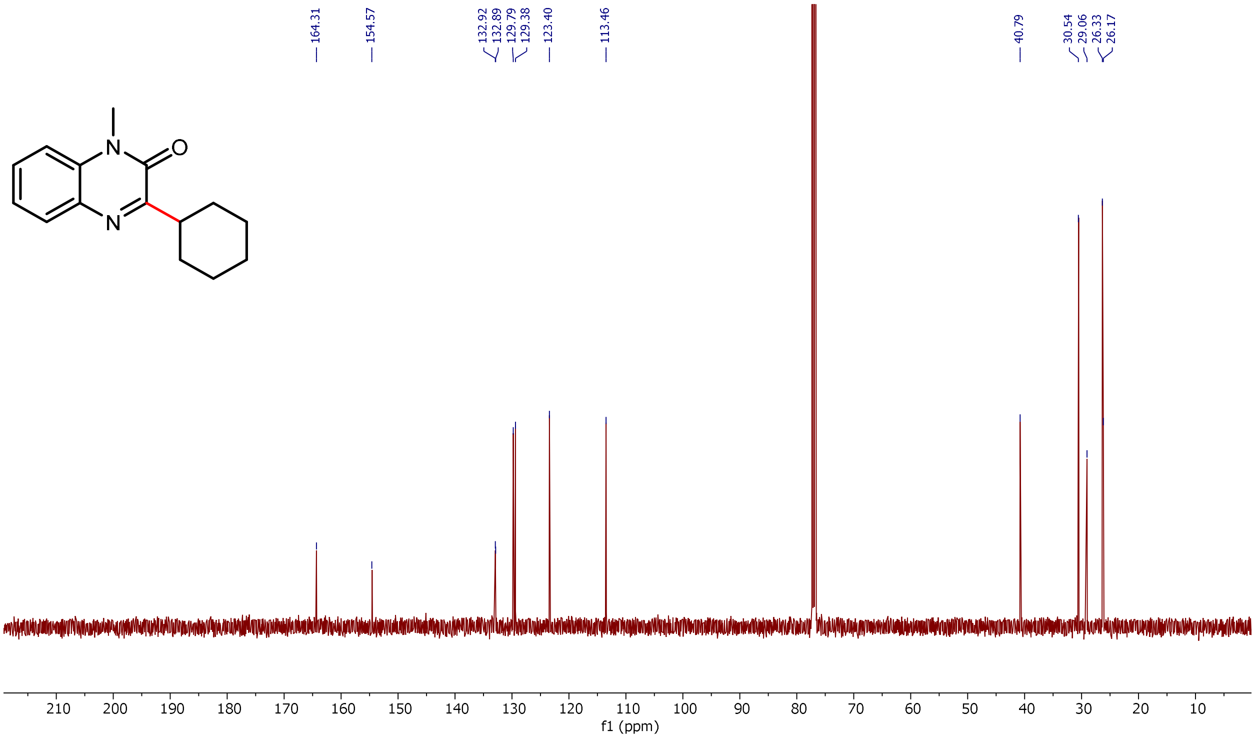

**Supplementary Figure 69a | $^{1}$H NMR (500 MHz, $CDCl_3$) of 8-cyclohexyl-1,3,7-trimethyl-3,7-dihydro-1*H*-purine-2,6-dione (61)**

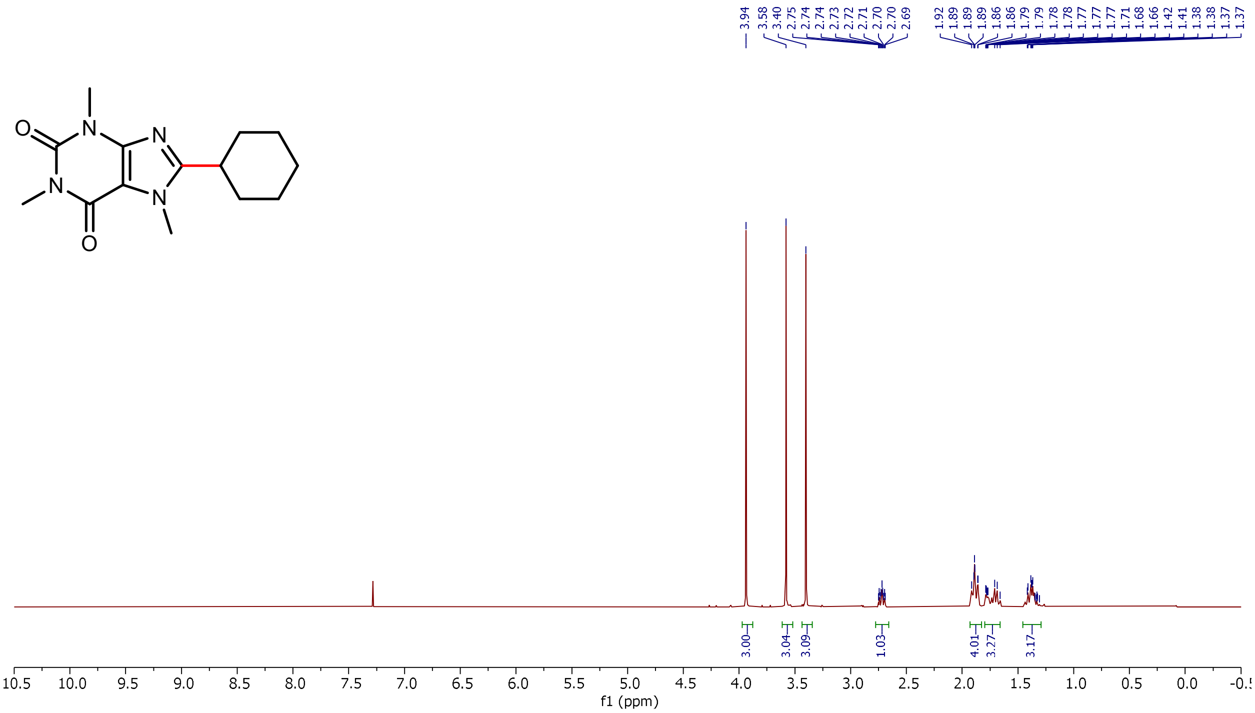

**Supplementary Figure 69b | $^{13}$C NMR (125 MHz, $CDCl_3$) of 8-cyclohexyl-1,3,7-trimethyl-3,7-dihydro-1*H*-purine-2,6-dione (61)**

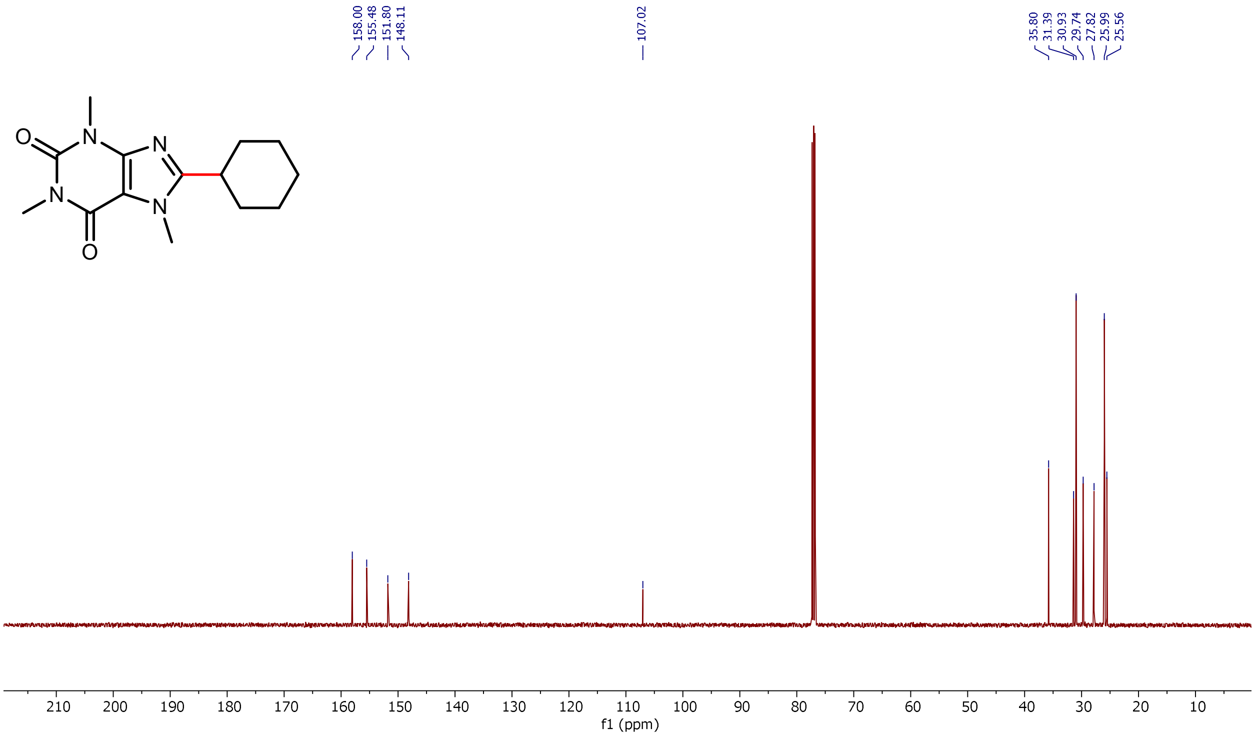

**Supplementary Figure 70a | $^{1}$H NMR (500 MHz, CDCl$_3$) of 8-cyclohexyl-7-methyl-1-(4-oxopentyl)-3,7-dihydro-1*H*-purine-2,6-dione (62)**

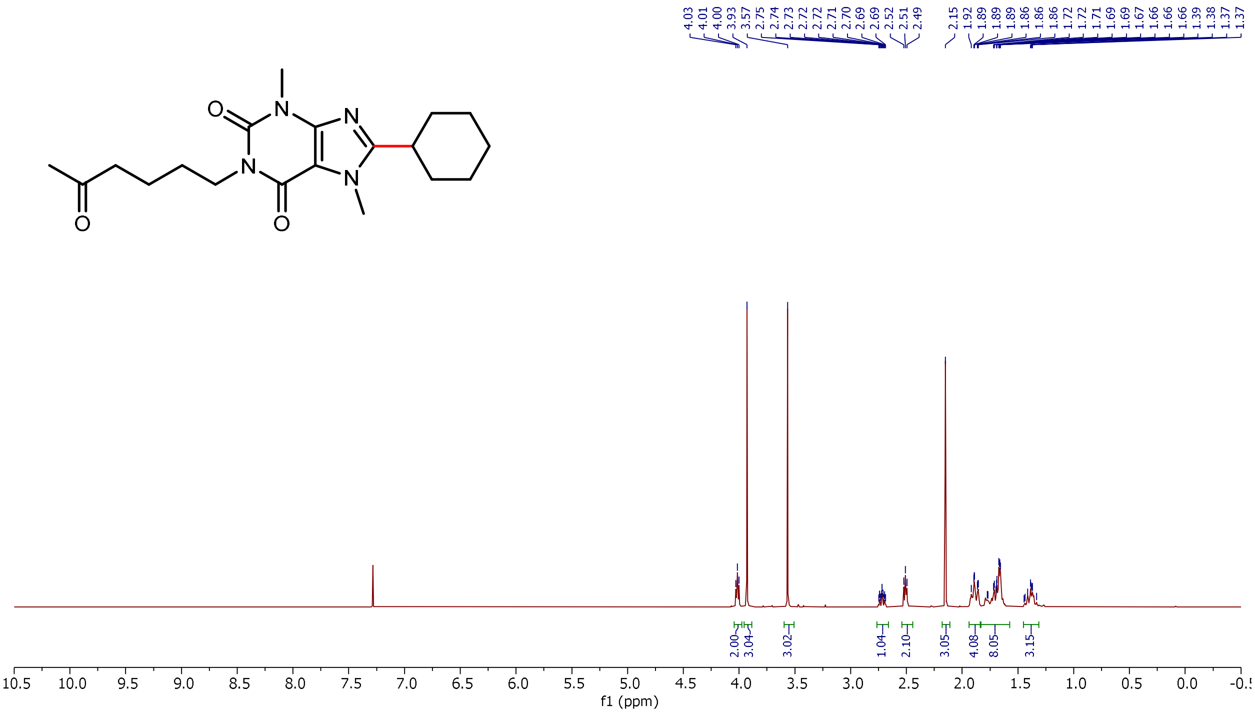


**Supplementary Figure 70b | $^{13}$C NMR (125 MHz, CDCl$_3$) of 8-cyclohexyl-7-methyl-1-(4-oxopentyl)-3,7-dihydro-1*H*-purine-2,6-dione (62)**

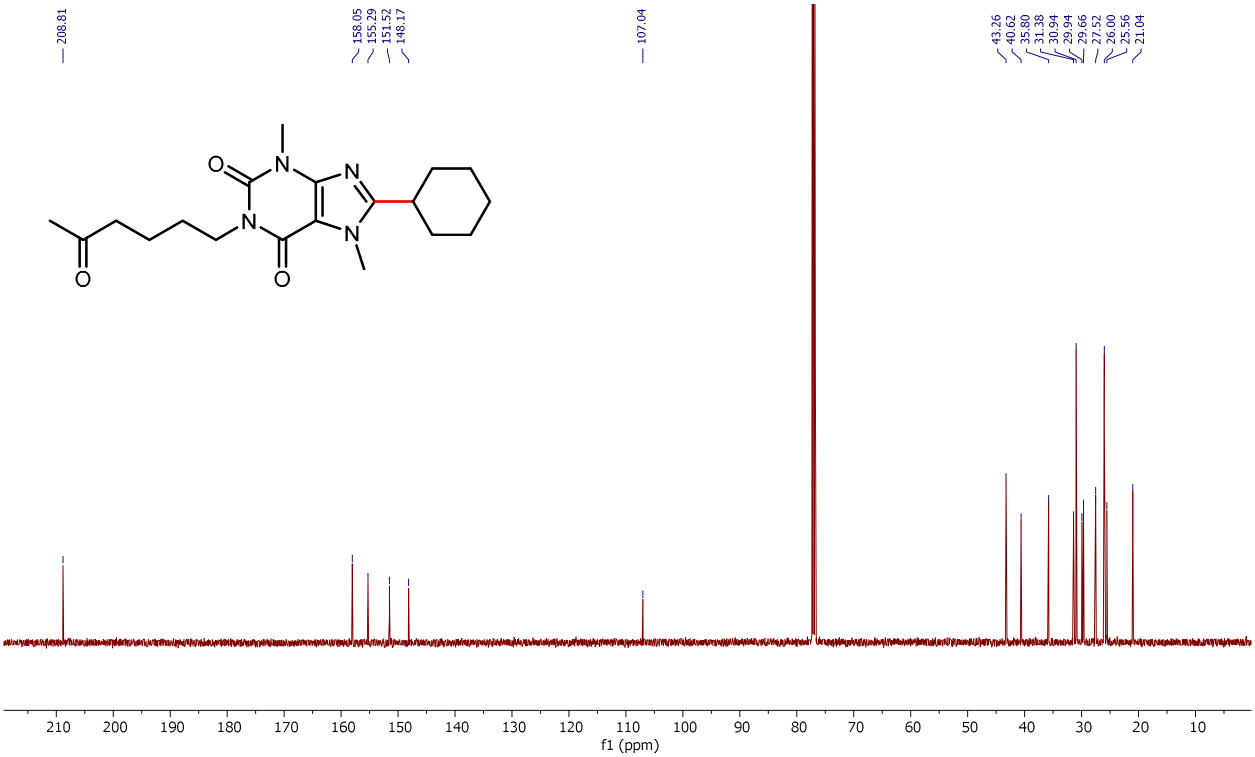

**Supplementary Figure 71a | $^1$H NMR (500 MHz, CDCl$_3$) of 2,6-dichloro-8-cyclohexyl-9-methyl-9*H*-purine (63)**

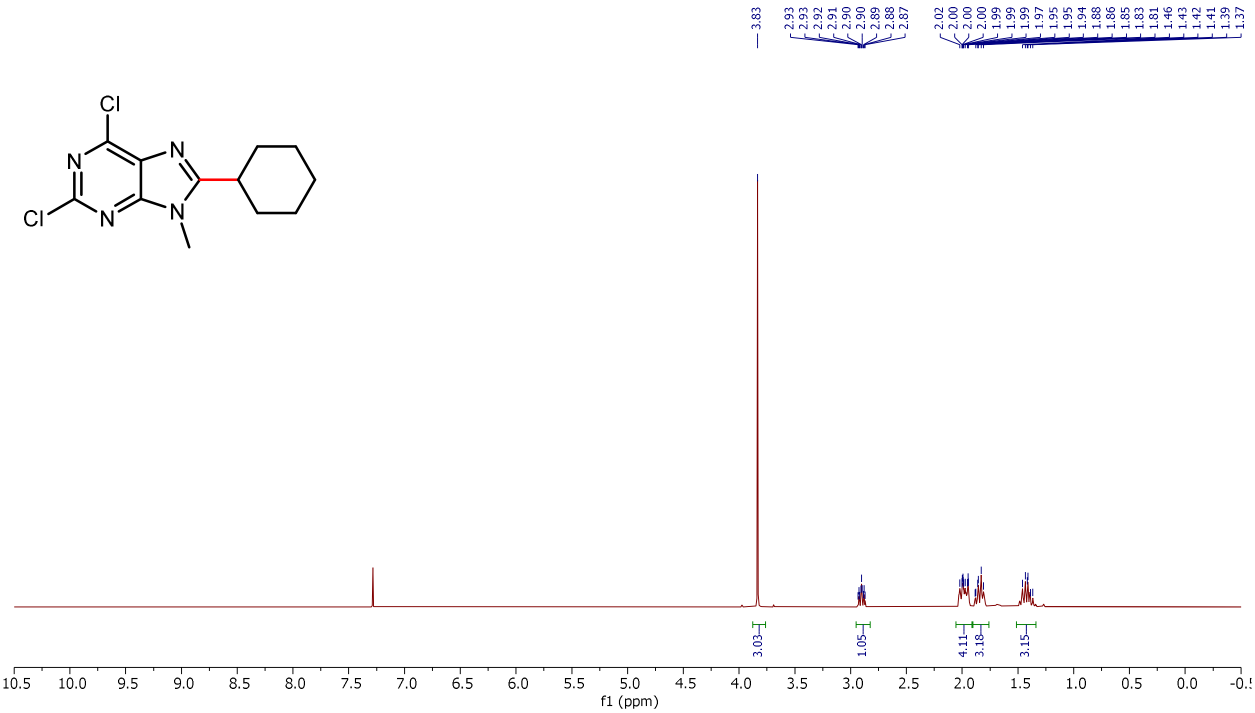


**Supplementary Figure 71b | $^{13}$C NMR (125 MHz, CDCl$_3$) of 2,6-dichloro-8-cyclohexyl-9-methyl-9*H*-purine (63)**

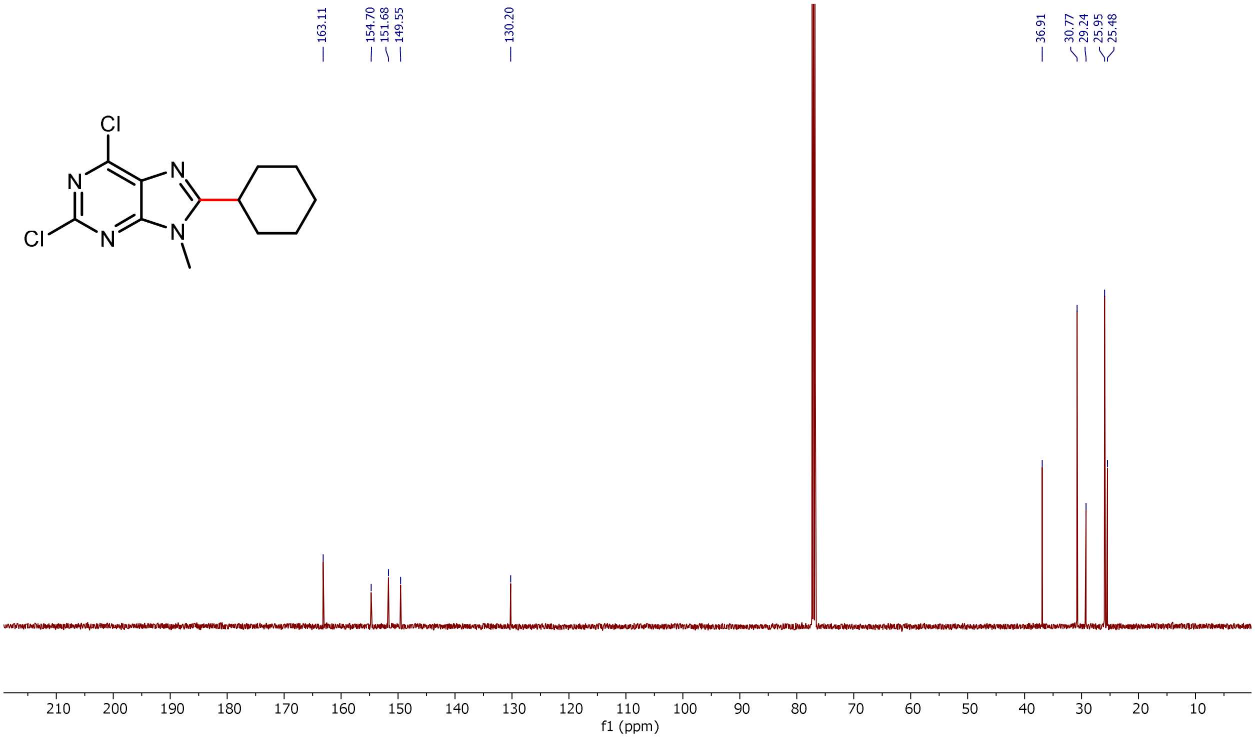

**Supplementary Figure 72a | $^{1}$H NMR (500 MHz, $CDCl_3$) of (*R*)-methyl-2-(2-cyclohexylisonicotinamido)propanoate (64a)**

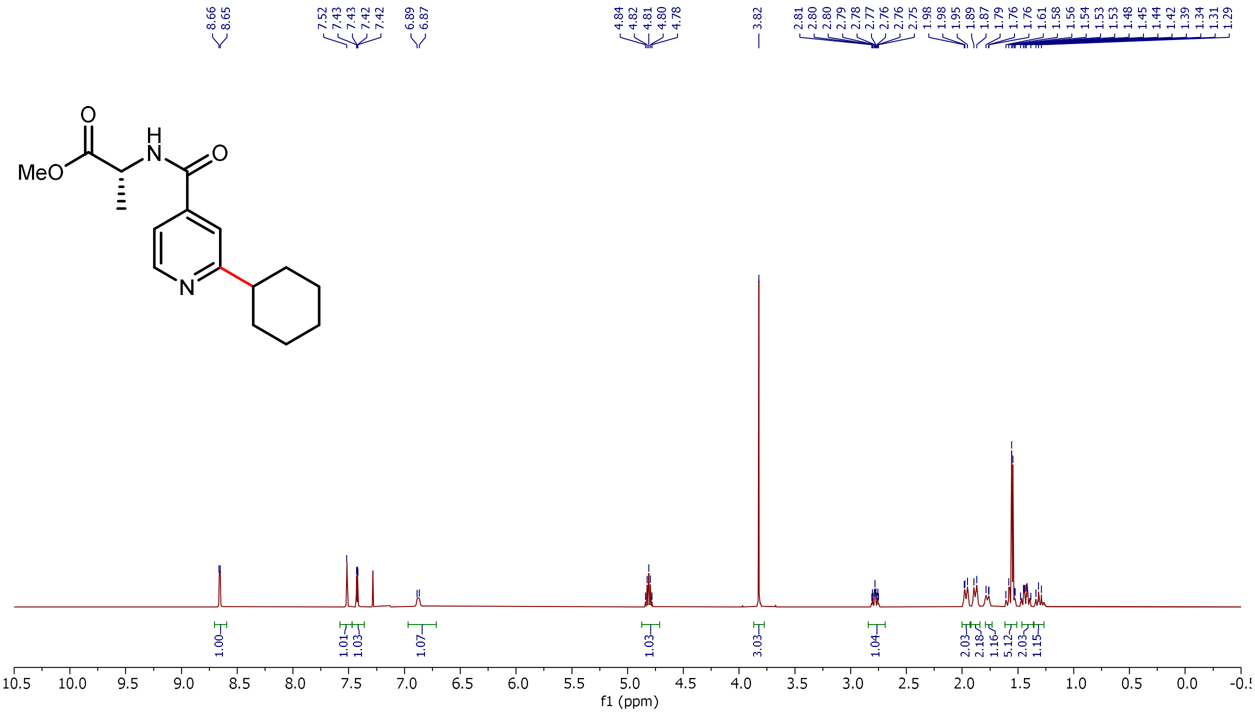

**Supplementary Figure 72b | $^{13}$C NMR (125 MHz, $CDCl_3$) of (*R*)-methyl-2-(2-cyclohexylisonicotinamido)propanoate (64a)**

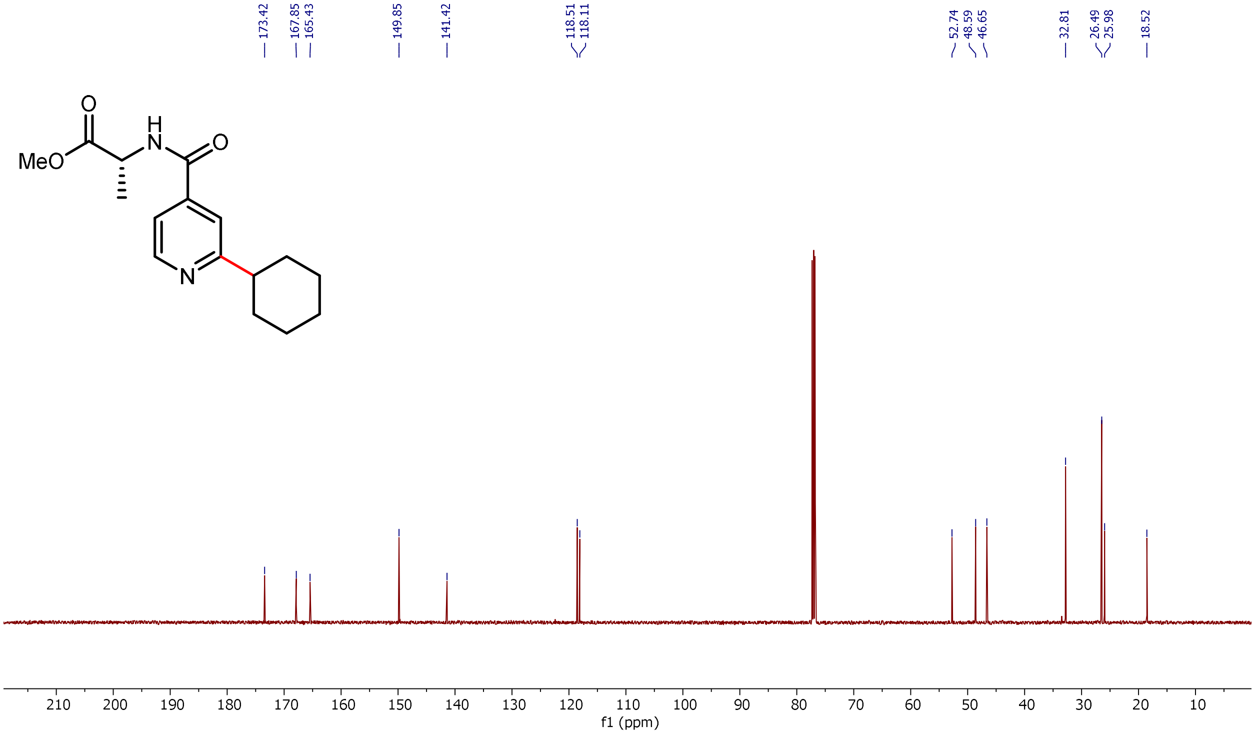

**Supplementary Figure 73a | $^{1}H$ NMR (500 MHz, $CDCl_3$) of (*R*)-methyl-2-(2,6-dicyclohexylisonicotinamido)propanoate (64b)**

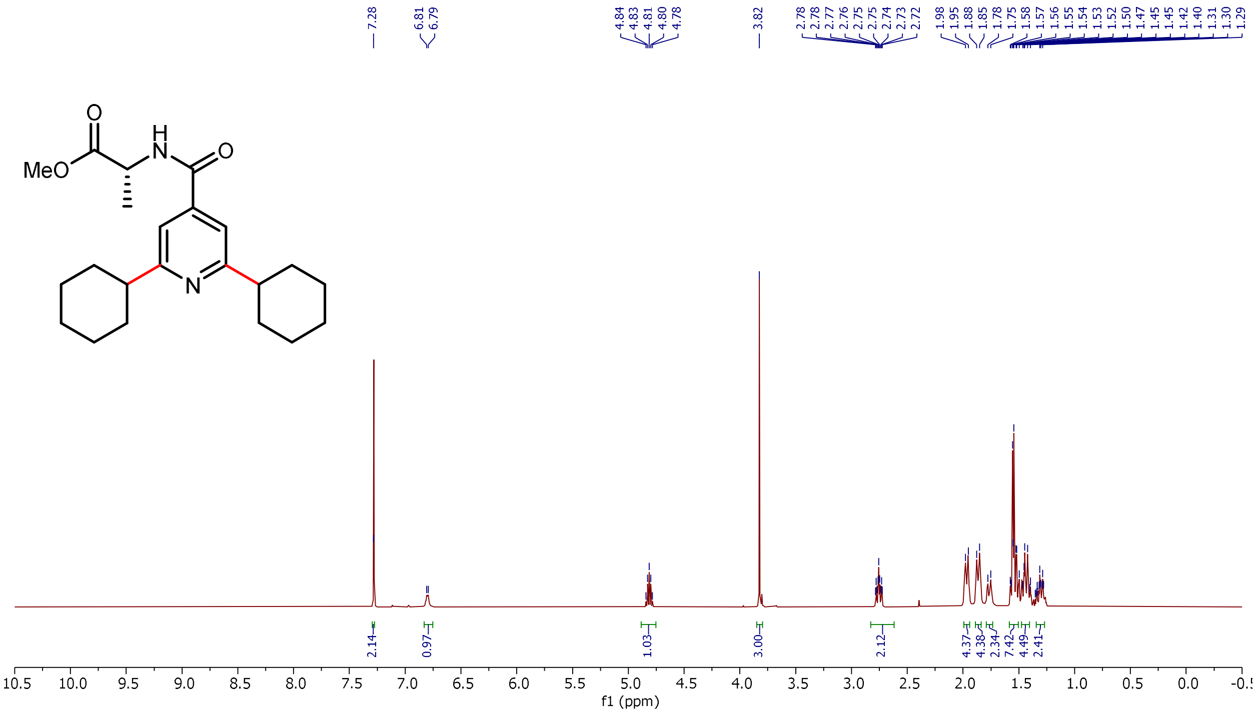


**Supplementary Figure 73b | $^{13}C$ NMR (125 MHz, $CDCl_3$) of (*R*)-methyl-2-(2,6-dicyclohexylisonicotinamido)propanoate (64b)**

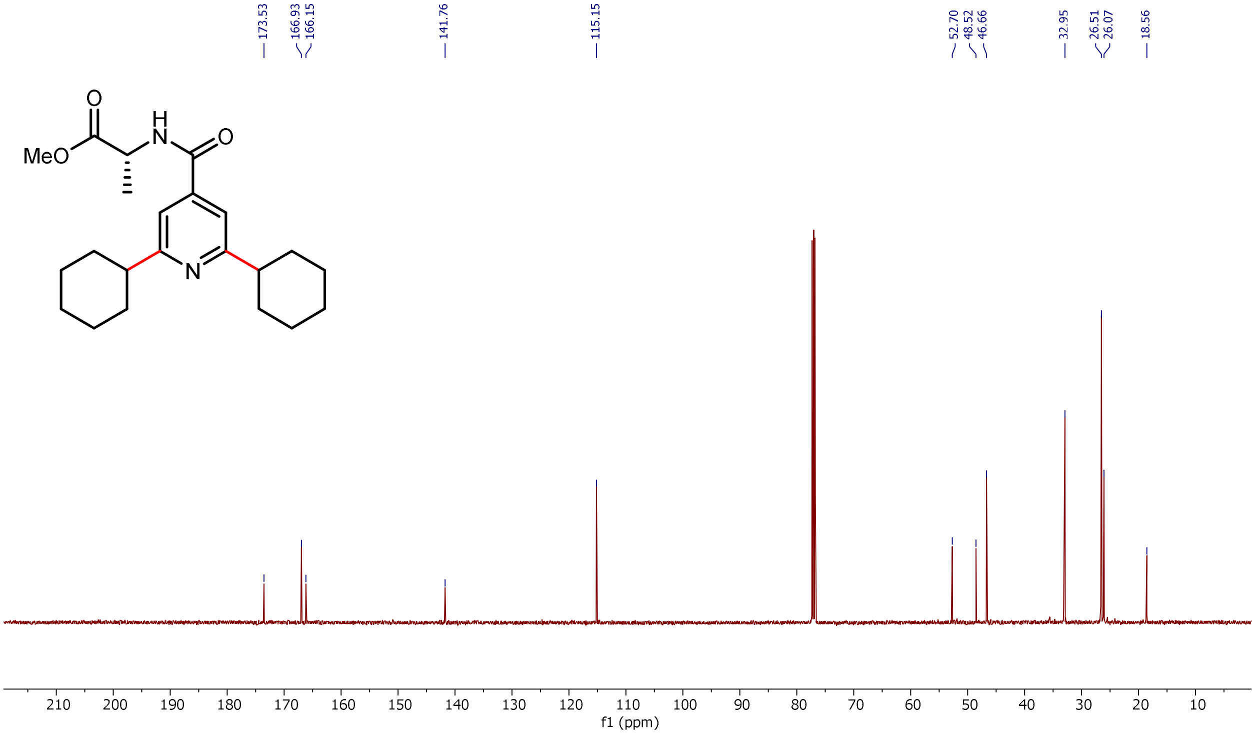

**Supplementary Figure 74a | $^{1}$H NMR (500 MHz, $CDCl_3$) of (1*R*,2*S*,5*R*)-2-isopropyl-5-methylcyclohexyl 2-cyclohexylisonicotinate (65)**

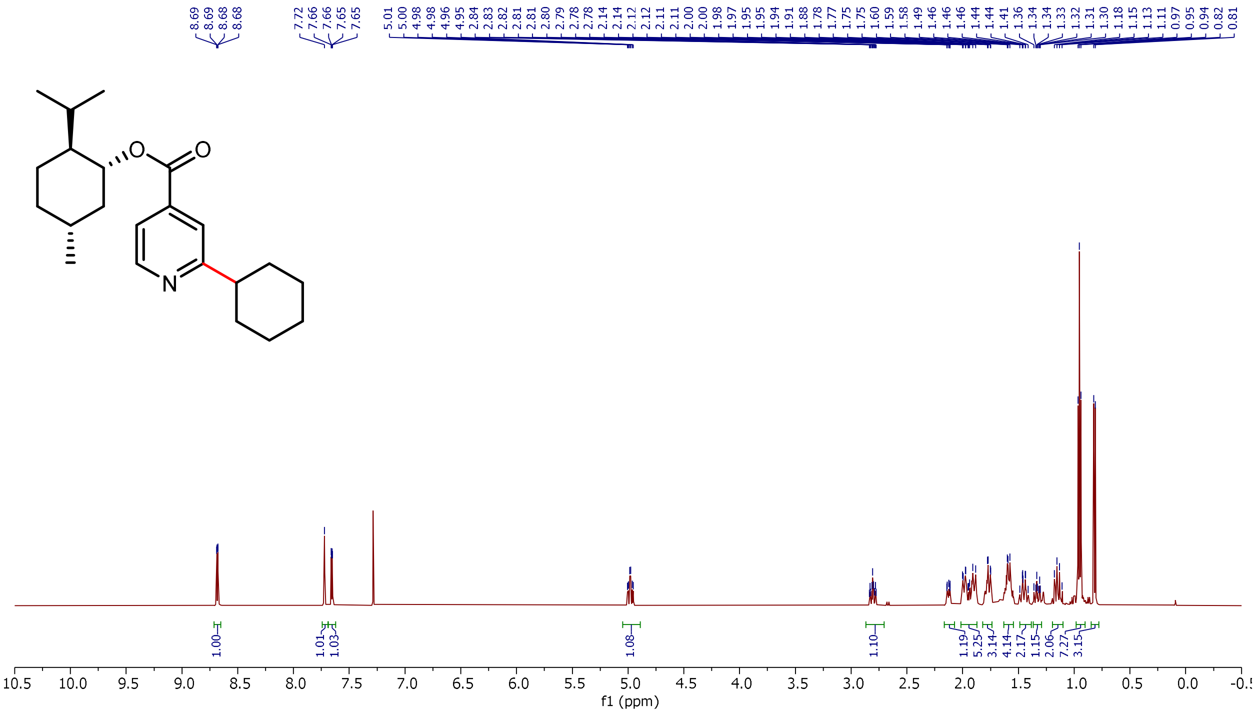


**Supplementary Figure 74b | $^{13}$C NMR (125 MHz, $CDCl_3$) of (1*R*,2*S*,5*R*)-2-isopropyl-5-methylcyclohexyl 2-cyclohexylisonicotinate (65)**

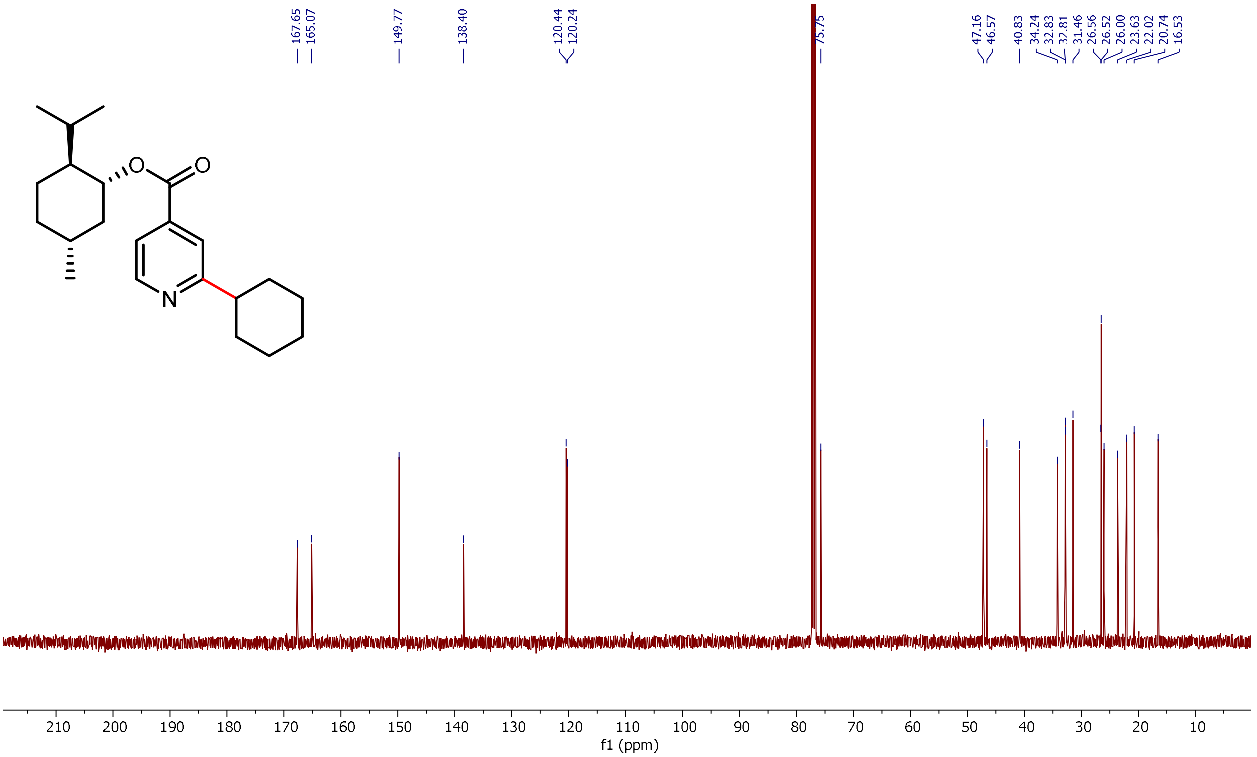

**Supplementary Figure 75a | $^{1}$H NMR (500 MHz, $CDCl_3$) of (*R*)-2-cyclohexyl-5-(1-methylpyrrolidin-2-yl)pyridine (66)**

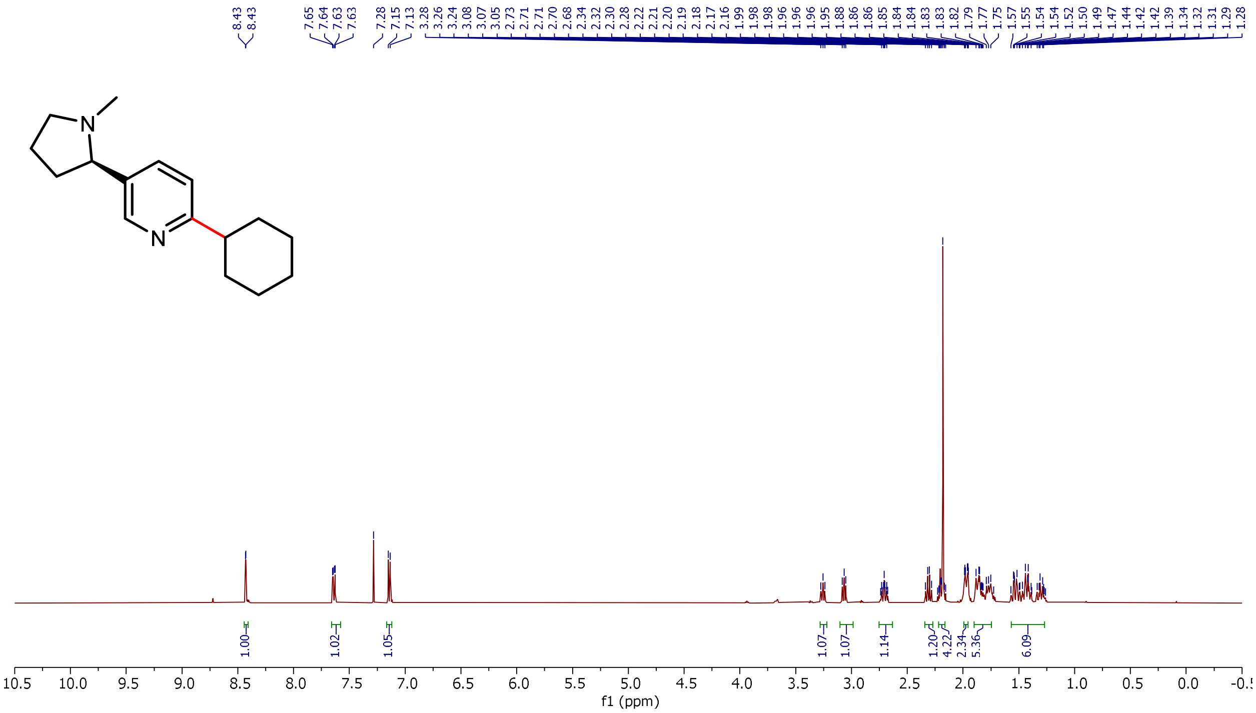


**Supplementary Figure 75b | $^{13}$C NMR (125 MHz, $CDCl_3$) of (*R*)-2-cyclohexyl-5-(1-methylpyrrolidin-2-yl)pyridine (66)**

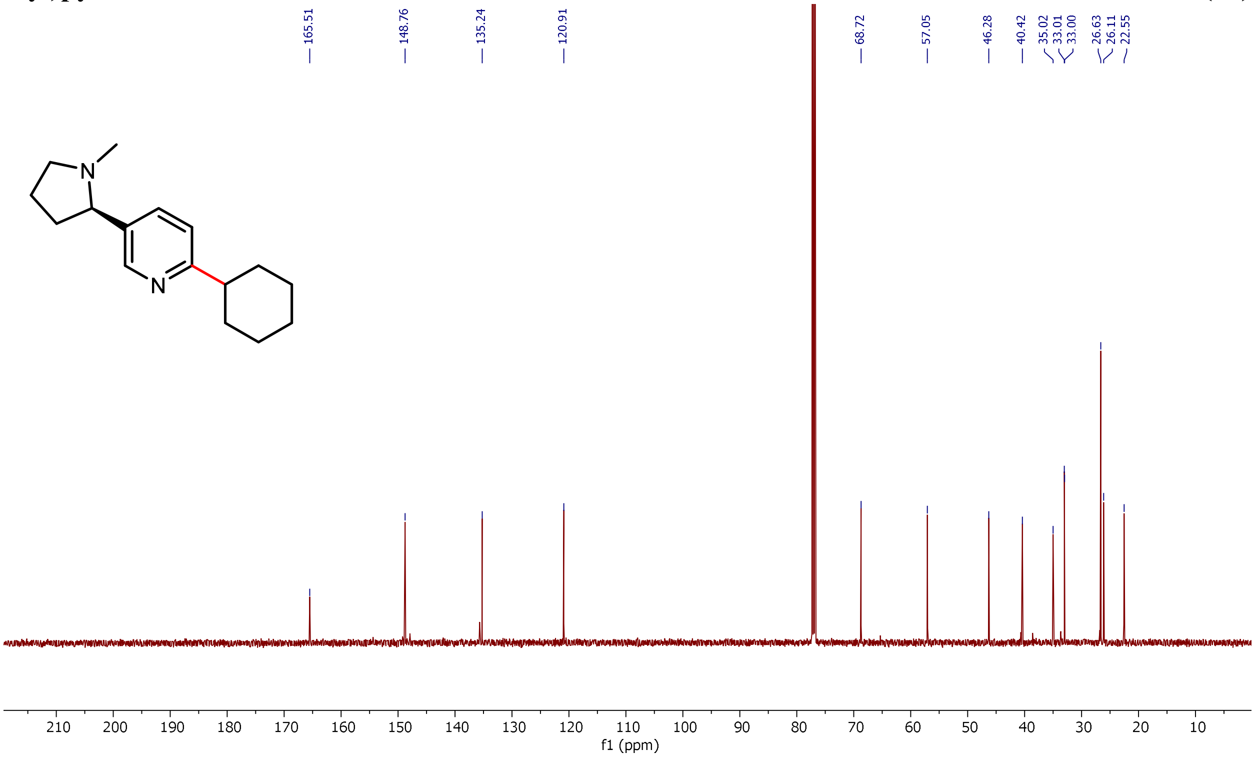

**Supplementary Figure 76a | $^1$H NMR (500 MHz, $CDCl_3$) of (*S*)-(2-cyclohexylquinolin-4-yl)((1*S*,2*S*,4*S*,5*R*)-5-ethylquinuclidin-2-yl)methanol (67)**

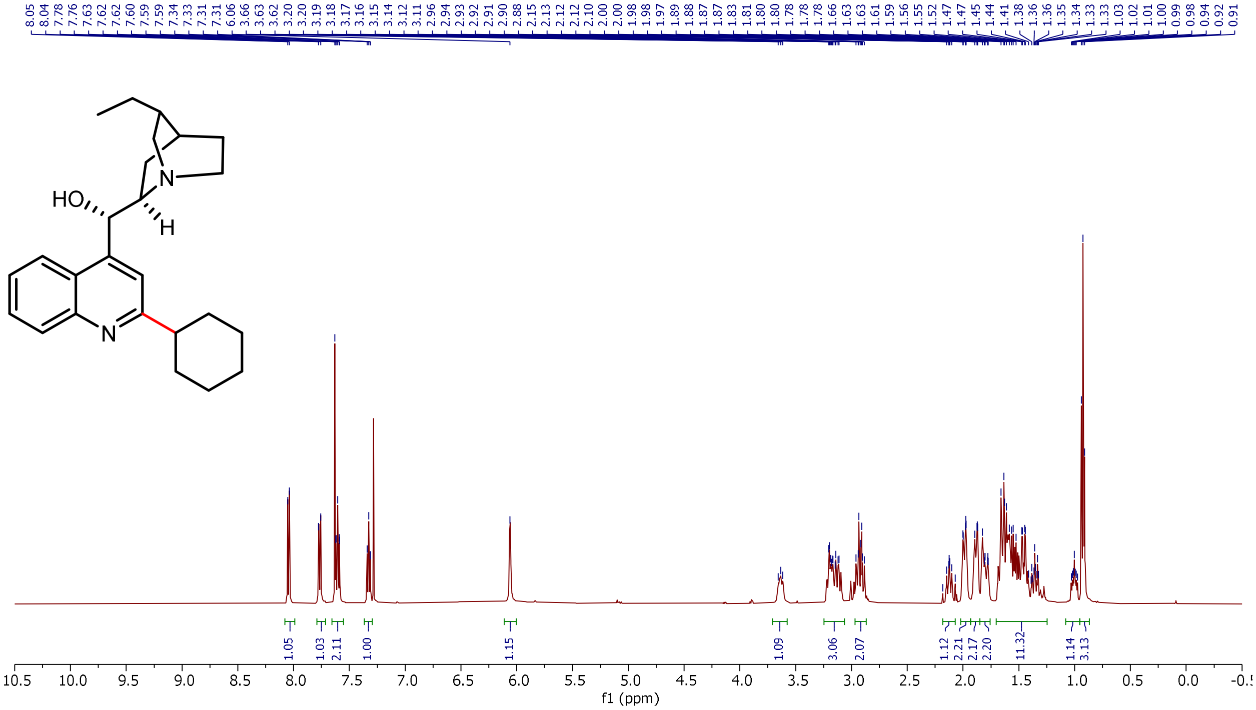


**Supplementary Figure 76b | $^{13}$C NMR (125 MHz, $CDCl_3$) of (*S*)-(2-cyclohexylquinolin-4-yl)((1*S*,2*S*,4*S*,5*R*)-5-ethylquinuclidin-2-yl)methanol (67)**

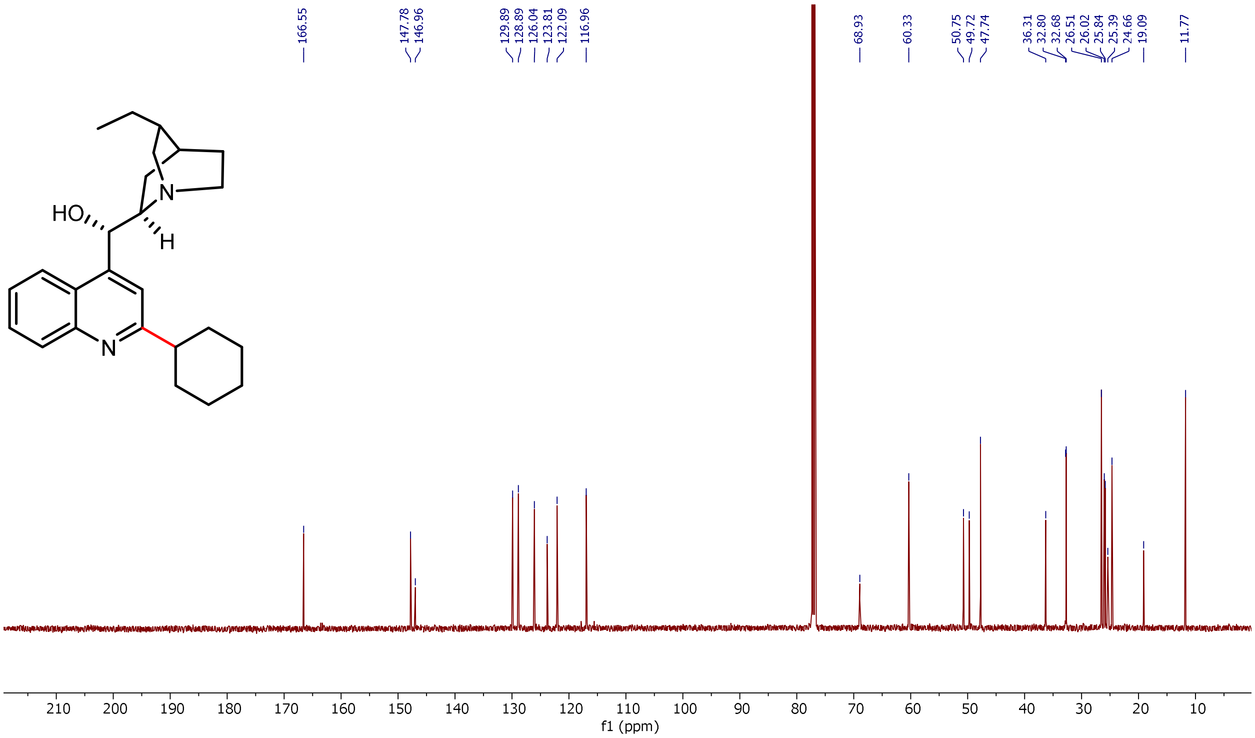

**Supplementary Figure 77a | $^{1}$H NMR (500 MHz, $CDCl_3$) of 1-(4-((1-cyclohexylisoquinolin-5-yl)sulfonyl)-1,4-diazepan-1-yl)ethan-1-one, undefined ratio of conformational isomers (68)**

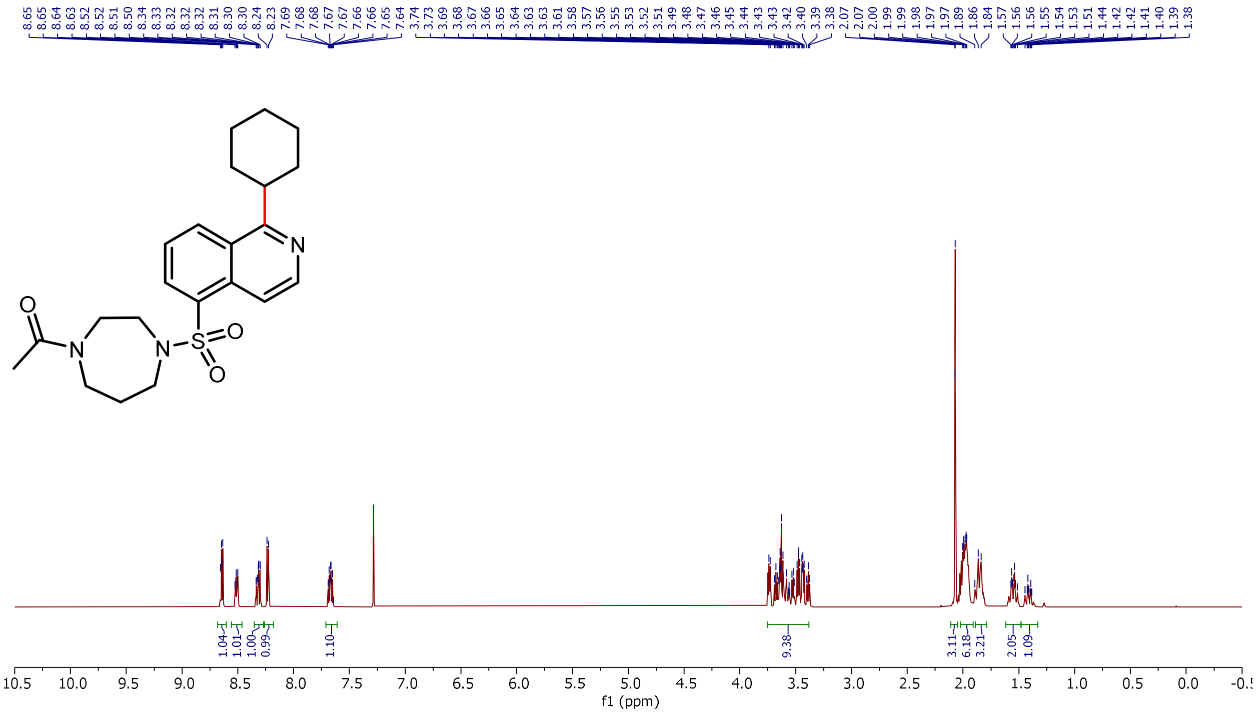


**Supplementary Figure 77b | $^{13}$C NMR (125 MHz, $CDCl_3$) of 1-(4-((1-cyclohexylisoquinolin-5-yl)sulfonyl)-1,4-diazepan-1-yl)ethan-1-one, undefined ratio of conformational isomers (68)**

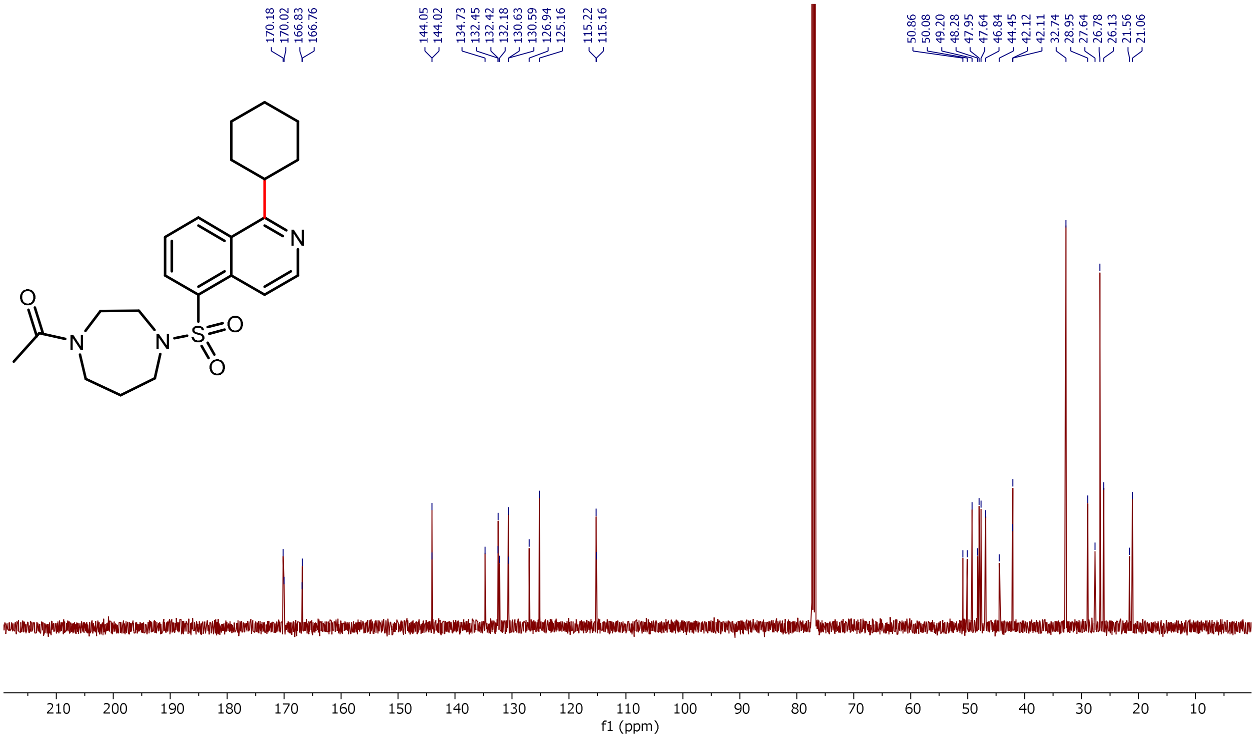

**Supplementary Figure 78a | $^{1}$H NMR (500 MHz, $CDCl_3$) of 3-(cyclohexylmethyl)-1,3-dimethylindolin-2-one (71)**

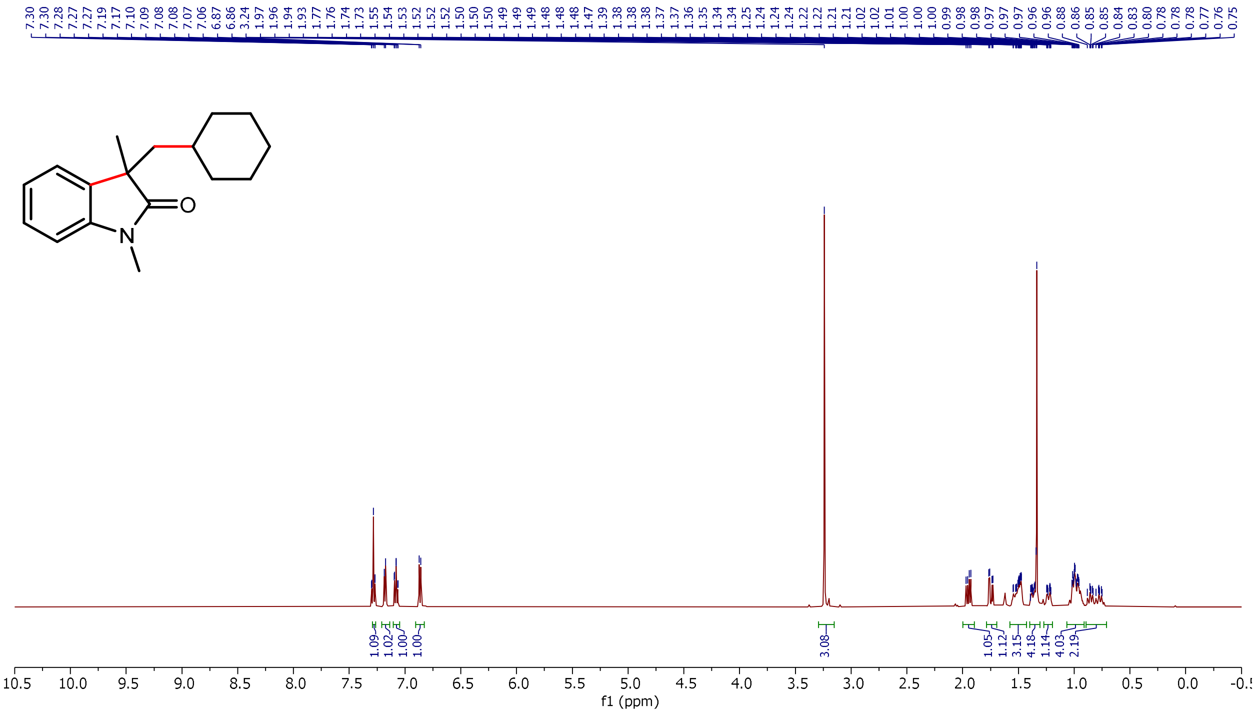


**Supplementary Figure 78b | $^{13}$C NMR (125 MHz, $CDCl_3$) of 3-(cyclohexylmethyl)-1,3-dimethylindolin-2-one (71)**

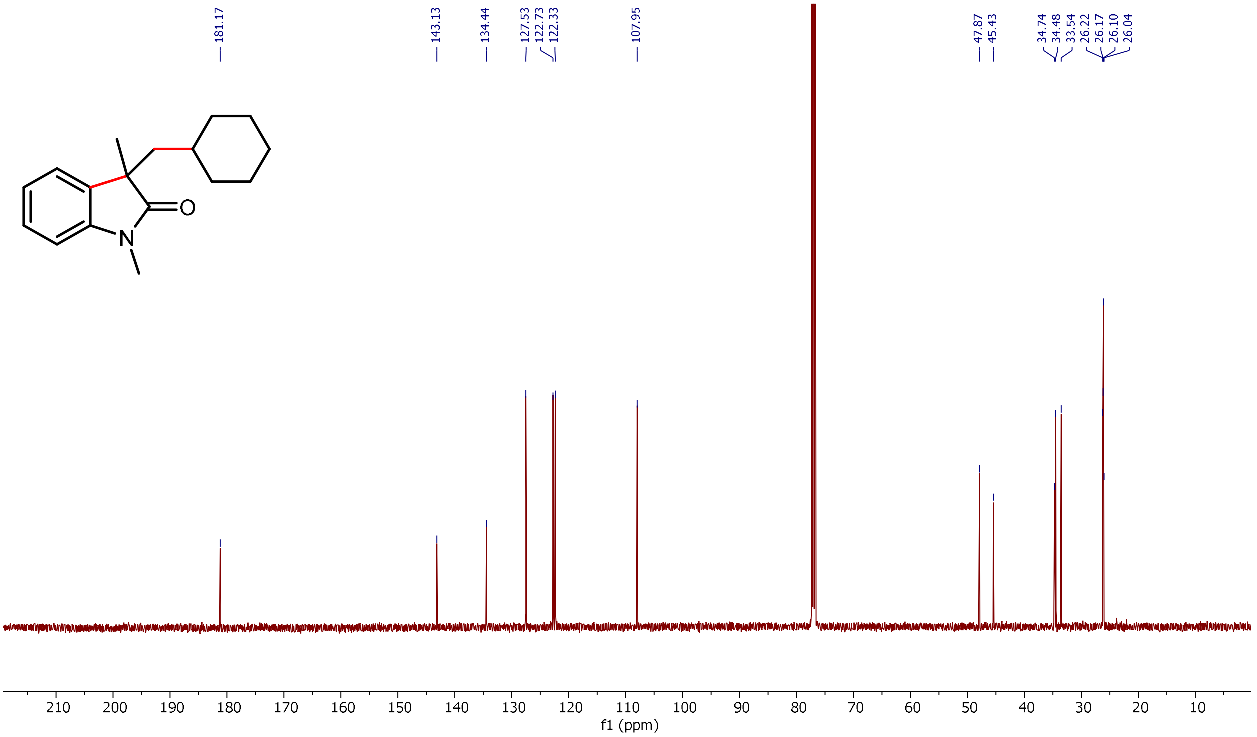

**Supplementary Figure 79a | $^{1}$H NMR (500 MHz, $CDCl_3$) of 3-(chloromethyl)-4-methyl-1-tosylpyrrolidine, 1:0.5 diastereomers (73)**

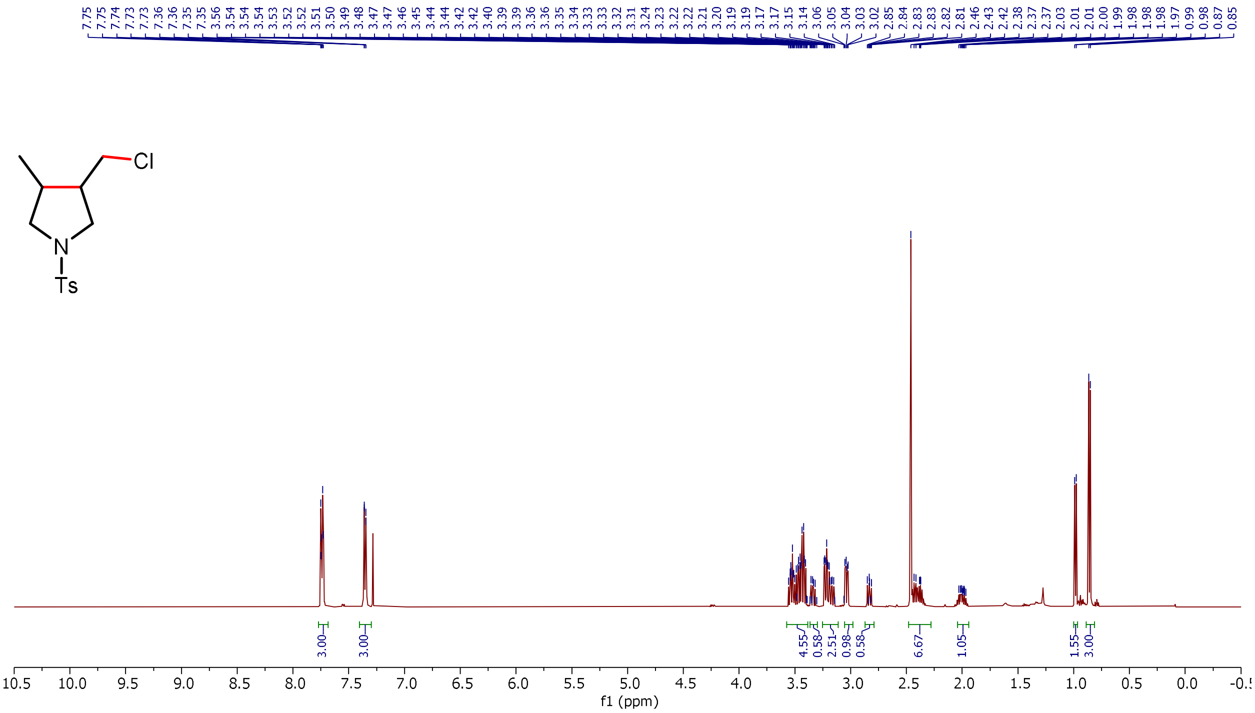

**Supplementary Figure 79b | $^{13}$C NMR (125 MHz, $CDCl_3$) of 3-(chloromethyl)-4-methyl-1-tosylpyrrolidine, 1:0.5 diastereomers (73)**

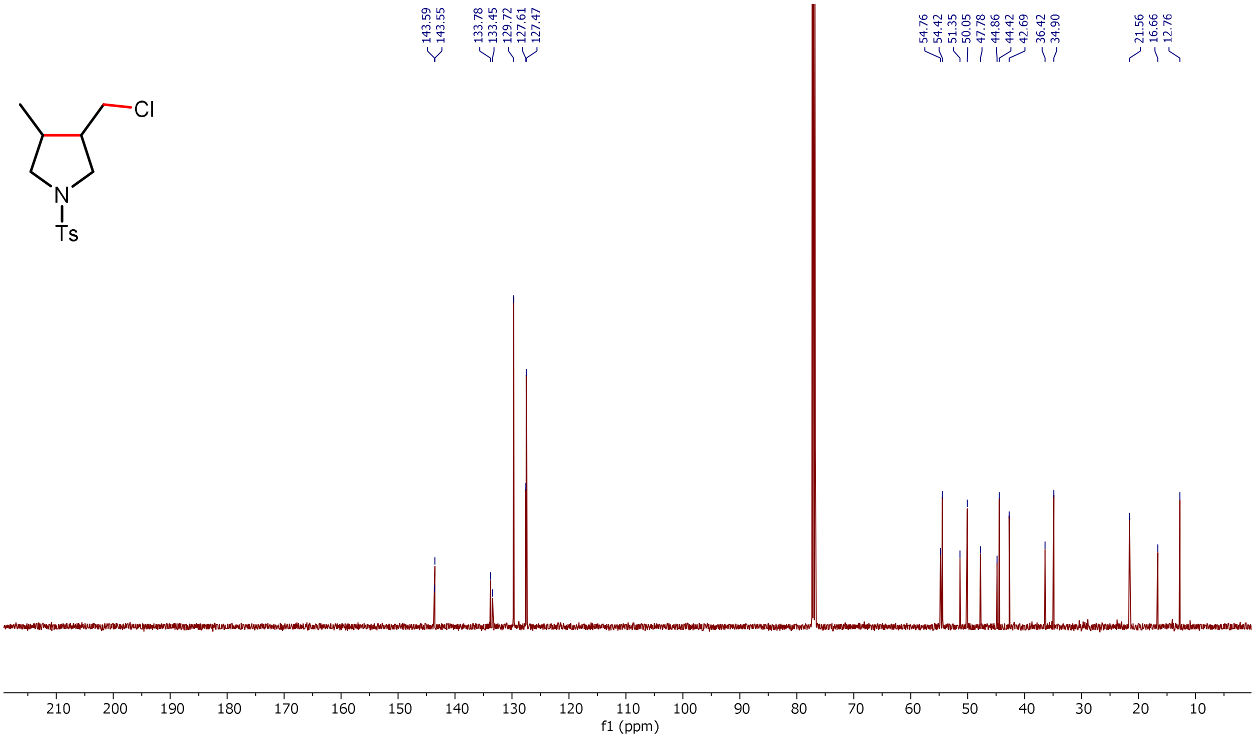

**Supplementary Figure 80a | $^1$H NMR (500 MHz, $CDCl_3$) of 4-(cyclohexyl-*d*$_{11}$)-2-phenylquinoline (3-*d*$_{11}$)**

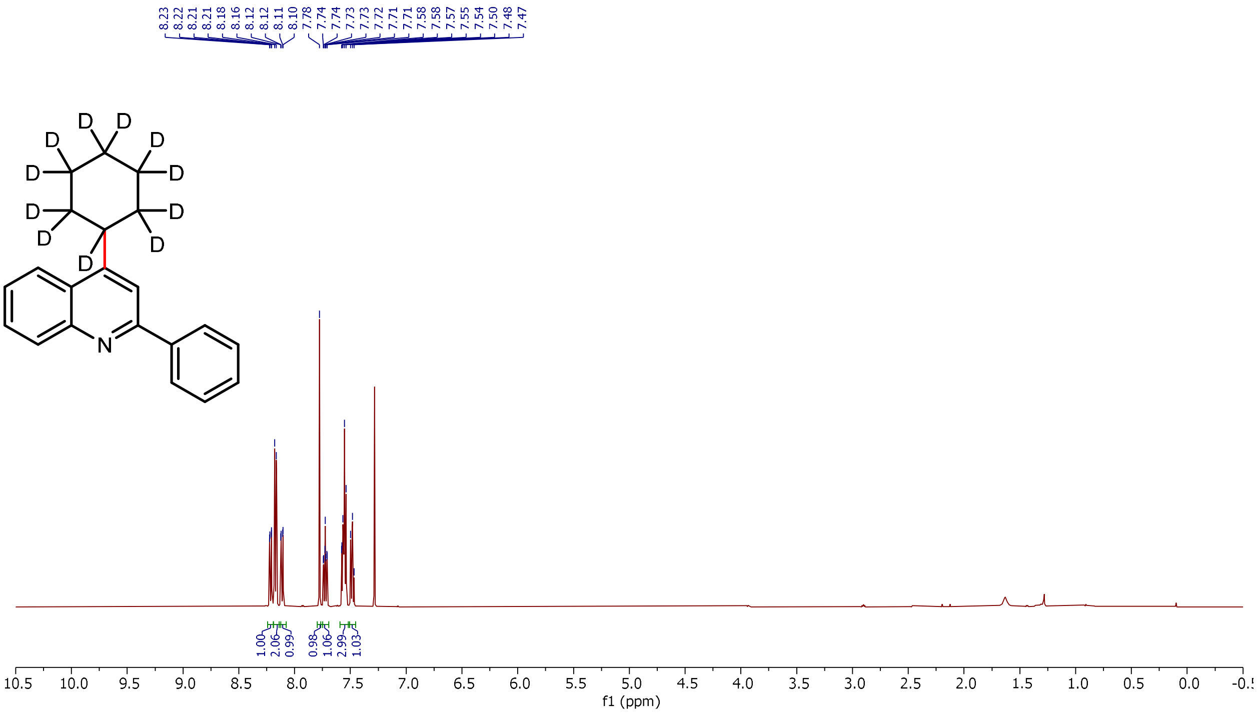

**Supplementary Figure 80b | $^{13}$C NMR (125 MHz, $CDCl_3$) of 4-(cyclohexyl-*d*$_{11}$)-2-phenylquinoline (3-*d*$_{11}$)**

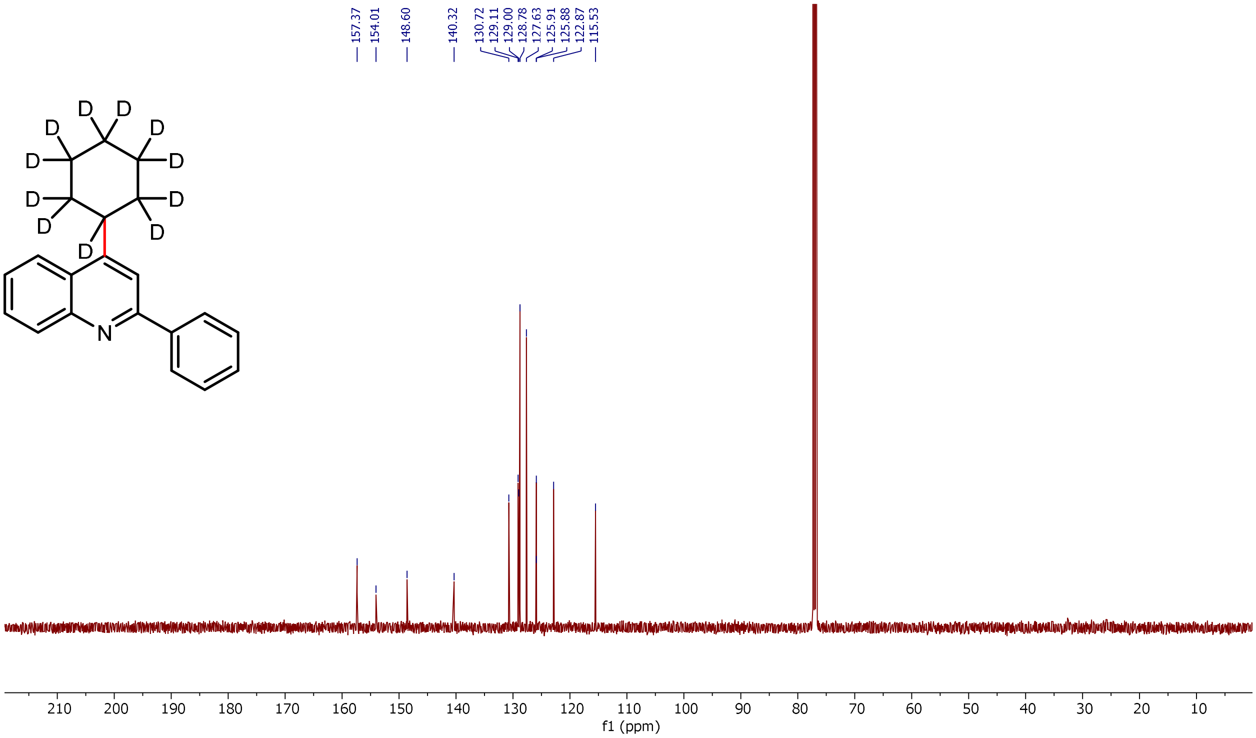

Supplementary Figure 80c, $^2$H NMR (77 MHz, $CDCl_3$) of 4-(cyclohexyl-$d_{11}$)-2-phenylquinoline (3-$d_{11}$)

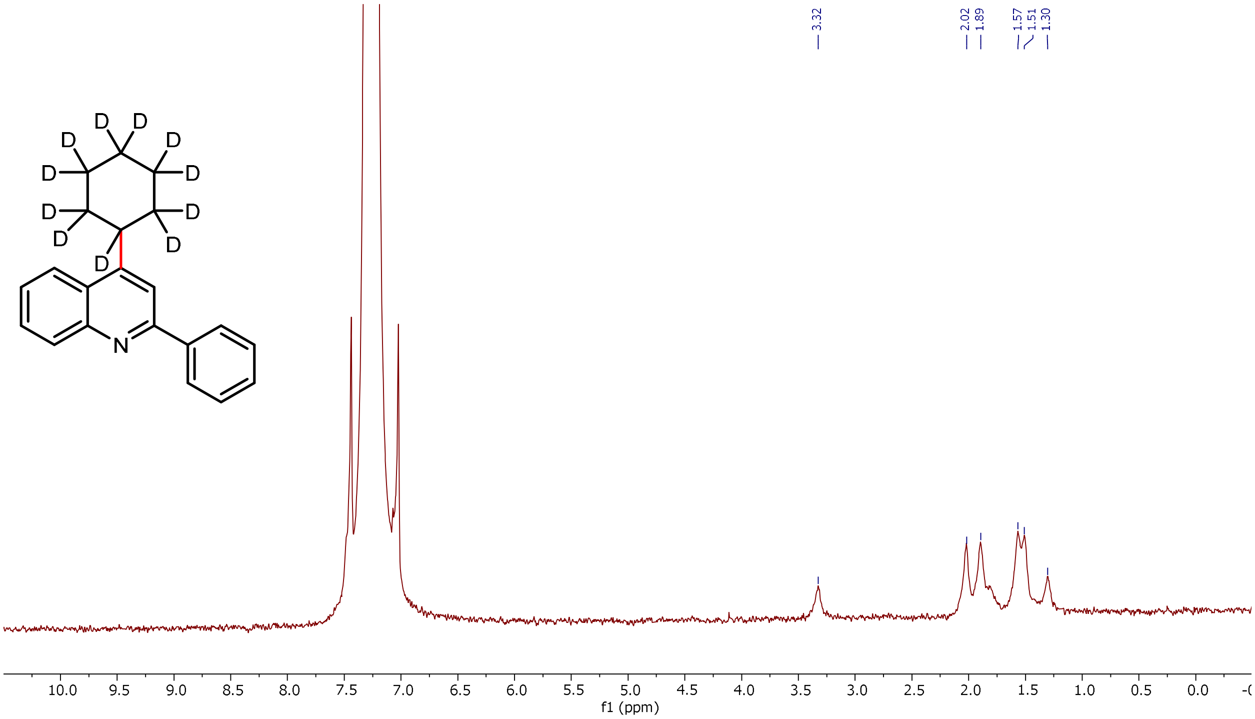

Supplement: Supplementary file 1 — Supplementary Information [file 41467_2021_24280_MOESM1_ESM.pdf]
